# Mixed positive and negative feedback loops drive diverse single-cell gene expression dynamics

**DOI:** 10.1038/s41467-026-75107-4

**Published:** 2026-08-17

**Authors:** Torkel E. Loman, Christian P. Schwall, Teresa Saez, Yujia Liu, James C. W. Locke

**Affiliations:** 1 Sainsbury Laboratory, University of Cambridge, Cambridge, UK; 2 Computer Science and AI Laboratory (CSAIL), Massachusetts Institute of Technology, Cambridge, MA USA; 3 Mathematical Institute, University of Oxford, Oxford, UK; 4 Present Address: Department of Biochemistry and Molecular Biology, University of Chicago, Chicago, IL USA

**Keywords:** Bacterial systems biology, Dynamical systems, Stochastic modelling, Single-cell imaging, Bacterial synthetic biology

## Abstract

Genetic circuits with only a few components can generate complex gene regulatory dynamics. Here, we combine stochastic modelling and single-cell time-lapse microscopy to map how a common circuit motif, the mixed positive/negative feedback loop, gives rise to diverse single-cell behaviours. Our minimal stochastic model of this motif reveals ten distinct classes of dynamic output, including stochastic pulsing, oscillations, and bistability. We systematically map how the circuit’s core parameters can be tuned to generate each of the behaviours. Experimental validation in two different mixed feedback circuits in the bacterium *Bacillus subtilis*, σ^B^ and σ^V^, confirms our model’s predictive power. Guided by our simulations, we are able to transition between selected dynamic behaviours by modulating in vivo parameters. Together, these results establish a quantitative framework for how mixed feedback loops generate diverse single-cell dynamics, improving our understanding of this common biological network motif and informing our efforts to engineer them for synthetic biology applications.

## Abstract (web summary)

A simple genetic circuit can generate complex gene regulatory dynamics. Here the authors use modelling and time-lapse microscopy to map how the mixed positive/negative feedback loop gives rise to ten distinct classes of single-cell behaviours.

## Introduction

Single-cell approaches have revealed that the outputs of gene circuits can be much more heterogeneous and dynamic than previously thought. Even small circuits with a few components can generate a wide range of regulatory dynamics, including bistability^1,2^, oscillations^3,4^, excitability^5,6^, and pulsatile activation^7,8^. Stochastic effects due to low molecule numbers often shape when and how these behaviours appear in individual cells^9,10^. In some contexts, this noise can be harnessed to generate functional phenotypic heterogeneity^11–13^. Yet despite this rich phenomenology, translating mechanistic circuit descriptions into general, testable predictions about trajectory-level behaviours in the noisy single-cell regime remains challenging.

Here, we focus on mixed positive/negative feedback loops, a widespread regulatory motif found in processes such as circadian rhythms^14^, neural signalling^15^, bacterial competence^16^, the cell cycle^17^, and DNA damage repair^18^. These architectures have also been proposed as useful regulatory motifs for synthetic biology applications^19^, and have a long history in theoretical biology, including analyses of switch-like and oscillatory behaviours^20,21^ and noise-induced dynamics^22^. Despite their broad relevance, a unifying analysis of the trajectory-level behaviours these circuits produce, and how these depend on circuit properties in the noisy regime, has yet to be established.

**Fig. 1:**
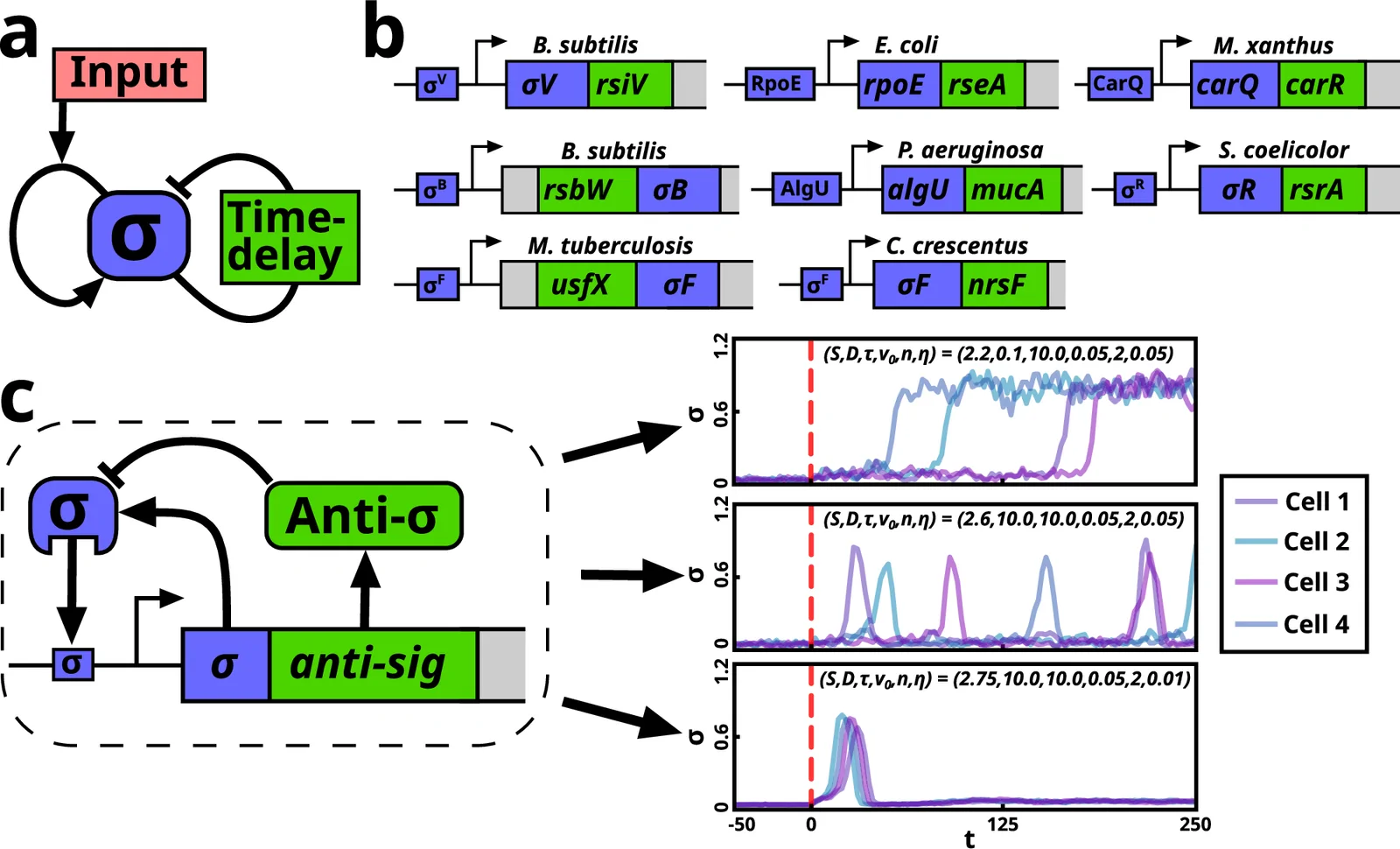
A general model of a mixed positive/negative feedback loop can reproduce experimentally observed sigma factor dynamics. **a** Schematic of the general mixed positive/negative feedback loop model. Here, a single component (*σ*) both activates and deactivates its own production. The activation depends on the presence of an input, and the deactivation is subject to a time delay. **b** The mixed positive/negative feedback loop is a common motif among alternative sigma factor circuits. Shown are simplified operon structures of alternative sigma factor circuits in a range of bacterial species, all consisting of a mixed positive/negative feedback. Anti-sigma factors (that repress sigma factor activity) are depicted in green, and sigma factors are depicted in blue. **c** Model simulations can capture known activation behaviours, including heterogeneous activation (top, as observed in e.g., σ^V^^37^), stochastic pulsing (middle, as observed in e.g., σ^W^^8^), and single pulse dynamics (bottom, as observed in σ^B^ under environmental stress^40^). Source code used to generate this figure can be found in the provided GitLab repository.

To address this, we develop a minimal stochastic model of a mixed positive/negative feedback loop (Fig. 1a). Stochastic modelling is essential for capturing intrinsic noise from low molecule numbers and extrinsic fluctuations from cellular processes like growth or energy availability^23–25^. While prior models have explained behaviours such as excitability in bacterial competence^16,26^ or circadian oscillations^27^, these efforts are often tailored to particular biological implementations or focus on a limited subset of behaviours and parameters. By directly modelling the effective properties of mixed feedback circuits, such as feedback strength, time delay, noise, and ultrasensitivity, we derive testable predictions about the range of trajectory-level behaviours these motifs can generate.

A central aspect of our approach is that we combine parameter scans in the minimal stochastic model with targeted perturbations in vivo. We systematically vary a small set of biologically interpretable parameters in the model and map how qualitative behaviours and transitions emerge across parameter space. We then use these maps to guide experiments that modulate corresponding circuit properties and test the predicted transitions at the single-cell level. This strategy builds on experimental precedents in which dynamical regimes were explored by varying circuit inputs and parameters in vivo, including in *Bacillus subtilis* competence^24,26,28^.

To validate our model predictions, we turn to bacterial alternative sigma factor circuits. Alternative sigma factors direct RNA polymerase to specific downstream targets and control key processes such as stress response, pathogenicity, and chemotaxis^29^. The number of alternative sigma factors varies between bacterial species, ranging from 0 (as found in e.g., *Mycoplasma pneumoniae*) to over 63 (as found in *Streptomyces coelicolor*)^30–32^. Crucially, many alternative sigma factor circuits share a conserved mixed feedback motif: the sigma factor activates an operon that encodes both itself and an anti-sigma factor that inhibits its function (Fig. 1b)^33–35^. This simple, well-characterised, structure makes sigma factor circuits ideal systems for dissecting how mixed feedback architecture shapes dynamic behaviour.

Single-cell time-lapse fluorescence microscopy has revealed diverse, and often stochastic, gene expression dynamics across alternative sigma factor circuits. Using microfluidic devices such as the mother machine^36^, studies have uncovered behaviours including heterogeneous activation (σ^V^ in *B. subtilis*^37^), stochastic pulsing (σ^M^, σ^W^, σ^X^, σ^D^, σ^B^ in *B. subtilis* and RpoS in *E. coli*^8,38,39^), and a single response pulse (σ^B^ in *B. subtilis*^40^). Here we use σ^V^ as a model system for heterogeneous activation and σ^B^ as a model system for pulsatile dynamics (see Supplementary Notes 1 and Supplementary Figs. 1, 2 for full network details).

Both systems contain mixed feedback architectures, with different levels of complexity. σ^V^ is activated by lysozyme stress via cleavage of its anti-sigma factor RsiV^41–47^, and in turn activates its own operon containing both *sigV* and *rsiV* (Supplementary Fig. 1). σ^B^ is regulated through a partner-switching mechanism involving its anti-sigma factor RsbW and the anti–anti-sigma RsbV^48–52^, forming a more complex feedback structure (Supplementary Fig. 2). Despite their biological differences, both systems share a core regulatory motif in which a sigma factor promotes expression of both itself and its anti-sigma factor.

In this work, we develop a general stochastic model of a mixed positive/negative feedback loop. The model predicts ten distinct dynamic behaviours, and maps the transitions between them across a small set of biologically meaningful parameters. Using genetically rewired σ^V^ and σ^B^ circuits in *B. subtilis* (Supplementary Figs. 3, 4), we experimentally validate the predicted transitions between behaviours. Together, this provides a compact framework linking circuit properties to noisy single-cell dynamics, and shows how native or engineered systems can be tuned to generate target behaviours.

## Results

### A general mixed positive/negative feedback loop model can reproduce experimentally observed sigma factor dynamics

To explore the range of dynamics that mixed feedback loop genetic circuits can generate, we developed a minimal stochastic model (Methods, Fig. 1a). In this work, we apply it to sigma factor networks, biologically relevant systems that frequently implement mixed feedback loops (Fig. 1b). The model is implemented as a stochastic differential equation (SDE) tracking the concentration of a single component (*σ*), which both activates and represses its own production through a Hill-type regulatory function. The self-repression is subject to a time delay. In the context of sigma factors, this delay can be motivated either by the anti-sigma factor undergoing additional steps before it can interact with the sigma factor^53^, or due to the presence of additional feedback mechanisms^54^.

The initial formulation of our model depended on 9 parameters. Because our aim was to understand general qualitative behaviours rather than fit specific datasets, we performed nondimensionalisation, reducing the model to six biologically interpretable parameters (Methods, Supplementary Notes 2, Supplementary Tables 1, 2). These are: the system’s degree of self-activation (*S*), self-deactivation (*D*), the time delay in the self-deactivation (*τ*), base operon activity (*v*_*0*_), ultrasensitivity (*n*), and noise amplitude (*η*). The deterministic version of the model, which excludes the noise parameter *η*, is given by:

1 $$\frac{{{\mathrm{d}}}\sigma (t)}{{{{\mathrm{d}}}t}}={v}_{0}+\frac{{(S\cdot \sigma (t))}^{n}}{{(S\cdot \sigma (t))}^{n}+{(D\cdot \sigma (t-\tau ))}^{n}+1\,}-\sigma (t)$$

We implemented noise using the chemical Langevin equation (CLE)^55^, a well-established method for modelling noise within gene regulatory networks^56^. By representing the system as a stochastic differential equation (SDE), the CLE enables fast stochastic simulations when compared to the more exact Gillespie algorithm^57,58^. Our results were not specific to the CLE, as we verified that simulating noise through the Gillespie algorithm generated equivalent results (Supplementary Notes 3, Supplementary Figs. 5, 6).

We implemented the delay, *τ*, as a distributed delay using the linear chain trick (Supplementary Notes 4.1, Supplementary Figs. 7, 8)^59,60^. This enabled us to model the system as an SDE, rather than as a Stochastic Delay Differential Equation (SDDE), which is advantageous for model simulation and analysis (Methods). For completeness, we also implemented an SDDE version of the model and confirmed it reproduces key results (Supplementary Notes 4.2, Supplementary Figs. 9, 10, Supplementary Table 3). We note that our minimal mixed feedback loop model captures the range of sigma factor response behaviours reported in the literature, including heterogeneous activation, stochastic pulsing, and single response pulse (Fig. 1c)^8,37,40^. Finally, we also replicated our main results in two different alternative implementations of the mixed positive/negative feedback loop, demonstrating that they are not unique to the specific implementation described above (Supplementary Notes 5, Supplementary Tables 4, 5, and Supplementary Figs. 11, 12).

### The model generates ten distinct response behaviours

**Fig. 2:**
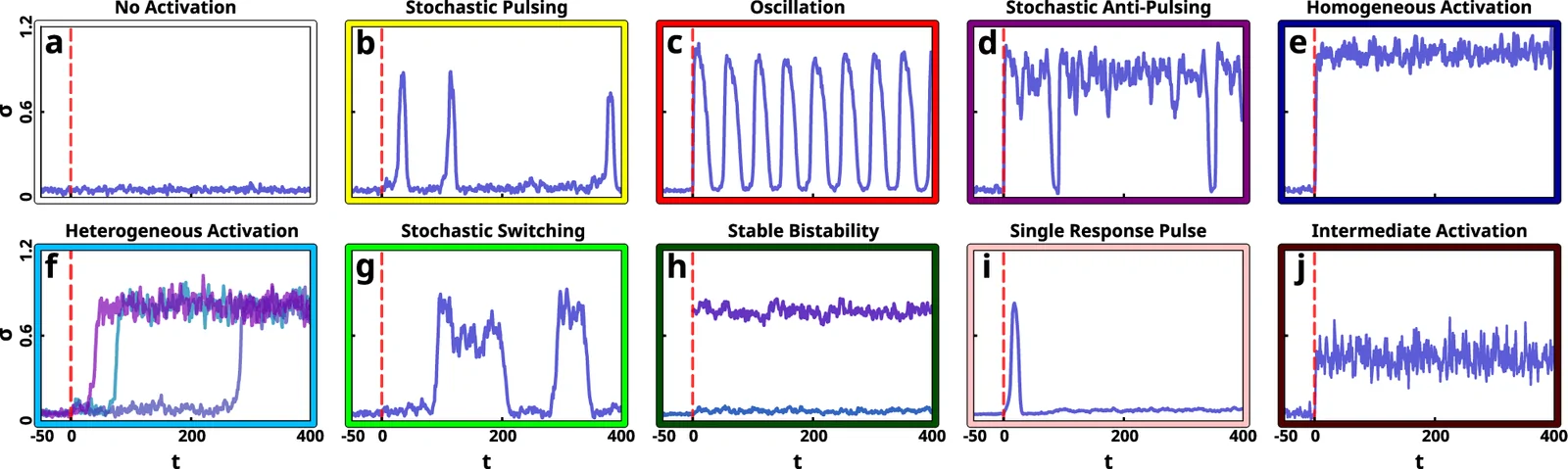
The model generates 10 distinct response behaviours. Simulations for 10 different parameter sets, demonstrating the 10 different behaviours the model can achieve. Initially, the system remains inactive; upon triggering the input (marked by the red dashed lines), distinct behaviours emerge: **a** No activation. **b** Stochastic pulsing. **c** Oscillation. **d** Stochastic anti-pulsing. **e** Homogeneous activation. **f** Heterogeneous activation (three different simulations displayed). **g** Stochastic switching. **h** Stable bistability. This panel depicts two simulations. The first (dark blue) is initiated at the initial (inactive) state at the same time as the simulations in the other panels. The second (purple) is initiated at the time of the input (dashed red line) in the active state. There is no intrinsic way for the system to switch between these two states (however, it could happen by e.g., the addition of exogenous *σ*). **i** Single response pulse. **j** Intermediate activation. Source code used to generate this figure can be found in the provided GitLab repository.

To explore the range of behaviours the model can exhibit, and to understand how these depend on its parameters, we developed a behaviour classification scheme. The scheme is primarily based on steady state analysis, where we note that the system can have either a fixed point in an inactive state, an active state, both, or a single fixed point of intermediate activity (Supplementary Notes 6, Supplementary Figs. 13, 14). Asymptotically, it will approach one of the stable steady states (if one exists). However, perturbations, either due to noise or onset of the input, can cause a transition to another state. By enumerating all possible combinations of these transitions (but also accounting for other dynamic features, e.g., using Fourier analysis to distinguish oscillations), we find a total of ten distinct behavioural classes (Fig. 2, Supplementary Notes 7, Supplementary Figs. 15–17). By simulating the model across a large set of randomised parameter values and checking that each simulation aligned with one of these ten classes, we confirmed that these classes are robust (Supplementary Figs. 18, 19). Rare additional behaviours (e.g., such as heterogeneous intermediate activation) that can be considered subclasses of these 10 are discussed in Supplementary Notes 7.4.

Through our classification scheme, we identified ten distinct response behaviours predicted by the model (Fig. 2):

- No activation: The system remains inactive. In the absence of the input, this is also the system’s default behaviour (Fig. 2a, Supplementary Fig. 20).
- Stochastic pulsing: The system exhibits randomly distributed pulses of activity in time, from an otherwise inactive state (Fig. 2b, Supplementary Fig. 21).
- Oscillation: The system alternates between low and high activity in a periodic manner (Fig. 2c, Supplementary Fig. 22).
- Stochastic anti-pulsing: The system activates in response to the input, but displays randomly distributed pulses of inactivity over time (Fig. 2d, Supplementary Fig. 23).
- Homogeneous activation: In response to the input, the system transitions from an inactive to an active state. The response times are instantaneous (and thus homogeneous across simulations) (Fig. 2e, Supplementary Fig. 24).
- Heterogeneous activation: In response to the input, the system transitions from an inactive to an active state. The response times are random (and thus heterogeneous across simulations) (Fig. 2f, Supplementary Fig. 25).
- Stochastic switching: The system randomly alternates between an inactive and an active state (Fig. 2g, Supplementary Fig. 26).
- Stable bistability: The system has an inactive and an active state, but cannot switch between them intrinsically. An extrinsic way to induce such a switch would be to add additional σ from an external source (Fig. 2h, Supplementary Fig. 27).
- Single response pulse: The system exhibits a single pulse of activity at the input’s onset, but no significant activity thereafter (Fig. 2i, Supplementary Fig. 28).
- Intermediate activation: Following input onset, the system transitions to and remains in a state of intermediate activity (Fig. 2j, Supplementary Fig. 29). Unlike for the homogeneous activation behaviours, mean activity in the steady state can be modulated by tuning *S* or *D* (Supplementary Notes 8.10). While the activation times are typically homogeneous, certain low-*D* valued regions display heterogeneous intermediate activation (Supplementary Notes 7.4).

These behaviours include all previously observed dynamics among bacterial sigma factors (a single response pulse, stochastic pulsing, heterogeneous activation, and homogeneous activation)^8,37,38,40^, as well as several novel behaviours. Detailed descriptions of each behaviour are provided in the supplementary information (Supplementary Notes 8).

### Systematic mapping of behaviours reveals their dependency on model parameters

**Fig. 3:**
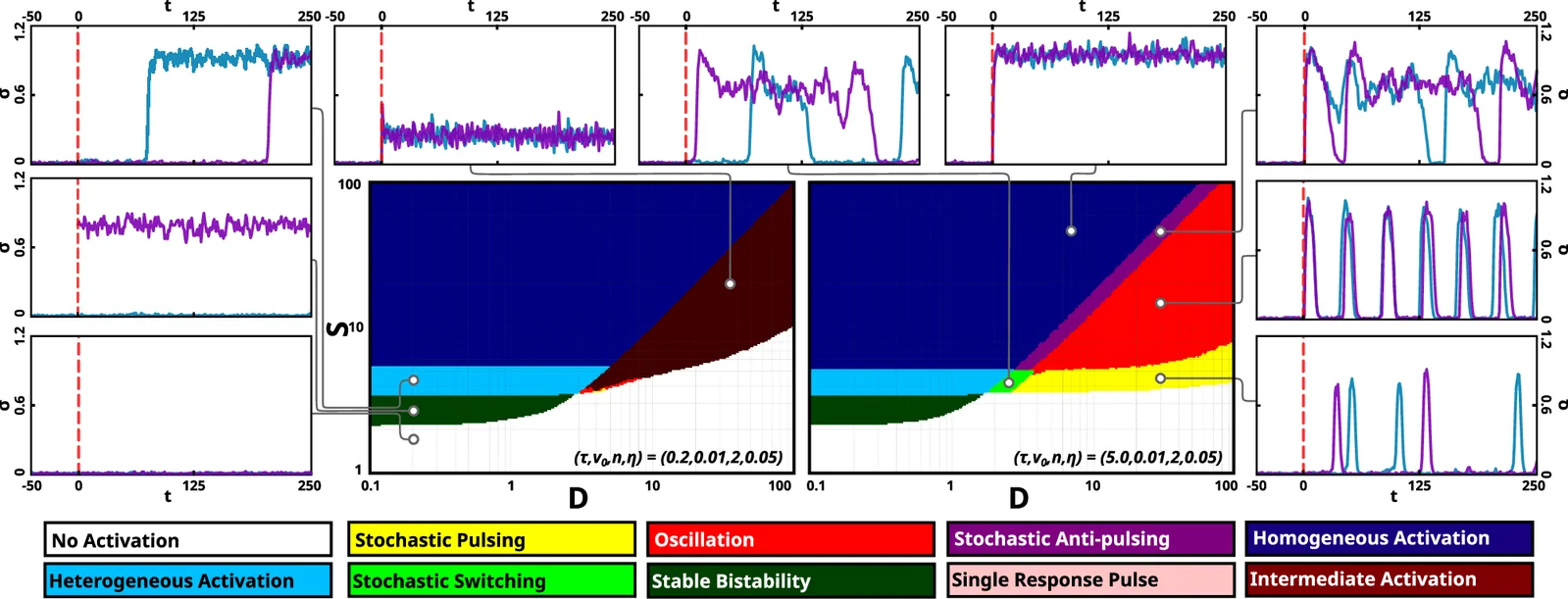
Mapping system behaviours across parameter space reveals distinct dynamic regimes. We applied our parameter set behaviour classifier to each point in the 6 d parameter space of the model and visualised 2 d slices in log-scale S-D space, with the remaining parameters, *v*_*0*_, *n*, and *η* held constant. The two maps shown here differ only in the value of the time delay *τ*: *τ = 0.2* (left) and *τ = 5.0* (right). Each point is coloured by the classified dynamic behaviour, and example trajectories from selected points illustrate representative single-cell simulations. While the exact boundaries shift with *τ*, both maps exhibit a common structure: a *D≲1* region with bistable and switching behaviours, and a *D ≳ 1* region that supports either intermediate activation (left) or oscillatory and pulsing dynamics (right). In these two maps, the transition occurs at around *D ≈ 2*, and in general, the transition depends on *v*_*0*_ but is typically close to 1. This general division by *D* is robust across parameter choices and is supported by stability analysis (Supplementary Fig. 30). The effects of varying *τ*, *v*_*0*_, *n*, and *η* are further explored in Supplementary Figs. 31–35 (which show additional behaviour maps). Finally, we note that quantitative properties of each behaviour change within their respective regions. E.g. the frequency of pulses within the stochastic pulsing (yellow) region increases with *S*. Source code used to generate this figure can be found in the provided GitLab repository.

Next, we investigated how the system’s behaviour depends on its six parameters, (*S*, *D*, *τ*, *v*_*0*_, *n*, and *η*). To do this, we developed a heuristic classifier that takes a parameter set as input and assigns it to one of the 10 behavioural classes (Supplementary Notes 9, Supplementary Fig. 15). The parameter set classifier uses a decision tree which in each node typically performs simulations initialised at the model’s steady states and measures the transition times to other steady states (Supplementary Fig. 17). We confirmed the accuracy of the classifier by empirically validating its classifications across a large random sample (Supplementary Figs. 18, 19). Applying the classifier across the six-dimensional (6 d) parameter space allowed us to construct a map showing which behaviour occurs for each parameter combination (Supplementary Notes 9). To visualise how individual system properties influence resulting dynamic behaviours, we plotted 2 d slices of this parameter space while holding the remaining four parameters constant (Fig. 3, Supplementary Figs. 30–35).

When examining parameter maps for various values of *τ*, *v*_*0*_, *n*, and *η*, we found that they can be broadly divided into two regions: *D ≲ 1* and *D ≳ 1* (Supplementary Fig. 30). Each region is characterised by a distinct behavioural transition as the system’s self-activation strength (*S*) increases. We note that *S* is a composite parameter which, after nondimensionalisation, also incorporates the system’s input strength. Hence, *S* can be tuned by either modifying the self-activation strength or by varying the input. In the *D ≲ 1* region, the model exhibits behaviours associated with bistability, such as stochastic switching, stable bistability, and heterogeneous activation. This is natural, as bistability can arise by a self-activation loop only, and this part of the circuit dominates for *D ≲ 1*. Conversely, the *D ≳ 1* region exhibits behaviours related to oscillations and excitability, including stochastic pulsing, oscillations, and stochastic anti-pulsing, although it can also exhibit intermediate activation under some conditions (Supplementary Notes 9, Supplementary Figs. 31–35). Again, these are behaviours which typically require some form of negative feedback, and are hence only expected when *D* is not too small.

Next, we characterised how the behaviours depend on the parameters *τ*, *v₀*, *n*, and *η* by measuring their effect on the prevalence of each behaviour (Supplementary Figs. 20–29). Generally, the time delay *τ* had the largest effect on system behaviour (after *S* and *D*), being especially influential in the *D* ≳ 1 region (Supplementary Notes 10, Supplementary Table 6). Figure 3 illustrates this effect: two maps are shown in which all parameters are held constant except *τ*, which is 0.2 in the left panel and 5.0 in the right. At low τ, the *D ≳ 1* region supports intermediate activation, while longer delays give rise to stochastic pulsing and oscillations. In contrast, *τ* has minimal impact in the *D ≲ 1* region, where weak self-deactivation renders the delay less consequential. Conversely, *v*_*0*_ (the base activity of the operon) had only minor effects on the *D ≳ 1* region. Meanwhile, in the *D ≲ 1* region, all non-trivial behaviours (i.e., behaviours except for no activation and homogeneous activation) grew less prevalent as *v*_*0*_ grew larger (Supplementary Notes 8, Supplementary Figs. 33, 34). Almost all behaviours were affected by *η* (the noise amplitude), while *n* (the degree of system ultrasensitivity) had the least pronounced effect on the system’s behaviour (Supplementary Notes 8, Supplementary Figs. 32–35).

**Fig. 4:**
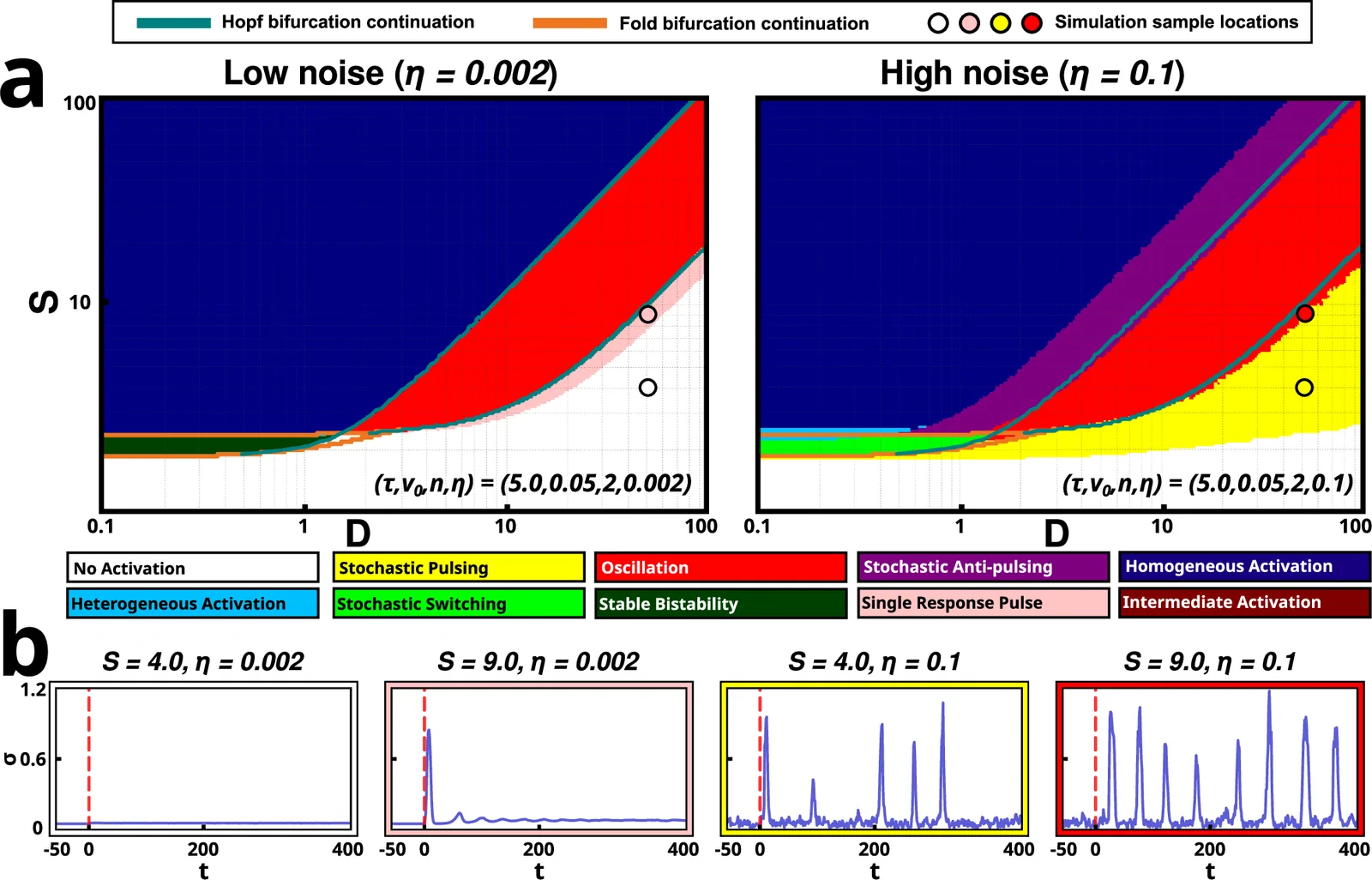
Stochasticity enables additional behaviours and shifts behavioural boundaries. **a** Behaviour maps in (*S*,*D*) space at fixed (*τ,v*_*0*_*,n*) = (*5.0,0.05,2*), shown for low noise (*η = 0.002*) and high noise (*η = 0.1*). Teal and orange curves indicate the Hopf and fold bifurcation continuations of the corresponding deterministic system (i.e., *η = 0.0*). At low noise, behavioural boundaries align closely with these bifurcation curves. At high noise, the boundaries shift. Furthermore, behaviours such as stochastic pulsing and stochastic anti-pulsing occur. **b** Simulations at *S = 4* and *S = 9* for the low- and high-noise regimes (parameter sets marked by coloured dots in **a**, with *D* = 50.0). Increasing noise converts no activation at *S = 4* into stochastic pulsing, and converts a single response pulse at *S = 9* into oscillations. Notably, in the latter case, the corresponding deterministic system does not exhibit a periodic orbit. An expanded version of this figure can be found in Supplementary Fig. 36. Source code used to generate this figure can be found in the provided GitLab repository.

Our behaviour maps also permit us to directly observe how noise affects behaviour space (Fig. 4). Most notably, we can see how noise-dependent behaviours, such as stochastic pulsing and switching, emerge as noise increases. Furthermore, we observe a systematic “shift” between the behavioural boundaries and what is predicted by deterministic steady state analysis alone. Here, at low noise levels, behavioural transitions coincide well with the bifurcation points of the deterministic system. However, as the noise amplitude increases, the “effective” bifurcation points, such as those marking the emergence of oscillatory behaviours, increasingly move in behaviour space (Fig. 4, Supplementary Fig. 36).

Finally, we note that σ^V^ and σ^B^ naturally occupy distinct regions of parameter space, with σ^V^ exhibiting heterogeneous activation, which occurs in the *D ≲ 1* regime, and σ^B^ exhibiting stochastic pulsing, which occurs in the *D ≳ 1* regime. This separation allows us to explore different parts of the behavioural map by selectively tuning parameters in each system. In the following sections, we use σ^V^ to probe transitions across the *D* axis and σ^B^ to examine transitions driven by changes in *S*, experimentally validating our model’s predictions in both cases.

### The predicted ability to transition between D ≲ 1 and D ≳ 1 behavioural domains is validated in the σ^V^ system

We first experimentally tested the transitions predicted for the bistability-dominated *D ≲ 1* region, using the *B. subtilis* alternative sigma factor σ^V^ as a model system. σ^V^ regulates the lysozyme stress response in *B. subtilis*, activating genes related to cell wall protection and repair^41,42,44,45,61^. In a previous study, we documented the behavioural transition of the wild-type system as *S* is varied^37^. In that study, lysozyme stress input was varied, which corresponds to a modulation of *S* in our simulations. Using single-cell time-lapse microscopy, the study demonstrated a transition from no activation, to heterogeneous activation, to more homogeneous activation, as lysozyme was increased. This is similar to the transitions observed when *S* is increased in the *D ≲ 1* region of the parameter map (Fig. 3). Although our model often (but not always) includes a stable bistable regime between the no-activation and heterogeneous-activation regions, this behaviour will appear as no activity in these simple input-onset experiments (Supplementary Tables 7, 8). With this consideration, previous experimental observations align well with the model predictions.

Our model predicts that the *D ≲ 1* and *D ≳ 1* regions can be reached within the same system, and that increasing *D* (the strength of self-deactivation) can shift the system from one regime to the other. To test this, we engineered a series of modified σ^V^ circuits in which *D* was increased by adding additional copies of the anti-sigma factor (*rsiV*) (Methods, Supplementary Fig. 3). As *rsiV* expression is upregulated by σ^V^ and RsiV inhibits σ^V^ activity^46,47^, this manipulation allows us to tune the system’s self-deactivation strength. We confirmed via RT-qPCR that the additional copies of *rsiV* decreased the levels of *sigV* expression under 1 µg/ml lysozyme stress conditions in liquid culture (Methods, Supplementary Notes 11, Supplementary Fig. 37).

**Fig. 5:**
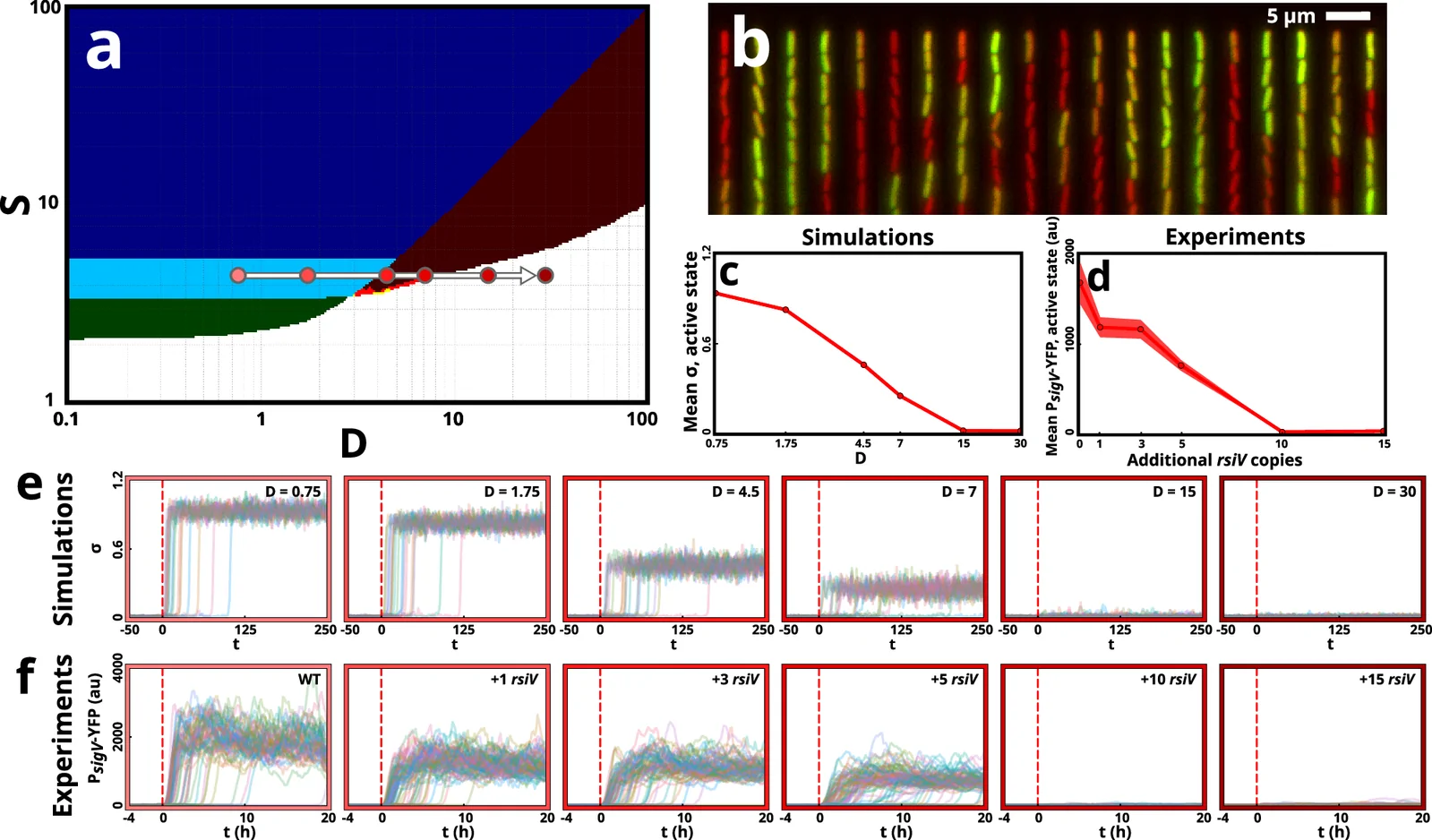
The model’s predicted transition with increasing D levels, from heterogeneous activation to no activity, is validated in the σ^V^ system. The model predicts that, as D is increased, σ^V^’s steady-state activity is reduced while its heterogeneous activation remains. For high enough *D* levels, the system is fully inactivated. **a** Six representative *D* values sampled from the model’s *S*-*D* behaviour map. **b** Tile of randomly selected mother machine channels with WT cells 90 min after induction with 1 µg/ml lysozyme. The cells contained a P_*sigV*_-YFP reporter (green colour) and a constitutively expressed P_*trpE*_-mCherry reporter (red) for segmentation. **c** Simulated ON-state mean σ levels for the six points marked in a decline as D increases. **d** For six different experimental *D* values, mean P_*sigV*_-YFP expression after 1 µg/ml lysozyme stress are shown (shaded region shows standard deviation). This confirms the decrease in ON-state activity with increasing D as predicted by the model. **e** Single-cell simulation traces (*n* = 20) for each condition in **a**. **f** Experimental single-cell fluorescence trajectories under 1 µg/ml lysozyme, recapitulating the simulated shift from heterogeneous activation to inactivity. We note that the third and fourth sets of simulations and experiments, respectively, display a combined heterogeneous activation/intermediate activation behaviour (marked as intermediate activation in **a**). A detailed description of the system’s potential for such hybrid behaviours can be found in Supplementary Notes 7.4. All experiments have *n* = 3–4 independent repeats. Source code used to generate this figure can be found in the provided GitLab repository. Source data for this figure is available in the Source Data file.

According to our model, increasing *D* should progressively reduce σ^V^ activity in the ON state while preserving heterogeneous activation; at sufficiently high *D*, all activity should be suppressed (Fig. 5a,c, e). We tested these predictions using single-cell microscopy in the mother machine microfluidic device (Fig. 5b). Six P_*sigV*_-*YFP* reporter strains, each containing different copy numbers of *rsiV* (and therefore different *D* levels), were subjected to 1 µg/ml lysozyme stress. As the copy number of *rsiV* increased, the trend of σ^V^ ON-state activity decreased as expected, while its heterogeneity remained, consistent with the model’s predictions. At the highest *D* levels, σ^V^ activity was entirely suppressed (Fig. 5d,f, Supplementary Figs. 38, 39).

Finally, we examined the σ^V^ system’s behaviour once it has transitioned into the *D ≳ 1* regime. According to our model, this regime supports two possible *S*-transitions depending on the values of the remaining parameters *τ*, *v*_*0*_, *n*, and *η*, with *τ* having the primary effect (Supplementary Notes 9). These transitions include either pulsing/oscillatory behaviours, or intermediate activation (Fig. 3, Supplementary Figs. 31–35).

**Fig. 6:**
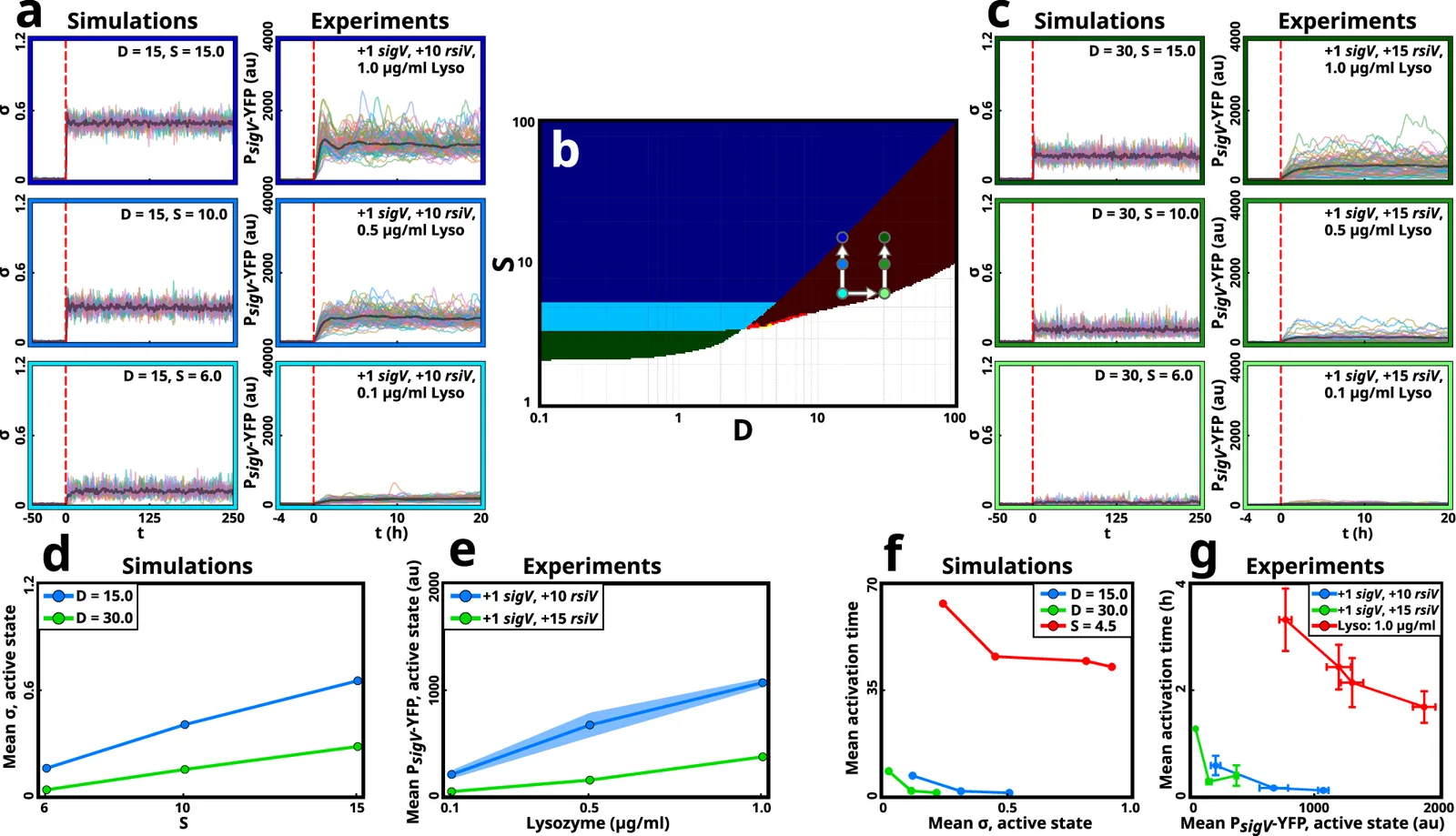
In the high-D regime for the σ^V^ system, increasing S produces the model-predicted intermediate-activation transition. Having shifted the σ^V^ system into the *D ≳ 1* regime (Fig. 5), we next varied *S* and tested which *S*-dependent transition the system exhibits. The model predicts that, for high *D* levels, σ^V^ exhibits an intermediate activation transition as *S* is varied (unlike the heterogeneous activation transition previously observed in the wild-type^37^). **a** For fixed *D = 15*, the model (left) shows an intermediate activation transition as S is increased (simulations for *S = 6*, *10*, *15*). The ON-state activity increases with *S*. Experimental single-cell traces (right) for three lysozyme concentrations (0.1, 0.5, 1 µg/ml) validate this transition in a high-*D* σ^V^ strain (which carries 10 additional *rsiV* and one additional *sigV* copy). **b** The six *S*-*D* combinations used in a and c’s location in parameter space. **c** As *D* is increased (to 30), the model (left) predicts a similar transition as in a, but with lower ON-state activity. We replicate this *D* increase by adding 5 *rsiV* copies to the strain used in a. Consistent with the model, this strain (right) replicates the transition in a, but with reduced ON-state activity. This confirms our prediction that activity levels are tuned by both *S* (positively) and *D* (negatively). Mean ON-state activity levels, as depending on *S*, for the simulations (**d**) and experiments (**e**) shown in **a** and **c**. Shaded regions show standard deviation (s.d.). In the model (**f**) and experiment (**g**), the intermediate activation transitions (blue and green lines) exhibit homogeneous activation. For comparison, the data at fixed *S* (*S = 4.5*) and increasing *D* from Fig. 5 (re-plotted here in red; fixed 1 µg/ml lysozyme in experiment) activates heterogeneously. Error bars show mean +/− s.d. All experiments have *n* = 3-4 independent repeats. Source code used to generate this figure can be found in the provided GitLab repository. Source data for this figure is available in the Source Data file.

To identify in which region of parameter space the system is in, we modulated *S* in our two high *D* strains by adding an additional copy of *sigV* driven by its own promoter and by varying lysozyme stress levels. We found that σ^V^’s transition specifically falls into the intermediate activation category (Fig. 6a–c). Here, our model predicts that the intermediate activation behaviour’s steady-state activity is smoothly tuned by both the levels of *S* and *D*. Specifically, high *S* increases σ^V^’s activity whilst high *D* reduces it (Fig. 6d). We confirmed these predictions experimentally (Fig. 6e, Supplementary Movie 1). We also note that activation times are homogeneous in both simulation and experiment, further suggesting that the system has transitioned out of the heterogeneous activation domain observed for lower *D* values (Fig. 6f–g). Together, these results confirm our prediction that, by tuning system parameters, a mixed feedback loop can transition between dynamically distinct behavioural regions.

### The predicted pulsing/oscillatory behavioural transition is validated in the σ^B^ system

Next, we experimentally tested the prediction from Fig. 3 that increasing *S* can drive the system from stochastic pulsing into sustained oscillations. To do so, we used σ^B^, the master regulator of the general stress response in *B. subtilis*^62–64^. Under persistent energy stress, σ^B^ exhibits stochastic pulsing^8,38^. Our parameter maps predict a range of behaviours that can be generated from stochastic pulsing, such as oscillations, by tuning the value of *S*.

Normally, observation of potential high-*S* behaviours (such as oscillations) by tuning energy stress levels is not possible in σ^B^, as high energy stress causes secondary effects such as cell death. To avoid this issue, we rewired the σ^B^ circuit to synthetically drive σ^B^ via its energy stress pathway, adapting an approach previously used to re-wire σ^B^ via the environmental stress pathway^38^. In a *B. subtilis* strain containing a reporter for σ^B^ activity, P_*sigB*_-*YFP*, we knocked out *rsbP* and *rsbU*, the upstream phosphatases that activate the anti-anti-sigma factor RsbV under stress (Methods). We also chromosomally integrated a mutated form of RsbP, which is constitutively active, under the control of an IPTG inducible promoter to drive σ^B^ expression (Supplementary Fig. 4). The mutated RsbP had the PAS and coiled-coil region removed causing it to be constitutively active^65^. This enabled us to experimentally vary *S* by adding IPTG to the growth medium, allowing us to investigate the system’s response across a range of values.

**Fig. 7:**
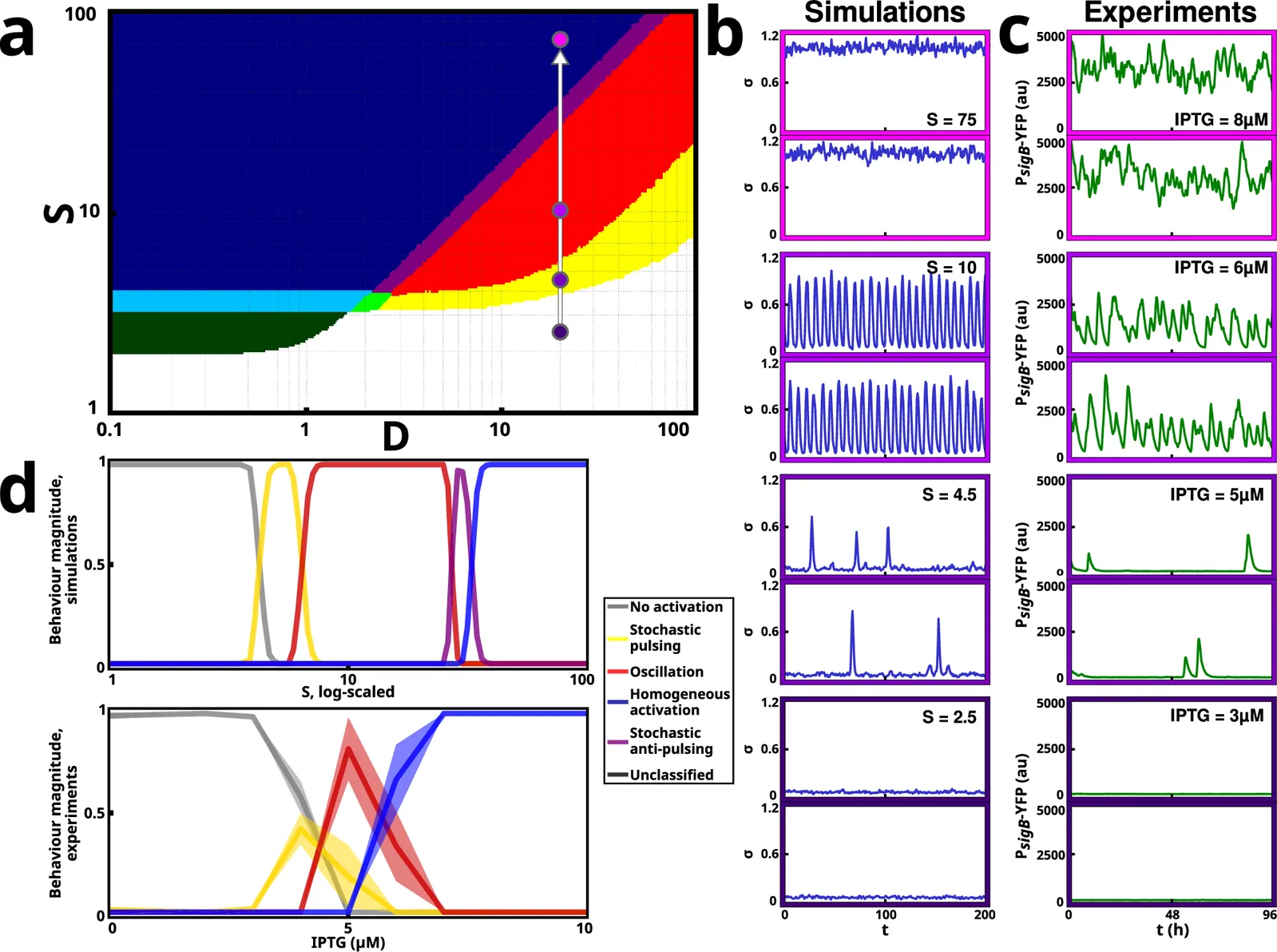
The model’s predicted transition through the pulsing/oscillatory region is validated in the σ^B^ system. **a** A behaviour map in which increasing self-activation strength (*S*) in the *D ≳ 1* region drives a transition from no activation, through stochastic pulsing and oscillations, into homogeneous activation. **b** Simulated trajectories at four different S values (purple dots in **a**) under constant input, illustrating the predicted progression through no activation, stochastic pulsing, oscillations, and homogeneous activation. **c** Representative single-cell traces of the rewired *B. subtilis* P_*sigB*_-YFP strain (Methods) at changing IPTG concentrations, showing the same behavioural sequence as in the model. **d** A neural network-based classifier applicable to both simulation and experiment was developed to assign time-trajectories to the different behavioural classes (Supplementary Notes 12). When applied to simulated (from the parameter slice in **a**) and experimental (0–10 µM IPTG) trajectories, the classifier confirms that both systems exhibit the same transition sequence. Line and shaded regions show mean +/− standard deviation over two experimental replicates held out from classifier training. Across the full dataset, 3–5 independent experimental replicates were collected per condition. We note that the experiments do not exhibit the stochastic anti-pulsing region seen in the model, potential explanations for this are discussed in the main text. Source code used to generate this figure can be found in the provided GitLab repository. Source data for this figure is available in the Source Data file.

We scanned our parameter space maps to determine which transition the system undergoes, given it starts in stochastic pulsing (the behaviour found in σ^B^), as *S* is modulated (Supplementary Notes 9). We note that stochastic pulsing is almost exclusively preceded (i.e., at lower *S*) by no activation, and followed (i.e., at higher *S*) by oscillation (Supplementary Tables 7, 8). In addition, at sufficiently high *S* the system activates homogeneously. Finally, for some systems, the oscillating and the homogeneous activation regions are separated by a region of stochastic anti-pulsing (Fig. 7a, Supplementary Tables 7, 8 and Supplementary Figs. 31–35).

We then measured the σ^B^ activity of our rewired P_*sigB*_-*YFP* strain in the mother machine microfluidic device across a range of IPTG concentrations (Methods). As IPTG levels increased from low to high, the σ^B^ system transitioned through no activation, stochastic pulsing, oscillation, and homogeneous activation behaviours, consistent with the model’s predictions (Fig. 7b, c, Supplementary Figs. 40–43, Supplementary Movie 2).

Next, we asked whether behaviours observed in simulated and experimental systems could be linked using a common trajectory-level analysis. Here, we developed a neural network-based classifier operating on single trajectories, rather than parameter sets (Supplementary Notes 12). This enables classification of both simulated and experimental trajectories, which are assigned to nine of the ten behaviours considered in this paper (the exception is stable bistability, which cannot be distinguished from no activation in single trajectories). First, we trained it on a combination of experimental σ^V^, σ^B^, and simulated trajectories, and confirmed its performance on a mixed simulated/experimental validation set (taking care not to use trajectories from the same experimental repeat in both training and validation) (Supplementary Figs. 44–49). Next, we applied it to a new experimental system (*B. subtilis* sigma factor σ^M^)^66^. Specifically, we analysed new mother-machine measurements of a P_*sigM*_-YFP reporter across a range of bacitracin concentrations^67^. Here, σ^M^ exhibits the single response pulse behaviour that was represented only in the simulated training data (which did not include any σ^M^ trajectories). Still, the classifier could correctly identify this (and other, such as stochastic pulsing) σ^M^ behaviours, demonstrating that our results can be generalised to new experimental systems (Supplementary Fig. 50).

Finally, we applied the trajectory-based classifier to the σ^B^ trajectories from Fig. 7. Here, we could quantitatively demonstrate that both the simulated and experimental systems undergo the same transition from no activation, through the stochastic pulsing and oscillation behaviours, to homogeneous activation (Fig. 7d). We note how the model’s stochastic anti-pulsing region is not reproduced experimentally. This discrepancy could reflect reporter kinetics (e.g., half-life) limiting temporal resolution to distinguish anti-pulses, or additional complexity of the σ^B^ pathway enabling a different transition than what is predicted by our minimal model. Furthermore, the stochastic anti-pulsing behaviour only occurs for some combinations of *τ*, *v*_*0*_, *n* and *η*, while for others, oscillation transitions directly to homogeneous activation (with the former being about six times more common, Supplementary Tables 7, 8, Figs. 31–35). Notably, the transition from stochastic pulsing to oscillations and then directly to homogeneous activation also matches the transition identified in a previous stochastic model of the full σ^B^ circuit^68^.

## Discussion

In this work, we present a general model of a mixed positive/negative feedback loop, which predicts ten distinct single-cell dynamical behaviours in the presence of molecular noise (Fig. 2). By employing an automated parameter set classification scheme (Supplementary Notes 7), we mapped the occurrence of these behaviours across parameter space, revealing how each behaviour depends on the system’s properties (parameters) (Fig. 3). A detailed analysis of each behaviour and its dynamics is provided in Supplementary Notes 8.

The mixed positive/negative feedback loop is a common motif in cellular pathways. Our work provides a comprehensive framework that predicts how such systems generate a wide range of behaviours. For bacterial sigma factors, a group of commonly studied systems whose circuits contain mixed positive/negative feedback loops, several predicted behaviours have already been observed using single-cell microscopy^37,39,40,69^. These include single response pulse, stochastic pulsing, and heterogeneous activation. As more sigma factor systems are explored, our model predicts that additional behaviours may be observed.

Crucially, our model also predicts how mixed feedback loops’ behaviours depend on a small number of biologically interpretable parameters (such as feedback strengths and noise levels). This includes inferring likely circuit properties from observed behaviours. For example, the σ^B^ circuit, which displays stochastic pulsing, is predicted to operate at a higher self-deactivation strength (*D*) than the σ^V^ circuit, which displays heterogeneous activation. More importantly, it predicts how tuning these parameters generates, and transitions the system between, different behaviours. By dividing parameter space into two approximate regions, *D ≲ 1* and *D ≳ 1*, we note how each undergoes a distinct behavioural transition as the input (or self-activation) parameter, *S*, is varied (Supplementary Fig. 30). For *D ≲ 1* the transition is characterised by bistable behaviours, while for *D ≳ 1* a range of pulsing and oscillatory behaviours are exhibited.

To validate our model, we examined these transitions in two real systems (the alternative sigma factors σ^V^ and σ^B^, both from *B. subtilis*). In σ^V^, we first showed that increasing *D* can move the system from the *D ≲ 1* region into the *D ≳ 1* region (Fig. 5). We then used high-*D* strains to identify the corresponding *S*-dependent transition (Fig. 6). Together, these results show that the two broad dynamical regions predicted by the model can be accessed within the same underlying circuit. In σ^B^, increasing *S* drove the predicted progression from no activation through stochastic pulsing and oscillations to homogeneous activation (Fig. 7). Next, we trained a neural network-based classifier that assigns both simulated trajectories and experimental single-cell traces to the different behavioural classes. This enabled a quantitative comparison showing that simulated and experimental trajectories in the σ^B^ system undergo the same sequence of behavioural transitions (Fig. 7d). We also showed how the classifier could correctly identify behaviours that were only present in the simulated training data (single response pulse) in a new experimental system (σ^M^). Together, these results support the idea that the model captures general principles that extend across mixed feedback loops. More broadly, the framework may also be relevant to mixed feedback circuits in other biological contexts, including ecological dynamics^70,71^ and neural signalling circuits^15^, as well as non-biological systems such as electronic control circuits^72^.

A central advantage of our approach is the use of a minimal, coarse-grained model to capture the essential dynamics of gene regulatory circuits. By not reproducing the detailed biochemistry of specific systems, our model allows us to make conclusions that can be generalised across mixed feedback loops. Furthermore, by testing these predictions in vivo, we confirm that our analysis holds when applied to real systems containing additional complexity, supporting the use of minimal models to make biologically relevant predictions. Finally, we show both how noise enables qualitatively distinct dynamic behaviours, such as stochastic pulsing and heterogeneous activation, and also how it can cause non-intuitive shifts of the boundaries between behavioural regimes in parameter space, effectively ‘warping’ the behavioural boundaries (Fig. 4, Supplementary Fig. 36).

Our insights are also relevant to synthetic biology, where mixed positive/negative feedback loops, such as those involving sigma factor circuits, have been proposed as potential regulators of synthetic systems^73–75^. Here, we have only experimentally tuned two of the six parameters (*S* and *D*). Furthermore, the large number of additional genes required to tune *D* limits the practicality of our approach for most applications. In the future, it will be important to explore whether synthetic mixed positive/negative feedback loops that allow for tuning additional properties can be developed. Here, our work provides a blueprint for predicting and engineering circuit behaviours from minimal models, offering a foundation for designing synthetic regulators with tailored dynamic responses.

## Methods

### Mathematical model

Our minimal model of a general mixed positive/negative feedback loop tracks the concentration of a single component (*σ*). Degradation/dilution was assumed to be linear. To make production as general as possible, it was modelled as a Hill function. The model is based on the following three assumptions:

- *σ* activates its own production.
- *σ* deactivates its own production.
- The deactivation is subject to a time delay.

To take these into account, production depended on both an activating term (*σ*) and a repressing term (*σ* subject to a time delay).

The time delay was implemented using the linear chain trick (LCT)^59,60^. While this introduces additional variables to the system, it has the advantage that the model can be represented as ODEs and SDEs (instead of DDEs and SDDEs). After confirming that the number of intermediaries used in the LCT had little effect on the model, we decided to implement it using three intermediaries (Supplementary Figs. 7, 8, Supplementary Notes 4.1). Finally, we also confirmed that all ten response behaviours could be recreated in a model with a discrete delay (Supplementary Fig. 9, Supplementary Notes 4.2).

Initially, our model depended on 9 parameters. Since we only sought to investigate qualitative behaviours, nondimensionalisation was performed. This section will describe the nondimensionalised model. The nondimensionalisation process and the non-nondimensionalised model are described in Supplementary Notes 2.

### Reactions

Initially, our model contains only two reactions, production and degradation (or dilution) of the sigma factor (*σ*, the model’s only species). An additional three production and degradation reactions are added by the LCT (which also adds three intermediary species, *A*_*1*_, *A*_*2*_, and *A*_*3*_). These reactions are described in Table 1.

**Table 1:** The reactions of the general sigma factor circuit model

Description	Reaction	Rate	Propensity
Production	∅ → *σ*	v_*0*_ + (*S*[σ])^*n*^/((*S*[σ])^*n*^ + (*D*[*A*_*3*_])^*n*^ + *1*)	v_*0*_ + (*S*[*σ*])^*n*^*/*((*S*[σ])^*n*^ *+ *(*D*[*A*_*3*_])^*n*^ + *1*)
Degradation/dilution	*σ → ∅*	1	[*σ*]
Production (LCT)	∅ → *A*_*1*_	[*σ*]/τ	[*σ*]/τ
	∅ → *A*_*2*_	[*A*_*1*_]/τ	[*A*_*1*_]/τ
	∅ → *A*_*3*_	[*A*_*2*_]/τ	[*A*_*2*_]/τ
Degradation/dilution (LCT)	*A*_*1*_ → ∅	1/τ	[*A*_*1*_]/τ
	*A*_*2*_ → ∅	1/τ	[*A*_*2*_]/τ
	*A*_*3*_ → ∅	1/τ	[*A*_*3*_]/τ

The model primarily consists of two reactions, production and degradation of the sigma factor (*σ*). However, since the time delay (of the negative feedback of *σ* on its own production) is implemented using the LCT, an additional 3 species (*A*_*1*_, *A*_*2*_, and *A*_*3*_) are introduced. This also adds an additional three production and three degradation reactions

### Parameters

Our nondimensionalised model consists of 6 parameters, as described in Table 2. The model does not have specific parameter values. Instead, every instance of the model, with specific values of *(S*,*D*,*τ*,*v*_*0*_,*n*,*η)* represents a potential mixed positive/negative feedback loop. E.g. by letting *S* be large and *D* small, we can investigate the behaviour of circuits with high degrees of self-activation and low degrees of self-deactivation. As we scan these parameters’ values, we can investigate how systems’ properties affect their behaviours. These scans are made possible through the nondimensionalisation process, which reduces the dimension of parameter space.

**Table 2:** The parameters of the (nondimensionalised) general sigma factor model

Parameter	Description
*S*	Degree of self-activation (also includes input strength).
*D*	Degree of self-deactivation.
*τ*	Length of the self-deactivation time delay.
*v*_*0*_	Operon base activity/leakage.
*n*	Hill coefficient (degree of non-linearity in the circuit).
*η*	Degree of noise in the circuit.

We note that the parameter *S* is a composite of the pre-nondimensionalised parameters $\hat{S}$ and $\hat{I}$, where $\hat{S}$ is the degree of system self-activation, and $\hat{I}$ the magnitude of the input signal (Supplementary Notes 2). Hence, modulation of a system’s input corresponds to modulation of the parameter *S*.

### Model simulations

We wish to investigate the model’s behaviour in response to an input. In the absence of the input (in response to which we wish to investigate the response), *S* = *0*. When we simulate our model for a specific parameter set *(S*,*D*,*τ*,*v*_*0*_,*n*,*η)*, we will initialise it with *S* = *0*, with the parameter *S* only attaining its designated value at the time of input onset (always at time *t* = *0*). E.g. a model instance may be defined by the parameter set *(S*,*D*,*τ*,*v*_*0*_,*n*,*η)=(5.0,5.0,10.0,0.1,2,0.1)*, and simulated throughout the time interval *(-500,2000)*. The simulations start with the parameter values *(0.0,5.0,10.0,0.1,2,0.1)*, which are changed to *(5.0,5.0,10.0,0.1,2,0.1)* at time *0*, at which point the system activates.

It would be possible to vary the value of *S* throughout a simulation, to model inputs with varying magnitude. While, for simplicity, this work will focus on systems with a step increase in the input, we do provide additional analysis for non-step inputs (Supplementary Notes 13, Supplementary Fig. 51).

Practically, the model was implemented in the Julia programming language using the Catalyst.jl modelling package^76^. All simulations were carried out using the DifferentialEquations.jl package^77^. The ImplicitEM solver was used as it handles stiff SDE systems well, and allows for adaptive time stepping^78^. Stochastic chemical kinetics were simulated using Gillespie’s direct algorithm using the JumpProcesses.jl package^79^.

### Automated parameter set classification

Initial steady state and stability analysis of our model determined that it may have either 0, 1, or 2 stable steady states (Supplementary Notes 6). While a dynamic system asymptotically approaches a stable steady state, perturbations may cause a transition towards another steady state. Such perturbations may either be transient (change in input magnitude) or asymptotic (noise). Based on the possible transitions between the system’s potential steady states, we designed a classification scheme for the system’s behaviour (Supplementary Notes 7). This scheme also takes into account the possibility of excitable and oscillatory behaviours. A total of 10 system behaviours (as described in the Results) were predicted. Further behaviours could be created by subdividing the initial ten, however, we deemed such subdivisions non-beneficial (Supplementary Notes 7.4). Further description and analysis of the individual behaviours can be found in Supplementary Notes 8.

To enable our mapping of behaviour across parameter space, we created a decision tree-based heuristic classifier algorithm. When provided with a parameter set *(S*,*D*,*τ*,*v*_*0*_,*n*,*η)*, the algorithm simulates the model multiple times, and assigns it one of the behavioural classes described in Figure 2. Primarily, decision tree decisions are based on transition times between potential steady states (accounting for the fact that stochastic systems do not have steady states in the strict sense, and that e.g., oscillatory systems have no stable steady states at all). Additional considerations, such as analysis of trajectory periodograms to detect oscillatory behaviours, are also made. The full algorithm is described in Supplementary Notes 9. In addition, to further prove its validity, we randomly generated 10 parameter sets from each behaviour (as classified by the algorithm). By showing that simulations of these parameter sets align with their designated behaviours, we demonstrate the efficacy of the algorithm (Supplementary Figs. 18, 19).

Finally, we note that an additional, trajectory-based classifier was developed. This was specifically developed to handle comparisons between simulated and experimental trajectories (as in the latter case, we lacked the ability to simulate an arbitrary number of trajectories for each condition). This classifier was based on an ensemble of neural networks and is described in Supplementary Notes 12.

### Bifurcation analysis

Bifurcation diagrams were generated using the BifurcationKit.jl software^80^.

### Experiments

#### Strains and media

All strains are derived from *B. subtilis* 168 or PY79. Deletions were made by recombination of linear DNA fragments homologous to the region of interest and replacing the gene of interest with an antibiotic resistance cassette. All strains had a housekeeping σ^A^ promoter-driven mCherry as a constitutive control and for segmentation.

Cells were grown in Spizizen’s Minimal Media (SMM)^81^. It contained 50 µg/ml tryptophan as an amino acid source and 0.5% glucose as a carbon source. Cultures were started from frozen stock in SMM and grown overnight at 30 °C to an OD between 0.3 and 0.8. The overnight cultures were resuspended to an OD of 0.01 and regrown to an OD of 0.1 at 37 °C.

#### Plasmid and construct descriptions

E. coli strain DH5α was used for plasmid cloning. Cloning was performed using standard molecular cloning techniques, including restriction enzyme digestion followed by T4 DNA ligation, Gibson assembly, and non-ligase-dependent cloning where appropriate. Plasmids were chromosomally integrated into the 168 or PY79 background via double crossover using standard techniques. The list below describes plasmids and genetic constructs present in the strains used in this study, including previously described or gifted reporter constructs where relevant. Details of selection marker and integration position/cassette are given at the beginning of each entry. Note that all plasmids below replicate in *E. coli* but not in *B. subtilis*. Primers used for cloning constructs generated in this study are listed in Supplementary Data 1.

1. **ppsB::P**_***trpE***_ **-** ***mCherry*** **ErmR** This construct provides uniform expression of mCherry from a σ^A^-dependent promoter, enabling automatic image segmentation (cell identification) in time-lapse movie analysis. A minimal σ^A^ promoter from the *trpE* gene was cloned into a vector with *ppsB* homology regions^38^. The original integration vector was a gift from A. Eldar^82^.
2. **sacA::P**_***sigB***_**-*YFP*** **CmR** This construct contains the target promoter of *sigB* cloned into the EcoRI/BamHI sites of AEC127^82^ yielding a Venus (YFP) reporter for σ^B^ activity^38^ (gift from M. Elowitz, Caltech).
3. **amyE::P**_***hyperspank***_**-*rsbP*Δ4** (**ΔPAS-CC**) The coding region of a *rsbP* mutant lacking PAS and the coiled-coil region along with a 5′ transcriptional terminator, was cloned downstream of the hyperspank IPTG-inducible promoter in plasmid pDR-111 (gift from D. Rudner, Harvard). *rsbP*Δ4 (ΔPAS-CC) is described in ref. ^65^. The strain carrying this construct, JJB441, was generated previously in the laboratory of M. Elowitz, Caltech.
4. ***sacA*::P**_***sigV***_***-YFP*** **CmR** This construct contains the target promoter of *sigV* cloned into the EcoRI/BamHI sites of AEC127^82^ yielding a Venus (YFP) reporter for σ^V^ activity^37^ (gift from M. Elowitz, Caltech).
5. ***sacA*::P**_***sigM***_***-YFP*** **CmR** This construct contains the target promoter of *sigM* cloned into the EcoRI/BamHI sites of AEC127^82^ yielding a Venus (YFP) reporter for σ^M^ activity^8^ (gift from M. Elowitz, Caltech).
6. ***amyE*::? x** (**P**_***sigV***_ **-*rsiV***) **SpecR** The target promoter of *sigV* with multiple copies of *rsiV* (?= 1x, 3x, 5x) were cloned into the sites BamHI/HindIII of pdL30. The pDL30 backbone is an *amyE* integration vector previously used for *B. subtilis* reporter construction^38^.
7. ***lacA*:: 5 x** (**P**_***sigV***_ **-*rsiV***) **TetR** The target promoter of *sigV* with five copies of *rsiV* was cloned into the sites EcoRI/NdeI of a variation of ECE174. The ECE174-derived backbone was obtained from M. Elowitz, Caltech.
8. ***pyrD*:: 5 x** (**P**_***sigV***_ **-*rsiV***) **NatR** The target promoter of *sigV* with five copies of *rsiV* was cloned into the sites EcoRI/BamHI of a variation of ECE174. The ECE174-derived backbone was obtained from M. Elowitz, Caltech.
9. ***hag*::P**_***sigV***_**-*sigV*** **ErmR** The target promoter of *sigV* driving *sigV* and 400 base pairs upstream and downstream homology regions of hag was cloned into EcoRV of pBS II SK.

#### Strain list

A list of all strains used in this article can be found in Table 3. Newly generated strains are available from the corresponding author, James C. W. Locke, upon reasonable request.

**Table 3:** List of strains used in this article

Strain Name	Genotype	Source
PY79	BGSC 1A747	BGSC
PB2	168 (trpC2)	BGSC
JP1/JLB54	PY79 *ppsB*::P_*trpE*_ - *mCherry* PhleoR	^8^
JP2	JP1; *ytvA*::neoR	^8^
JLB047	JP2; *sacA*::P_*sigV*_-YFP CmR	^37^
JLB130	JLB047; *hag*::ErmR	^37^
JLB154	JLB130; sigV::TetR	^37^
JLB212	JLB130; *amyE*::P_*sigV*_-*rsiV* SpecR	^37^
JLB288	JLB130; *amyE*::3x P_*sigV*_-*rsiV* SpecR	This work
JLB293	JLB130; *amyE*::5x P_*sigV*_-*rsiV* SpecR	This work
JLB309	JP2; *sacA*::P_*sigM*_-YFP CmR *hag*::ErmR	This work
JLB325	JLB293; *lacA*::5x P_*sigV*_-*rsiV* TetR	This work
JLB324	JLB325; *pyrD*::5x P_*sigV*_-*rsiV* NatR	This work
JLB327	JLB047; *lacA*::5x P_*sigV*_-*rsiV* TetR *amyE*::5x P_*sigV*_-*rsiV* SpecR *hag*:: P_*sigV*_-*sigV* ErmR	This work
JLB345	JLB047; *amyE*::5x P_*sigV*_-*rsiV* SpecR *lacA*::5x P_*sigV*_-*rsiV* TetR *pyrD*::5x P_*sigV*_-*rsiV* NatR *hag*::P_*sigV*_-*sigV* ErmR	This work
JJB176	PB2; *ppsB*::P_*trpE*_-*mCherry* PhleoR	^38^
JJB213	JJB176; *sacA*::P_*sigB*_-YFP CmR	^38^
JJB240	JJB213; *rsbU*::KanR	^38^
JJB441	JJB240; *amyE*::P_*hyperspank*_-*rsbP*Δ4 (ΔPAS-CC) SpecR	Generated previously in laboratory of M. Elowitz
JLB264	JJB441; *rsbQP*::TetR	This work
JLB346	JLB264; *hag*::ErmR	This work

#### Microscopy

An inverted Nikon Ti-E microscope (Nikon, Netherlands) with a Photometrics Prime sCMOS camera (Photometrics, USA) and a 100x phase Plan Apo (NA 1.4) objective (Nikon, Netherlands) were used for all experiments. Brightfield illumination was provided by a LED lamp (CoolLED, UK) and for epifluorescence illumination a Lumencore Solar II light engine (Lumencore USA) was used. Chroma filters (Chroma, Bellows Falls, USA) #41027 for the RFP channel and #49003 for the YFP channel were used in combination with the epifluorescence. For temperature control a stage incubator (Solent Scientific, UK) was set at 37 °C for all experiments. MetaMorph (Molecular Devices, Sunnyvale, CA, USA) controlled the camera, the motorised stage (Nikon, Amsterdam, Netherlands) and the microscope.

#### Microfluidic designs

Two microfluidic designs were used in this study. All experiments on σ^B^ and σ^M^ were done in a traditional mother machine device^36^. The master for these chips was fabricated by exploiting a Kloe Dilase 650, direct laser writer (Kloe, France). More details on the fabrication can be found in the Supplementary Notes 14. All experiments on σ^V^ were done in a mother machine device with side trenches^83^. This epoxy master was a kind gift from the Elowitz lab.

#### Microfluidic chip fabrication

All microfluidics chips were made using Sylgard 184 polydimethylsiloxane (PDMS) (Dow Corning, USA) using a 10:1 base to curing agent ratio. The PDMS was poured onto the silicon master and cured at 65 °C for 2 h. Chips were cut out and inlets were punched into the PDMS using a 1 mm biopsy puncher (WPI, UK). The chips were then plasma bonded to glass bottom dishes (Wilco Wells, Netherlands) using a Femto Plasma System (Diener, Germany) (12 s at 30 W and 0.35 mbar). To strengthen the bonding of the PDMS to the glass dish the chips were put into the oven for 10 min at 65 °C.

#### Microfluidic experiments

10 ml of the regrown overnight culture with an OD of 0.1 were spun down at 4000 rpm (3000 g) for 10 min and resuspended in 100 μl. This cell suspension was loaded into the feeding channels and spun for 5 min at 3000 rpm using a spin coater (Polos SPIN150i, SPS) to improve the loading of the cells into the growth channels.

The loaded chips were then mounted onto the microscope stage and connected to a syringe pump (KD Scientific, USA) using tygon tubing (Cole Parmer, USA). A constant flow rate of 0.15 ml/h and a temperature of 37 °C was maintained throughout the experiment. In all experiments with P_*sigV*_-YFP or P_*sigM*_-YFP cells were first grown in SMM overnight for ~100 frames (with 1 frame every 10 mins) before the media was switched to contain various levels of lysozyme (0.1, 0.5 or 1 μg/ml) or bacitracin (10 or 25 μg/ml). Switching was provided by disconnecting the tubing from a syringe with no stress (SMM only) and connecting the tubing to a new syringe with stress (lysozyme in the case of all experiments with σ^V^ and bacitracin for all experiments with σ^M^). Subsequently, the tubing was flushed for 10 min at 10 ml/h. In all experiments with P_*sigB*_-YFP, different levels of IPTG (0, 2, 3, 4, 5, 6, 7, 8, 9, 10 μM) were present from the beginning of the experiment, and the media was not switched.

All movies were analysed using a modified version of the Schnitzcells package^84^ for MATLAB (Mathworks, USA) optimised for mother machine experiments.

#### RT-qPCR experiment

*B. subtilis* cultures were grown from frozen stock as described in the Strains and Media section. Each culture was divided into two 10 ml aliquots: one untreated control and one supplemented with 1 µg/ml lysozyme. The aliquots were incubated at 37 °C for 30 min. After incubation, two 2 ml samples were collected from each aliquot into RNase-free microcentrifuge tubes and centrifuged at 20,000 g for 5 min. The supernatant was discarded, and previously autoclaved 0.5 mm glass beads were added to each pellet. The samples were then flash-frozen in liquid nitrogen and stored at −80 °C until further use.

RT-qPCR was carried out for all *B. subtilis* cultures sampled. Total RNA was isolated using the Monarch Spin RNA Isolation Kit (New England Biolabs) according to the manufacturer’s instructions. cDNA was synthesised from equal amounts of RNA using the Transcriptor First Strand cDNA Synthesis Kit (Roche). Each bacterial culture was divided into control and lysozyme-treated samples, and two biological replicates were processed per condition. qPCR was carried out for each cDNA replicate. Primers for reference genes (*16S, rpoC*, *gyrA* and *alaS*) and target genes (*sigV*) (Supplementary Table 9) were designed using Benchling primer design software for SYBR Green-based qPCR. Reactions were prepared using LightCycler 480 SYBR Green I Master Mix (Roche) and run on the LightCycler 96 Real-Time PCR System (Roche). PCRs were performed under the following conditions: 95 °C for 2 min, 40 cycles of 95 °C for 5 s and 60 °C for 30 s, and a final melt curve stage from 60 °C to 95 °C. Control reactions included no-template controls and reverse transcription controls lacking RNA or reverse transcriptase. Gene expression was normalised by subtracting the Cq value of the reference gene from the Cq value of the gene of interest. Average expression ratios were calculated using the 2^–ΔΔCq method, where ΔΔCq represents ΔCq (gene of interest in treated sample) minus ΔCq (gene of interest in untreated control), following the protocol of Czechowski et al.^85^. The reference gene *rpoC* was selected based on stable expression across growth conditions. Independent biological experiments were performed twice, and technical reproducibility was confirmed by repeated qPCR analyses of identical cDNA samples.

#### qPCR primer sequences

A list of all qPCR primers used in this article can be found in Supplementary Table 9.

### Statistics and reproducibility

No statistical method was used to predetermine sample size. For mother machine experiments, experimental conditions were independently replicated 3–5 times. Individual experiments contained 19–100 continuously tracked single-cell channels per condition, which was sufficient to identify and classify the dynamic behaviours analysed in this study. RT-qPCR experiments were performed in two biologically independent repeats.

Channels in which no surviving cell was present at the end of the movie were excluded from further analysis, because complete trajectories were required for behavioural classification. No other exclusion criteria were applied.

The experiments were not randomised. Experimental conditions were predefined and independently replicated. The investigators were not blinded to allocation during experiments and outcome assessment.

Statistical analyses and trajectory classification were performed in Julia using built-in functions and custom scripts. Mathematical simulations were performed in Julia using the Catalyst.jl package.

### Reporting summary

Further information on research design is available in the Nature Portfolio Reporting Summary linked to this article.

## Supplementary information

Supplementary Information

Description of Additional Supplementary Files

Supplementary Movie 1

Supplementary Movie 2

Supplementary Data 1

Reporting Summary

Transparent Peer Review file

## Source data

Source Data

## Data Availability

All data used in this study are available at: https://gitlab.developers.cam.ac.uk/slcu/teamjl/loman_schwall_etal_2026^86^. Repository README files describe the repository structure. The source data supporting the figures which are not generated from source code are available in the Source Data file. Source data are provided with this paper.

## Code availability

All code for carrying out all analysis and the generation of all figures, can be found here: https://gitlab.developers.cam.ac.uk/slcu/teamjl/loman_schwall_etal_2026^86^. Repository README files and code comments direct users on its use. Julia’s default package manager can be used to reproduce and install the exact package environment used. The repository contains the 6 d behaviour maps, which is available for download and further investigation (these can also be re-generated using the repository code). Furthermore, our neural network-based classifier of dynamic trajectories is available as a Julia package at: https://gitlab.developers.cam.ac.uk/slcu/teamjl/dynamictrajectoriesclassification (including documentation and installation instructions).
