# Supplementary material for: Mixed positive and negative feedback loops drive diverse single-cell gene expression dynamics
Source: Nat Commun. 2026 Aug 17;17:9855. doi: 10.1038/s41467-026-75107-4 (PMC13578229; doi:10.1038/s41467-026-75107-4)
Supplement: Supplementary file 1 [file 41467_2026_75107_MOESM1_ESM.pdf]

Mixed positive and negative feedback loops drive diverse single-cell gene expression dynamics - Supplementary Information

Torkel E. Loman*, Christian P. Schwall*, Teresa Saez, Yujia Liu, James C. W. Locke

This is the supplementary information for the "Mixed positive and negative feedback loops drive diverse single-cell gene expression dynamics" article. It is provided as 15 different "Supplementary Note" sections (each containing subsections). Supplementary figures and tables are embedded within the supplementary notes. All data used in this paper, as well as code for carrying out all analysis and the generation of all figures, can be found here: `https://gitlab.developers.cam.ac.uk/slcu/teamjl/loman_schwall_etal_2026`.

Contents

1 Sigma factor circuits **3**

2 Model formulation and nondimensionalisation **7**

3 Replication of results using Gillespie's algorithm **11**

4 Modelling the time delay **13**
4.1 Determining the number of intermediaries in the linear chain trick . . . 13
4.2 Replication of results using a discrete delay . . . 13

5 Alternative model formulations **17**
5.1 Model with alternative mixed activating/repressive Hill connection . . . 17
5.2 Model with alternative implementation of the negative feedback . . . 18

6 Steady state analysis **20**
6.1 Steady state computations . . . 20
6.2 Nullcline analysis . . . 20
6.3 Bifurcation analysis . . . 21

7 Behaviour classification **24**
7.1 Determining the model's possible response behaviours . . . 24
7.2 Defining active and inactive states in the model . . . 26
7.3 Automated classification of the model's response behaviour . . . 27
7.4 List of behavioural subclasses . . . 32

8 Behaviour descriptions **34**
8.1 No activation . . . 34
8.2 Stochastic pulsing . . . 34
8.3 Oscillations . . . 35
8.4 Stochastic anti-pulsing . . . 36
8.5 Homogeneous activation . . . 37
8.6 Heterogeneous activation . . . 37
8.7 Stochastic switching . . . 39
8.8 Stable bistability . . . 39
8.9 Single response pulse . . . 40

8.10 Intermediate activation . . . 41
8.11 Behaviour occurrence analysis . . . 43

9 Behaviour maps **44**
9.1 Generating behaviour maps . . . 44
9.2 Bifurcation diagrams across the behaviour map . . . 44
9.3 Additional behaviour maps . . . 44

10 Behaviour transition analysis **52**
10.1 Counting the transitions between different behaviour combinations . . . 52
10.2 Noise warps parameter space . . . 52

11 Additional experimental data **56**
11.1 RT-qPCR validation of σ^V behaviours . . . 56
11.2 Additional σ^V data . . . 57
11.3 Additional σ^B data . . . 60

12 Generalised classifier **65**
12.1 Generalised classifier description . . . 65
12.2 Generalised classifier training data . . . 66
12.3 Generalised classifier validation . . . 66

13 Non-instantaneous S activation **74**

14 Experimental set-up **76**
14.1 Wafer fabrication . . . 76

15 qPCR Primer Sequences **78**

Supplementary Note 1: Sigma factor circuits

In this article, we validate our model using the σ^V and σ^B systems (which control the lysozyme and general stress response of *Bacillus subtilis*, respectively). Circuit diagrams for these can be found in Supplementary Figures 1 and 2 (for σ^V and σ^B, respectively). Additionally, Supplementary Figures 3 and 4 describe the rewired circuits used for the validation experiments (for σ^V and σ^B, respectively).

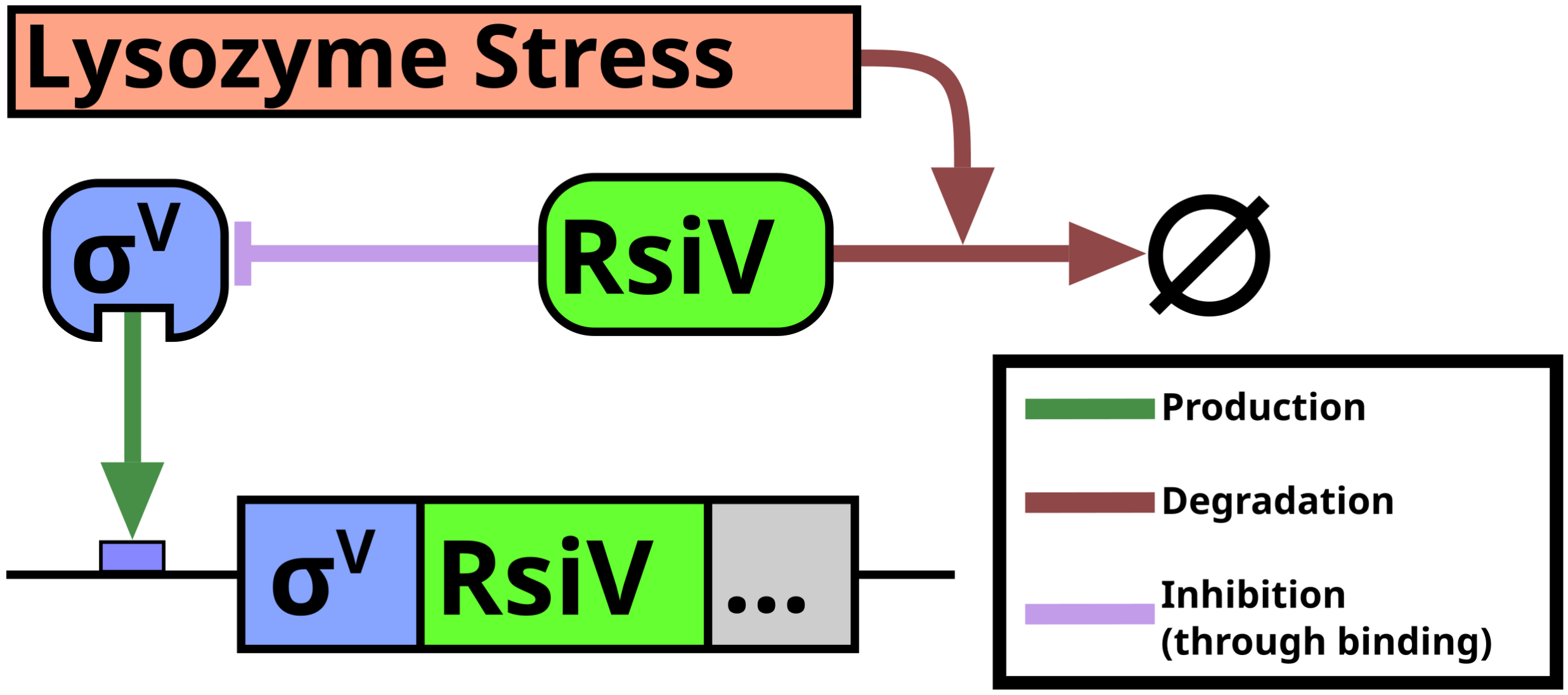

Supplementary Figure 1: **Simplified schematic of the σ^V regulatory circuit highlighting the core mixed feedback motif.** Under non-stress conditions, σ^V is bound to, and kept inactive by, its anti-sigma factor RsiV. Lysozyme stress will activate the degradation of RsiV, in the process of which σ^V is released. Free σ^V activates the genes required for the lysozyme stress response. It also activates the production of itself (σ^V), and its anti-sigma factor (RsiV), creating a mixed positive/negative feedback loop. Additional components involved in σ^V activation are omitted here for clarity.

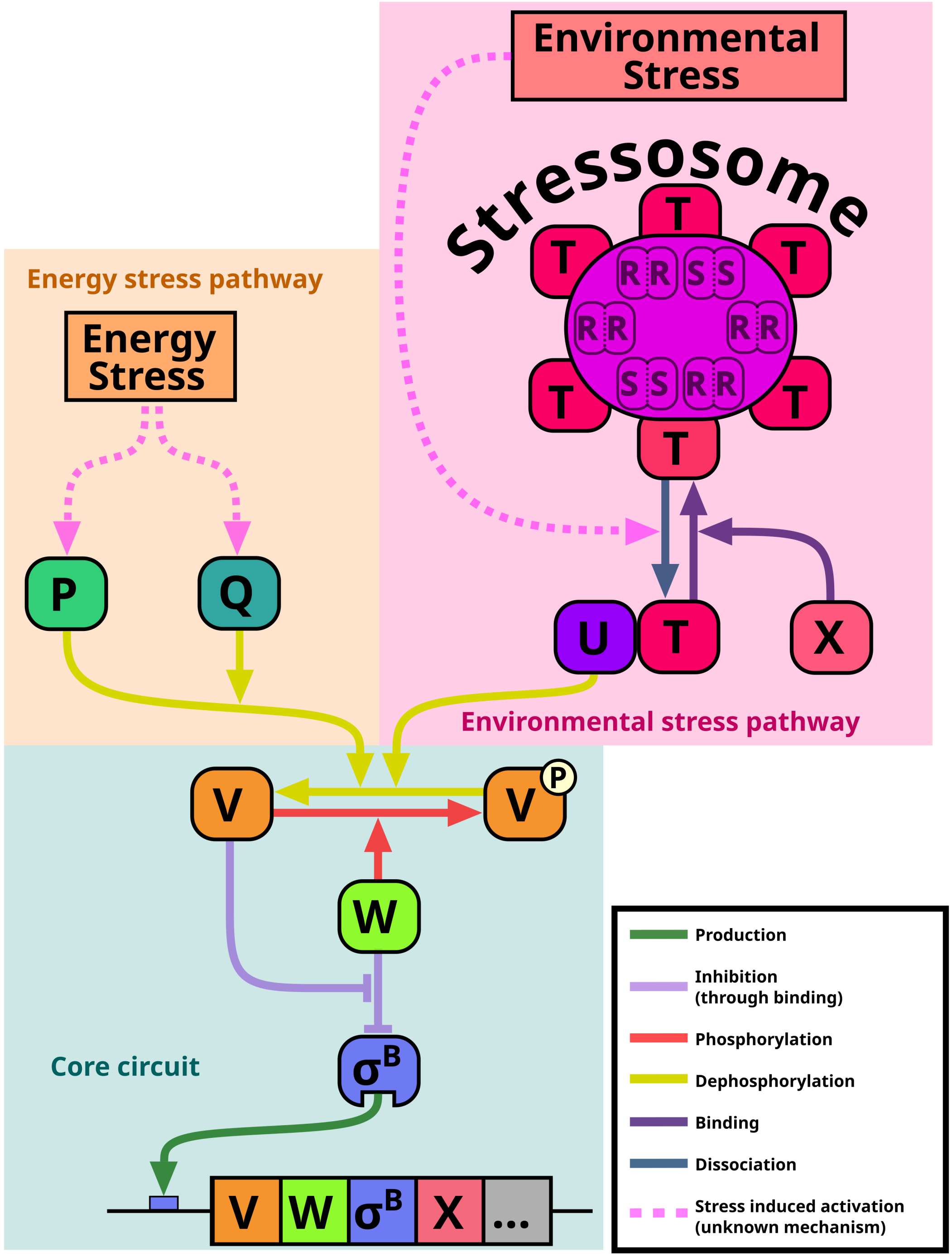

Supplementary Figure 2: **The σ^B regulatory network consists of a core circuit, which is activated by two distinct upstream pathways.** Under non-stress conditions, σ^B is bound, and held inactive by, its anti-sigma factor (RsbW, W in the figure). An anti-anti-sigma factor (RsbV, V in the figure) is inactivated by phosphorylation. The upstream pathways are triggered by two distinct types of stress (environmental and energy stress), each activating their respective phosphatase (RsbTU and RsbP, respectively). These then dephosphorylate, and thus activate, RsbV. Once activated, RsbV binds RsbW, which simultaneously releases σ^B in a partner switching mechanism. This permits σ^B to activate the general stress response of *B. subtilis*. In addition, σ^B activates the production of itself, RsbW, and RsbV, creating a mixed positive/negative(/positive) feedback loop. The environmental stress response phosphatase, RsbTU, consists of RsbU and RsbT (U and T, respectively, in the figure). Under non-stress conditions, RsbT is bound to the stressosome protein complex. The stressosome also consists of RsbS and RsbR (S and R, respectively, in the figure) dimers. It senses environmental stress (via an unknown mechanism), in response to which RsbT is released. Free RsbT can bind RsbU and activate the core σ^B circuit. In addition, RsbX (X in the figure), which is produced by active σ^B, facilitates the rebinding of RsbT to the stressosome. The details of the energy stress response are less well understood. The phosphatase RsbP (P in the figure), together with its associated factor RsbQ (Q in the figure), mediates σ^B activation in response to energy stress. In this work, we only use a rewired version of the σ^B circuit (where the WT energy and environmental stress sensing pathways have been disabled, Supplementary Figure 4).

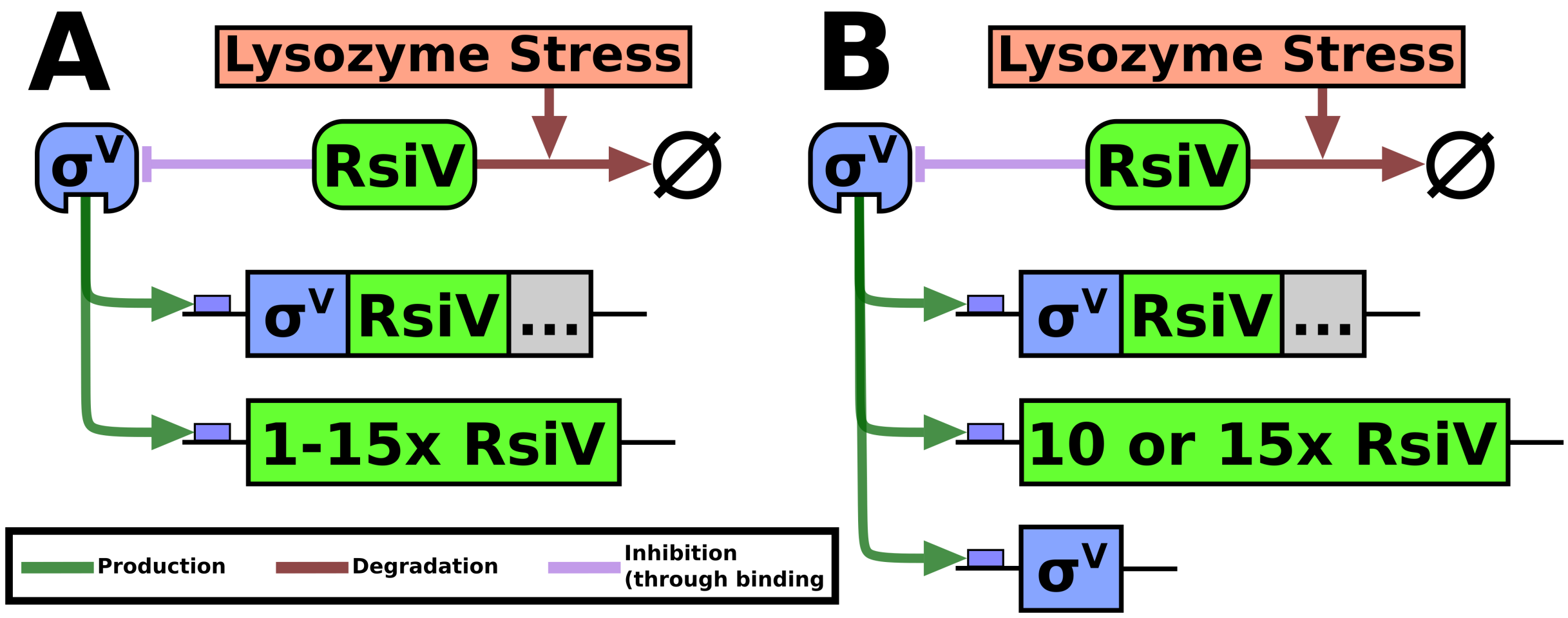

Supplementary Figure 3: **By rewiring the σ^V circuit, in addition to S, we can control D levels.** (A) We add an additional P_{sigV}-driven operon containing either 1, 3, 5, 10, or 15 copies of *rsiV*. By modulating the number of copies of *rsiV* we can control the circuit's self-deactivation strength, D. (B) To the strains in A with either 10 or 15 copies of *rsiV*, we add a single P_{sigV}-driven operon containing one copy of *sigV* only. This increases the circuit's self-activation strength, S. This enables us to reach high S levels with less severe stress strengths (reducing secondary effects on the bacteria).

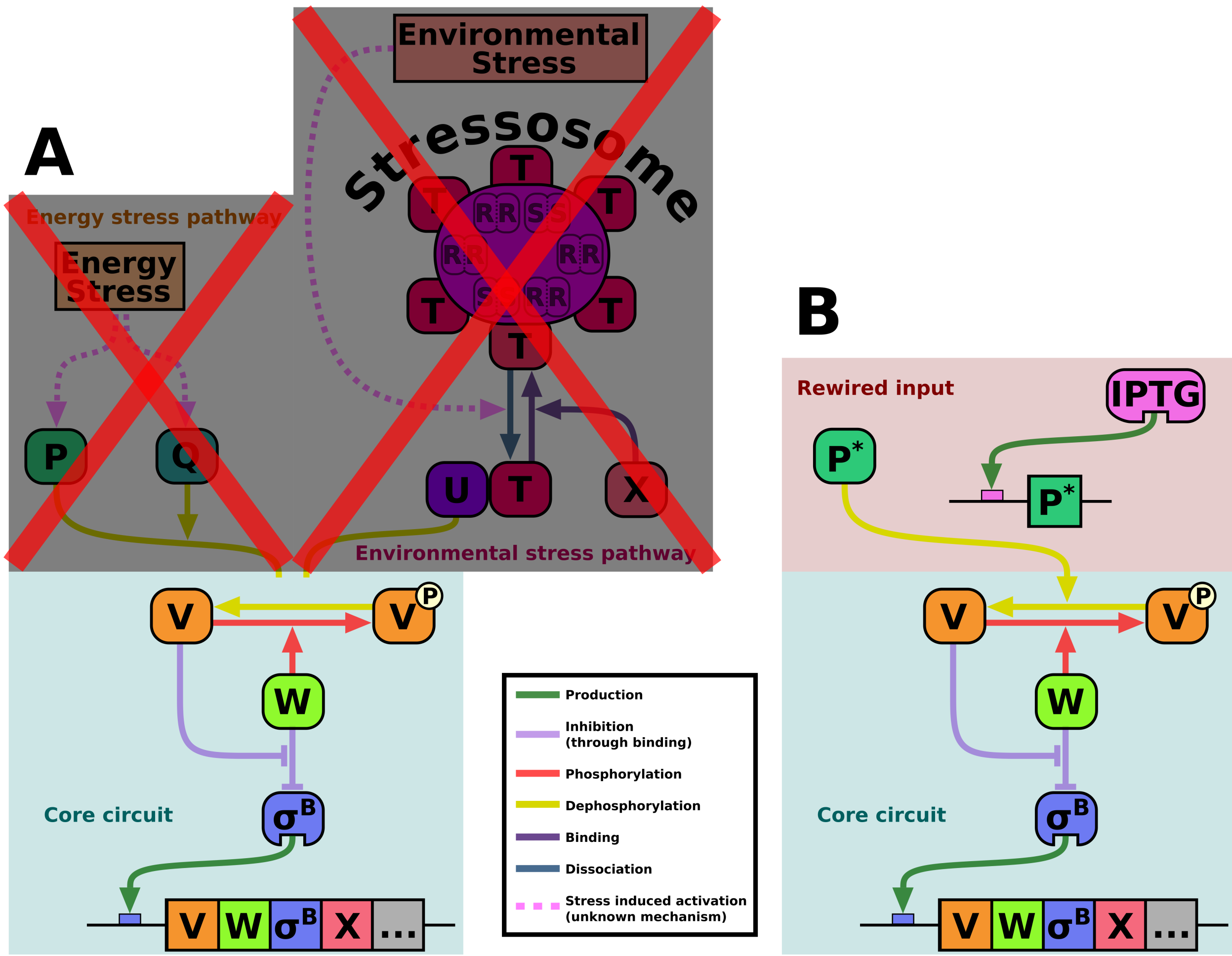

Supplementary Figure 4: **By rewiring the σ^B circuit we can control S levels.** (A) First, we delete the native energy and environmental stress response upstream pathways to σ^B. This prevents any wild-type activation of the circuit. (B) Next, we add a constitutively active RsbP (P^*) to the circuit. The production of P^* is controlled by IPTG. This way, we can control the circuit input (S) by modulating the IPTG levels.

Supplementary Note 2: Model formulation and nondimensionalisation

Our model is a chemical reaction network (CRN) type model. It initially consisted of a single species (σ), which has a corresponding production reaction (modelled as a Hill function) and a (linear) degradation/dilution reaction. Furthermore, by implementing the time delay using the linear chain trick (LCT) (Supplementary Note 4.1) we introduce additional species and reactions. We chose to use three intermediaries in the LCT (Supplementary Note 4.1, which generates an additional three production and degradation reactions (all linear). All the reactions of the initial model are described in Supplementary Table 1.

Supplementary Table 1: **The reactions of the (non-nondimensionalised) general sigma factor circuit model.** Here, ˆ is used to denote variables and parameters before nondimensionalisation (it is, however, not used to modify the species names in the reactions). The reactions' propensities are formed by multiplying their rates with their substrates.

Description	Reaction	Rate	Propensity
Production	$\varnothing \longrightarrow \sigma$	$\hat{v}_0 + \hat{v}\frac{(\hat{I}\cdot\hat{S}[\hat{\sigma}])^{\hat{n}}}{(\hat{I}\cdot\hat{S}[\hat{\sigma}])^{\hat{n}}+(\hat{D}[\hat{A}_3])^{\hat{n}}+\hat{K}^{\hat{n}}}$	$\hat{v}_0 + \hat{v}\frac{(\hat{I}\cdot\hat{S}[\hat{\sigma}])^{\hat{n}}}{(\hat{I}\cdot\hat{S}[\hat{\sigma}])^{\hat{n}}+(\hat{D}[\hat{A}_3])^{\hat{n}}+\hat{K}^{\hat{n}}}$
Degradation/dilution	$\sigma \longrightarrow \varnothing$	$\hat{k}_{deg}$	$\hat{k}_{deg}\cdot[\hat{\sigma}]$
Production (LCT)	$\varnothing \longrightarrow A_1$	$\frac{[\hat{\sigma}]}{\hat{\tau}}$	$\frac{[\hat{\sigma}]}{\hat{\tau}}$
	$\varnothing \longrightarrow A_2$	$\frac{[\hat{A}_1]}{\hat{\tau}}$	$\frac{[\hat{A}_1]}{\hat{\tau}}$
	$\varnothing \longrightarrow A_3$	$\frac{[\hat{A}_2]}{\hat{\tau}}$	$\frac{[\hat{A}_2]}{\hat{\tau}}$
Degradation/dilution (LCT)	$A_1 \longrightarrow \varnothing$	$\frac{1}{\hat{\tau}}$	$\frac{[\hat{A}_1]}{\hat{\tau}}$
	$A_2 \longrightarrow \varnothing$	$\frac{1}{\hat{\tau}}$	$\frac{[\hat{A}_2]}{\hat{\tau}}$
	$A_3 \longrightarrow \varnothing$	$\frac{1}{\hat{\tau}}$	$\frac{[\hat{A}_3]}{\hat{\tau}}$

Before nondimensionalisation, the model consists of nine parameters. These are described in Supplementary Table 2.

Through the chemical Langevin equations (CLE) [1], the reactions in Supplementary Table 1 generate the following system of stochastic differential equations (SDEs):

$$\begin{cases} \mathrm{d}[\hat{\sigma}] & = \mathrm{d}\hat{t}\cdot(\hat{v}_0 + \hat{v}\frac{(\hat{I}\cdot\hat{S}[\hat{\sigma}])^{\hat{n}}}{(\hat{I}\cdot\hat{S}[\hat{\sigma}])^{\hat{n}}+(\hat{D}[\hat{A}_3])^{\hat{n}}+\hat{K}^{\hat{n}}} - \hat{k}_{deg}[\hat{\sigma}]) \\ & +\sqrt{\hat{v}_0 + \hat{v}\frac{(\hat{I}\cdot\hat{S}[\hat{\sigma}])^{\hat{n}}}{(\hat{I}\cdot\hat{S}[\hat{\sigma}])^{\hat{n}}+(\hat{D}[\hat{A}_3])^{\hat{n}}+\hat{K}^{\hat{n}}}}\cdot\mathrm{d}\hat{W}_1 - \sqrt{\hat{k}_{deg}[\hat{\sigma}]}\cdot\mathrm{d}\hat{W}_2 \\ \mathrm{d}[\hat{A}_1] & = \mathrm{d}\hat{t}(\frac{[\hat{\sigma}]}{\hat{\tau}} - \frac{[\hat{A}_1]}{\hat{\tau}}) \\ & +\sqrt{\frac{[\hat{\sigma}]}{\hat{\tau}}}\cdot\mathrm{d}\hat{W}_3 - \sqrt{\frac{[\hat{A}_1]}{\hat{\tau}}}\cdot\mathrm{d}\hat{W}_4 \\ \mathrm{d}[\hat{A}_2] & = \mathrm{d}\hat{t}(\frac{[\hat{A}_1]}{\hat{\tau}} - \frac{[\hat{A}_2]}{\hat{\tau}}) \\ & +\sqrt{\frac{[\hat{A}_1]}{\hat{\tau}}}\cdot\mathrm{d}\hat{W}_5 - \sqrt{\frac{[\hat{A}_2]}{\hat{\tau}}}\cdot\mathrm{d}\hat{W}_6 \\ \mathrm{d}[\hat{A}_3] & = \mathrm{d}\hat{t}(\frac{[\hat{A}_2]}{\hat{\tau}} - \frac{[\hat{A}_3]}{\hat{\tau}}) \\ & +\sqrt{\frac{[\hat{A}_2]}{\hat{\tau}}}\cdot\mathrm{d}\hat{W}_7 - \sqrt{\frac{[\hat{A}_3]}{\hat{\tau}}}\cdot\mathrm{d}\hat{W}_8 \end{cases}$$

Supplementary Table 2: **The parameters of the (non-nondimensionalised) general sigma factor circuit model.** Here, ˆ is used to denote parameters before nondimensionalisation. Dimensions for the parameters are given using minutes for time, and µM for species amounts, however, these specifics will not be used further.

Parameter	Description	Unit
$\hat{v}_0$	Transcriptional leakage of the operon.	µM·min^{-1}
$\hat{v}$	Maximal operon activity.	µM·min^{-1}
$\hat{I}$	Input signal magnitude.	-
$\hat{S}$	Degree of system self-activation.	-
$\hat{D}$	Degree of system self-deactivation.	-
$\hat{K}$	Activation threshold.	µM
$\hat{n}$	Hill coefficient (degree of non-linearity in the circuit).	-
$\hat{k}_{deg}$	Degradation/dilution rate.	min^{-1}
$\hat{\tau}$	Rate parameter of the LCT (length of the time-delay).	min

where $\mathrm{d}\hat{W}_i$ is a Wiener process. There exists one such noise term for each reaction (subscripts correspond to the order of the reactions in Supplementary Table 1). We will next describe the nondimensionalisation procedure where we reduce the number of parameters of this model, while preserving its dynamics. We start by considering the first SDE only:

$$\mathrm{d}[\hat{\sigma}] = \mathrm{d}\hat{t} \cdot (\hat{v}_0 + \hat{v}\frac{(\hat{I} \cdot \hat{S}[\hat{\sigma}])^{\hat{n}}}{(\hat{I} \cdot \hat{S}[\hat{\sigma}])^{\hat{n}} + (\hat{D}[\hat{A}_3])^{\hat{n}} + \hat{K}^{\hat{n}}} - \hat{k}_{deg}[\hat{\sigma}]) + \sqrt{...} \cdot \mathrm{d}\hat{W}_1 - \sqrt{...} \cdot \mathrm{d}\hat{W}_2.$$

Here, we multiply both sides by $\frac{\hat{k}_{deg}}{\hat{v}}$:

$$\mathrm{d}\frac{\hat{k}_{deg}[\hat{\sigma}]}{\hat{v}} = \mathrm{d}(\hat{k}_{deg} \cdot \hat{t}) \cdot (\frac{\hat{v}_0}{\hat{v}} + \frac{(\hat{I} \cdot \hat{S}[\hat{\sigma}])^{\hat{n}}}{(\hat{I} \cdot \hat{S}[\hat{\sigma}])^{\hat{n}} + (\hat{D}[\hat{A}_3])^{\hat{n}} + \hat{K}^{\hat{n}}} - \frac{\hat{k}_{deg}[\hat{\sigma}]}{\hat{v}}) + \frac{\hat{k}_{deg}}{\hat{v}}(\sqrt{...} \cdot \mathrm{d}\hat{W}_1 - \sqrt{...} \cdot \mathrm{d}\hat{W}_2).$$

Next, we multiply both the numerator and denominator of the fraction by $\frac{1}{K^n}$. Within the fraction, multiply the three terms (not identical to 1) by $(\frac{\hat{v}}{\hat{v}})^{\hat{n}}$ and $(\frac{\hat{k}_{deg}}{\hat{k}_{deg}})^{\hat{n}}$. This yields:

$$\mathrm{d}\frac{\hat{k}_{deg}[\sigma]}{\hat{v}} = \mathrm{d}(\hat{k}_{deg} \cdot \hat{t}) \cdot (\frac{\hat{v}_0}{\hat{v}} + \frac{(\frac{\hat{I}\cdot\hat{S}\cdot\hat{v}}{\hat{k}_{deg}\cdot\hat{K}}\frac{\hat{k}_{deg}[\hat{\sigma}]}{\hat{v}})^{n}}{(\frac{\hat{I}\cdot\hat{S}\cdot\hat{v}}{\hat{k}_{deg}\cdot\hat{K}}\frac{\hat{k}_{deg}[\hat{\sigma}]}{\hat{v}})^{n} + (\frac{\hat{D}\cdot\hat{v}}{\hat{k}_{deg}\cdot\hat{K}}\frac{\hat{k}_{deg}[\hat{A}_3]}{\hat{v}})^{n} + 1} - \frac{\hat{k}_{deg}[\hat{\sigma}]}{\hat{v}}) + \frac{\hat{k}_{deg}}{\hat{v}}(\sqrt{...} \cdot \mathrm{d}\hat{W}_1 - \sqrt{...} \cdot \mathrm{d}\hat{W}_2).$$

In a similar manner, the noise terms can be rewritten as:

$$\frac{\hat{k}_{deg}}{\hat{v}}\sqrt{\hat{v}_0 + \hat{v}\frac{(\hat{I} \cdot \hat{S}[\hat{\sigma}])^{\hat{n}}}{(\hat{I} \cdot \hat{S}[\hat{\sigma}])^{\hat{n}} + (\hat{D}[\hat{A}_3])^{\hat{n}} + \hat{K}^{\hat{n}}}}\mathrm{d}\hat{W}_1 = \frac{1}{\sqrt{\hat{v}}}\sqrt{\frac{\hat{v}_0}{\hat{v}} + \frac{(\frac{\hat{I}\cdot\hat{S}\cdot\hat{v}}{\hat{k}_{deg}\cdot\hat{K}}\frac{\hat{k}_{deg}[\hat{\sigma}]}{\hat{v}})^{\hat{n}}}{(\frac{\hat{I}\cdot\hat{S}\cdot\hat{v}}{\hat{k}_{deg}\cdot\hat{K}}\frac{\hat{k}_{deg}[\hat{\sigma}]}{\hat{v}})^{\hat{n}} + (\frac{\hat{D}\cdot\hat{v}}{\hat{k}_{deg}\cdot\hat{K}}\frac{\hat{k}_{deg}[\hat{A}_3]}{\hat{v}})^{\hat{n}} + 1}}\mathrm{d}\hat{W}_1$$

$$\frac{\hat{k}_{deg}}{\hat{v}}\sqrt{\hat{k}_{deg} \cdot [\hat{\sigma}]}\mathrm{d}\hat{W}_2 = \frac{\hat{k}_{deg}}{\sqrt{\hat{v}}}\sqrt{\frac{\hat{k}_{deg}[\hat{\sigma}]}{\hat{v}}}\mathrm{d}\hat{W}_2.$$

Finally, we consider the equations of the time-delay intermediaries:

$$\begin{cases} \mathrm{d}[\hat{A}_1] = dt(\frac{[\hat{\sigma}]}{\hat{\tau}} - \frac{[\hat{A}_1]}{\hat{\tau}}) + \sqrt{\frac{[\hat{\sigma}]}{\hat{\tau}}} \cdot \mathrm{d}\hat{W}_3 - \sqrt{\frac{[\hat{A}_1]}{\hat{\tau}}} \cdot \mathrm{d}\hat{W}_4 \\ \mathrm{d}[\hat{A}_2] = dt(\frac{[\hat{A}_1]}{\hat{\tau}} - \frac{[\hat{A}_2]}{\hat{\tau}}) + \sqrt{\frac{[\hat{A}_1]}{\hat{\tau}}} \cdot \mathrm{d}\hat{W}_5 - \sqrt{\frac{[\hat{A}_2]}{\hat{\tau}}} \cdot \mathrm{d}\hat{W}_6 \\ \mathrm{d}[\hat{A}_3] = dt(\frac{[\hat{A}_2]}{\hat{\tau}} - \frac{[\hat{A}_3]}{\hat{\tau}}) + \sqrt{\frac{[\hat{A}_2]}{\hat{\tau}}} \cdot \mathrm{d}\hat{W}_7 - \sqrt{\frac{[\hat{A}_3]}{\hat{\tau}}} \cdot \mathrm{d}\hat{W}_8 \end{cases}$$

$$\iff$$

$$\begin{cases} \mathrm{d}\frac{\hat{k}_{deg}\cdot[\hat{A}_1]}{\hat{v}} = d(\hat{k}_{deg}\cdot\hat{t})(\frac{\hat{k}_{deg}\cdot[\hat{\sigma}]}{\hat{v}}\frac{1}{\hat{k}_{deg}\hat{\tau}} - \frac{\hat{k}_{deg}\cdot[\hat{A}_1]}{\hat{v}}\frac{1}{\hat{k}_{deg}\hat{\tau}}) + \frac{\hat{k}_{deg}}{\sqrt{\hat{v}}}\sqrt{\frac{\hat{k}_{deg}\cdot[\hat{\sigma}]}{\hat{v}}\frac{1}{\hat{k}_{deg}\hat{\tau}}} \cdot \mathrm{dd}\hat{W}_3 \\ -\frac{\hat{k}_{deg}}{\sqrt{\hat{v}}}\sqrt{\frac{\hat{k}_{deg}\cdot[\hat{A}_1]}{\hat{v}}\frac{1}{\hat{k}_{deg}\hat{\tau}}} \cdot \mathrm{d}\hat{W}_4 \\ \mathrm{d}\frac{\hat{k}_{deg}\cdot[\hat{A}_2]}{\hat{v}} = d(\hat{k}_{deg}\cdot\hat{t})(\frac{\hat{k}_{deg}\cdot[\hat{A}_1]}{\hat{v}}\frac{1}{\hat{k}_{deg}\hat{\tau}} - \frac{\hat{k}_{deg}\cdot[\hat{A}_2]}{\hat{v}}\frac{1}{\hat{k}_{deg}\hat{\tau}}) + \frac{\hat{k}_{deg}}{\sqrt{\hat{v}}}\sqrt{\frac{\hat{k}_{deg}\cdot[\hat{A}_1]}{\hat{v}}\frac{1}{\hat{k}_{deg}\hat{\tau}}} \cdot \mathrm{d}\hat{W}_5 \\ -\frac{\hat{k}_{deg}}{\sqrt{\hat{v}}}\sqrt{\frac{\hat{k}_{deg}\cdot[\hat{A}_2]}{\hat{v}}\frac{1}{\hat{k}_{deg}\hat{\tau}}} \cdot \mathrm{d}\hat{W}_6 \\ \mathrm{d}\frac{\hat{k}_{deg}\cdot[\hat{A}_3]}{\hat{v}} = \mathrm{d}(\hat{k}_{deg}\cdot\hat{t})(\frac{\hat{k}_{deg}\cdot[\hat{A}_2]}{\hat{v}}\frac{1}{\hat{k}_{deg}\hat{\tau}} - \frac{\hat{k}_{deg}\cdot[\hat{A}_3]}{\hat{v}}\frac{1}{\hat{k}_{deg}\hat{\tau}}) + \frac{\hat{k}_{deg}}{\sqrt{\hat{v}}}\sqrt{\frac{\hat{k}_{deg}\cdot[\hat{A}_2]}{\hat{v}}\frac{1}{\hat{k}_{deg}\hat{\tau}}} \cdot \mathrm{d}\hat{W}_7 \\ -\frac{\hat{k}_{deg}}{\sqrt{\hat{v}}}\sqrt{\frac{\hat{k}_{deg}\cdot[\hat{A}_3]}{\hat{v}}\frac{1}{\hat{k}_{deg}\hat{\tau}}} \cdot \mathrm{d}\hat{W}_8. \end{cases}$$

Now, we can perform the following substitutions (note, here is where the noise scaling parameter η is introduced):

$$\begin{cases} [\sigma] &= \frac{\hat{k}_{deg}\cdot[\hat{\sigma}]}{\hat{v}} \\ [A_1] &= \frac{\hat{k}_{deg}\cdot[\hat{A}_1]}{\hat{v}} \\ [A_2] &= \frac{\hat{k}_{deg}\cdot[\hat{A}_2]}{\hat{v}} \\ [A_3] &= \frac{\hat{k}_{deg}\cdot[\hat{A}_3]}{\hat{v}} \\ t &= \hat{k}_{deg}\hat{t} \\ \eta W_i &= \frac{\hat{k}_{deg}}{\sqrt{\hat{v}}}\hat{W}_i \\ S &= \frac{\hat{S}\cdot\hat{I}\cdot\hat{v}}{\hat{k}_{deg}\cdot\hat{K}} \\ D &= \frac{\hat{D}\cdot\hat{v}}{\hat{k}_{deg}\cdot\hat{K}} \\ \tau &= \hat{k}_{deg}\hat{\tau} \\ v_0 &= \frac{\hat{v}_0}{\hat{v}} \\ n &= \hat{n} \end{cases}$$

which generate the following, nondimensionalised, set of SDEs:

$$
\begin{cases}
d[\sigma] & = dt \cdot (v_0 + \frac{(S[\sigma])^n}{(S[\sigma])^n + (D[A_3])^n + 1} - [\sigma]) \\
 & + \eta \sqrt{v_0 + \frac{(S[\sigma])^n}{(S[\sigma])^n + (D[A_3])^n + 1}} \cdot \mathrm{d}W_1 - \eta \sqrt{[\sigma]} \cdot \mathrm{d}W_2 \\
d[A_1] & = dt(\frac{[\sigma]}{\tau} - \frac{[A_1]}{\tau}) \\
 & + \eta \sqrt{\frac{[\sigma]}{\tau}} \cdot \mathrm{d}W_3 - \eta \sqrt{\frac{[A_1]}{\tau}} \cdot \mathrm{d}W_4 \\
d[A_2] & = dt(\frac{[A_1]}{\tau} - \frac{[A_2]}{\tau}) \\
 & + \eta \sqrt{\frac{[A_1]}{\tau}} \cdot \mathrm{d}W_5 - \eta \sqrt{\frac{[A_2]}{\tau}} \cdot \mathrm{d}W_6 \\
d[A_3] & = dt(\frac{[A_2]}{\tau} - \frac{[A_3]}{\tau}) \\
 & + \eta \sqrt{\frac{[A_2]}{\tau}} \cdot \mathrm{d}W_7 - \eta \sqrt{\frac{[A_3]}{\tau}} \cdot \mathrm{d}W_8.
\end{cases}
$$

A corresponding set of ODEs is also computed, and primarily used for steady state analysis:

$$
\begin{cases}
\frac{d[\sigma]}{dt} & = v_0 + \frac{(S[\sigma])^n}{(S[\sigma])^n + (D[A_3])^n + 1} - [\sigma] \\
\frac{d[A_1]}{dt} & = \frac{[\sigma]}{\tau} - \frac{[A_1]}{\tau} \\
\frac{d[A_2]}{dt} & = \frac{[A_1]}{\tau} - \frac{[A_2]}{\tau} \\
\frac{d[A_3]}{dt} & = \frac{[A_2]}{\tau} - \frac{[A_3]}{\tau}.
\end{cases}
$$

The reactions and parameters of the model after nondimensionalisation are provided in the Methods section.

Supplementary Note 3: Replication of results using Gillespie's algorithm

To make the model simulations directly comparable to the stability analysis (which is carried out in continuous space), and to allow for the reduction in parameters using nondimensionalisation, we used the chemical Langevin equation (CLE) to implement noise [1]. Continuous space is also required for our definition of inactive and active states (Supplementary Note 7.2). However, *stochastic chemical kinetics* (which we here simulate using Gillespie's algorithm) typically yields more realistic trajectories [2, 3]. Especially due to the general nature of our model, we consider the CLE a sound approach (checks also suggest that we are simulating the CLE in a noise regime where its approximations hold). However, to show that the results hold in discrete space, we used a non-nondimensionalised version of our model, simulated using the Gillespie algorithm, to recreate all of the response behaviours (Supplementary Figure 5). This demonstrates that the observed behaviours were not dependent on the CRN interpretation.

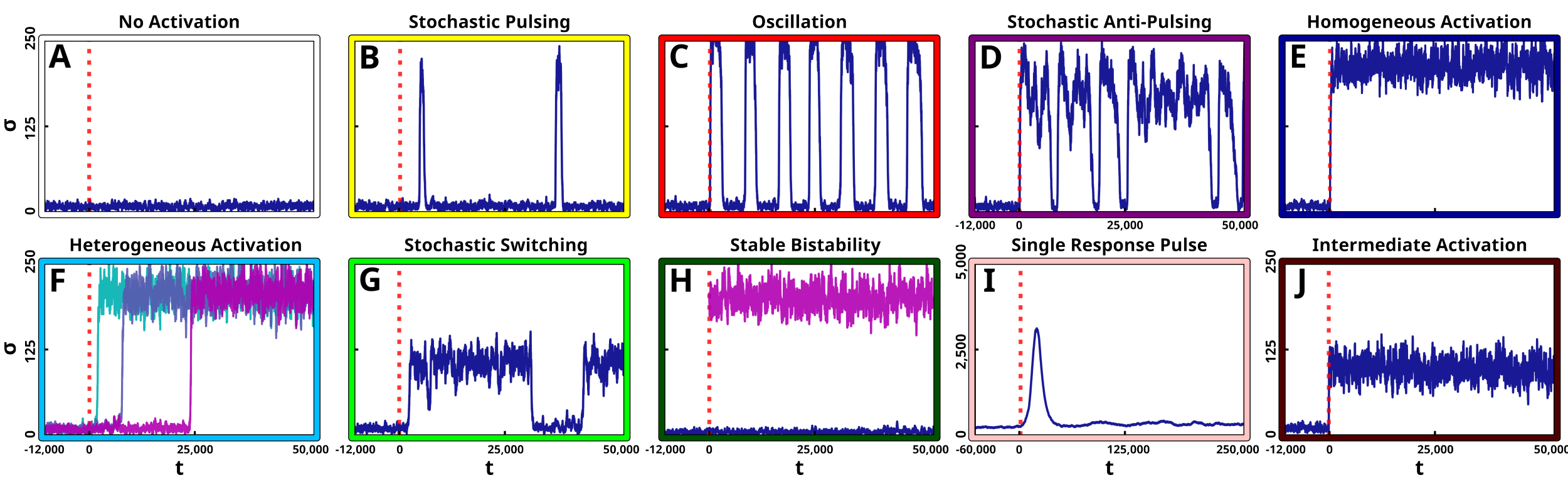

Supplementary Figure 5: **All the model behaviours can be recreated using Gillespie simulations.** Example simulations of the system's ten behaviours, but simulated using the Gillespie algorithm. The time scales in the various plots are different (k_{deg} was used to modulate copy numbers, and thus stochasticity, which also affected the time scales). Simulations were carried out using the non-nondimensionalised model, as described in Supplementary Table 1. The behaviours shown are: (A) No activation. (B) Stochastic pulsing. (C) Oscillation. (D) Stochastic anti-pulsing. (E) Homogeneous activation. (F) Heterogeneous activation. (G) Stochastic switching. (H) Stable Bistability. (I) Single response pulse. (J) Intermediate activation. Source code used to generate this figure can be found in the provided GitLab repository.

Next, we wanted to show that our primary results (that the model's behaviours can be mapped across parameter space) could also be recreated using Gillespie simulations. Here, we constructed a rudimentary version of our classifier (Supplementary Note 7), but which was based on Gillespie simulations. Using it, we mapped the model's behaviours (Supplementary Figure 6). We note that this Gillespie simulation-based map reproduces similar behavioural patterns to those which were observed in the CLE-based maps (described in Supplementary Note 9).

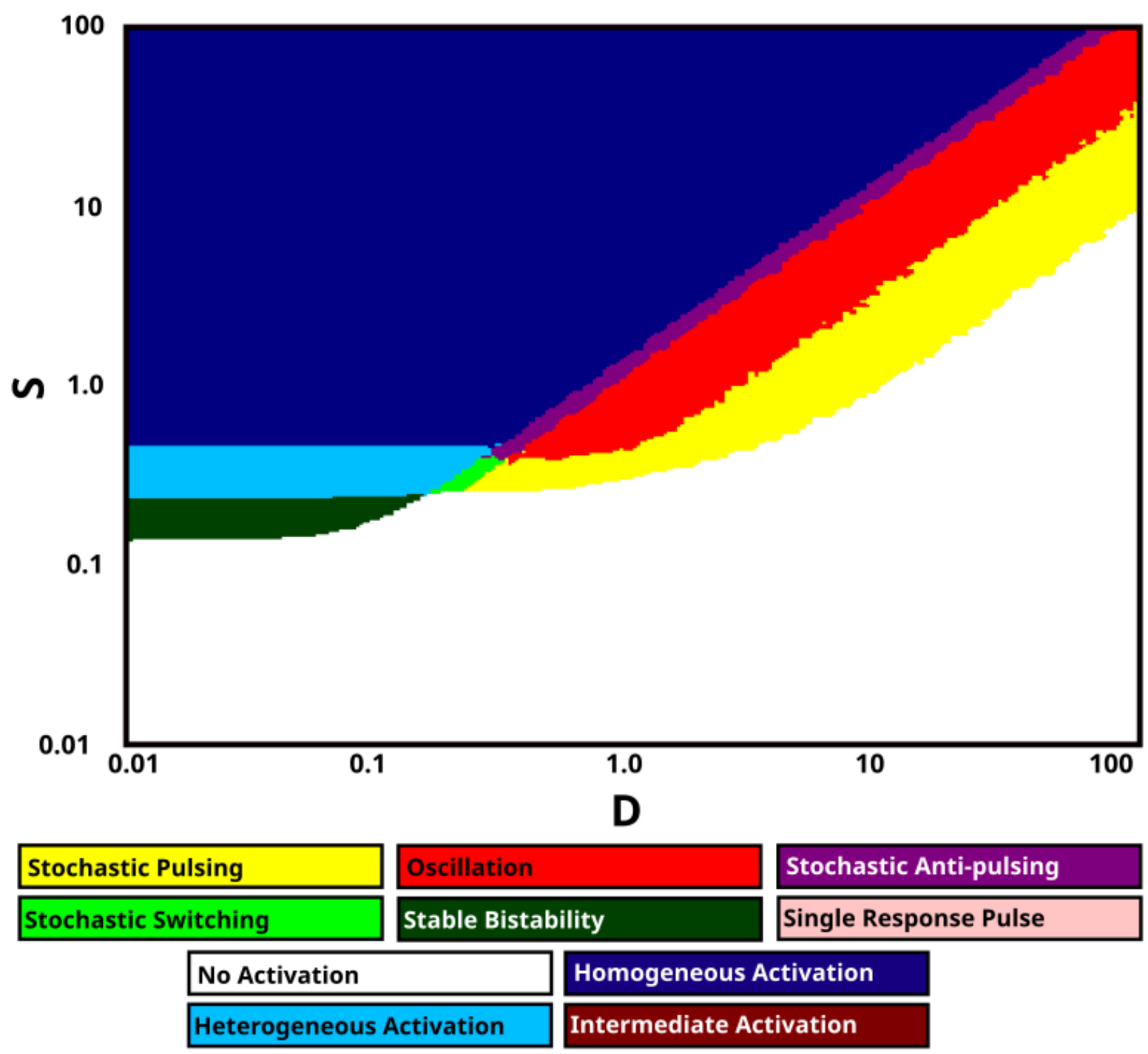

Supplementary Figure 6: **A Gillespie simulation-based classifier reproduces the behaviour maps generated by the CLE-based classifier.** Our Gillespie simulation-based classifier performs simulations based on the non-nondimensionalised model (Supplementary Note 2). This map shows the model behaviours in (logarithmic) S, D-space for the parameter set $(v_0, v, I, K, n, d, \tau) = (0.1, 2.0, 1.0, 15.0, 3, 0.01, 100.0)$. The map shows a similar behaviour distribution to that is generated by the CLE-based classifier (Supplementary Note 9). Source code used to generate this figure can be found in the provided GitLab repository.

Supplementary Note 4: Modelling the time delay

4.1 Determining the number of intermediaries in the linear chain trick

The simplest implementation of a time-delay is a discrete delay, which generates a *delay differential equation* (DDE) (such an implementation is investigated in Supplementary Note 4.2). Here, the delay is defined by a single parameter only (τ, the length of the delay). An alternative approach is to use a distributed delay, generating an *integro-differential equation* (IDE) [4–6]. It has been shown that distributed delay may produce qualitatively different results from discrete delays, and these differences may have important biological implications [7]. Distributed delays often depend on more than one parameter, e.g. the gamma distributed delay depends on a rate parameter τ (determining the length of the delay) and a shape parameter n. A gamma-distributed delay can be approximated using the *linear chain trick* (LCT), which replaces the delay term with a set of additional species that are produced and degraded at a linear rate [8–10]. This has the advantage that an IDE can be replaced with an ODE, which are significantly easier to analyse and simulate (in our stochastic case, an SIDE is replaced by an SDE). While the LCT depends on only a single parameter (τ, the rate), in its implementation, one also has to determine the number of intermediaries introduced (this number corresponds to the shape parameter, n, of the corresponding gamma-distributed delay). We postulated that the shape would only have a minor effect on the behaviour, and that the complexity added to the model by making the number of intermediaries variable would not merit the introduction of an additional parameter. We thus decided to choose a fixed number of intermediaries.

According to [11], for two LCT implementations, with rate parameters τ_1 and τ_2, and shape parameters N_1 and N_2, the length of their delays is equivalent if

$$\frac{\tau_1}{N_1} = \frac{\tau_2}{N_2}.$$

We test this in practice by investigating the period of our general model for a range of values of τ and N (Supplementary Figure 7). Our tests show that the estimate improves as N becomes large. Especially, only for $N = 1$ or $N = 2$ is there a noticeable difference. Hence, we select $N = 3$ (the smallest value for which the estimate holds well). Finally, we also note that (in practical simulations) the A_1 and A_3 variables are highly correlated (with the latter being a delay of the former) (Supplementary Figure 8). This further suggests that the choice of the number of intermediaries has little effect on the model.

4.2 Replication of results using a discrete delay

In addition to our main model, where we use a distributed delay implemented through the LCT, we also developed a model depending on a discrete delay. This model contains only two reactions (Supplementary Table 3). The parameters are identical to those of the distributed delay implementation. Finally, the RRE generates a single ODE:

$$\frac{d[\sigma]}{dt} = v_0 + \frac{(S[\sigma](t))^n}{(S[\sigma](t))^n + (D[\sigma](t-\tau))^n + 1} - [\sigma](t)$$

with a corresponding SDE generated by the CLE:

$$\begin{aligned} d[\sigma] = dt \cdot (v_0 &+ \frac{(S[\sigma](t))^n}{(S[\sigma](t))^n + (D[\sigma](t-\tau))^n + 1} - [\sigma](t)) \\ &+ \eta\sqrt{v_0 + \frac{(S[\sigma](t))^n}{(S[\sigma](t))^n + (D[\sigma](t-\tau))^n + 1}} \cdot \mathrm{d}W_1 - \eta\sqrt{[\sigma](t)} \cdot \mathrm{d}W_2. \end{aligned}$$

Finding steady states (and their stability) is much harder for DDEs than ODEs. In addition, there are many fewer algorithms available for simulations of DDEs and SDDEs (as compared to ODEs and SDEs). Hence,

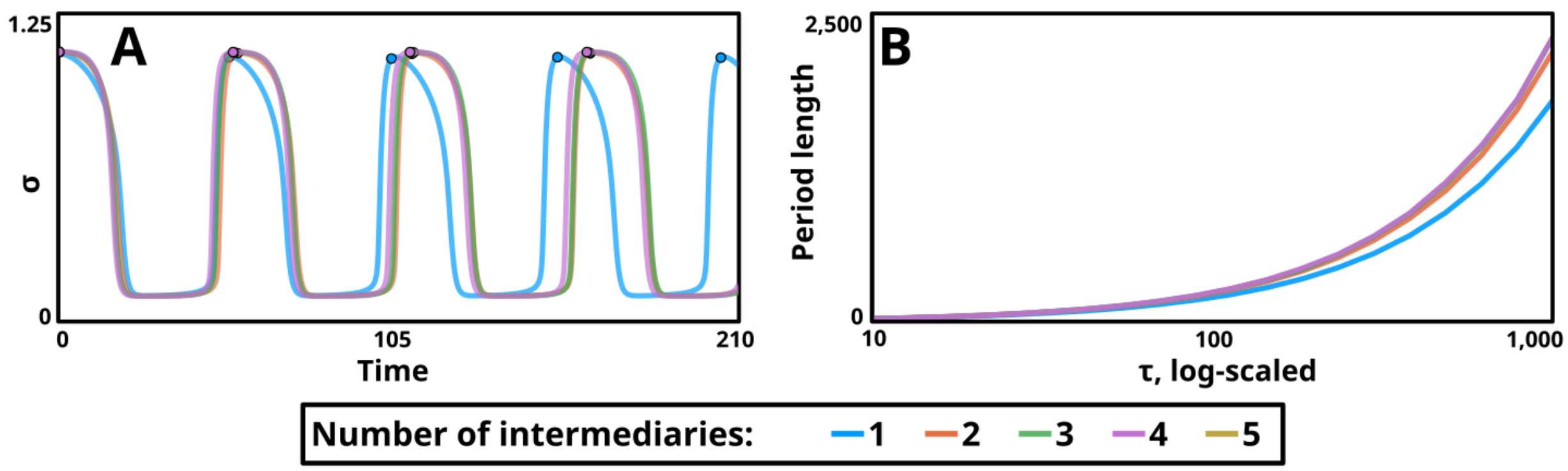

Supplementary Figure 7: **The number of intermediaries selected in the LCT has a small effect on the model's time-delay.** (A) Deterministic (using the reaction rate equation (RRE) interpretation) simulations of the model, implemented using various values of N (intermediaries in the LCT). All parameters are identical, except for τ, which is set according to the rule $\frac{\tau_1}{N_1} = \frac{\tau_2}{N_2}$, ensuring similar time-delay in all model implementations. As the oscillations persist, the simulations (especially the $N = 1$ and $N = 2$ ones) get desynchronised (B) For a range of values of τ, the period length is measured for model implementations using $N = 1, 2, 3, 4, 5$. As τ grows large, the period lengths start to diverge. However, this difference is most distinct for $N = 1$ and $N = 2$. The previously mentioned rule is used to produce similar τ values for the different implementations, with the x-axis indicating the value of τ for $N = 1$. Source code used to generate this figure can be found in the provided GitLab repository.

Supplementary Table 3: **The reactions of the discrete delay implementation of the general sigma factor model.** Here, (t) is added at the end of species concentrations, to distinguish which terms are subject to a time-delay.

Description	Reaction	Rate	Propensity
Production	$\varnothing \longrightarrow \sigma$	$v_0 + \frac{(S[\sigma](t))^n}{(S[\sigma](t))^n + (D[\sigma](t-\tau))^n + 1}$	$v_0 + \frac{(S[\sigma](t))^n}{(S[\sigma](t))^n + (D[\sigma](t-\tau))^n + 1}$
Degradation/dilution	$\sigma \longrightarrow \varnothing$	1	$[\sigma](t)$

we will limit our analysis of this model to demonstrating its ability to generate the ten different response behaviours observed in the distributed delay implementation of our model (Supplementary Figure 9). For completeness, we also compare simulations of the model where we have implemented the delay through the LCT and using a discrete delay. The simulations use the same parameter set (where τ has been adjusted in the LCT case according to its number of intermediaries and the rule in Supplementary Note 4.1). Here, we note that while the simulations are similar, the two approaches generate slightly different shapes of the oscillation (Supplementary Figure 10).

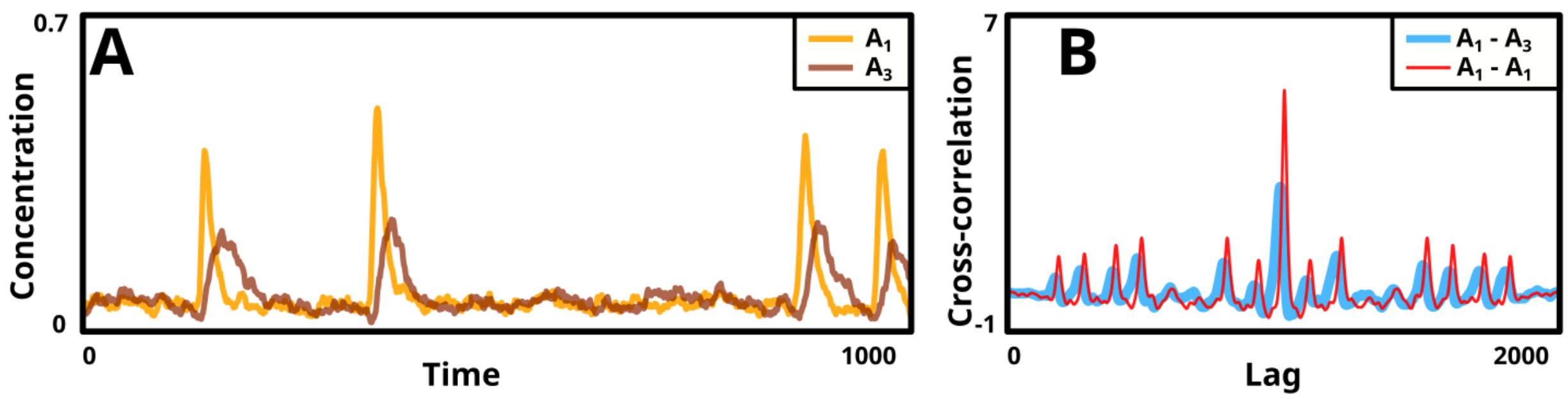

Supplementary Figure 8: **The A_1 and A_3 components can be compared using cross-correlation.** (A) The concentration of the A_1 (yellow) and A_3 (brown) components throughout a single (stochastic pulsing) simulation (input active throughout the simulation). The two trajectories are closely correlated. (B) The cross-correlation between A_1 and A_3 (blue) and between A_1 and itself (red). The distinct peak in the middle of the diagram indicates that the two curves are closely correlated (indeed, the A_1 to A_3 correlation is almost as good as A_1 to itself). The slight offset between the two curves along the x-axis indicates the length of the delay between the two components. Source code used to generate this figure can be found in the provided GitLab repository.

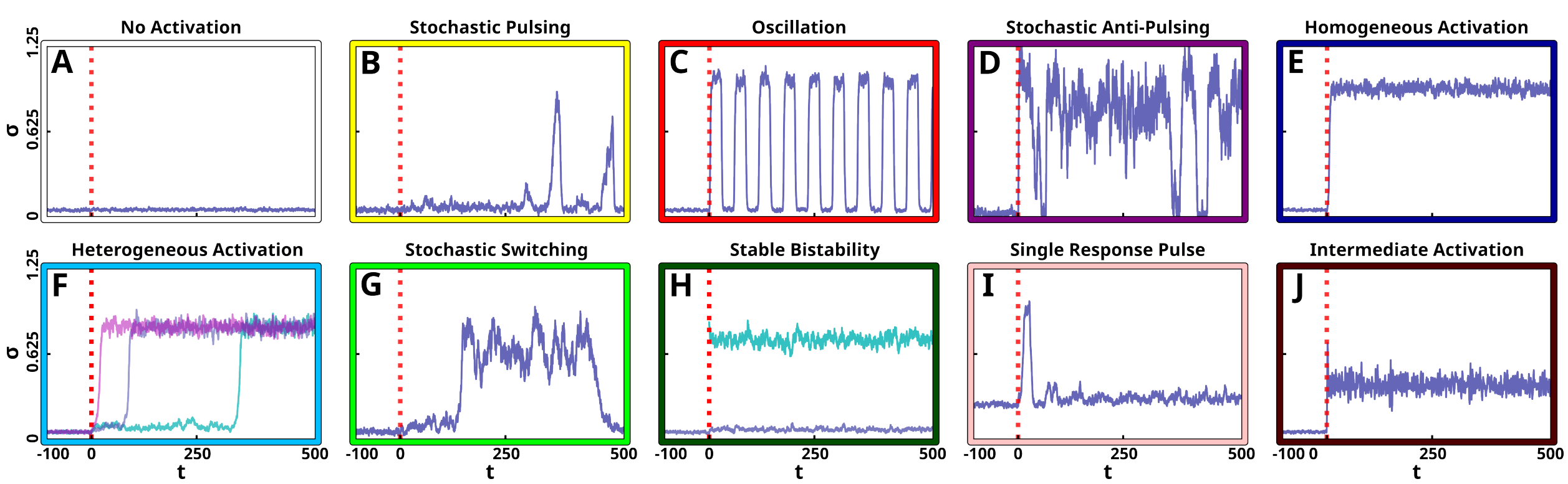

Supplementary Figure 9: **All the model behaviours can be recreated using the discrete delay implementation of the model.** Example simulations of the system's ten behaviours, but simulated using the discrete delay model. The behaviours shown are: (A) No activation. (B) Stochastic pulsing. (C) Oscillation. (D) Stochastic anti-pulsing. (E) Homogeneous activation. (F) Heterogeneous activation. (G) Stochastic switching. (H) Stable Bistability. (I) Single response pulse. (J) Intermediate activation. Source code used to generate this figure can be found in the provided GitLab repository.

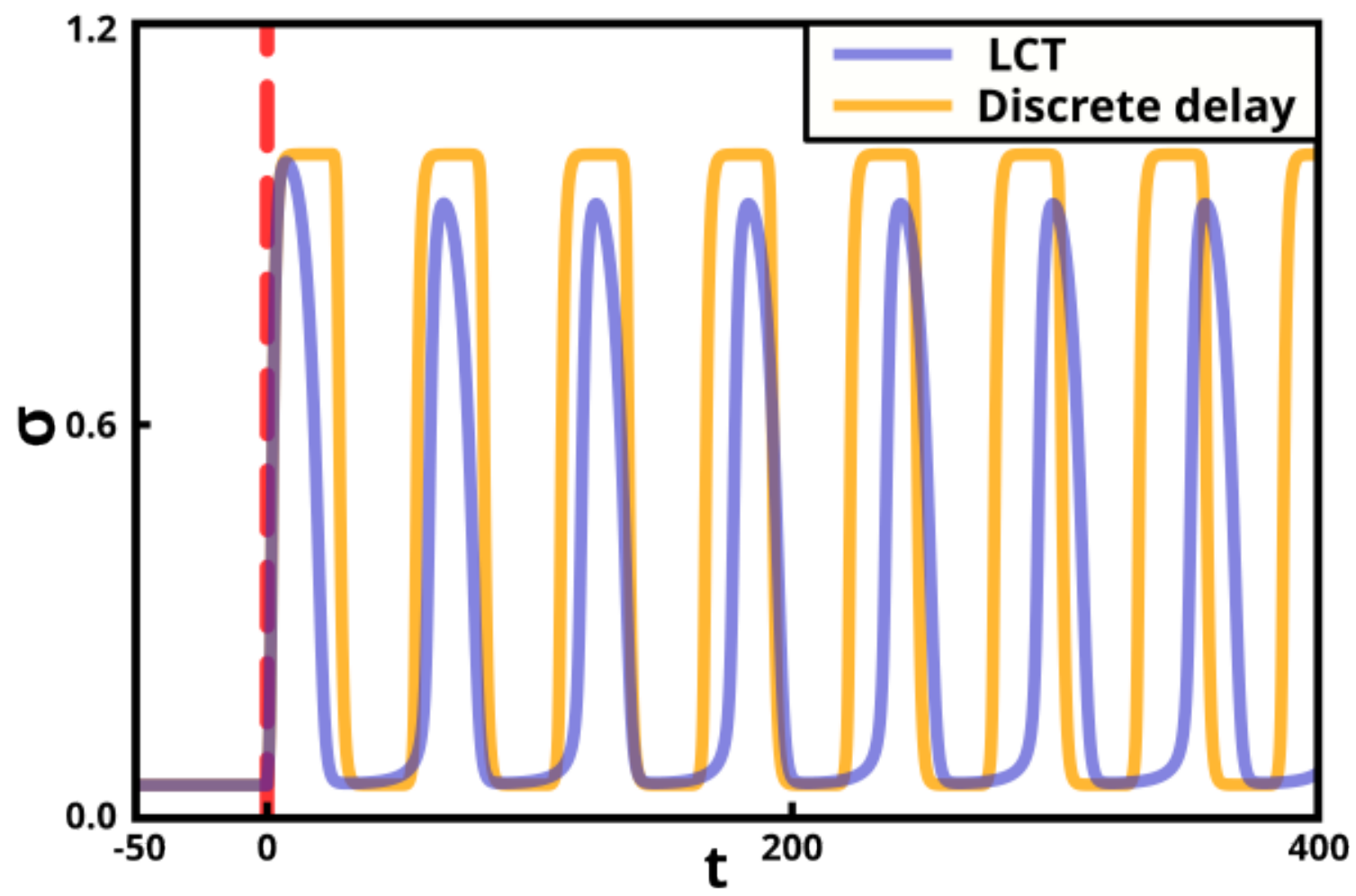

Supplementary Figure 10: **The same parameter set generates similar behaviours when simulated using the LCT and using a discrete implementation of the time delay.** Model simulations for $(S, D, v_0, n, \eta) = (5, 10, 0.05, 2, 0)$. For the discrete delay, $\tau = 25$ and for the LCT implementation $\tau = 25/3$ (which should yield similar effective time delays, according to the rule presented in Supplementary Note 4.1). The two models yield similar simulations, albeit with slightly altered shapes of the oscillation. Source code used to generate this figure can be found in the provided GitLab repository.

Supplementary Note 5: Alternative model formulations

Throughout the paper, we will use the model presented in Supplementary Note 2. However, to show that our results are general to the mixed positive/negative feedback loop circuit, and not specific to the implementation we used, we here consider two alternative model formulations.

5.1 Model with alternative mixed activating/repressive Hill connection

First, we consider a model with an alternative formulation for the mixed activating/repressive Hill connection. Here, instead of using $v_0 + \frac{(S[\sigma])^n}{(S[\sigma])^n + (D[A_3])^n + 1}$ as the activation function, we will use $v_0 + \frac{(S[\sigma])^n}{(S[\sigma])^n+1}\frac{1}{(D[A_3])^n+1}$. This generates the chemical reaction network defined by the reactions in Supplementary Table 4.

Supplementary Table 4: **The reactions of the (nondimensionalised) alternative sigma factor circuit model.** By changing the form at which σ is produced under mixed activation, we generate an alternative implementation of the mixed positive/negative feedback loop. The parameters have the same interpretation as presented in Table 2.

Description	Reaction	Rate	Propensity
Production	$\varnothing \longrightarrow \sigma$	$v_0 + \frac{(S[\sigma])^n}{(S[\sigma])^n+1}\frac{1}{(D[A_3])^n+1}$	$v_0 + \frac{(S[\sigma])^n}{(S[\sigma])^n+1}\frac{1}{(D[A_3])^n+1}$
Degradation/dilution	$\sigma \longrightarrow \varnothing$	$\hat{k}_{deg}$	$\hat{k}_{deg} \cdot [\hat{\sigma}]$
Production (LCT)	$\varnothing \longrightarrow A_1$	$\frac{[\hat{\sigma}]}{\hat{\tau}}$	$\frac{[\hat{\sigma}]}{\hat{\tau}}$
	$\varnothing \longrightarrow A_2$	$\frac{[\hat{A}_1]}{\hat{\tau}}$	$\frac{[\hat{A}_1]}{\hat{\tau}}$
	$\varnothing \longrightarrow A_3$	$\frac{[\hat{A}_2]}{\hat{\tau}}$	$\frac{[\hat{A}_2]}{\hat{\tau}}$
Degradation/dilution (LCT)	$A_1 \longrightarrow \varnothing$	$\frac{1}{\hat{\tau}}$	$\frac{[\hat{A}_1]}{\hat{\tau}}$
	$A_2 \longrightarrow \varnothing$	$\frac{1}{\hat{\tau}}$	$\frac{[\hat{A}_2]}{\hat{\tau}}$
	$A_3 \longrightarrow \varnothing$	$\frac{1}{\hat{\tau}}$	$\frac{[\hat{A}_3]}{\hat{\tau}}$

Using the chemical Langevin equation, this model generates the following set of SDEs:

$$
\begin{cases}
d[\sigma] & = dt \cdot (v_0 + \frac{(S[\sigma])^n}{(S[\sigma])^n+1}\frac{1}{(D[A_3])^n+1} - [\sigma]) \\
& +\eta\sqrt{v_0 + \frac{(S[\sigma])^n}{(S[\sigma])^n+1}\frac{1}{(D[A_3])^n+1}} \cdot \mathrm{d}W_1 - \eta\sqrt{[\sigma]} \cdot \mathrm{d}W_2 \\
d[A_1] & = dt(\frac{[\sigma]}{\tau} - \frac{[A_1]}{\tau}) \\
& +\eta\sqrt{\frac{[\sigma]}{\tau}} \cdot \mathrm{d}W_3 - \eta\sqrt{\frac{[A_1]}{\tau}} \cdot \mathrm{d}W_4 \\
d[A_2] & = dt(\frac{[A_1]}{\tau} - \frac{[A_2]}{\tau}) \\
& +\eta\sqrt{\frac{[A_1]}{\tau}} \cdot \mathrm{d}W_5 - \eta\sqrt{\frac{[A_2]}{\tau}} \cdot \mathrm{d}W_6 \\
d[A_3] & = dt(\frac{[A_2]}{\tau} - \frac{[A_3]}{\tau}) \\
& +\eta\sqrt{\frac{[A_2]}{\tau}} \cdot \mathrm{d}W_7 - \eta\sqrt{\frac{[A_3]}{\tau}} \cdot \mathrm{d}W_8.
\end{cases}
$$

To demonstrate that our results hold for this implementation of the mixed positive/negative feedback loop, we replicate all ten behaviours (as presented in Figure 2) using it (Supplementary Figure 11).

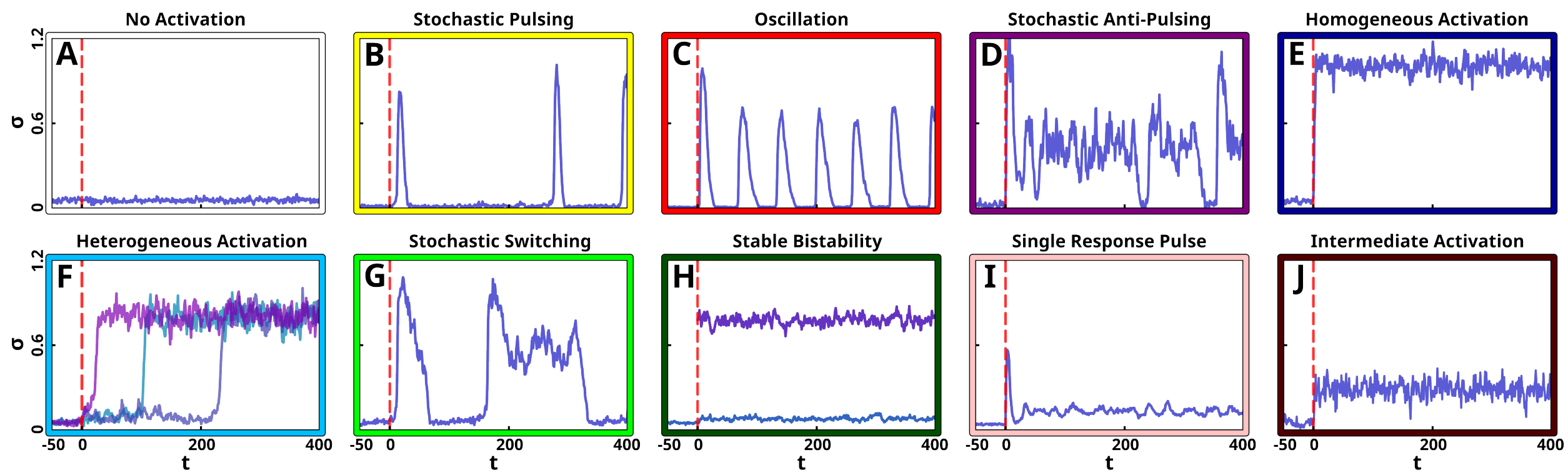

Supplementary Figure 11: **All the model behaviours can be recreated using an alternative model implementation.** Example simulations of the system's ten behaviours, but simulated using the alternative model presented in Supplementary Note 5.1. The behaviours shown are: (A) No activation. (B) Stochastic pulsing. (C) Oscillation. (D) Stochastic anti-pulsing. (E) Homogeneous activation. (F) Heterogeneous activation. (G) Stochastic switching. (H) Stable Bistability. (I) Single response pulse. (J) Intermediate activation. Source code used to generate this figure can be found in the provided GitLab repository.

5.2 Model with alternative implementation of the negative feedback

Next, we consider a model with an alternative formulation of how A_3 inhibits the production of σ. Here, A_3 directly subtracts how much σ is available for driving the production reaction. Instead of using $v_0 + \frac{(S[\sigma])^n}{(S[\sigma])^n+(D[A_3])^n+1}$ as the activation function, we will use $v_0+\frac{(S\cdot softplus_{10}([\sigma]-[A_3]))^n}{(S\cdot softplus_{10}([\sigma]-[A_3]))^n+1}$, where $softplus_{10}(x) = log(1+e^{10x})/10$ (essentially a smooth approximation of $max(0,x)$). This generates the chemical reaction network defined by the reactions in Supplementary Table 5.

Supplementary Table 5: **The reactions of the (nondimensionalised) alternative sigma factor circuit model.** By changing the form at which σ is produced under mixed activation, we generate an alternative implementation of the mixed positive/negative feedback loop. The parameters have the same interpretation as presented in Table 2.

Description	Reaction	Rate	Propensity
Production	$\varnothing \longrightarrow \sigma$	$v_0 + \frac{(S\cdot softplus_{10}([\sigma]-[A_3]))^n}{(S\cdot softplus_{10}([\sigma]-[A_3]))^n+1}$	$v_0 + \frac{(S\cdot softplus_{10}([\sigma]-[A_3]))^n}{(S\cdot softplus_{10}([\sigma]-[A_3]))^n+1}$
Degradation/dilution	$\sigma \longrightarrow \varnothing$	$\hat{k}_{deg}$	$\hat{k}_{deg} \cdot [\hat{\sigma}]$
Production (LCT)	$\varnothing \longrightarrow A_1$	$\frac{[\hat{\sigma}]}{\hat{\tau}}$	$\frac{[\hat{\sigma}]}{\hat{\tau}}$
	$\varnothing \longrightarrow A_2$	$\frac{[\hat{A}_1]}{\hat{\tau}}$	$\frac{[\hat{A}_1]}{\hat{\tau}}$
	$\varnothing \longrightarrow A_3$	$\frac{[\hat{A}_2]}{\hat{\tau}}$	$\frac{[\hat{A}_2]}{\hat{\tau}}$
Degradation/dilution (LCT)	$A_1 \longrightarrow \varnothing$	$\frac{1}{\hat{\tau}}$	$\frac{[\hat{A}_1]}{\hat{\tau}}$
	$A_2 \longrightarrow \varnothing$	$\frac{1}{\hat{\tau}}$	$\frac{[\hat{A}_2]}{\hat{\tau}}$
	$A_3 \longrightarrow \varnothing$	$\frac{1}{\hat{\tau}}$	$\frac{[\hat{A}_3]}{\hat{\tau}}$

Using the chemical Langevin equation, this model generates the following set of SDEs:

$$\begin{cases} d[\sigma] &= dt \cdot (v_0 + \frac{(S \cdot softplus_{10}([\sigma]-[A_3]))^n}{(S \cdot softplus_{10}([\sigma]-[A_3]))^n+1} - [\sigma]) \\ & +\eta\sqrt{v_0 + \frac{(S \cdot softplus_{10}([\sigma]-[A_3]))^n}{(S \cdot softplus_{10}([\sigma]-[A_3]))^n+1}} \cdot \mathrm{d}W_1 - \eta\sqrt{[\sigma]} \cdot \mathrm{d}W_2 \\ d[A_1] &= dt(\frac{[\sigma]}{\tau} - \frac{[A_1]}{\tau}) \\ & +\eta\sqrt{\frac{[\sigma]}{\tau}} \cdot \mathrm{d}W_3 - \eta\sqrt{\frac{[A_1]}{\tau}} \cdot \mathrm{d}W_4 \\ d[A_2] &= dt(\frac{[A_1]}{\tau} - \frac{[A_2]}{\tau}) \\ & +\eta\sqrt{\frac{[A_1]}{\tau}} \cdot \mathrm{d}W_5 - \eta\sqrt{\frac{[A_2]}{\tau}} \cdot \mathrm{d}W_6 \\ d[A_3] &= dt(\frac{[A_2]}{\tau} - \frac{[A_3]}{\tau}) \\ & +\eta\sqrt{\frac{[A_2]}{\tau}} \cdot \mathrm{d}W_7 - \eta\sqrt{\frac{[A_3]}{\tau}} \cdot \mathrm{d}W_8. \end{cases}$$

To demonstrate that our results hold for this implementation of the mixed positive/negative feedback loop, we replicate all ten behaviours (as presented in Figure 2) using it (Supplementary Figure 12).

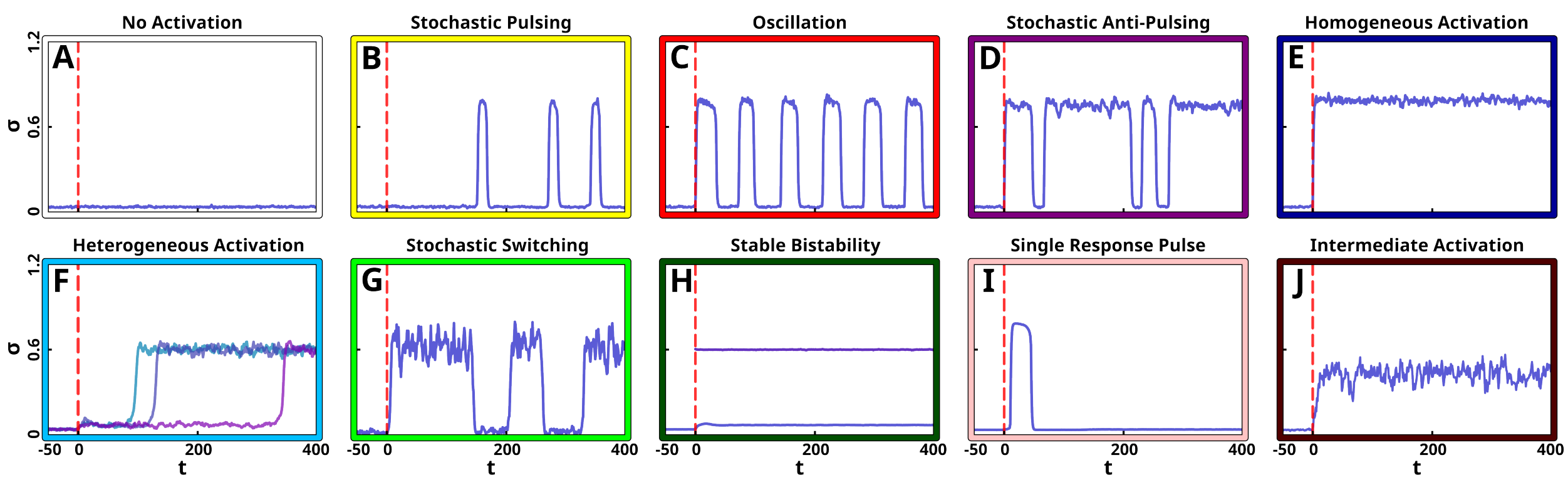

Supplementary Figure 12: **All the model behaviours can be recreated using an alternative model implementation.** Example simulations of the system's ten behaviours, but simulated using the alternative model presented in Supplementary Note 5.2. The behaviours shown are: (A) No activation. (B) Stochastic pulsing. (C) Oscillation. (D) Stochastic anti-pulsing. (E) Homogeneous activation. (F) Heterogeneous activation. (G) Stochastic switching. (H) Stable Bistability. (I) Single response pulse. (J) Intermediate activation. Source code used to generate this figure can be found in the provided GitLab repository.

Supplementary Note 6: Steady state analysis

6.1 Steady state computations

There exists a range of approaches for finding the steady states of a system of ODEs. Here, we will use algebraic manipulation to find a single polynomial whose roots correspond to the steady states of our system. Polynomial roots, and hence our model's steady states, can be computed reliably.

We start with the model's ODE system (generated through the reaction rate equations) and set the derivatives equal to 0 (to find the steady states):

$$\begin{cases} 0 &= v_0 + \frac{(S[\sigma])^n}{(S[\sigma])^n + (D[A_3])^n + 1} - [\sigma] \\ 0 &= \frac{[\sigma]}{\tau} - \frac{[A_1]}{\tau} \\ 0 &= \frac{[A_1]}{\tau} - \frac{[A_2]}{\tau} \\ 0 &= \frac{[A_2]}{\tau} - \frac{[A_3]}{\tau}. \end{cases}$$

here, the last three equations trivially yield $[A_i] = [\sigma]$, we thus only need to solve:

$$0 = v_0 + \frac{(S[\sigma])^n}{(S[\sigma])^n + (D[\sigma])^n + 1} - [\sigma]$$

which we can rewrite as:

$$(v_0 - [\sigma])((S[\sigma])^n + (D[\sigma])^n + 1) = (S[\sigma])^n$$
$$\Longleftrightarrow$$
$$(S^n + D^n)[\sigma]^{n+1} - (S^n + v_0 \cdot S^n + v_0 \cdot D^n)[\sigma]^n + [\sigma] - v_0 = 0$$

which gives us our desired polynomial. System steady states values ($[\sigma]^*$) can be found as this polynomial's roots. Furthermore, according to Descartes' rule of signs, this polynomial has at most three real roots, setting a limit for the maximum number of steady states of our system. Since all three steady states cannot be stable we are left with the alternatives of either zero, one, or two stable steady states.

6.2 Nullcline analysis

Nullclines (curves along which the rate of change, with respect to a specified system variable, is 0) are commonly used during phase space analysis. In this section, we will first compute our system's nullclines, and then investigate how their properties depend on the system's parameters. The nullclines will also be shown in Supplementary Note 8 and they are important for our classification of the system's behaviour (Supplementary Note 7).

The nullclines can be computed by solving the equations $[\dot{\sigma}] = 0$ and $[\dot{A}_i] = 0$ (generating the curves $NC_\sigma([\sigma])$ and $NC_{A_i}([\sigma])$, respectively). Let's briefly consider the LCT with N intermediaries:

$$\begin{cases} 0 &= v_0 + \frac{(S[\sigma])^n}{(S[\sigma])^n + (D[A_N])^n + 1} - [\sigma] \\ 0 &= \frac{[\sigma]}{\tau} - \frac{[A_i]}{\tau} \end{cases}$$

The second set of nullclines are, trivially,

$$[A_i] = [\sigma].$$

The first one can be derived as:

$$([\sigma] - v_0)((S[\sigma])^n + (D[A_N])^n + 1) = (S[\sigma])^n$$
$$\iff$$
$$(D[A_N])^n = \frac{(S[\sigma])^n}{[\sigma] - v_0} - (S[\sigma])^n - 1$$
$$\iff$$
$$[A_N] = \frac{1}{D}\sqrt[n]{(S[\sigma])^n(\frac{1}{[\sigma] - v_0} - 1) - 1}$$

We note that the root of $NC_\sigma([\sigma])$ is not defined for negative arguments. While the initial equations might have had a solution for these values, they would result in $[A_N] < 0$. Since the phase space of a CRN is limited to non-negative species concentrations, these values of the nullcline are not of interest. We can safely take the root, and ignore the nullcline where the root is undefined.

We will use three intermediaries in our implementation of the LCT, however, for phase space analysis we will display the system in $[\sigma]$-$[A_3]$ space (ignoring the $[A_1]$ and $[A_2]$ dimensions). The two relevant nullclines are:

$$[A_3] = NC_\sigma([\sigma]) = \frac{1}{D}\sqrt[n]{(S[\sigma])^n(\frac{1}{[\sigma] - v_0} - 1) - 1}$$

$$[A_3] = NC_{A_3}([\sigma]) = [\sigma].$$

These intersect either once or thrice (Supplementary Note 6.1), producing a system with either one or three steady states (Supplementary Figure 13).

While the curve $NC_A([\sigma])$ is unaffected by the parameters (S, D, τ, v_0, n, and η), their values do affect the curve NC_σ. Changing S (the degree of system self-activation) changes its shape. For large or small values of S, NC_σ is a descending line, permitting only a single steady state. Only for intermediate values of S is bistability possible (Supplementary Figure 13B). For $S = 0$ (corresponding to a system with no input), NC_σ is a straight line at $[\sigma] = v_0$ (yielding a single steady state at $([\sigma], [A_i]) = (v_0, v_0)$ for the system before input). Changing D will only scale NC_σ, but not affect its shape further (Supplementary Figure 13C). For some critical values of S (where the system is close to, or in, the bistability region) v_0 may affect the shape of NC_σ. For most values of S, however, modulating v_0 has little effect on NC_σ (Supplementary Figure 13D). Increasing n increases the sharpness of the curve (Supplementary Figure 13E). Finally, η and τ do not affect NC_σ, however, τ may affect stability properties.

6.3 Bifurcation analysis

Additional information of our system can be inferred by considering its bifurcation diagrams. These describe how the system's steady states depends on its parameters. Here, we will only analyse the bifurcation diagrams with respect to the parameter S. This parameter describes the degree of input (or stress) to the system (as well as its degree of self-activation), and the bifurcation diagrams thus show how the steady states of the system are affected by the input's magnitude. We note two types of bifurcation diagrams (which one is observed mainly depends on the value of D, Supplementary Figure 14G). One displays bistability for intermediate values of S (this is known as a bistable switch, Supplementary Figure 14A,D)). The other has a single steady state for all values of S (Supplementary Figure 14C,F). For some parameter values, the bistable diagram may instead be monostable, with the two larger valued steady states being unstable (Supplementary Figure 14B,E). The value of v_0 has little effect on the single steady state diagrams, but affects the shape of the bistable switch (Supplementary Figure 14H). Next, n also affects the shape of the bistable switch (which requires $n > 1$, Supplementary Figure 14I). Finally, the steady state values are unaffected by the value of τ, but for large time-delays the single steady state diagrams develop an increasing region of instability (corresponding to an oscillation) (Supplementary Figure 14J). All bifurcation diagrams are computed using the BifurcationKit.jl Julia package [12].

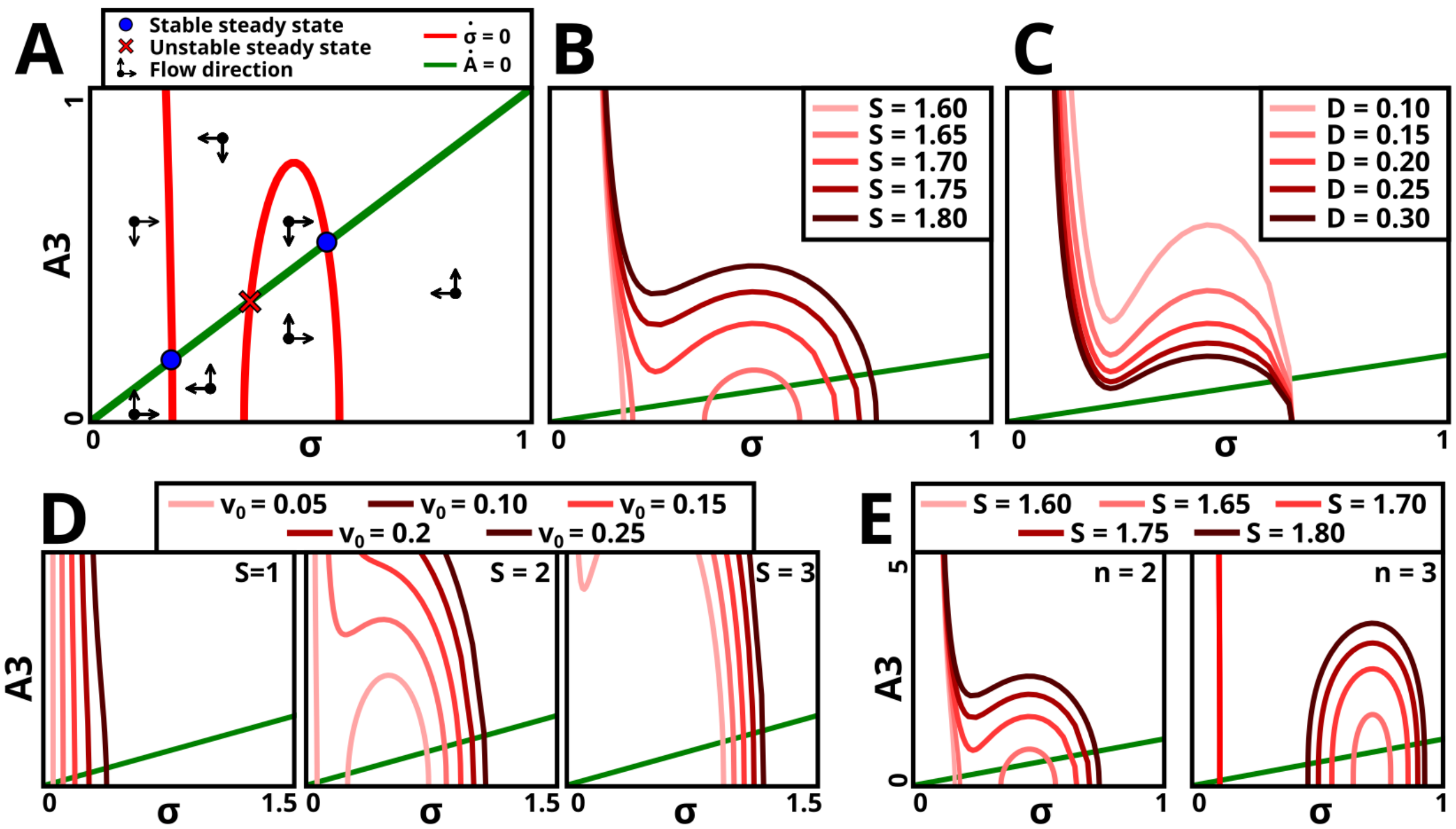

Supplementary Figure 13: **The model's nullclines plotted in phase space.** The two nullclines NC_σ (shades of red) and the NC_A (green). (A) The steady states of the system can be found as the nullclines' intersections. Also, in each region of the phase space, as separated by the nullclines, the direction of flow of the system can be determined, this is marked by the arrows. (B) The parameter S affects the shape of NC_σ. For low and high S there is only one steady state (with small and large $[\sigma]$, respectively). Only for intermediate values of S is the system bistable. (C) Modulating the parameter D only scales NC_σ, but does not affect the shape. (D) For small or large values of S, changing v_0 only has a minor effect on NC_σ. However, for intermediate values of S, modulating v_0 changes the shape. (E) Increasing n makes the NC_σ sharper, emphasising bistability. The parameters τ and η do not affect NC_σ. Finally, we note that our analysis here is primarily carried out as if our 4-variable system (with variables $\sigma(t)$, $A_1(t)$, $A_2(t)$ and $A_3(t)$) is a 2-variable system (with variables $\sigma(t)$ and $A_3(t)$). However, the dynamics of the 4-variables is *very* similar to the 2-variable one with only a single intermediate (A) (Supplementary Figure 8), and that these two systems have identical nullclines. Source code used to generate this figure can be found in the provided GitLab repository.

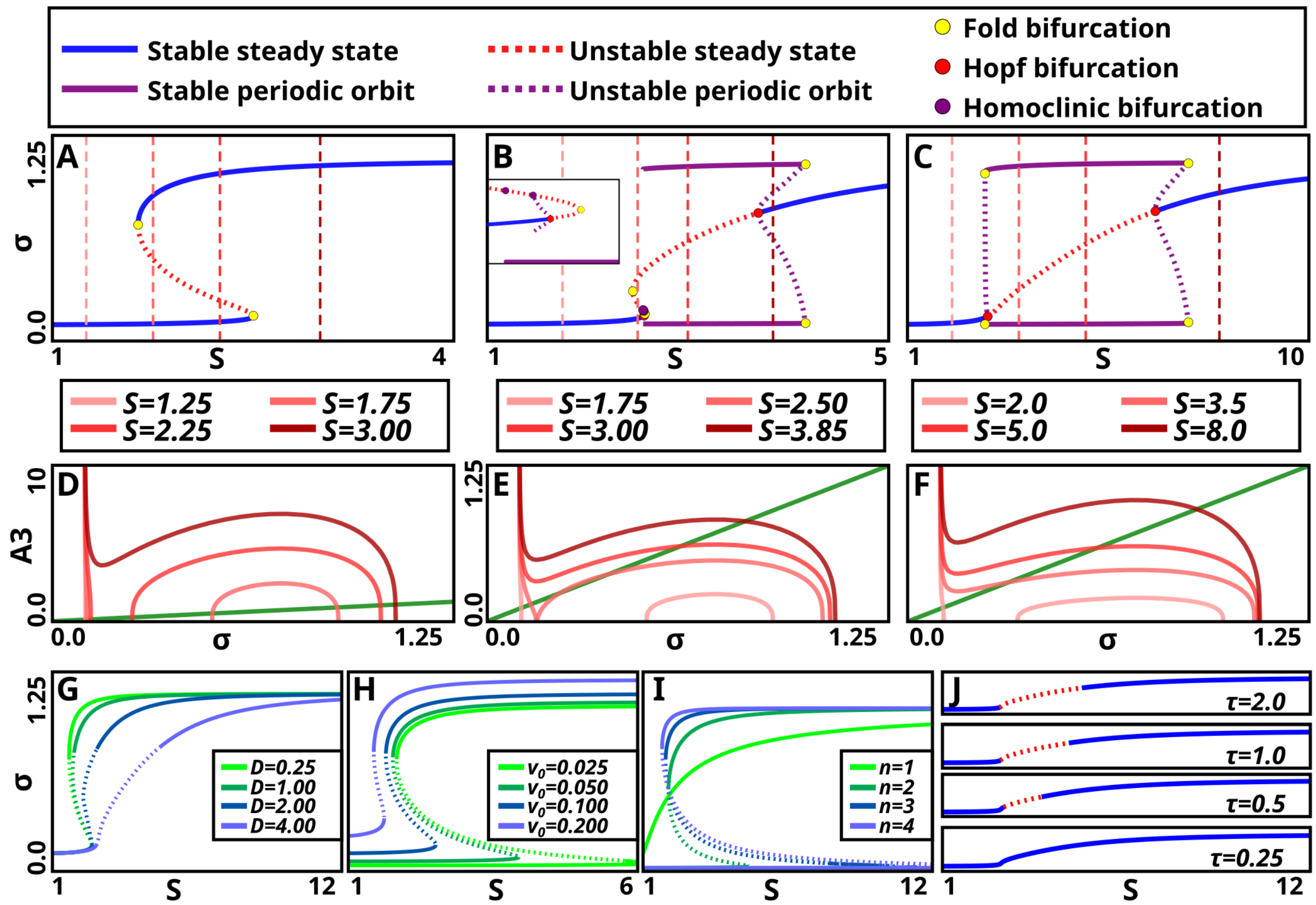

Supplementary Figure 14: **The model's bifurcation diagrams with respect to the parameter *S*.** (A-F) For three different model instances, the system's bifurcation diagram is plotted (top), and the nullclines of the same system plotted in phase space (bottom). The $[\dot{\sigma}] = 0$ nullcline is plotted for four different values of S (these values are also marked with dotted lines in the bifurcation diagram). (A,D) The system where it exhibits a bistable switch. (B,E) The system may also exhibit a region with two unstable steady states, and a single stable one. (C,F) Finally, an instance that does not exhibit bistability, and smoothly transition from a single inactive state to a single active state. (G-I) Bifurcation diagrams plotted for a selection of parameter values. Solid lines indicate stable steady states and dotted unstable ones. (G) As the parameter D is modulated, it exhibits the range of bifurcation diagrams in A-C. (H) Modulating the parameter v_0 affects the size of the bistability region. (I) Bifurcation diagrams for a few values of n, only for $n > 1$ does the system exhibit bistability. (J) As the parameter τ is increased, the size of the mono-unstable region increases. Source code used to generate this figure can be found in the provided GitLab repository.

Supplementary Note 7: Behaviour classification

7.1 Determining the model's possible response behaviours

To examine our model we determined the behaviours that it might exhibit. The behaviours were classified according to the system's possible transitions between its steady states. The system either contains a single unstable steady state (and thus oscillates), or one or two stable steady states (as shown in Supplementary Note 6.1). If the system is bistable, one (stable) steady state corresponds to an *inactive value*, and one to an *active value*. If it is monostable, the steady state corresponds to either an *inactive value*, an *active value*, or some *intermediate value*.

Given a system with at least a single steady state, the solution will asymptotically tend toward a steady state. However, fluctuations may push the system out of a steady state (either transitioning to another steady state, or, in an excitable behaviour, into a temporary pulse which eventually returns to the initial steady state). Two types of fluctuations may push the system out of a steady state. One type is stochastic noise. Alternatively, at the onset of the input (S changed from 0 to its designated value), the steady states of the system shift, causing the system to exist in a non-steady state. This constitutes a perturbation to the system. The remaining paragraphs of this section will describe how we classify a model instance according to the types of transitions between states it exhibits.

In the absence of the input ($S = 0$), the system has a single steady state at $([\sigma], [A_i]) = (v_0, v_0)$ (where it rests). At the input's onset, the system may shift into an active state (briefly ignoring the possibility of an intermediate state, which is handled separately at the end). This transition is driven by fluctuations both due to the input's onset, as well as noise. This time of activation we call the *initial activation time* (IAT). If the system reaches an active state, it may similarly transition back into an inactive state, this time we call the *deactivation time* (DT). Finally, if the system deactivates, it may again activate. The time to reactivate after having deactivated we call the *reactivation time* (RT, which is different from the initial activation time in that it is only driven by noise). Deactivation times that follow either a normal activation or an initial activation should be identically distributed (hence we do not need to split these into initial and normal deactivation times). Here, the initial activation is a transient behaviour, while deactivations and reactivations are asymptotic behaviours.

We classify the times for this state shift as either *instantaneous* (happens immediately upon input onset, activation, or deactivation), *heterogeneous* (the transition happens after some delay, and not necessarily immediately), and *infinite* (the transition never happens). The three types of transitions, to the power of the three classes of transitions, generate 27 different alternative patterns of transitions. However, several will not happen (e.g. a system with infinite activation time will not have a reactivation time). Also, some combinations will yield behaviours that we assign to the same class. The full classification can be found in Supplementary Figure 15.

This scheme does not work on a system with a single steady state of intermediate activity. For what remains of this section, we will describe how we classify these systems. If this state is unstable, we will classify it as an oscillation (using the observation that a well-behaved CRN with only unstable steady states adopts a limit cycle). If the steady state is stable, we will classify the system as an *intermediate activation* (where the system, at the input's onset, immediately and homogeneously, transitions into a stable state of intermediate activity). One could imagine separate behaviours using intermediate states (e.g. stochastic pulsing from an intermediate state). Most of these, however, would have little qualitative difference from behaviours already defined in Supplementary Figure 15 (e.g. stochastic switching between an inactive state and an intermediate state). We rigorously scanned for new behaviours depending on steady states of intermediate activity, and only found three potential such cases:

- A limited *heterogeneous intermediate activation*, where the system, at the onset of the input, heterogeneously transitioned to a stable steady state of intermediate activity. However, the activation times were short and bounded (and did not seem to depend on the system remaining in a quasi-stable steady state, as is the case for normal heterogeneous activation). The region in parameter space where this

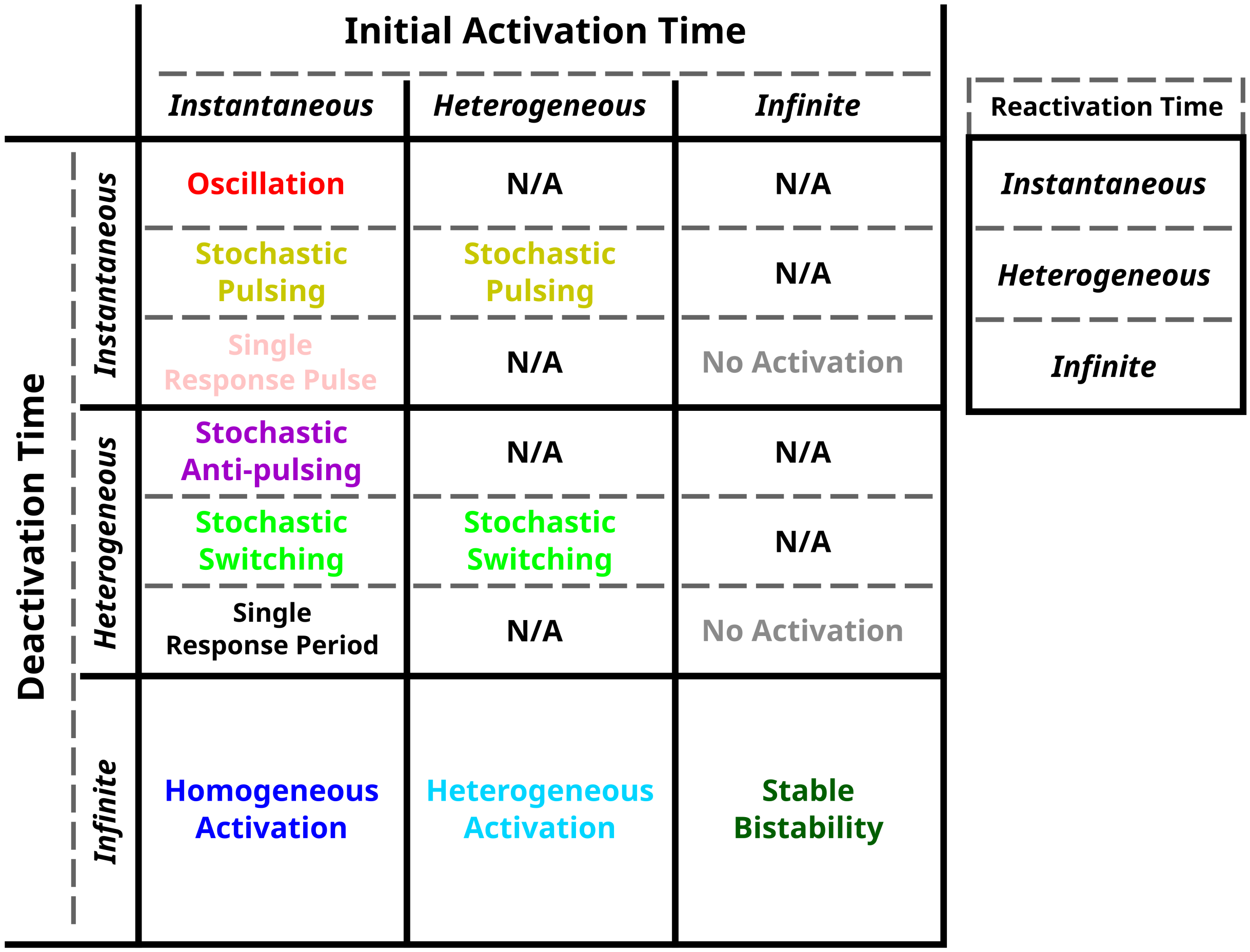

Supplementary Figure 15: **The model's potential behaviours are classified through its activation and deactivation times.** We define the model's *initial activation time* (IAT, time from input onset to it first reaching an active state), its *deactivation time* (DT, time from it reaching an active state to it reaching an inactive state) and its *reactivation time* (RT, time from it reaching an inactive state to it reaching an active state). Each set of times can either be classified as *instantaneous* (the transition is immediate), *heterogeneous* (the transition happens, but not necessarily instantaneously, over an ensemble the times are heterogeneous), and *infinite* (the transition never happens). The behaviours are located in a 3x3x3 grid, where the first dimension corresponds to the IAT type, the second to the DT type, and the third to the RT type. In the figure, the first two dimensions (IAT and DT) are shown in a 3x3 grid, with each field containing three subfields (corresponding to the third dimension, RT). The text in each subfield indicates what behaviour those combinations generate. A system with infinite DT cannot activate, hence no separate RT fields are given in these cases. Furthermore, if the IAT is infinite, we assume the RT is also infinite (the IAT is produced by perturbation due to input onset and noise, while the RT onset is only due to noise, if the former cannot yield a transition, neither should the latter). Using a similar argument, we assume that if the IAT is heterogeneous so is the RT. This produces a couple of impossible combinations (e.g. IAT infinite, DT instantaneous, and RT heterogeneous), which are marked as N/A. Next, the stochastic switching and stochastic pulsing behaviours can be subdivided depending on whether the IAT is instantaneous or heterogeneous, however, both these cases are assigned the same class (hence these two classifications each occur twice in the grid). Finally, the IAT instantaneous, DT heterogeneous, and RT infinite combination would yield a behaviour we call *single response period*. However, we have never encountered this behaviour, which is purely theoretical.

behaviour occurred was also small, and adjacent to those of normal intermediate activation. Hence this sub-behaviour was classified as an intermediate activation.

- In some cases, the system exhibited a response pulse, followed by relaxation into an intermediate state. However, due to the higher levels of $[\sigma]$ in the steady state, the response pulse would be less distinct. This was classified as an intermediate activation.
- For some bistable systems, the active state was narrowly in the region of intermediate activation. Otherwise, the system behaved like a bistable system. Again, this happened in a very narrow parameter region (adjacent to that where normal bistability occurred). In these cases, the system was considered as a bistable system with an inactive and an active stable steady state.

We recognise that more granularity could be added to the scheme, further subdividing the classes. This could include re-defining stochastic pulsing depending on whether there is an initial pulse in response to the input's onset (or if they only occur asymptotically). However, we decided further subdivisions would not be beneficial. In Supplementary Note 7.4, we list potential subdivisions of the 10 behaviours defined in the section.

7.2 Defining active and inactive states in the model

In Supplementary Note 7.3, we use the local minimum and maximum of the nullclines to define whether a system is in an active or in an inactive state (Supplementary Figure 16). These can be found as the values of $[\sigma]$ where $NC_\sigma([\sigma])'_{[\sigma]} = 0$. Again, while they cannot be found analytically, we can compute a polynomial with roots that both can reliably be found numerically and which corresponds to the nullcline's local minimum and maximum. First, we note that:

$$\frac{d}{dx} p \cdot f(x)^{1/n} = \frac{p}{n} f(x)^{1/n-1} \cdot f'(x)$$

since we are only interested in values where $NC_\sigma([\sigma]) \geq 0$ we only need to consider the inside function. We thus solve

$$\frac{d}{d[\sigma]}[(S[\sigma])^n(\frac{1}{[\sigma]-v_0} - 1) - 1] = 0$$

$$\Longleftrightarrow$$

$$n \cdot S^n \cdot [\sigma]^{n-1}(\frac{1}{[\sigma]-v_0} - 1) - S^n[\sigma]^n(\frac{1}{([\sigma]-v_0)^2}) = 0$$

$$\Longleftrightarrow$$

$$n(\frac{1}{[\sigma]-v_0} - 1) - [\sigma](\frac{1}{([\sigma]-v_0)^2}) = 0$$

$$\Longleftrightarrow$$

$$-\frac{[\sigma]}{n} + [\sigma] - v_0 - ([\sigma]-v_0)^s = 0$$

$$\Longleftrightarrow$$

$$[\sigma]^2 + (2v_0 + 1 - \frac{1}{n})[\sigma] + v_0^2 + v_0 = 0$$

which is our desired polynomial. Its roots can be computed as:

$$[\sigma]_0^{\pm} = \frac{1}{2n} - \frac{1}{2} - v_0 \pm \sqrt{(\frac{1}{2n} = \frac{1}{2} - v_0)^2 - v_0^2 - v_0}$$

$$\Longleftrightarrow$$

$$[\sigma]_0^{\pm} = \frac{1}{2n} - \frac{1}{2} - v_0 \pm \sqrt{\frac{1}{4n^2} + \frac{1}{4} - \frac{1}{2n} - \frac{v_0}{n}}$$

which either generates two roots for positive $[\sigma]$, or, when v_0 exceeds a critical value v_0^*, complex roots (that is, no local minimum and maximum exists).

As $v_0 \longrightarrow v_0^*$ the two roots approach each other. As they do, the region where the system has an intermediate state diminishes (this region will be defined as $[\sigma]_0^- < [\sigma] < [\sigma]_0^+$). For $v_0 > v_0^*$ we will still want to define active and inactive states, but will not be able to do so through the nullclines local minimum and maximum (since these no longer exist). For $v_0 > v_0^*$, we define the activity threshold as the critical value of the two roots as they absorb each other at v_0^* ($[\sigma]_0^* = [\sigma]_0^+(v_0^*) = [\sigma]_0^-(v_0^*)$. First, we note that the v_0^* can be found as a function of n only, by solving:

$$\frac{1}{4n^2} + \frac{1}{4} - \frac{1}{2n} - \frac{v_0}{n} = 0$$

which gives:

$$v_0^* = \frac{n}{4} - \frac{1}{2} + \frac{1}{4n}$$

and finally, we find $[\sigma]_0^*$ as the value of the roots where the square root is 0:

$$[\sigma]_0^* = \frac{1}{2n} - \frac{1}{2} - v_0$$

When classifying the behaviours of our model, we will need to define whether a state is to be considered *active*, *inactive*, or *intermediately active*. To do so, we use a scheme based on the local minimum and maximum of the system's nullcline (more specifically, the $[\dot{\sigma}] = 0$ nullcline, also called NC_σ). Here, states with a value of $[\sigma] < T_{ia}$ (with T_{ia} being the local minimum of the nullcline) are inactive. States with a value of $[\sigma] > T_a$ (with T_a being the local maximum of the nullcline) are active. Remaining states (with $T_{ia} \geq [\sigma] \leq T_a$) are intermediately active. The scheme is described in detail in Supplementary Figure 16.

7.3 Automated classification of the model's response behaviour

To enable us to classify the behaviour of the system at a given parameter set, we designed a classification algorithm. An initial algorithm was based on finding the steady states of the RRE interpretation of the system. While this algorithm yielded results similar to the final one, due to noise's tendency to shift a system's fixed points the algorithm had to be modified [13]. Over the next few paragraphs, as well as in Supplementary Figure 17, we present a heuristic algorithm that was created by modifying the initial one. Since the efficiency of the algorithm is not necessarily confirmed by its description, we demonstrate its performance through showing trial classifications. We generated a large number of parameter sets, and classified them. That the classifier generates appropriate classifications for this random sample can be seen in Supplementary Figures 18 and 19.

In the first step of our heuristic algorithm, the steady states of the parameter set are classified. First, the steady states of the deterministic RRE interpretation of the model are determined. If the system is bistable it is classified as "bistable". Else, the median σ concentration of the system (measured over several long simulations, $11,000$ time units) is found. The system is then classified as "inactive", "active", or "intermediate" (corresponding according to what property the median value has according to the scheme in Supplementary Figure 16).

Next, the steady state(s) are computed (as the deterministic steady states in the bistable case, by using the median values of each component of the system in the single steady state cases). From these, the system's reactivation time (RT, inactive and bistable systems only) and deactivation time (DT, active and bistable systems only) are computed. This is done by starting a number of simulations ($N = 40$) in the inactive (for RT) or active (for DT) and measuring the mean times it takes the system to pass the threshold (T_a for RT and T_{ia} for DT). If the mean passage time is lower than an (arbitrarily selected) threshold (of

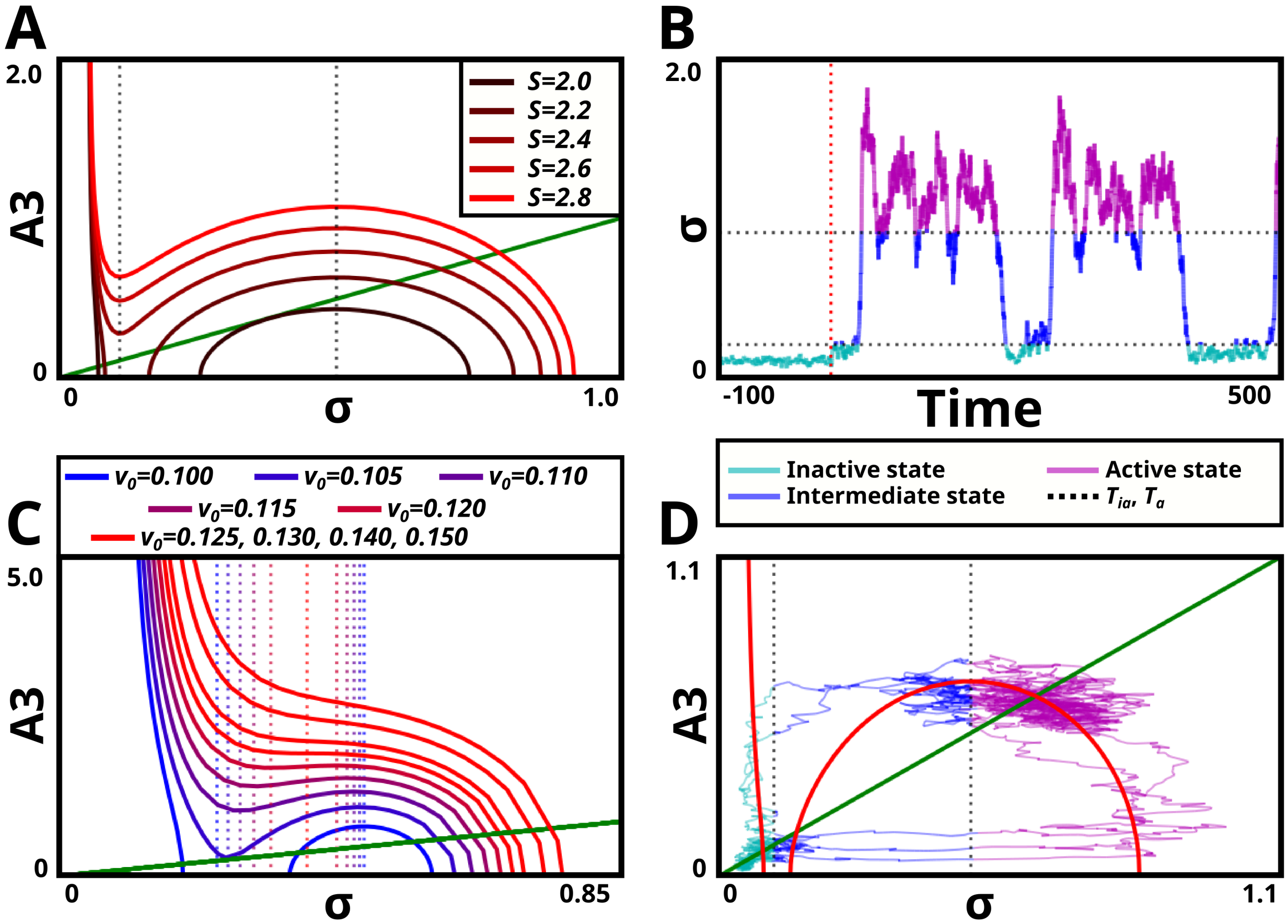

Supplementary Figure 16: **We use the nullclines to define what states of the model are active and which are inactive.** (A) The nullcline NC_σ (for $[\dot{\sigma}] = 0$) may have a local minimum and maximum (their $[\sigma]$ values are called T_{ia} and T_a). T_{ia} and T_a are independent of the values of S (and D, τ, and η). (B,D) A single simulation of the model, with T_{ia} and T_a marked in black dotted line. The trajectories are coloured according to whether the current state is inactive (teal), intermediate active (blue), or active (purple). (B) The simulation shown in time. (D) The simulation shown in phase space (the nullclines plotted in red and green). (C) As the value of v_0 increases, the thresholds T_{ia} and T_a approach each other. Here, for a range of values of v_0, the nullcline (solid line) and the corresponding thresholds (dotted lines) are plotted. For large values of v_0 ($v_0 > v_0^*$), neither local minimum nor maximum exists (bright red lines). Here, both T_{ia} and T_a are set to the boundary threshold obtained at the annihilation point. Source code used to generate this figure can be found in the provided GitLab repository.

25 time units), they are classified as *instantaneous*. If a certain percentage of the simulations (arbitrarily selected value of 85%) had not passed the threshold at the end of the simulation (length $2,000$ time units), the times are classified as *infinite*. Else, they are classified as *heterogeneous*. A fourth case, with passage times being very similar, but with a mean higher than the instantaneous activation threshold can be considered (*delayed homogeneous passage*). However, this happened so rarely that its occurrence is most likely due to random chance. Hence we do not include this case.

Finally, for some cases, the IAT were measured and classified. These were measured by starting a number of simulations ($N = 40$) in the state corresponding to system with no input ($(\sigma, A_i) = (v_0, v_0)$) and measuring the mean time for the simulations to pass the threshold to an active state (T_a). If the mean was lower than a threshold (25 time units) the IAT were classified as instantaneous. Else they were classified as *heterogeneous or infinite*. In this case, the additional effect of the transient effect due to the change

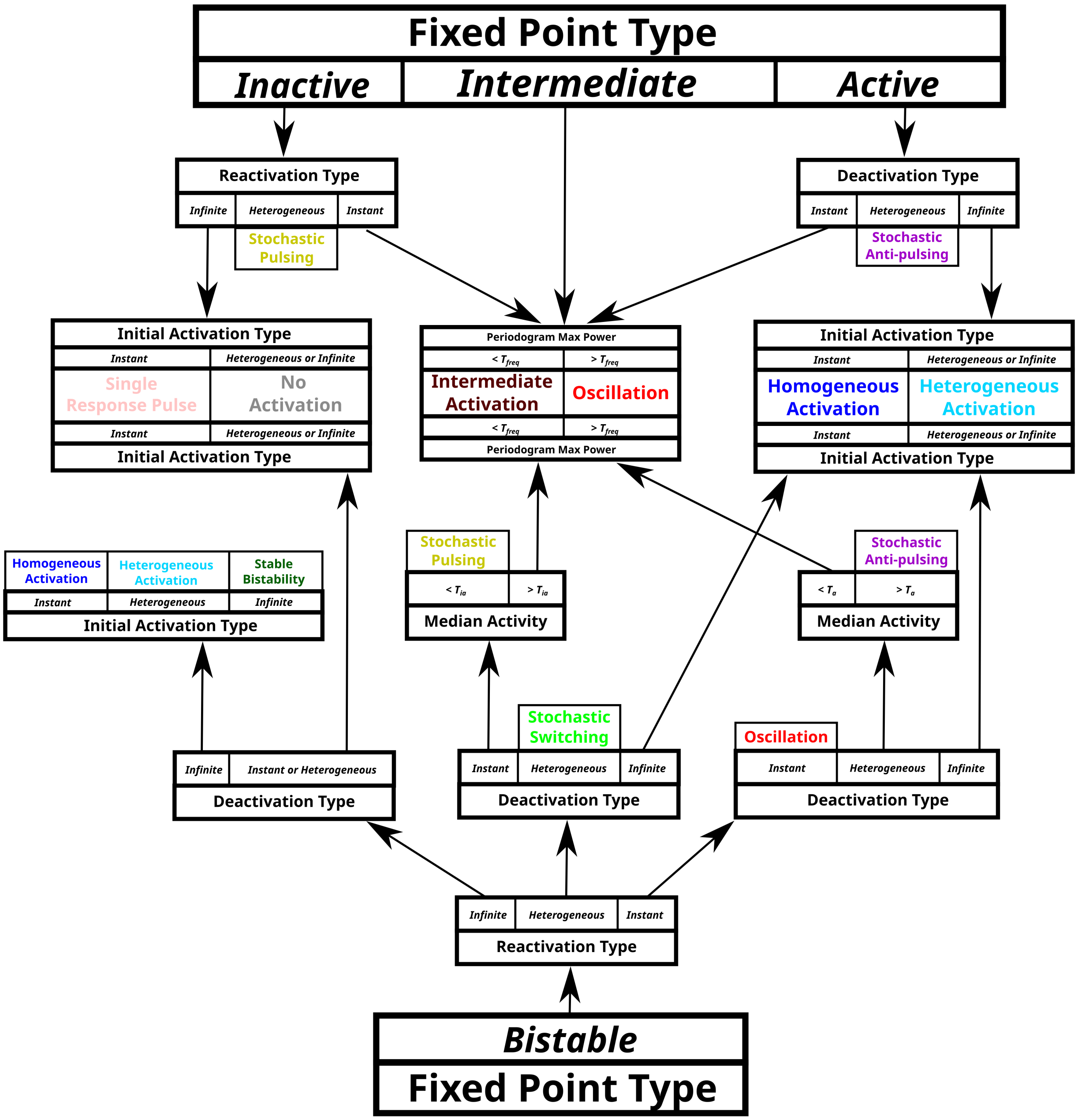

Supplementary Figure 17: **A decision tree heuristic is used to classify the response behaviour of a model instance.** The system's fixed point type (inactive, intermediate, active, or bistable) is first measured, then in succession its: reactivation type, deactivation type, and initial activation type (each either classified as infinite, heterogeneous, or instantaneous). Boxes with coloured texts indicate a classification result. In some cases, it is checked whether the system's median activity surpasses some threshold (T_{ia} being the nullcline's local minimum and T_a its local maximum, Supplementary Note 7.2). Similarly, it is checked whether the maximum value of the system's Welch type periodogram exceeds some threshold value ($T_{freq} = 10$).

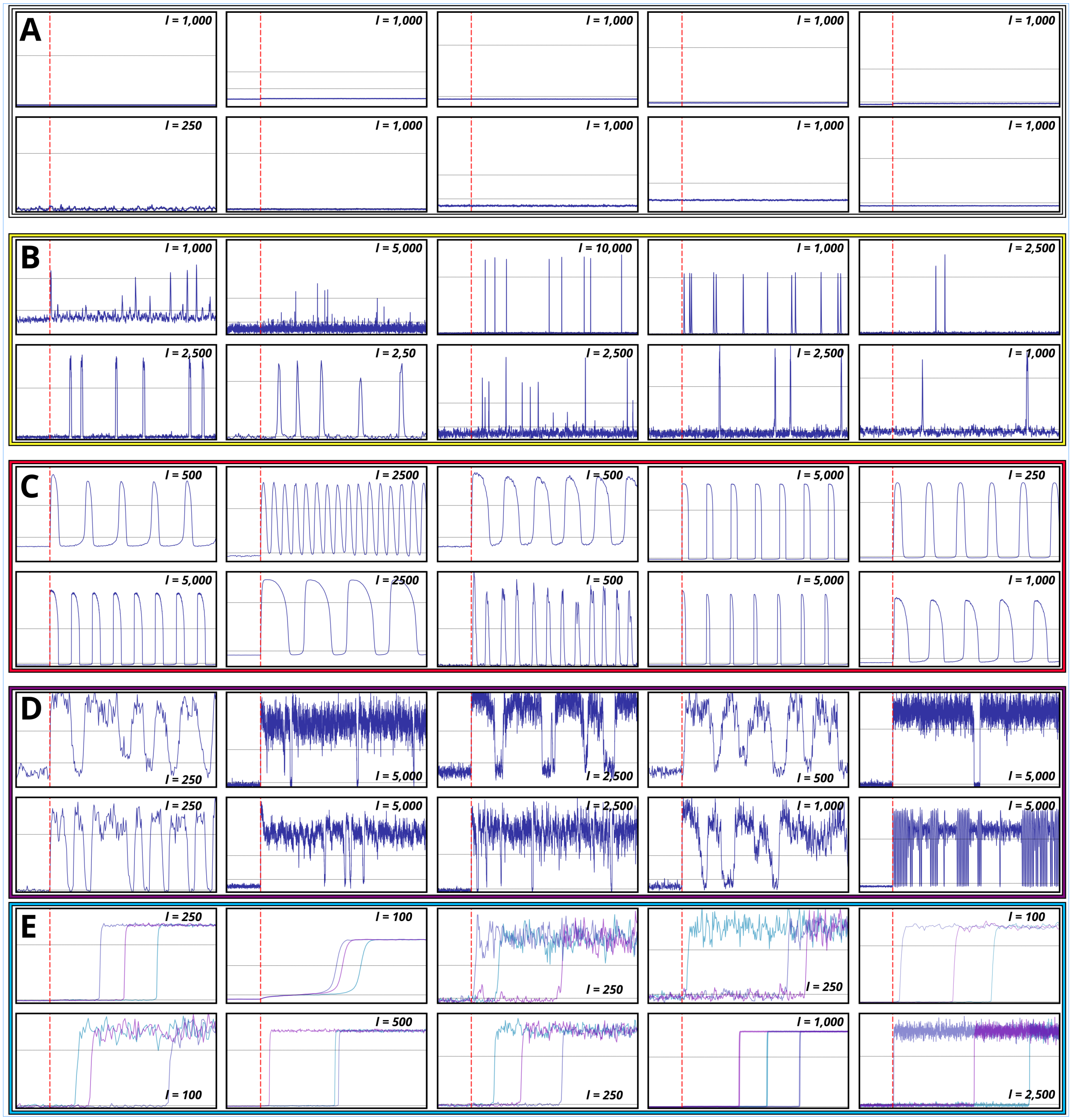

Supplementary Figure 18: **Example model simulations and the algorithm's corresponding classification (part 1).** Random parameter sets were generated and classified according to our algorithm. (A-E) Example trajectories from the parameter sets ($\sigma(t)$ in blue, the dotted black lines indicate T_{ia} and T_a, and dotted red lines the activation of the input). All y-axes are bound by $(0.0, 1.25)$. All x-axes are bound by $(-l/5, l)$, where l is marked in each plot (stress is added at time 0). (A) Sample simulated trajectories that were classified as the "no activation" behaviour. (B) Sample simulated trajectories that were classified as the "stochastic pulsing" behaviour. (C) Sample simulated trajectories that were classified as the "oscillation" behaviour. (D) Sample simulated trajectories that were classified as the "stochastic anti-pulsing" behaviour. (E) Sample simulated trajectories that were classified as the "heterogeneous activation" behaviour. Source code used to generate this figure can be found in the provided GitLab repository.

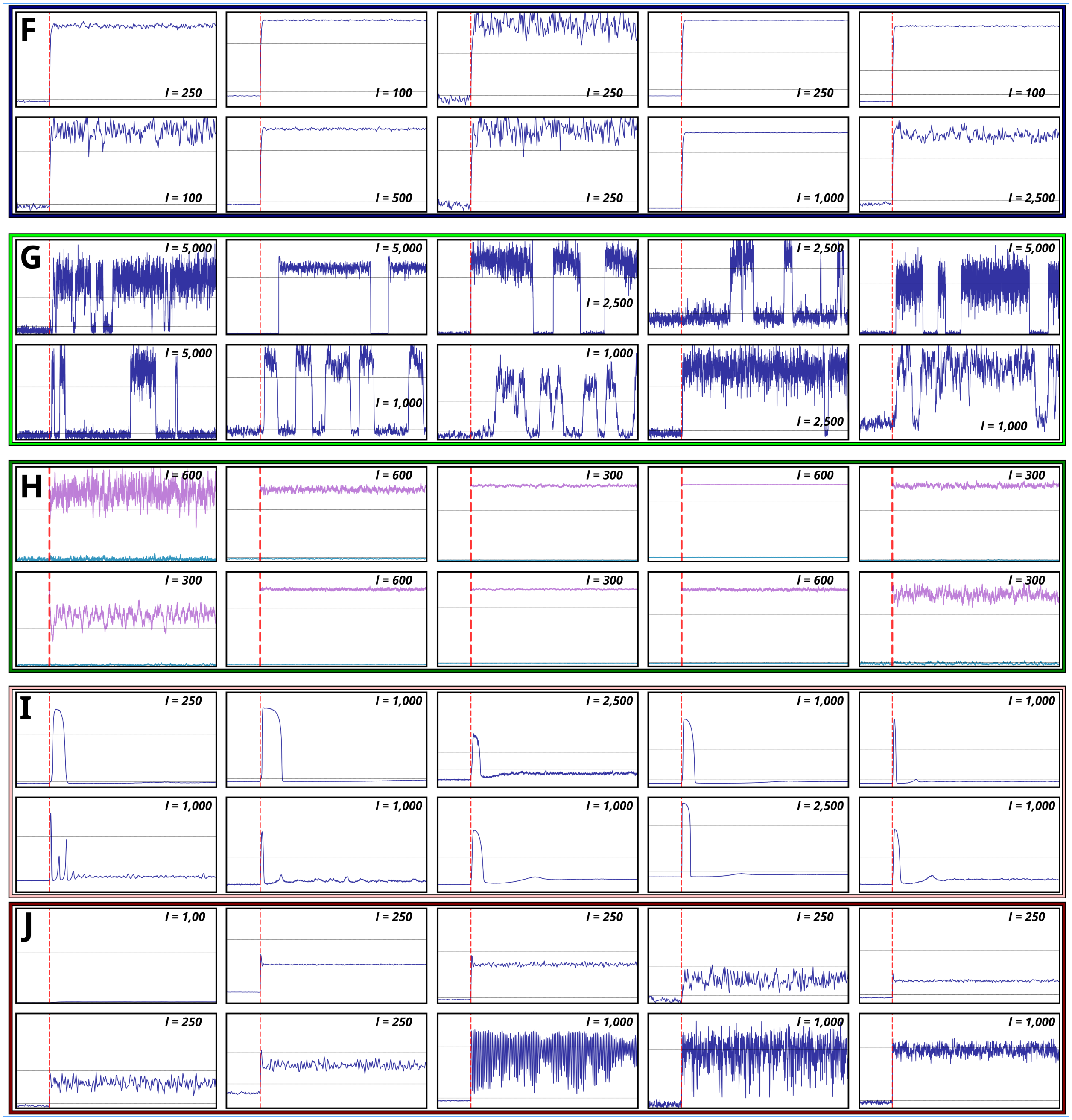

Supplementary Figure 19: **Example model simulations and the algorithm's corresponding classification (part 2).** Random parameter sets were generated and classified according to our algorithm. (F-J) Example trajectories from the parameter sets ($\sigma(t)$ in blue, the dotted black lines indicate T_{ia} and T_a, and dotted red lines the activation of the input). All y-axes are bound by $(0.0, 1.25)$. All x-axes are bound by $(-l/5, l)$, where l is marked in each plot (stress is added at time 0). (F) Sample simulated trajectories that were classified as the "homogeneous activation" behaviour. (G) Sample simulated trajectories that were classified as the "stochastic switching" behaviour. (H) Sample simulated trajectories that were classified as the "stable bistability" behaviour. (I) Sample simulated trajectories that were classified as the "single response pulse" behaviour. (J) Sample simulated trajectories that were classified as the "intermediate activation" behaviour. Source code used to generate this figure can be found in the provided GitLab repository.

in parameter values would have worn off, and the passage times should be asymptotically identical to the reactivation times. Hence, in this case, the IAT distribution contributed no additional information of the system, and was not used.

Inactive systems with RT heterogeneous were classified as *stochastic pulsing*, while active systems with DT heterogeneous were classified as *stochastic anti-pulsing*. Inactive systems with RT infinite were classified according to their initial activation times (*single response pulse* if instantaneous, else *no activation*). Similarly, active systems were classified as *homogeneous activation* if the IAT were instantaneous, and else as *heterogeneous activation*.

Intermediate systems, inactive systems with instant activation times, and active systems with instant deactivation times were classified according to their frequency properties. The system was simulated for a long time ($11,000$ time units) and the Welch-type periodogram was computed using the last $10,000$ time units of the simulation (with the mean activity subtracted). It was then checked whether the maximum value of the periodogram passed a specific threshold (T_{freq}, arbitrarily set to $T_{freq} = 10$). If it did, the system was classified as an oscillation, else as an intermediate activation.

For a bistable system, RT was first classified. If it was infinite, then DT and IAT were found. For DT infinite, and IAT instant, the system was classified as homogeneous activation. For DT infinite, and IAT heterogeneous, the system was classified as *heterogeneous activation*. For DT infinite, and IAT infinite the system was classified as *stable bistability*. If DT was heterogeneous or infinite, the system was classified as a single response pulse if IAT was instantaneous, else it was classified as no activation.

If instead RT was heterogeneous, then DT was measured. If it also was heterogeneous, the system was classified as *stochastic switching*. If DT instead was instantaneous, the median activity of the system was measured (see the previous description). If the median activity was inactive, the system was classified as stochastic pulsing, else it was classified as either *oscillation* or *intermediate activation*, depending on its frequency properties (see the previous description). If DT was infinite, then IAT was measured. If IAT was instantaneous the system was classified as homogeneous activation, else as *heterogeneous activation*.

Finally, if RT was instantaneous, then DT was measured. If DT also was instantaneous the system was classified as oscillation. If it was heterogeneous the median activity of the system was measured (see the previous description). If the median activity was active, the system was classified as stochastic anti-pulsing, else it was classified as either oscillation or intermediate activation, depending on its frequency properties (see the previous description). Finally, if DT was infinite, then the IAT was measured. If IAT was instantaneous, the system was classified as homogeneous activation, else as heterogeneous activation.

7.4 List of behavioural subclasses

While we in this work present 10 different potential response behaviours, these can potentially be subdivided further. While preparing this work, we decided that the 10 behaviours described well represent the potential dynamics of the mixed positive/negative feedback loop. However, for completeness, we here present additional potential behaviours that the model can exhibit.

- **Heterogeneous intermediate activation**: In our classifier, we classify any behaviour which reaches (and stays in) a state of intermediate activity as intermediate activation. However, potentially, it is possible to subdivide this behaviour into *heterogeneous intermediate activation* and *homogeneous intermediate activation* (depending on whether the initial activation time is heterogeneous or homogeneous). We note that while the mean activation time of the normal heterogeneous activation behaviour can get arbitrarily large, the activation times of the heterogeneous intermediate activation behaviour is fairly bounded. Furthermore, this behaviour only occurs in a limited region in the $D \approx 1$ region.

- **Stable bistability with intermediate activation**: Like the stable bistability behaviour, but where one stable steady state occurs for intermediate activity (and the other is an inactive state). Under our scheme, we would classify this as stable bistability.

- **Stochastic switching with intermediate activation**: Like the stochastic switching behaviour, but where one stable steady state occurs for intermediate activity (and the other is an inactive state). Under our scheme, we would classify this as stochastic switching. Indeed, some parameter sets classified as stochastic switching are technically stochastic switching with intermediate activation.
- **Single response pulse with intermediate activation**: A behaviour where there is an initial pulse of activity which reaches the active state, but which then settles to an intermediate activation state. Under our scheme, this behaviour is classified as intermediate activation.
- **Stochastic switching with oscillation and no activation**: In this behaviour, the system randomly switches between an oscillation and an inactive state. This behaviour can be observed in the region of the bifurcation diagrams where the limit cycle coexists with a stable inactive state (Supplementary Figure 14). Generally, this behaviour occurs between the stochastic pulsing and oscillation regions. Under our scheme, this behaviour is classified as stochastic pulsing.
- **Stochastic switching with oscillation and activation**: In this behaviour, the system randomly switches between an oscillation and an active state. This behaviour can be observed in the region of the bifurcation diagrams where the limit cycle coexists with a stable active state (Supplementary Figure 14). Generally, this behaviour occurs between the oscillation and stochastic anti-pulsing regions. Under our scheme, this behaviour is classified as stochastic anti-pulsing.
- **Single response period**: While this behaviour can be derived from our classification grid (Supplementary Figure 15), it was in practice never observed in the model.
- **Heterogeneous deactivation**: The reverse of heterogeneous activation, and in practice we have classified this as no activation. This behaviour (occurring between stable bistability and no activation) has two steady states, however, the active one is not long-term stable. Here, an inactive system will remain inactive. Meanwhile, a system (which, through some external means, has been put) in an active state will deactivate after some random time.

Supplementary Note 8: Behaviour descriptions

This section gives a brief overview of the observed behaviours, as well as sample trajectories (Supplementary Figures 20-29).

8.1 No activation

The no activation behaviour is characterised by the system staying constantly inactive (Supplementary Figure 20). Given small values of S, or $D \gg S$, the system adopts the no activation behaviour. This is the default behaviour of any system where the input is absent ($S = 0$), or negligible ($S \approx 0$). Since this is a default behaviour, we do not further investigate how the parameters affect its prevalence.

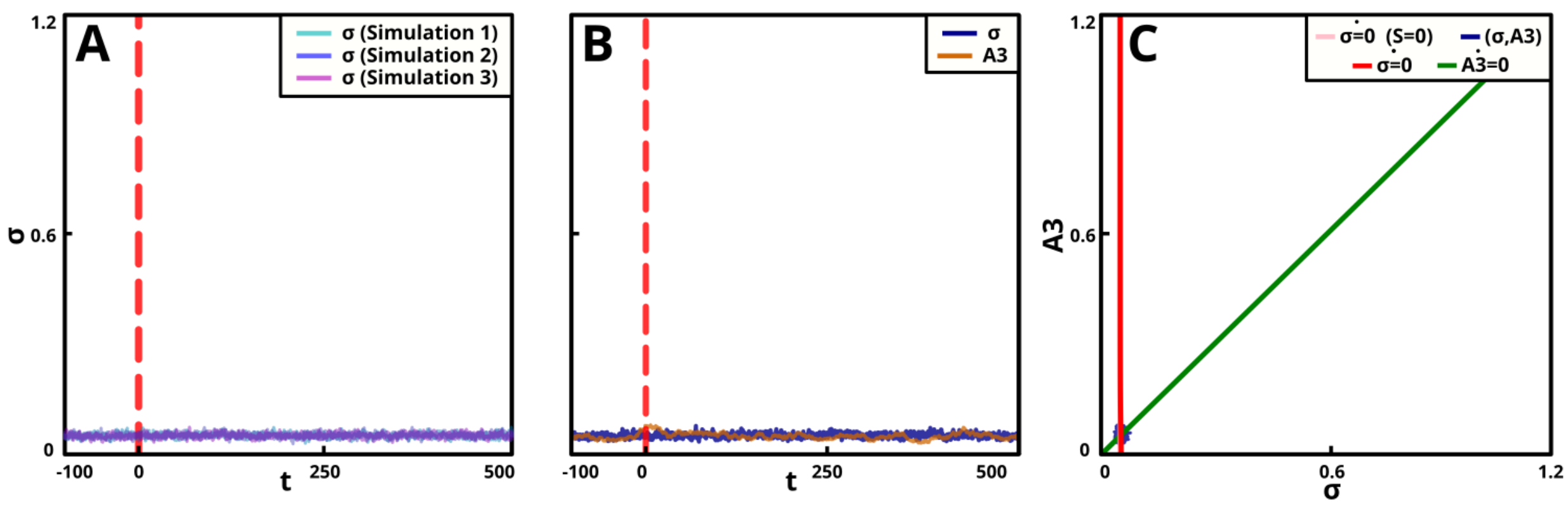

Supplementary Figure 20: **The no activation behaviour.** (A-C) Simulations of the no activation behaviour for the parameter set $(S, D, \tau, v_0, n, \eta) = (1.0, 0.1, 10.0, 0.1, 2, 0.1)$. (A) Three different simulations of the behaviour. (B-C) The same simulation, shown both over time and in phase space. (B) The concentration of the sigma factor (σ, blue line) and the (third) intermediary ($A = A_3$, yellow line) over time. (C) The state of the system in [σ]-[A] space (blue trajectory). The nullclines: NC_A (green), and NC_σ (pink for input absent, $S = 0$, and red for input present), are also displayed. Source code used to generate this figure can be found in the provided GitLab repository.

8.2 Stochastic pulsing

In the stochastic pulsing behaviour, the system contains only a single steady state, which corresponds to an inactive state. However, random fluctuations may push the system over a boundary that triggers a single pulse of activity. Since this is driven by noise, the pulses occur randomly in time (Supplementary Figure 21). This is a so-called excitable behaviour [14].

The stochastic pulsing behaviour occurs only in the rightmost ($D \gtrsim 1$) region of the S-D map. The stochastic pulsing behaviour requires S to be relatively small (so that the system is inactive in its single steady state). For large values of η, the behaviour can also occur in the ($D \lesssim 1$) region (Supplementary Figure 31). This is unlikely to be due to a genuine pulsing behaviour (i.e. noise amplified due to e.g. excitability), and rather be due to very high noise levels adding enough fluctuations to an otherwise inactive system so that it occasionally passes the threshold used to determine activity (indeed, at high noise, it becomes hard to distinguish individual behaviours as the system rather exhibits a "noisy mess"). Next, stochastic pulsing is strongly dependent on the parameters τ and η, increasing with their values. Conversely, stochastic pulsing cannot be maintained when either τ or η becomes small. Finally, there is also a slight trend of the behaviour becoming increasingly prevalent with the value of v_0 (Supplementary Figure 21D).

Depending on parameter values, the stochastic pulsing behaviour may or may not be accompanied by an

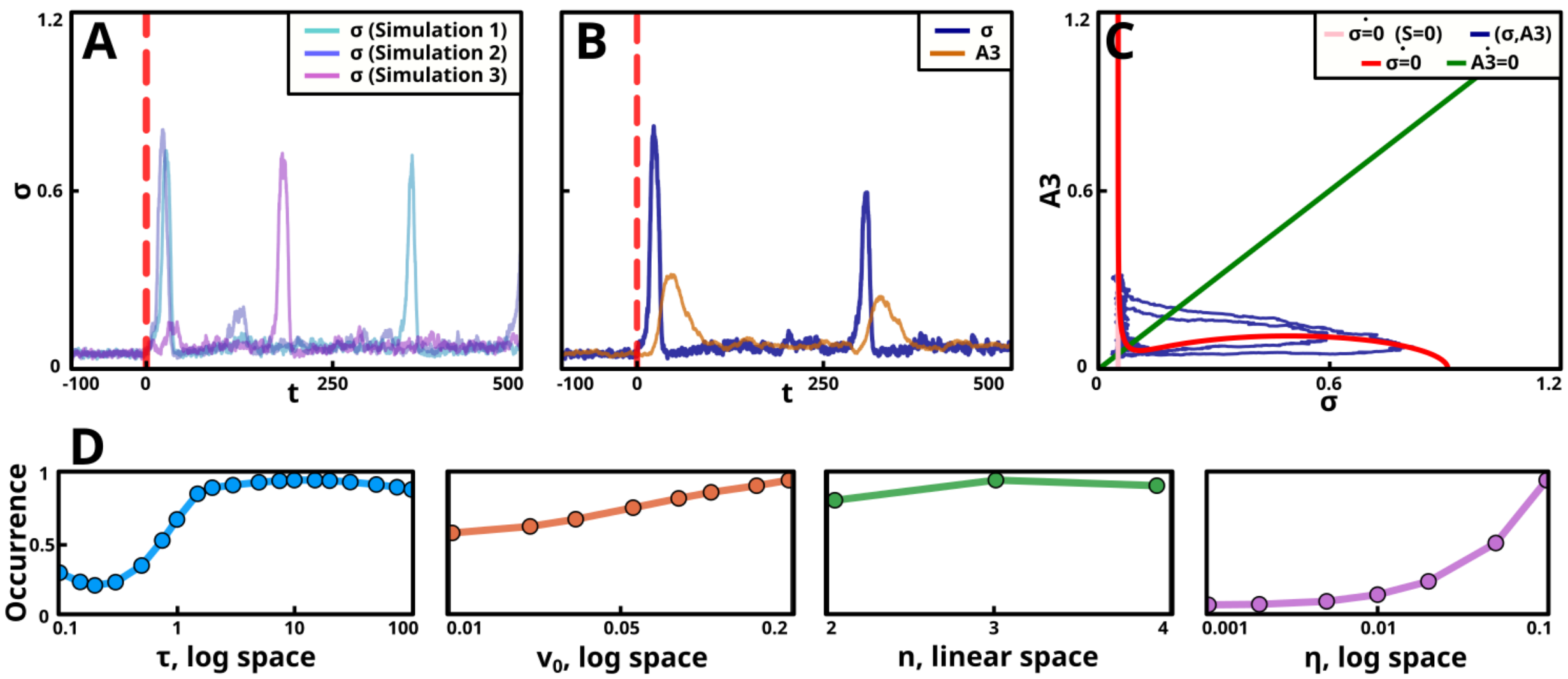

Supplementary Figure 21: **The stochastic pulsing behaviour.** (A-C) Simulations of the stochastic pulsing behaviour for the parameter set $(S, D, \tau, v_0, n, \eta) = (5.0, 50.0, 10.0, 0.05, 2, 0.05)$. (A) Three different simulations of the behaviour. (B-C) The same simulation, shown both over time and in phase space. (B) The concentration of the sigma factor (σ, blue line) and the (third) intermediary ($A = A_3$, yellow line) over time. (C) The state of the system in $[\sigma]$-$[A]$ space (blue trajectory). The nullclines: NC_A (green), and NC_σ (pink for input absent, $S = 0$, and red for input present), are also displayed. Note that, for this plot, the y-axis is for a different range than what is used in the other behaviour plots (to ensure that the trajectories can be distinguished clearly) (D) The occurrence analysis of the behaviour shows how its frequency depends on the parameters τ, v_0, n, and η (Supplementary Note 8.11)). Source code used to generate this figure can be found in the provided GitLab repository.

initial pulse of σ activity at the input's onset (like in the single response pulse behaviour). Typically this occurs for larger values of S, while for smaller values the system still exhibits asymptotic pulses, but not necessarily the transient activation pulse. We deemed these two behaviours not different enough to merit specific classifications, but we note the possible distinction.

Stochastic pulsing has been observed in a large number of sigma factors (σ^B, σ^D, σ^M, σ^W, and σ^X in *B. subtilis*, as well as RpoS in *E. coli*, [15, 16]). It has been suggested as a way to create population heterogeneity, possibly as a part of a bet-hedging behaviour [15]. Finally, it has been suggested that several sigma factors may each adopt a stochastic pulsing behaviour as a way of sharing a common resource (RNA Polymerase). Here, at each point in time, only a single sigma factor would be active, and thus utilising the resource [16].

8.3 Oscillations

In the oscillation behaviour, the system adopts a limit cycle where it oscillates around a single, unstable, steady state (Supplementary Figure 22). Potentially the behaviour could also be generated by the system regularly switching between an active and an inactive state. While this mechanism is accounted for in our classification scheme, we have not confirmed whether it actually generates oscillations in the model.

The oscillation behaviour occurs only in the rightmost ($D \gtrsim 1$) region of the S-D map. Here, oscillations require the region's single steady state to be unstable, something which typically occurs for intermediate levels of S (Supplementary Figure 31). The prevalence of the behaviour is mostly unaffected by the levels of v_0, n, and η. It does require a relatively large time-delay (large τ) and is absent for small values of τ. This makes sense since negative feedbacks (D non-negligible), when subject to time-delays (τ large enough), are

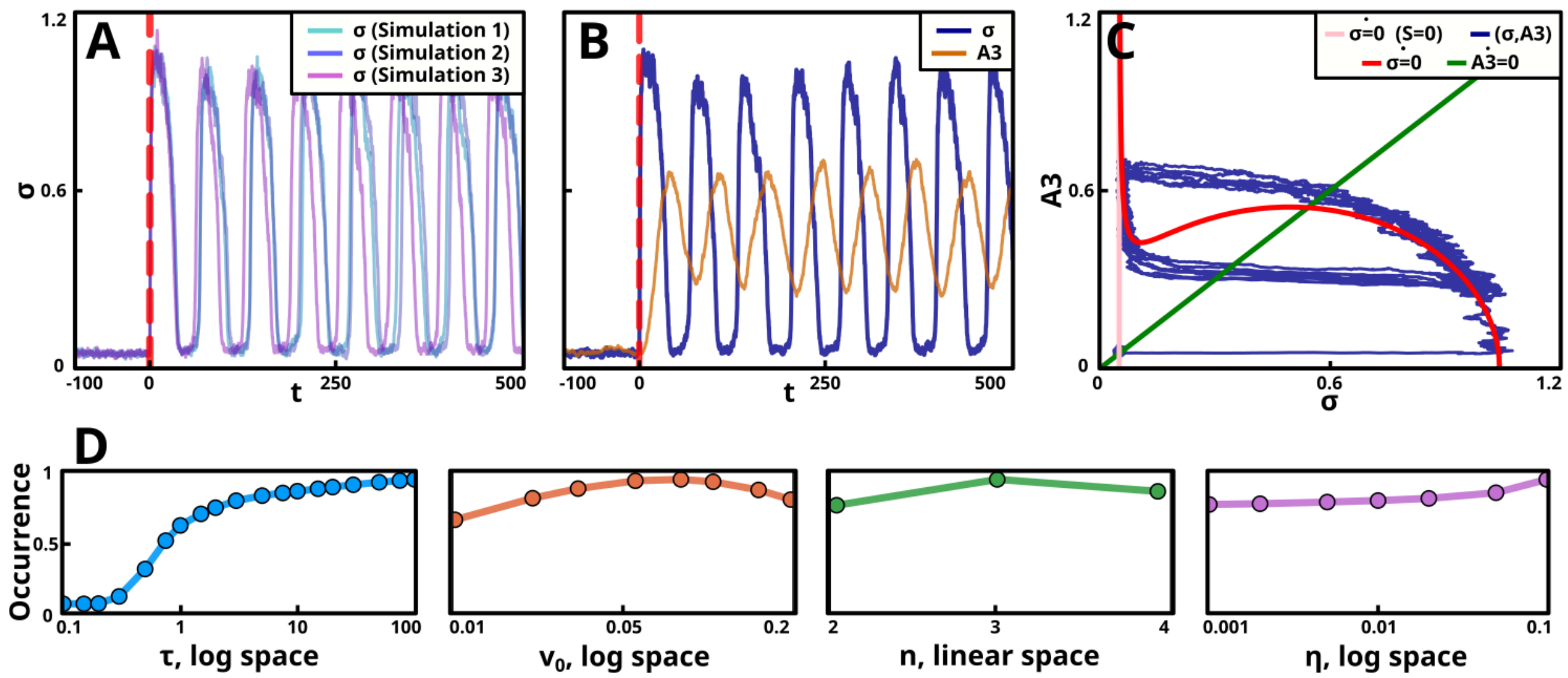

Supplementary Figure 22: **The oscillation behaviour.** (A-C) Simulations of the oscillation behaviour for the parameter set $(S, D, \tau, v_0, n, \eta) = (5.0, 5.0, 10.0, 0.05, 2, 0.05)$. (A) Three different simulations of the behaviour. (B-C) The same simulation, shown both over time and in phase space. (B) The concentration of the sigma factor (σ, blue line) and the (third) intermediary ($A = A_3$, yellow line) over time. (C) The state of the system in $[\sigma]$-$[A]$ space (blue trajectory). The nullclines: NC_A (green), and NC_σ (pink for input absent, $S = 0$, and red for input present), are also displayed. (D) The occurrence analysis of the behaviour shows how its frequency depends on the parameters τ, v_0, n, and η (Supplementary Note 8.11). Source code used to generate this figure can be found in the provided GitLab repository.

known to induce limit cycles (Supplementary Figure 22D).

Oscillations have been observed in a cell-free *E. coli* σ^{28} system (it is also observed in the σ^{B} mutants investigated in this article). In addition, it occurs in a large number of other cellular systems [17–19]. Most notably it is ubiquitous among various circadian rhythm systems [20]).

8.4 Stochastic anti-pulsing

In the stochastic anti-pulsing behaviour, the system contains only a single steady state, which corresponds to an active state. However, random fluctuations may push the system over a boundary, triggering a single (anti-)pulse of inactivity (Supplementary Figure 23). In many ways, it is the reverse of the stochastic pulsing behaviour.

Like the stochastic pulsing behaviour, stochastic anti-pulsing occurs in the rightmost ($D \gtrsim 1$) region of the S-D map. However, while stochastic pulsing occurs in the region between the no activity and oscillation behaviours, stochastic anti-pulsing occurs in the region between the oscillation and homogeneous activation behaviours. Like for stochastic pulsing, occurrences of stochastic anti-pulsing in the $D \lesssim 1$ region (when η is large) is likely an artefact (Supplementary Figure 31). Just like stochastic pulsing, stochastic anti-pulsing becomes increasingly prevalent with increasing values of τ and η (longer time-delay and more noise, respectively) and disappears when either value becomes small (Supplementary Figure 23D).

To our knowledge, stochastic anti-pulsing has not been observed in any experimental system (neither among sigma factors nor in the wider literature).

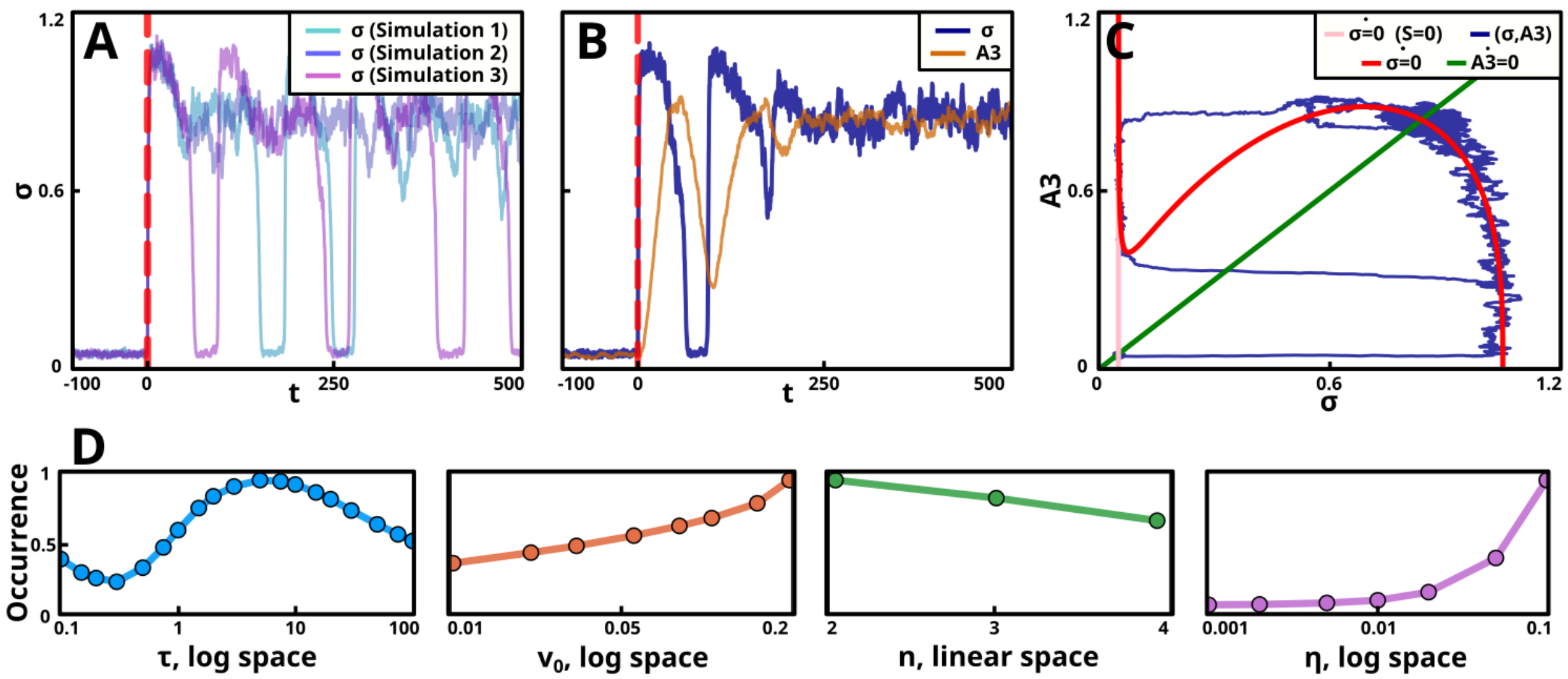

Supplementary Figure 23: **The stochastic anti-pulsing behaviour.** (A-C) Simulations of the stochastic anti-pulsing behaviour for the parameter set $(S, D, \tau, v_0, n, \eta) = (75.0, 50.0, 10.0, 0.05, 2, 0.05)$. (A) Three different simulations of the behaviour. (B-C) The same simulation, shown both over time and in phase space. (B) The concentration of the sigma factor (σ, blue line) and the (third) intermediary ($A = A_3$, yellow line) over time. (C) The state of the system in $[\sigma]$-$[A]$ space (blue trajectory). The nullclines: NC_A (green), and NC_σ (pink for input absent, $S = 0$, and red for input present), are also displayed. (D) The occurrence analysis of the behaviour shows how its frequency depends on the parameters τ, v_0, n, and η (Supplementary Note 8.11). Source code used to generate this figure can be found in the provided GitLab repository.

8.5 Homogeneous activation

In the homogeneous activation behaviour, at the time of input onset, the system switches from an inactive to an active state. The switch is immediate (and thus homogeneous across an ensemble of activations, as compared to the heterogeneous activation behaviour where it is not). The system is monostable, with only the active steady state (Supplementary Figure 24).

Given any combination of parameters, if S becomes sufficiently large, a homogeneous activation behaviour is always produced. Also, if S is higher than some threshold value, given a small enough value of D ($D \lesssim S$), a homogeneous activation behaviour will also be produced (Supplementary Figure 31). The occurrence analysis for this behaviour shows similar occurrence frequencies for all parameters. The variations we see are likely due to some parameters enlarging the regions of the other behaviours, reducing the counts for homogeneous activation, rather than the parameters affecting this behaviour directly. (Supplementary Figure 24D).

Theoretically, given a strong enough input, most sigma factors should activate homogeneously. However, the inherent properties of their circuits may put an upper limit to the degree to which the circuit can be activated (preventing this behaviour). The activation of many sigma factors is tied to stress. A strong enough stress will likely kill the bacteria (or cause other secondary effects which might prevent proper activation of the sigma factor), preventing high enough input magnitudes to generate homogeneous activation.

8.6 Heterogeneous activation

In the heterogeneous activation behaviour, at the time of input onset, the system switches from an inactive to an active state, but with highly heterogeneous response times. Typically, the system will have two stable steady states, one with low and one with high activity. Stochastic fluctuations may, however, push the system from the inactive state (where it is at the input's onset) across a separatrix and into the active state. Since this transition is dependent on noise, the activation time of the system is random (and thus

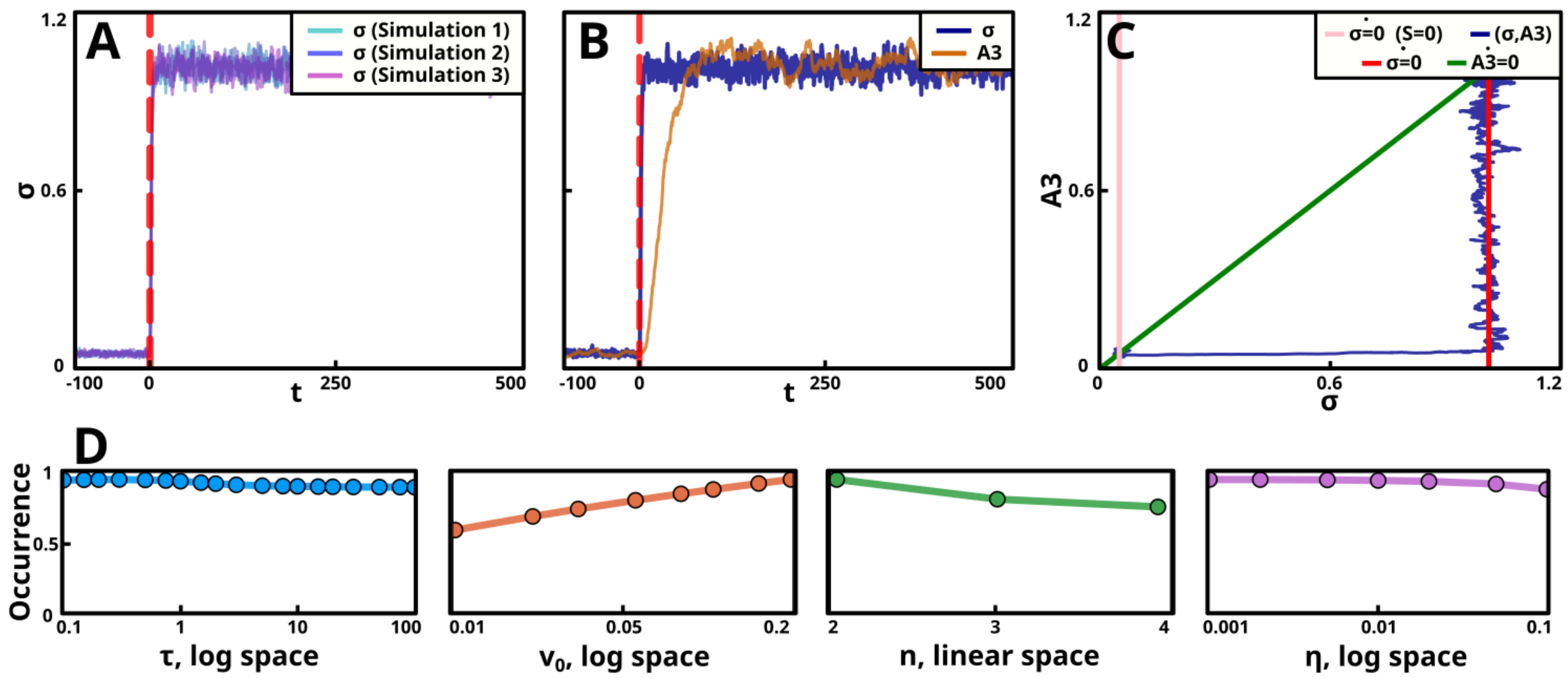

Supplementary Figure 24: **The homogeneous activation behaviour.** (A-C) Simulations of the homogeneous activation behaviour for the parameter set $(S, D, \tau, v_0, n, \eta) = (3.0, 0.1, 10.0, 0.05, 2, 0.05)$. (A) Three different simulations of the behaviour. (B-C) The same simulation, shown both over time and in phase space. (B) The concentration of the sigma factor (σ, blue line) and the (third) intermediary ($A = A_3$, yellow line) over time. (C) The state of the system in $([\sigma], [A]$ space (blueNC_σ trajectory). The nullclines: NC_A (green), and NC_σ (pink for input absent, $S = 0$, and red for input present), are also displayed. (D) The occurrence analysis of the behaviour shows how its frequency depends on the parameters τ, v_0, n, and η (Supplementary Note 8.11). Source code used to generate this figure can be found in the provided GitLab repository.

heterogeneous across a population). While the inactive state is only stable in the short term (due to noise eventually pushing the system out of it), the active state is long-term stable, with noise-driven transitions from it to the inactive state being impossible in non-absurd timeframes (Supplementary Figure 25).

Since the heterogeneous activation behaviour is dependent on bistability, it only occurs in the leftmost ($D \lesssim 1$) region of the S-D map, and only for an intermediate range of S values (the region where bistability occurs, Supplementary Figure 31). In addition, the prevalence of the behaviour increases with the value of n (increased ultrasensitivity is associated with more distinct bistability) and η (the system is clearly dependent on noise to make the inactive state long-term unstable). For large values of v_0 the behaviour is diminished (this is in agreement with Supplementary Figure 14, which suggests small v_0 promotes bistability). This also holds as η becomes large, likely due to high amplitude noise enabling transitions from the active to the inactive state (yielding a stochastic switching behaviour), or alternatively, makes the inactive state so unstable that activations become homogeneous (Supplementary Figure 25D).

Just like there exists bistable systems where the active state is long-term stable and the inactive one is not, there exist those with a long-term stable inactive state and an active state which is only short-term stable. Here, when the input parameter is changed from the value 0 to S, the system remains inactive. However, if the parameter is increased further (enough to activate the system), and then reduced back S again, one would see a heterogeneous deactivation system. We deemed this feature not interesting enough to distinguish it from the no activation behaviour.

The σ^V system of *B. subtilis* has been observed to activate heterogeneously in response to lysozyme stress [21]. In addition, the behaviour has been observed in non-sigma factor systems [22, 23].

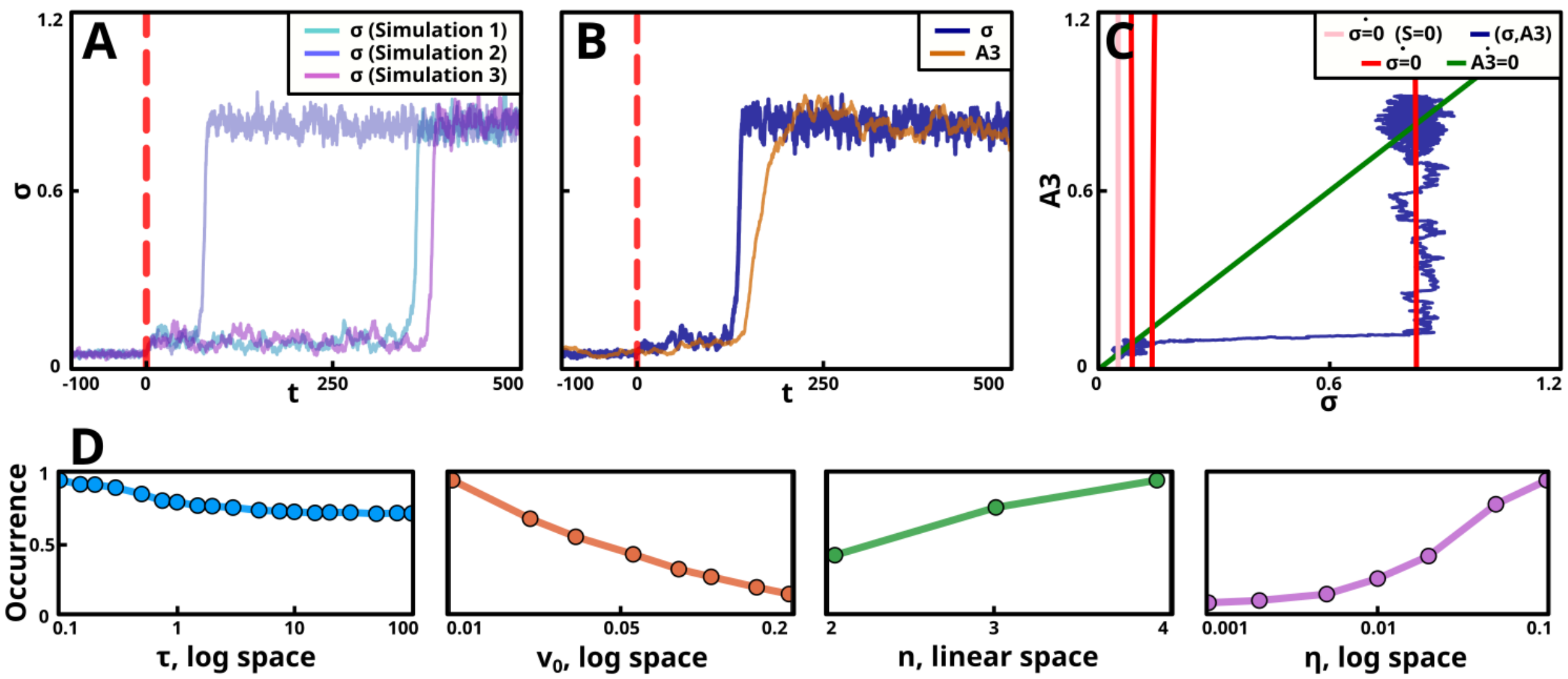

Supplementary Figure 25: **The heterogeneous activation behaviour.** (A-C) Simulations of the heterogeneous activation behaviour for the parameter set $(S, D, \tau, v_0, n, \eta) = (2.2, 0.1, 10.0, 0.05, 2, 0.05)$. (A) Three different simulations of the behaviour. (B-C) The same simulation, shown both over time and in phase space. (B) The concentration of the sigma factor (σ, blue line) and the (third) intermediary ($A = A_3$, yellow line) over time. (C) The state of the system in $[\sigma]$-$[A]$ space (blue trajectory). The nullclines: NC_A (green), and NC_σ (pink for input absent, $S = 0$, and red for input present), are also displayed. (D) The occurrence analysis of the behaviour shows how its frequency depends on the parameters τ, v_0, n, and η (Supplementary Note 8.11). Source code used to generate this figure can be found in the provided GitLab repository.

8.7 Stochastic switching

In stochastic switching, the system has two steady states, neither of which are long-term stable. Noise may drive the system from either steady state, across the separatrix, and into the other state. These switches occur randomly in time (Supplementary Figure 26).

The stochastic switching behaviour occurs in the leftmost ($D \lesssim 1$) region of the S-D map, typically for values of S lower than those that yield heterogeneous activation, but larger than what yields no activity. The behaviour also occurs in the centre of the plot, at $D \approx S \approx 1$ (Supplementary Figure 31). As a noise-driven behaviour, it requires a large enough value of η, and does not occur when η is small. It is also slightly less prevalent for small values of τ and large values of v_0 (Supplementary Figure 26D).

The stochastic switching behaviour has been observed in several cellular systems [24, 25], but not to our knowledge among bacterial sigma factors.

8.8 Stable bistability

In the stable bistability behaviour, the system has two steady states, both of which are long-term stable (no asymptotic switching between the two states occurs). At the activation of the input, the system will remain in its inactive state, and is in that sense no different from the no activation behaviour. However, some external factor could cause a switch to the active state (at which point it will remain there until further external perturbations cause a switch to the inactive state). Such a factor could be increasing S so that the system activates through a heterogeneous or homogeneous activation behaviour. When S is then reduced to the value yielding stable bistability, the system will be active instead of inactive. Alternatively, an external source of σ could be added, pushing the system to the active state (Supplementary Figure 27).

Like the previous bistability-based behaviours, stable bistability occurs in the leftmost ($D \lesssim 1$) region of the

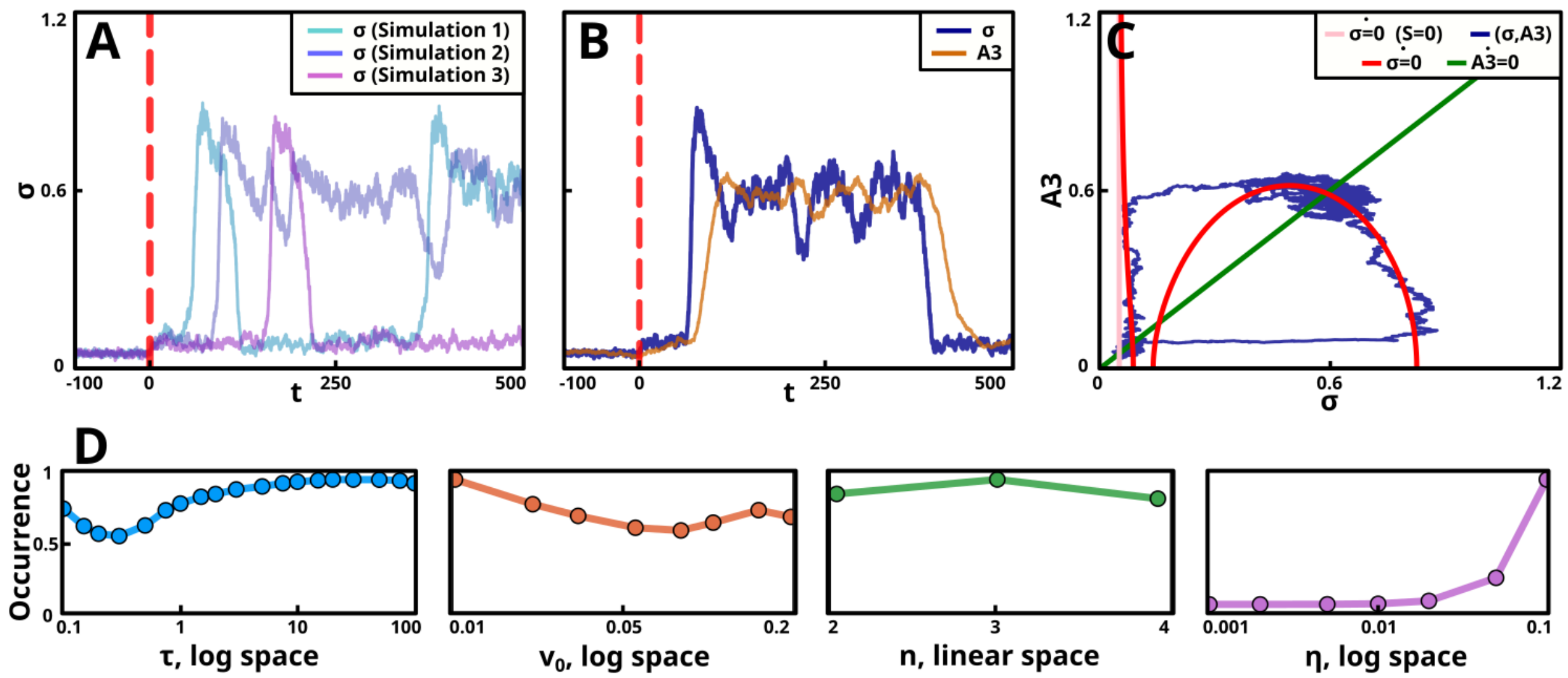

Supplementary Figure 26: **The stochastic switching behaviour.** (A-C) Simulations of the stochastic switching behaviour for the parameter set $(S, D, \tau, v_0, n, \eta) = (2.25, 1.1, 10.0, 0.05, 2, 0.05)$. (A) Three different simulations of the behaviour. (B-C) The same simulation, shown both over time and in phase space. (B) The concentration of the sigma factor (σ, blue line) and the (third) intermediary ($A = A_3$, yellow line) over time. (C) The state of the system in $[\sigma]$-$[A]$ space (blue trajectory). The nullclines: NC_A (green), and NC_σ (pink for input absent, $S = 0$, and red for input present), are also displayed. (D) The occurrence analysis of the behaviour shows how its frequency depends on the parameters τ, v_0, n, and η (Supplementary Note 8.11). Source code used to generate this figure can be found in the provided GitLab repository.

S-D map, and only for an intermediate range of S values (for higher values of S than no activity, but lower than heterogeneous activation, Supplementary Figure 31). Likely due to its dependency on bistability, the behaviour becomes more prevalent with increasing n. It is also dependent on having a low value for η (since increasing noise may create transitions between the two steady states). The behaviour also disappears for large values of v_0. Since it occurs for low values of D, where self-deactivation is weak, τ has little effect on its prevalence (Supplementary Figure 27D).

Stable bistability type behaviours are well known in development, where the selection of a system steady state may act as an irreversible decision of the developmental fate of a cell [26, 27]. To our knowledge, it has not been observed among bacterial sigma factors.

8.9 Single response pulse

In the single response pulse behaviour, the system is inactive as the input persists. However, the input's onset causes a large enough perturbation to the system that it displays a single pulse of activity (Supplementary Figure 28). This is an excitable behaviour [14]. Especially for low-noise regimes, this behaviour may be generated by a damped oscillation (Supplementary Figure 19).

Since the behaviour is dependent on excitability, it occurs in the rightmost ($D \gtrsim 1$) region of the S-D map. It requires small values of S (where the steady state is inactive). However, the values should be larger than what yield a no activation behaviour. We note that the single response pulse behaviour typically occupies a very slim range of S values. However, since the model is nondimensionalised, this region may correspond to a large region in a real system. As an excitable behaviour, it is reliant on a large enough time-delay (and thus disappears as τ becomes small). The behaviour also requires η to be small. Since the pulse is due to a deterministic phenomenon (input onset changing the steady state properties of the system) it is not dependent on noise. However, for larger η, stochasticity becomes prominent enough to cause the excitability

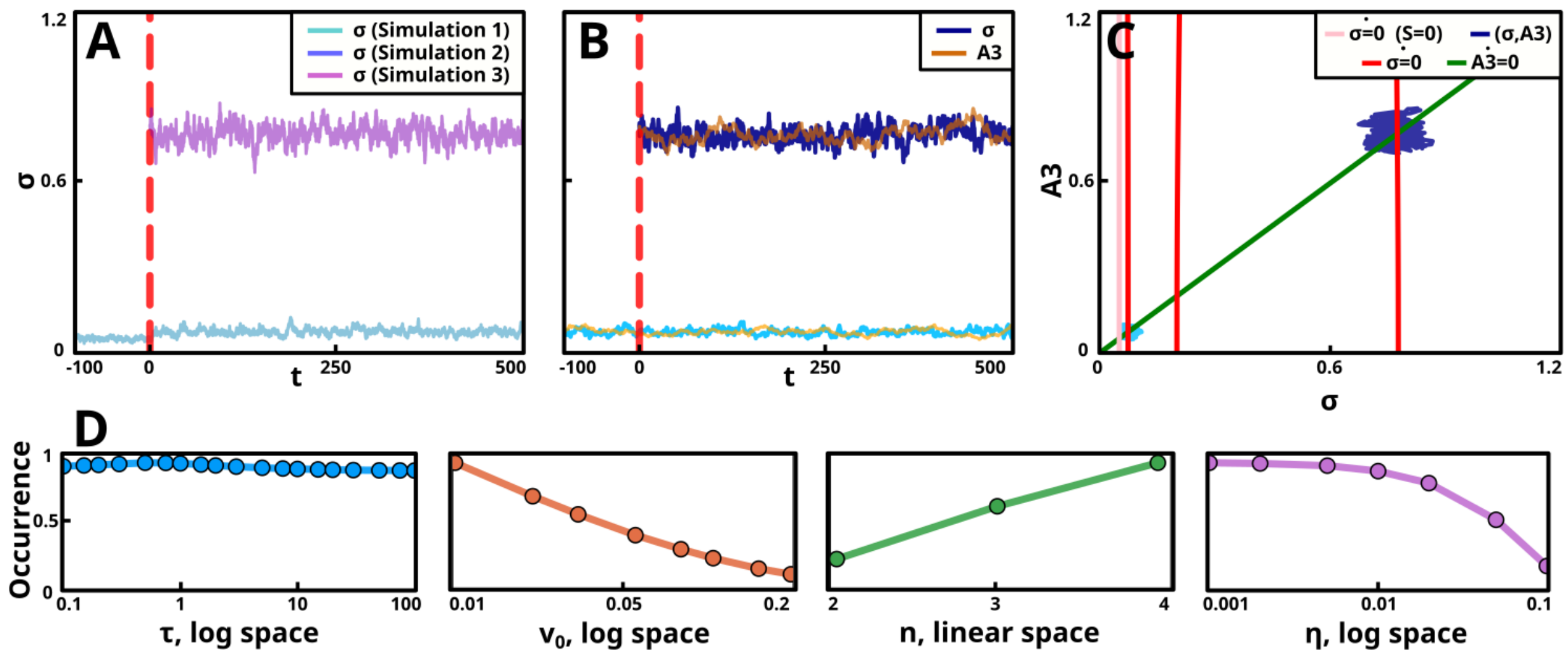

Supplementary Figure 27: **The stable bistability behaviour.** (A-C) Simulations of the stable bistability behaviour for the parameter set $(S, D, \tau, v_0, n, \eta) = (2.5, 0.1, 10.0, 0.025, 2, 0.05)$. (A) Three different simulations of the behaviour. Simulations 1 and 2 are performed in the inactive state, and simulation 3 in the active state (only present in the presence of the input). (B-C) Two sets of simulations, each one shown over time and in phase space. (B) Two simulations of the model, one in the system's inactive state and one in its active state. For each simulation, both the concentration of the sigma factor (σ, blue line) and the (third) intermediary ($A = A_3$, yellow line) are shown (lighter colours correspond to the inactive state simulation and darker colours to the active state simulation). (C) The same simulations as in B, with their trajectories shown in $[\sigma]$-$[A]$ space (blue trajectories). The nullclines: NC_A (green), and NC_σ (pink for input absent, $S = 0$, and red for input present), are also displayed. (D) The occurrence analysis of the behaviour shows how its frequency depends on the parameters τ, v_0, n, and η (Supplementary Note 8.11). Source code used to generate this figure can be found in the provided GitLab repository.

to be triggered by stochastic fluctuations as well, turning the behaviour into a stochastic pulsing one. The behaviour becomes more prevalent with larger values of v_0 and smaller values of n (Supplementary Figure 28D). The behaviour typically occurs in very slim regions of parameter space (Supplementary Figure 32), however, due to the nondimensionalised nature of the model, we cannot draw major conclusions for what this means for real systems.

The σ^B system in *B. subtilis* yields a single response pulse to the onset of environmental stress [28]. In addition, the antithetic feedback loop (a network motif which maintains the concentration of a regulated species at some stable level) can also generate single response pulses when its input changes [29]. However, while these motifs have similarities, they seem qualitatively different, as the antithetic feedback loop has not been related to stochastic pulsing.

8.10 Intermediate activation

In the intermediate activation behaviour, the system instantaneously activates in response to the input, but instead of reaching an active state (like in homogeneous activation), the system stops at some intermediate value (Supplementary Figure 29).

The intermediate activation behaviour occurs in the rightmost ($D \gtrsim 1$) region of the S-D map. It can be found for intermediate values of S, where the system's single steady state is neither inactive nor active (Supplementary Figure 31). The behaviour is also dependent on the value of τ. For large values of τ the steady state is unstable for intermediate values, yielding an oscillation. Only as τ becomes small does intermediate activation occur. The behaviour's prevalence has little dependency on the other parameter values. The

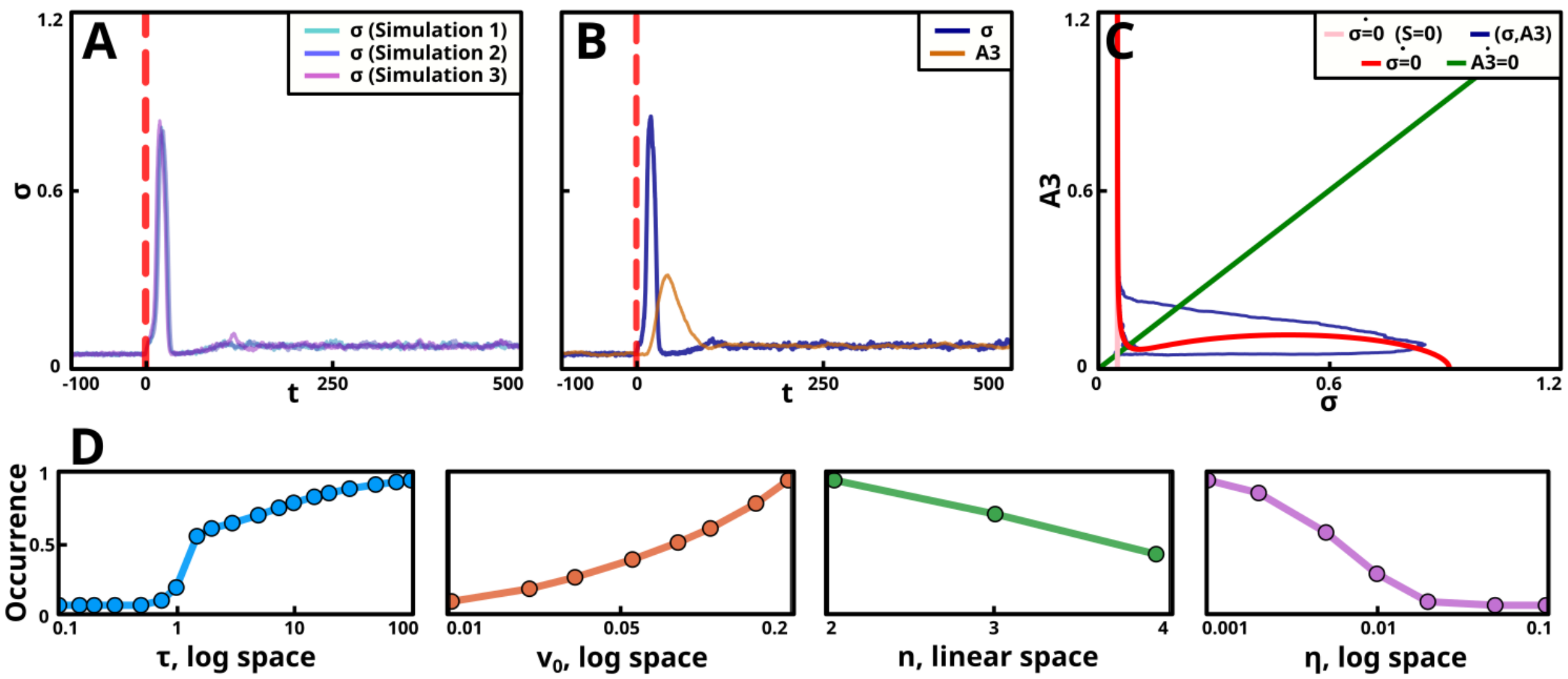

Supplementary Figure 28: **The single response pulse behaviour.** (A-C) Simulations of the single response pulse behaviour for the parameter set $(S, D, \tau, v_0, n, \eta) = (2.2, 6.0, 10.0, 0.1, 2, 0.025)$. (A) Three different simulations of the behaviour. (B-C) The same simulation, shown both over time and in phase space. (B) The concentration of the sigma factor (σ, blue line) and the (third) intermediary ($A = A_3$, yellow line) over time. (C) The state of the system in $[\sigma]$-$[A]$ space (blue trajectory). The nullclines: NC_A (green), and NC_σ (pink for input absent, $S = 0$, and red for input present), are also displayed. (D) The occurrence analysis of the behaviour shows how its frequency depends on the parameters τ, v_0, n, and η (Supplementary Note 8.11). Source code used to generate this figure can be found in the provided GitLab repository.

i

slight increase for large η is likely due to noise causing otherwise inactive states to spend long enough time in intermediate values to be deemed as intermediate activation (Supplementary Figure 29D).

For $D \approx 1$, one sometimes encounters a behaviour that can be considered heterogeneous intermediate activation, where the system reaches an intermediate steady state but with a random (non-instant) activation time. This behaviour only occurs for a slim parameter region (adjacent to the intermediate activation region) and reaches a state very close to being active. Also, unlike the normal heterogeneous activation behaviour, where the mean activation time can get arbitrarily large, the mean activation time for this behaviour seems bound to a relatively narrow range. Hence we decided this behaviour did not merit its own classification.

When considering single trajectories, intermediate activation is similar to homogeneous activation. However, they behave differently under tuning of S (and D). This can be seen in the bifurcation analysis (Supplementary Note 6.3). Here, intermediate activation appears in the same region as oscillation, but when τ is small enough that the system's single steady state is stable. Specifically, the mean activity level of the intermediate activation behaviour can always be tuned by modulating S (or D). Conversely, the homogeneous activation behaviour's mean activity level is highly robust to perturbations in S and D. The only exception is when these perturbations move the model into another, non-homogeneous activation, behavioural domain.

In practice, it may be hard to distinguish intermediate activation from homogeneous activation. This could likely only be done when one may vary S to see whenever the single steady state smoothly transitions from inactive to active, assuming intermediately active states in between (e.g. like what was done in Figure 6). Also, for larger values of η, the intermediate activation behaviour displays large fluctuations, from inactive to active states. This makes it hard to distinguish whether noisy experimental data is an intermediate activation behaviour, or simply large degrees of technical noise added upon some other behaviour. So while intermediate activation has likely been observed in both sigma factor and non-sigma factor systems, it is

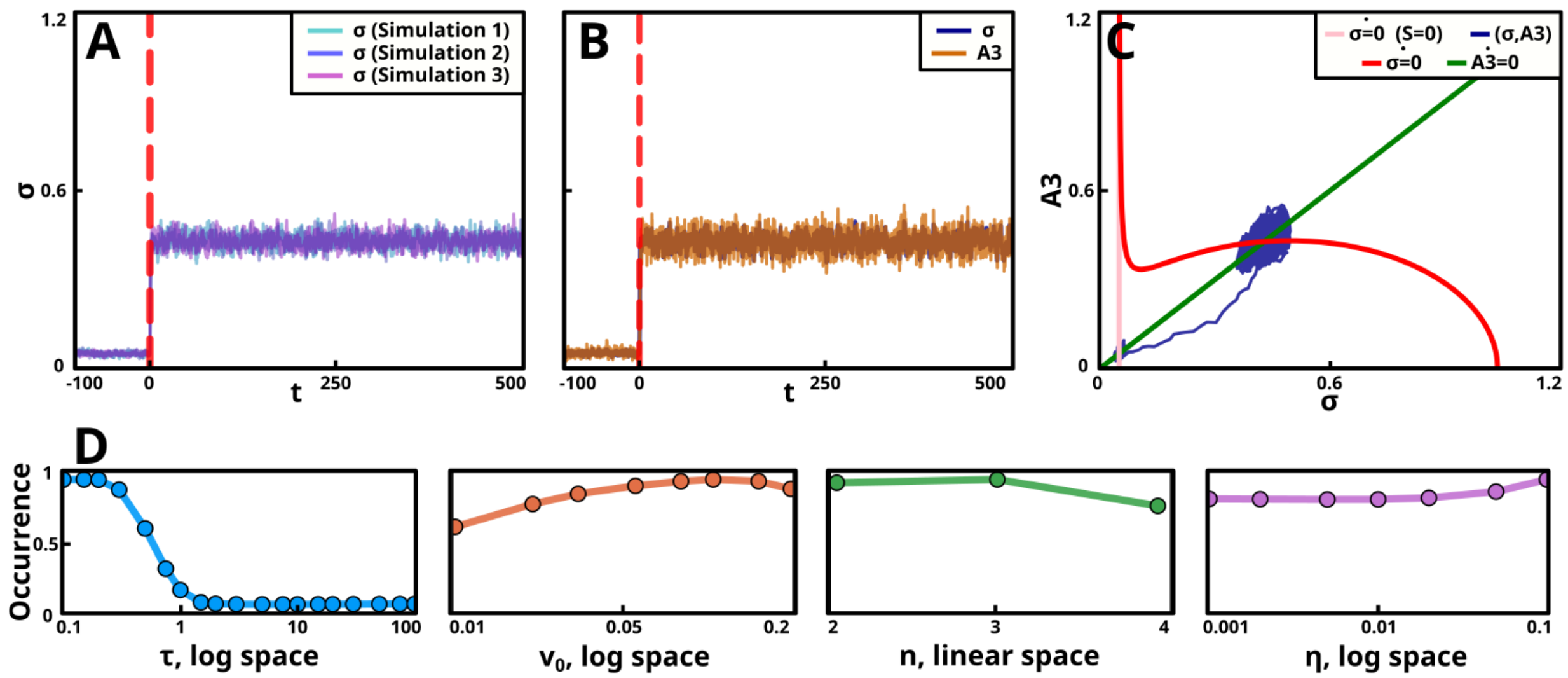

Supplementary Figure 29: **The intermediate activation behaviour.** (A-C) Simulations of the intermediate activation behaviour for the parameter set $(S, D, \tau, v_0, n, \eta) = (5.0, 5.0, 0.5, 0.05, 2, 0.05)$. (A) Three different simulations of the behaviour. (B-C) The same simulation, shown both over time and in phase space. (B) The concentration of the sigma factor (σ, blue line) and the (third) intermediary ($A = A_3$, yellow line) over time. (C) The state of the system in $[\sigma]$-$[A]$ space (blue trajectory). The nullclines: NC_A (green), and NC_σ (pink for input absent, $S = 0$, and red for input present), are also displayed. (D) The occurrence analysis of the behaviour shows how its frequency depends on the parameters τ, v_0, n, and η (Supplementary Note 8.11). Source code used to generate this figure can be found in the provided GitLab repository.

hard to determine whenever it actually was this behaviour that was observed.

8.11 Behaviour occurrence analysis

In Supplementary Figures 20-29 we, for each behaviour, measure how the frequency of its occurrence depends on the four parameters τ, v_0, n, and η. This section details how this analysis was performed. We first consider our previously-computed parameter grid of evaluated behaviours, $B(S_i, D_i, \tau_i, v_{0,i}, n_i, \eta_i)$. We define the count $\hat{C}(b, \tau_j)$ as the number of times the behaviour b occurs in the grid $B(...)$ for τ_i assuming some specific value τ_j. Here,

$$b \in \{\text{no activation, stochastic pulsing, oscillation, ...}\}$$

We then create the occurrence vector for the behaviour b and the grid $\{\tau_i\}$: $\hat{C}_{\tau,i}(b)$. Finally, we normalise $\hat{C}_{\tau,i}(b)$ by dividing every element by its maximum value. This creates $C_{\tau,i}(b)$, for which $0 \leq C_{\tau,i}(b) \leq 1 \ \forall \ i$ holds. We create similar occurrence vectors for v_0, n, and η (vectors could also have been created for S and D, but we found it simpler to consider the maps in Supplementary Figure 31). The primary purpose of this additional normalisation step, is to simplify the occurrence plots presented in 20-29.

During the count, $n = 2$ was discounted from the grid (except for when measuring the occurrence for the parameter n). The reason was that, for $n = 2$, for some values of v_0, the system's nullcline lacked local minimum and maximum, and the system could only be classified as no activation or homogeneous activation (Supplementary Note 7.2). This produced artefacts in the occurrence plots. Also, the highest value of η (0.20) was discounted as for this value a few grids had missing grid values (where we had avoided making a classification since the condition of the CLE had been broken).

Supplementary Note 9: Behaviour maps

9.1 Generating behaviour maps

We have divided the possible behaviours of our model into ten different classes (Supplementary Note 7.1). Our classification algorithm can be used to automatically assign a parameter set to one of these classes (Supplementary Note 7.3). To create our behaviour maps, we classified the system for all parameter values in a parameter grid: $\{S_i, D_i, \tau_i, v_{0,i}, n_i, \eta_i\}$. The values in S_i and D_i (for which a larger number of values was required to yield high resolution maps) were log10-distributed. The values of τ_i, $v_{0,i}$, and η_i were approximately log-distributed (but hand-picked, to avoid long decimals when writing them out). n_i was set to $\{2, 3, 4\}$.

The minimum and maximum values of S_i and D_i were chosen so that further decreases (or increases) did not noticeably change the maps (Supplementary Figure 32-35). The minimum values of τ, v_0, and η were chosen so that the time-delay, base production, and noise levels, respectively, were negligible. For the maximum value of τ the time delay was so large that further increases just made limit cycles longer, but no additional changes in behaviours were produced. For the maximum value of v_0 (0.2), it constituted a significant portion of the maximum production (1.0). For the maximum value of η (0.20) noise became so large that it obscured other behaviours (also, large noise fluctuations would increasingly make the CLE approximation less reliable). It was assumed that n (if > 1) would have no major impact on the system, and so only three values were chosen (this assumption is mostly validated in Supplementary Figures 20-29). Finally, we checked that the behaviour maps stopped changing as the parameter values reached their minimum and maximum values (Supplementary Figures 32-35).

Since the classifier was dependent on stochastic simulations, the classification of each parameter value was random. To reduce the noise we postulated that the behaviour of each parameter set was likely similar to that of its neighbours. In a single behaviour map, each grid point has 8 neighbouring grid points (in the S-D space). The colour of a grid point was chosen by a single, weighed, vote of the point and its neighbours (where the point's colour was weighed 3, its direct neighbour's colours were weighed 2, and its diagonal neighbour's colours were weighed 1).

9.2 Bifurcation diagrams across the behaviour map

We note that for small values of S, the system remains inactive (this corresponds to the behaviour when there is no input). As S is increased, the system passes through a region with some non-trivial behaviour (behaviour which is neither no activity nor homogeneous activation), to reach the homogeneous activation behaviour for large S (corresponding to a system immediately activating, and staying active, once the input is activated). Meanwhile, we see that D splits parameter space into two regions, each with its own distinct set of behaviours. For small D ($D \lesssim 1$) the system experiences a region of bistability as S is varied. This region includes the heterogeneous activation, stochastic switching, and stable bistability behaviours. For large D ($D \gtrsim 1$) the system experiences a smooth, monostable, transition from an inactive to an active state as S is increased (for large τ, the single state may be unstable). This region contains the stochastic pulsing, oscillation, stochastic anti-pulsing, single response pulse, and intermediate activation behaviours (Supplementary Figure 30). The single response pulse and intermediate activation behaviours does not occur for the parameter set used in Supplementary Figure 30, but can be found in Supplementary Figure 31.

9.3 Additional behaviour maps

To complement the behaviour map shown in the main article, we here show a large number of additional behaviour maps, permitting more careful analysis of how the parameters affect the system's behaviour.

First, we consider a succession of S-D space behaviour maps, where a single parameter (either τ, v_0, n, or η) is modulated across the series. Here, we can see how changing that parameter affects the system's

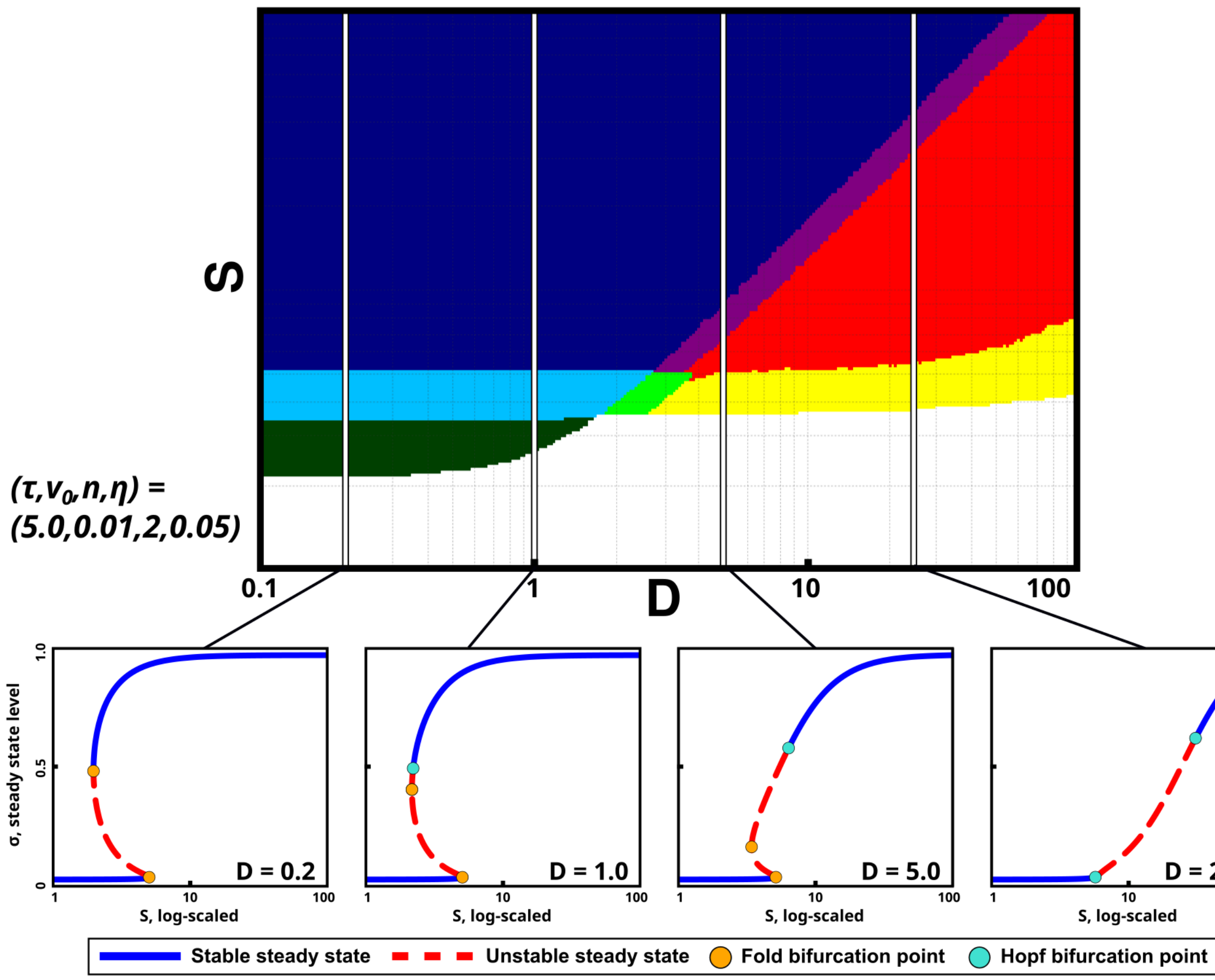

Supplementary Figure 30: **The behaviour map consists of a bistable and a single steady state part.** The same behaviour plot as in Figure 3. In addition, four lines indicate ranges of S for which the displayed bifurcation diagrams are created. In the leftmost part of the behaviour plot ($D \lesssim 1$) the system displays bistability, while in the rightmost part ($D \gtrsim 1$) the system only has a single (possibly unstable) steady state. The two regions are also characterised by two distinct sets of behaviours. Potential periodic orbits have been omitted from the bifurcation diagrams. Source code used to generate this figure can be found in the provided GitLab repository.

behaviour (Supplementary Figure 31).

If we consider τ, it has little effect on the leftmost part of the behaviour space (for low D values, where the system is bistable). This makes sense, since in this region self-deactivation is weak (which is the feedback the time-delay affects). In the rightmost part, τ affects the transition from oscillations (high τ) to intermediate activation (low τ). Again, it makes sense that oscillations require a high enough τ, since these rely on the presence of a time-delay. Finally, reducing τ also reduces the size of the stochastic pulsing and stochastic anti-pulsing regions. Again, this makes sense since these are excitable behaviours, which are related to oscillations (Supplementary Figure 31A).

Tuning v_0 has little effect on the types of behaviours encountered, however, the regions of the non-trivial behaviours are enlarged as v_0 is reduced. This especially holds for the behaviours depending on bistability (heterogeneous activation, stochastic switching, and stable bistability), as well as oscillations (Supplementary Figure 31B). This agrees with the bifurcation diagrams, where a low v_0 was shown to extend the bistability region (Supplementary Figure 14).

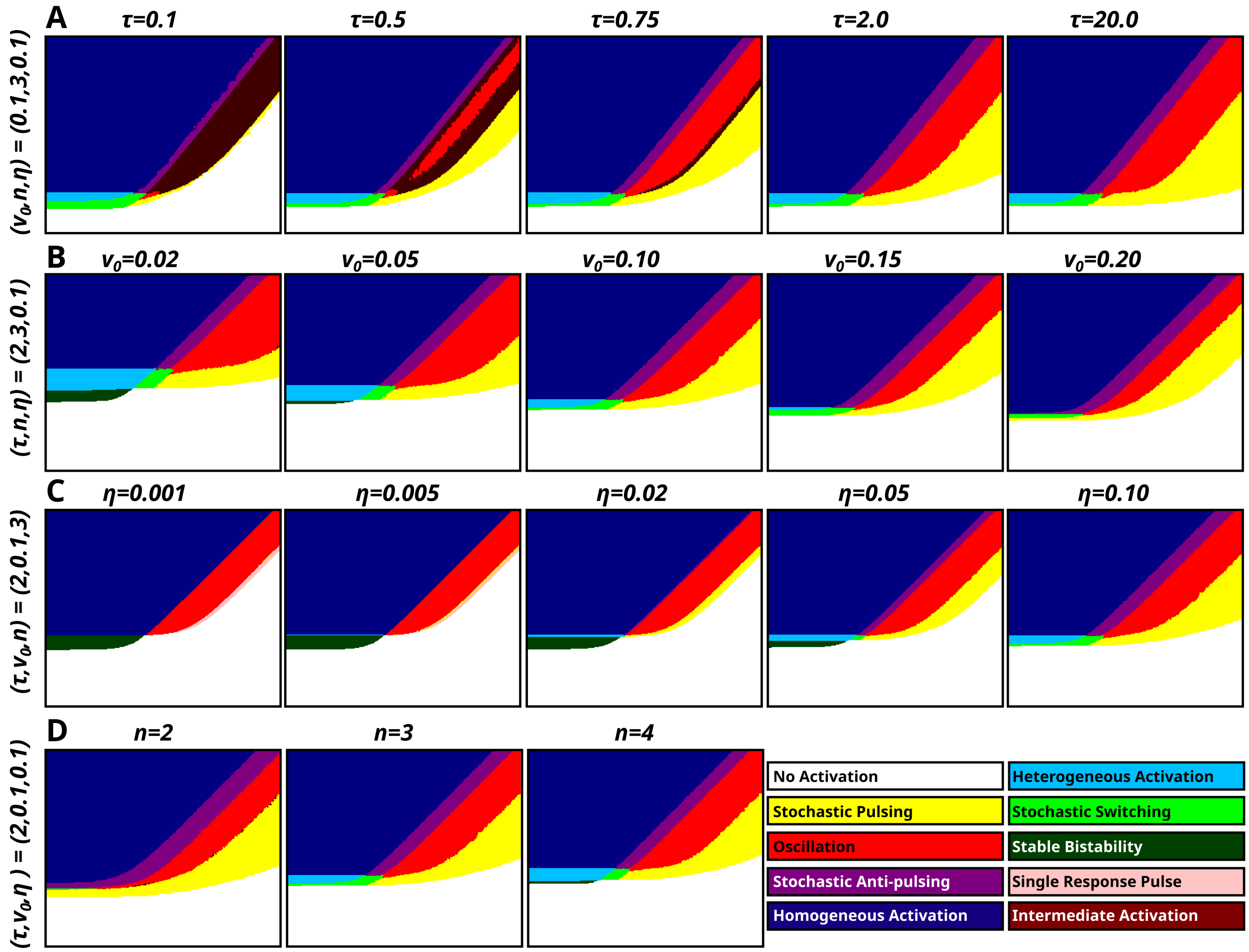

Supplementary Figure 31: **The behaviour map is modulated by the remaining four parameters.** (A-D) For all behaviour maps, the parameter set $(\tau, v_0, n, \eta) = (2.0, 0.1, 3, 0.1)$, with $S \in (1.0, 100.0)$, and $D \in (0.1, 100.0)$ is used (however, for each map one parameter is modified, as described above it in the figure). All maps are created as described in Supplementary Note 9 (A) The behaviour map for a range of τ values. (B) The behaviour map for a range of v_0 values. (C) The behaviour map for a range of η values. (D) The behaviour map for a range of n values. Source code used to generate this figure can be found in the provided GitLab repository.

Tuning of the noise parameter, η, primarily affects the system's noise dependent behaviours. All these (stochastic pulsing, stochastic anti-pulsing, stochastic switching, and heterogeneous activation) diminish as η is reduced. The stochastic switching region becomes larger (to a limit) as η increases. The heterogeneous activation region also becomes larger, but then shrinks and disappears as η becomes large enough. This is likely caused by noise becoming so large that the inactive state cannot be maintained. Finally, the stochastic pulsing and stochastic anti-pulsing regions become larger as η increases. Most notably, these regions extend into the $D \lesssim 1$ region for very high η. This might not be stochastic (anti-)pulsing due to excitability (as in the $D \gtrsim 1$ region), but is rather caused by the stochastic fluctuations becoming so large that the system will not stay reliably inactive/active. Also, the single response pulse behaviour seems to require η to be low enough (Supplementary Figure 31C).

The primary effect of changing n (the degree of ultrasensitivity) seems to be to increase the sizes of the non-trivial behaviour regions (Supplementary Figure 31D).

Finally, it is also possible to create a 2d grid of parameter maps, showing the changes with regards to two different parameters. These show similar trends as shown in Supplementary Figure 31. For a number of such grids of maps, please consider Supplementary Figures 32-35.

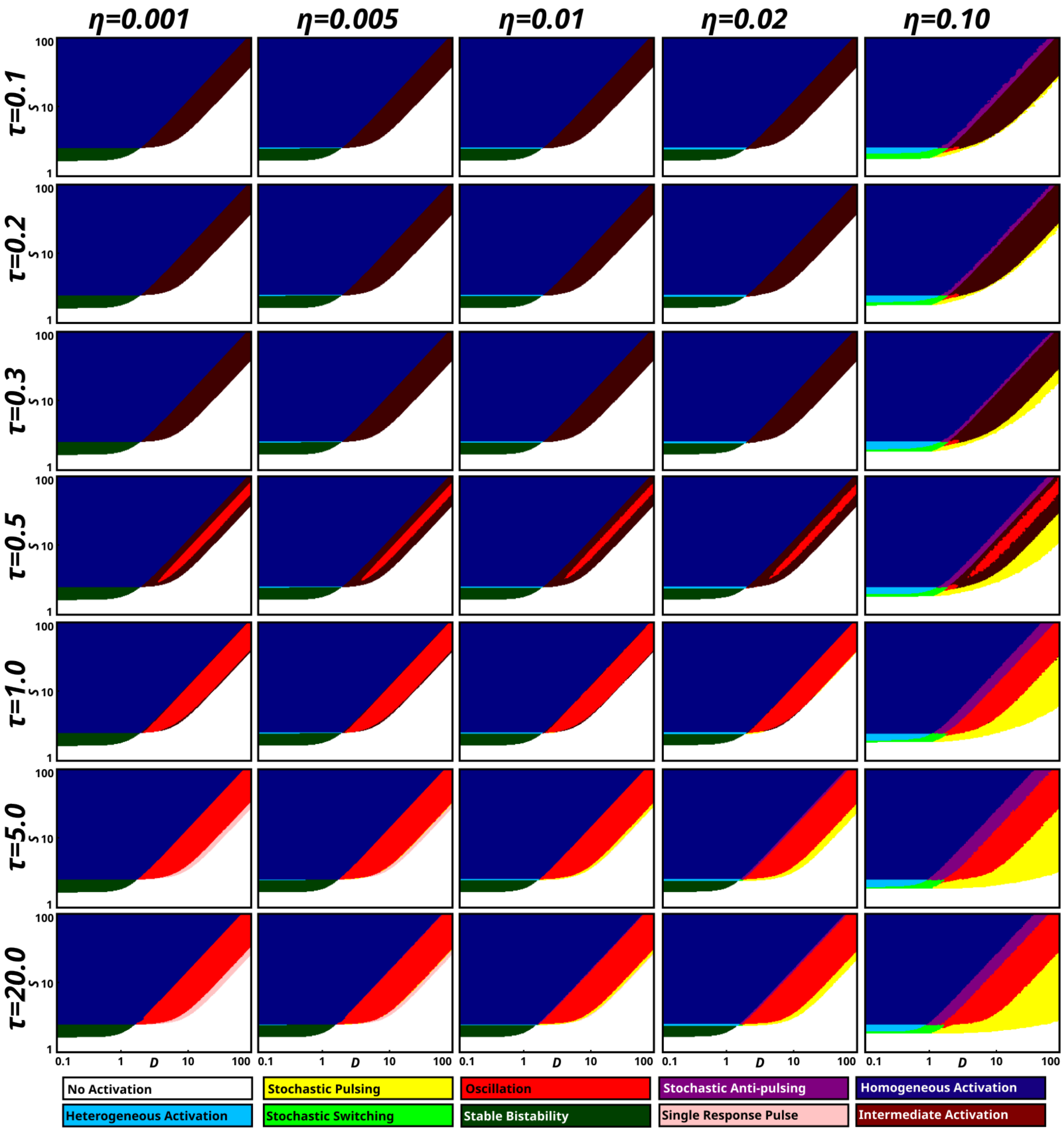

Supplementary Figure 32: **A grid of behaviour maps, for a range of values of the parameters τ and η.** The maps are plotted as described in Methods. For each map, we set $(v_0, n) = (0.1, 3)$, with $S \in (1.0, 150.0)$ (y-axis), and $D \in (0.1, 150.0)$ (x-axis) are used. The values of τ and η are modified as indicated by the grid. The four upper maps in the leftmost column ($\eta = 0.001$, $\tau \in \{4/3, 20/9, 20/3, 100/3\}$) contain a slim region of the single response pulse behaviour (between the oscillatory and the no activation regions). Source code used to generate this figure can be found in the provided GitLab repository.

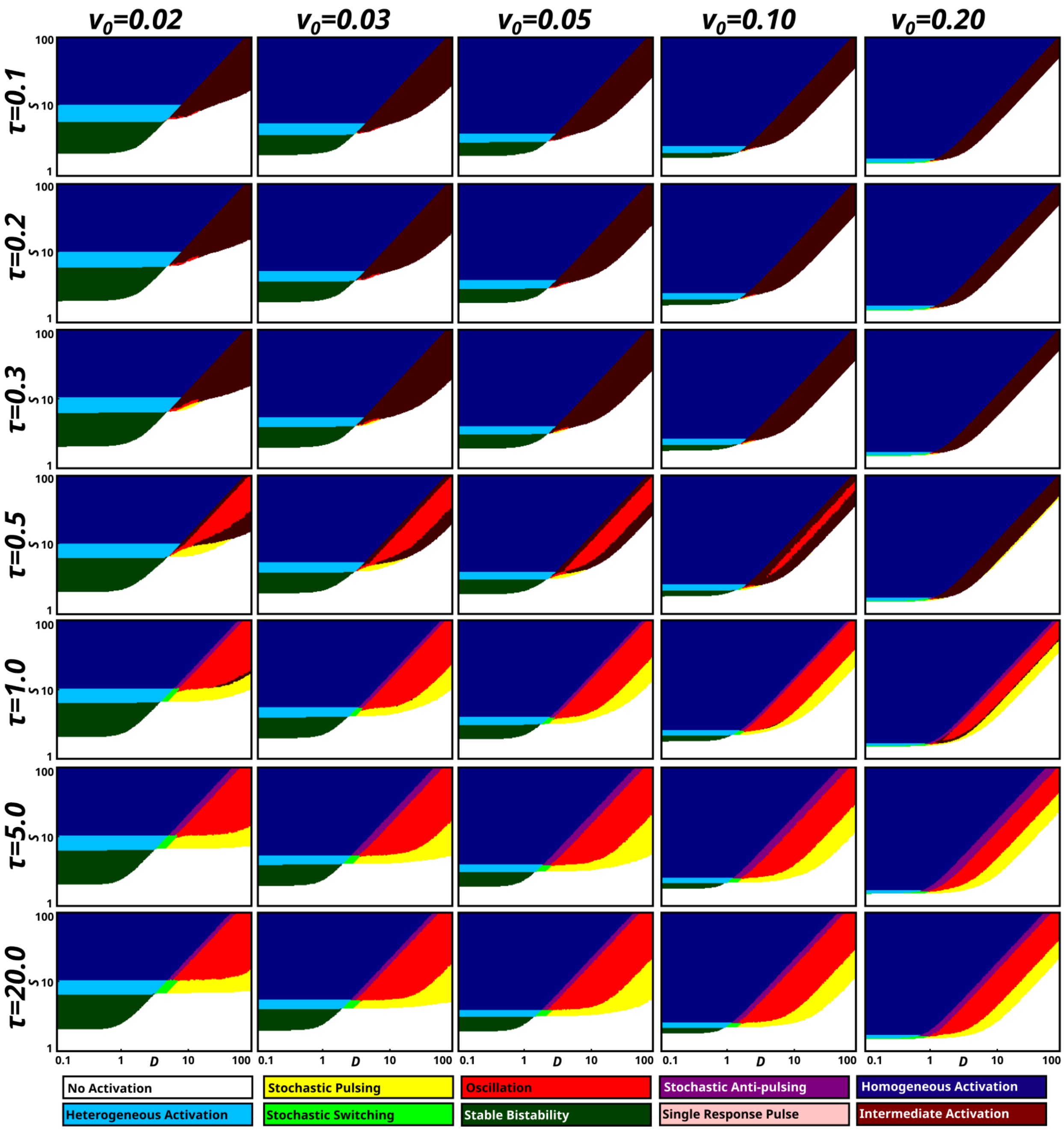

Supplementary Figure 33: **A grid of behaviour maps, for a range of values of the parameters τ and v_0.** The maps are plotted as described in Methods. For each map, we set $(n, \eta) = (3, 0.05)$, with $S \in (1.0, 150.0)$ (y-axis), and $D \in (0.1, 150.0)$ (x-axis) are used. The values of τ and v_0 are modified as indicated by the grid. Source code used to generate this figure can be found in the provided GitLab repository.

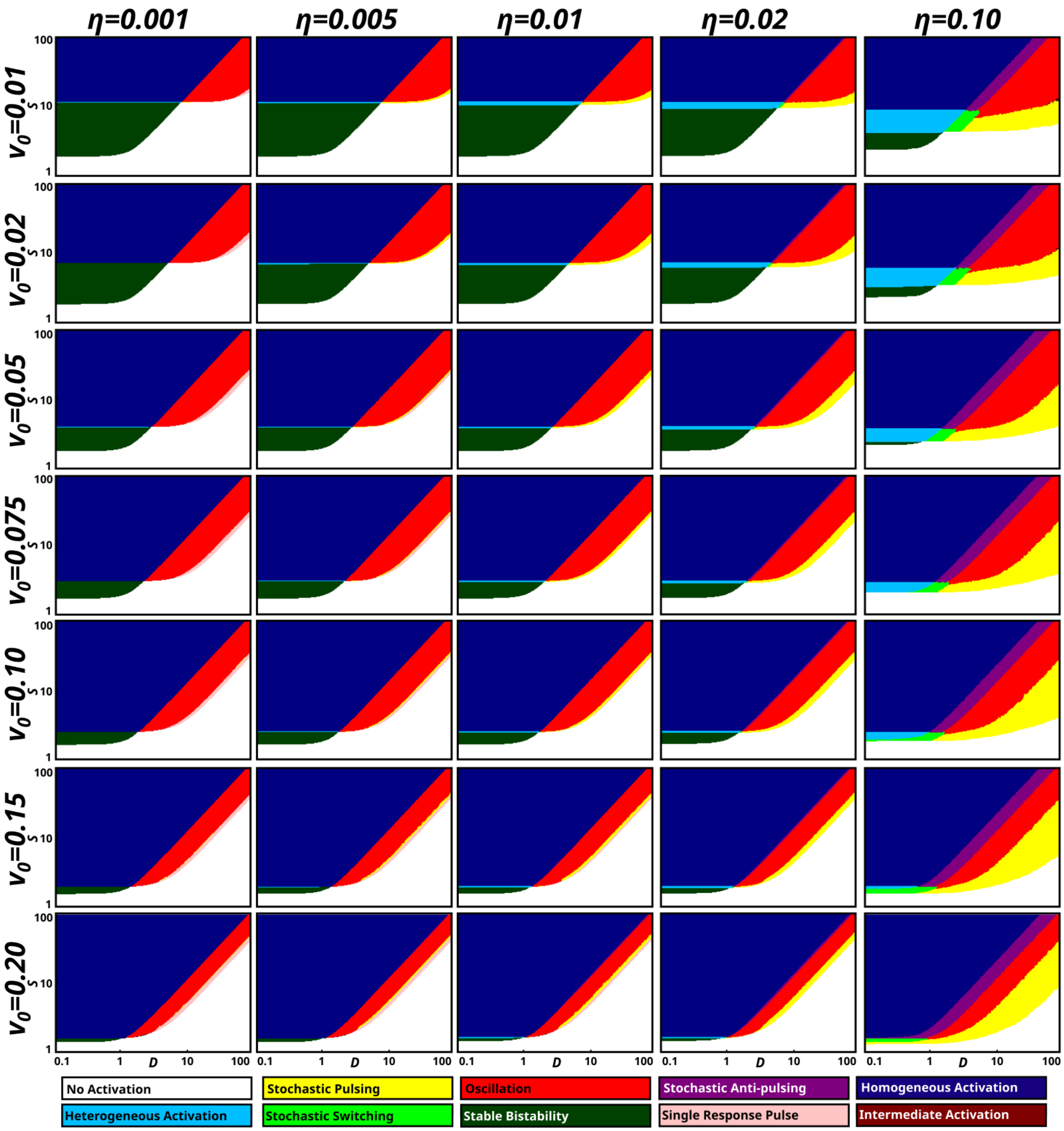

Supplementary Figure 34: **A grid of behaviour maps, for a range of values of the parameters η and v_0.** The maps are plotted as described in Methods. For each map, we set $(\tau, n) = (10/3, 3)$, with $S \in (1.0, 150.0)$ (y-axis), and $D \in (0.1, 150.0)$ (x-axis) are used. The values of η and v_0 are modified as indicated by the grid. The maps in the top two rows, and the two leftmost in the third highest row, contain a slim region of the single response pulse behaviour. Source code used to generate this figure can be found in the provided GitLab repository.

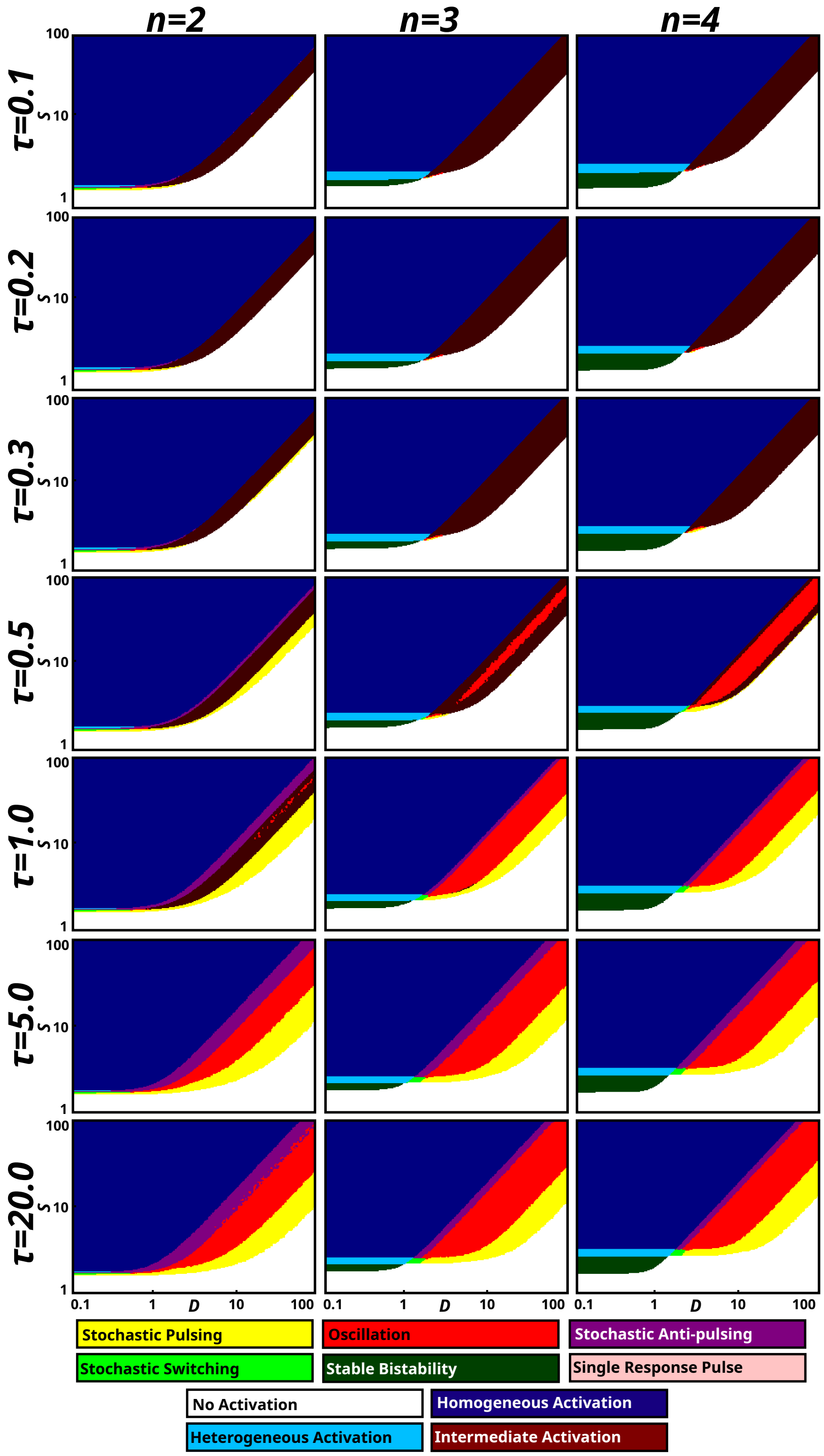

Supplementary Figure 35: **A grid of behaviour maps, for a range of values of the parameters τ and n.** The maps are plotted as described in Methods. For each map, we set $(, v_0, \eta) = (0.1, 0.05)$, with $S \in (1.0, 150.0)$ (y-axis), and $D \in (0.1, 150.0)$ (x-axis) are used. The values of τ and n are modified as indicated by the grid. Source code used to generate this figure can be found in the provided GitLab repository.

Supplementary Note 10: Behaviour transition analysis

For parts of our analysis, we depend on the relative occurrences of the various transitions between different behaviours, and how these depend on the model's parameters. This analysis is presented here.

10.1 Counting the transitions between different behaviour combinations

First, we investigated which (non-S/D) parameter(s) had the largest effect on the number of system transitions (i .e. transitions from one behaviour to another one as the critical parameter is changed). Here, τ had the largest impact on the behaviour of the system displayed. Next, v_0 and η had similar impact, with n having the smallest impact (Supplementary Table 6). Next, we scanned our 6-dimensional behaviour map, and counted all transitions from one behaviour to another one as the parameter S was varied. A list of such transitions and their counts is presented in Supplementary Table 7. In Supplementary Table 8, we instead counts the occurrence of each transition (including all behaviours as S is varied from small to large). These tables described which transitions are common (and rare) throughout behavioural space.

Supplementary Table 6: **The relative frequency of behavioural transitions per parameter.** We wanted to determine which parameter (after S and D had the highest impact on displayed behaviour. To do this, for each parameter, we computed the average number of (non-trivial) transitions as that parameter was varied across its full range of values. Here, a non-trivial transition is one not including the no activation or homogeneous activation behaviours. Here, τ had the largest impact on the displayed behaviour, followed by v_0 and η. n had the smallest effect on which behaviours were displayed.

**Parameter**	**Normalised transition count**
τ	2.4980
v_0	1.0518
n	0.3944
η	0.9203

10.2 Noise warps parameter space

It is known that noise has a tendency to "warp" systems beyond just deviations from a mean behaviour. E.g. in [30] it was noted that the response pulse amplitude (of a single response pulse type system) increased with the amount of noise (rather than creating pulse amplitude variability). Generally, this is a known phenomenon for non-linear systems. It has also been noted that noise can induce oscillations in an otherwise non-oscillating system [31]. However, the phenomenon of noise causing non-intuitive changes to system behaviours has not been studied more systematically. By creating behaviour maps of stochastic behaviours, and comparing these to the corresponding deterministic cases, we have the opportunity to carry out this investigation. Here, we can note that noise does systematically "warps" the behavioural regions, as compared to what would be expected from the deterministic cases.

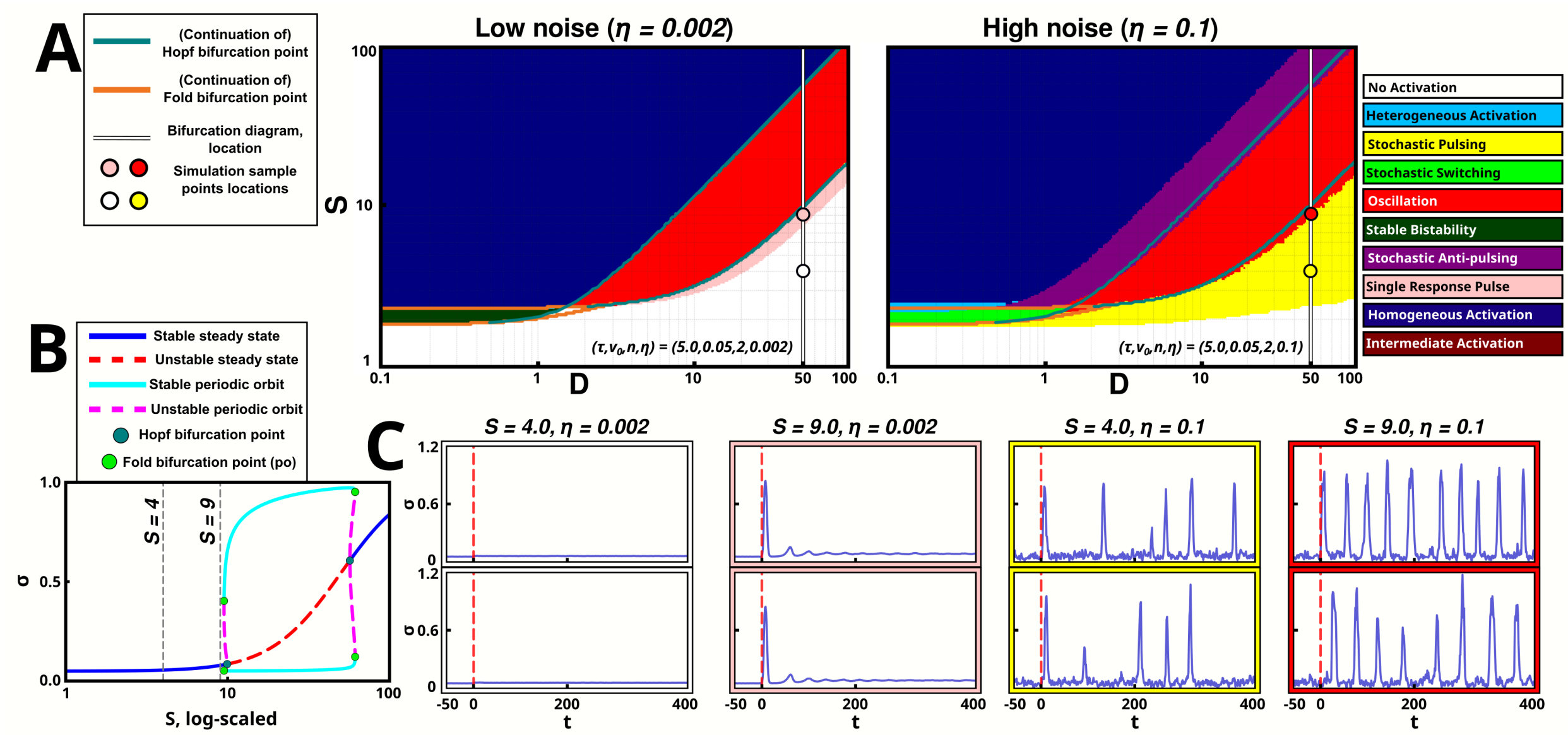

Supplementary Figure 36: **Stochasticity warps parameter space with respect to behavioural transitions.** This is an expanded version of Figure 4 in the main article text. (A) The same behaviour map, shown for a low and a high noise regime. In the map, we have plotted the bifurcation points of the deterministic system (studied in more detail in Supplementary Figures 14 and 30) across 2d (S, D)-space. Generally, the fold bifurcations are associated with bistability and the Hopf bifurcations with oscillations. We note that, for the low-noise regime, the behavioural transitions align well with the bifurcation points of the deterministic system. Meanwhile, as noise is increased, the behaviour regions shift. (B) Bifurcation diagram for the parameter S, at the marked D value ($D = 50$). The two S values (4 and 9) which are marked in A, and for which simulations are carried out in C, are marked with horizontal lines. (C) Simulations for $S = 4$ and $S = 9$ (marked by grey dashed lines in the bifurcation diagram and dots in the behaviour maps in A), for the low and high noise regimes, respectively. We note that noise transitions the no activation behaviour (at $S = 4$) to a stochastic pulsing behaviour. Next, it transitions the single response pulse behaviour (at $S = 9$) to an oscillation behaviour (even if this parameter does not exhibit a periodic orbit for the deterministic case). Source code used to generate this figure can be found in the provided GitLab repository.

Supplementary Table 7: **List of behavioural transition points.** For each behavioural transition, the behaviour before and after the transition (as S increases) is noted. Next, "Transition points" denote the number of points in our 6-dimensional parameter space where the transition occurs as S is increased one step (showing how common the transition is). The list is sorted from the most common transitions to the less common ones.

**Initial Behaviour**	**New Behaviour**	**Transition points**
No activation	Stable bistability	351372
Heterogeneous activation	Homogeneous activation	321327
Stable bistability	Heterogeneous activation	272292
No activation	Stochastic pulsing	242073
Stochastic pulsing	Oscillation	242051
Stochastic anti-pulsing	Homogeneous activation	215812
Oscillation	Stochastic anti-pulsing	200767
Intermediate activation	Homogeneous activation	135444
No activation	Intermediate activation	124724
No activation	Single response pulse	103933
Oscillation	Homogeneous activation	95710
No activation	Homogeneous activation	81993
Stable bistability	Homogeneous activation	79009
Single response pulse	Oscillation	56565
Single response pulse	Stochastic pulsing	43174
Stochastic switching	Heterogeneous activation	29945
No activation	Stochastic switching	28820
Stochastic pulsing	Intermediate activation	28133
No activation	Oscillation	24667
Intermediate activation	Stochastic anti-pulsing	20040
Stochastic pulsing	Stochastic switching	18355
Stochastic switching	Stochastic anti-pulsing	15824
No activation	Heterogeneous activation	9248
Stochastic switching	Oscillation	5226
Single response pulse	Intermediate activation	4615
Stochastic anti-pulsing	Heterogeneous activation	2161
Intermediate activation	Heterogeneous activation	2019

Supplementary Table 8: **List of full behavioural transitions.** For each possible transition where S goes from small to large in our behaviour map, we check the behaviours it transitions through (taking the first occurrence of each behaviour). Here, we list the 20 most common such transitions and how frequently they occur. With one exception (intermediate activation to oscillation to intermediate activation), transition from, and then back into, a behaviour should not occur. To reduce noise, we have filtered out such cases. This includes some cases of transitions that include int. act. → oscillation → int. act., which is a fairly common (valid) transition (and the one exception from this list).

**Transition**	**Count**
no act. → stable bistability → het. act. → hom. act.	272202
no act. → stoch. pulsing → oscillation → stoch. anti-pulsing → hom. act.	129105
no act. → int. act. → hom. act.	84198
no act. → hom. act.	81557
no act. → stable bistability → hom. act.	79009
no act. → single response pulse → oscillation → hom. act.	34748
no act. → int. act. → oscillation → hom. act.	31226
no act. → stoch. switching → het. act. → hom. act.	25560
no act. → stoch. pulsing → oscillation → hom. act.	19613
no act. → single response pulse → stoch. pulsing → oscillation → stoch. anti-pulsing → hom. act.	18638
no act. → single response pulse → stoch. pulsing → oscillation → hom. act.	17344
no act. → oscillation → hom. act.	13413
no act. → stoch. pulsing → int. act. → oscillation → stoch. anti-pulsing → hom. act.	10737
no act. → stoch. pulsing → stoch. switching → stoch. anti-pulsing → hom. act.	10208
no act. → single response pulse → oscillation → stoch. anti-pulsing → hom. act.	9612
no act. → het. act. → hom. act.	9147
no act. → stoch. pulsing → int. act. → stoch. anti-pulsing → hom. act.	8616
no act. → oscillation → int. act. → hom. act.	7270
no act. → single response pulse → oscillation → int. act. → hom. act.	8616
no act. → stoch. pulsing → oscillation → int. act. → hom. act.	7270

Supplementary Note 11: Additional experimental data

11.1 RT-qPCR validation of σ^V behaviours

In Supplementary Figure 37, we confirmed via RT-qPCR that adding additional copies of *rsiV* decreased the levels of *sigV* expression.

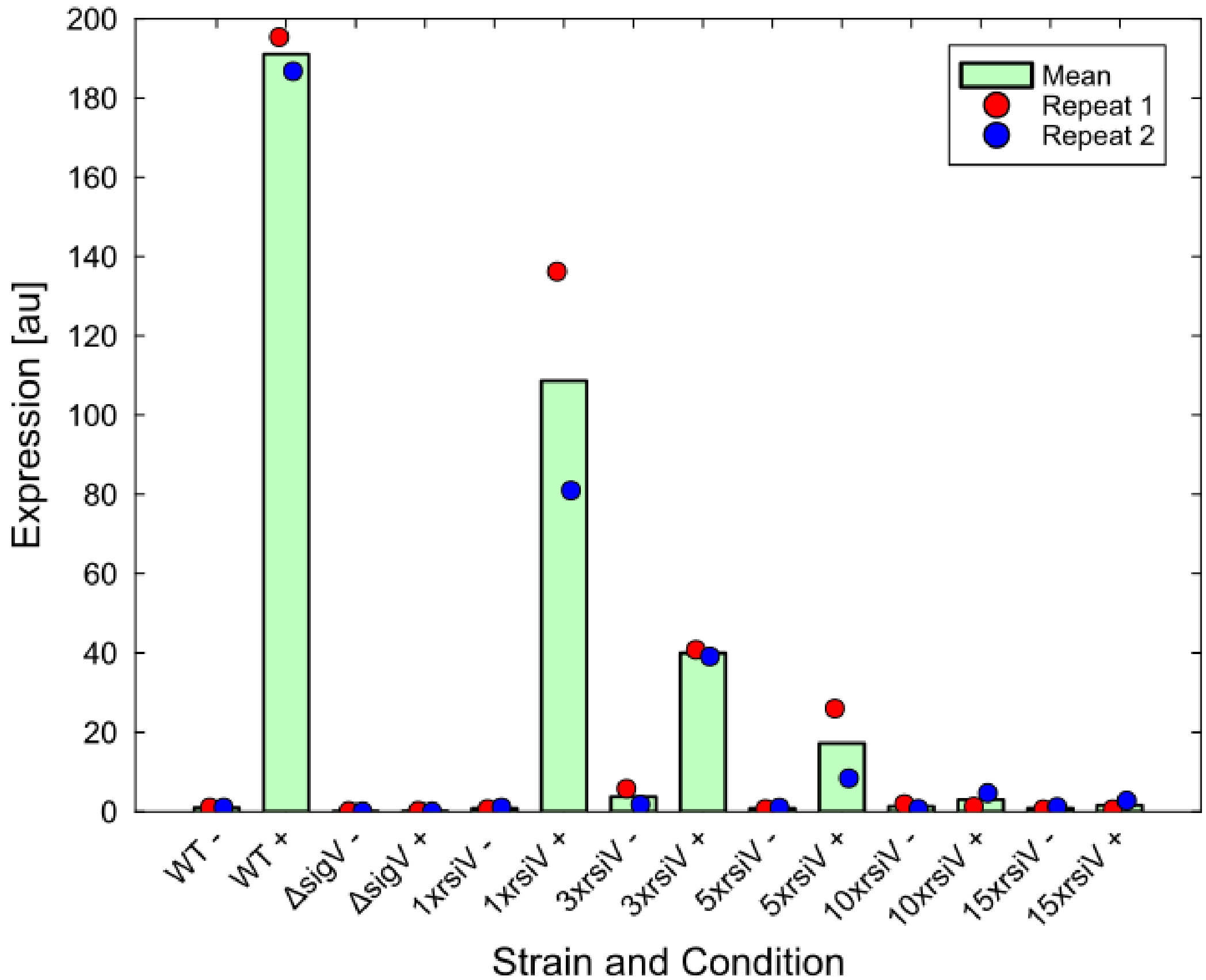

Supplementary Figure 37: **With increasing *rsiV* copy number σ^V expression is suppressed.** Bars show normalised RT-qPCR expression levels for each strain after 30 min either under 0 µg/ml lysozyme (-) or 1 µg/ml lysozyme (+). Red dots represent Repeat 1 and blue dots represent Repeat 2. Expression values were normalised to the internal reference gene *rpoC* and are plotted without scaling between repeats. Source data for this figure is available in the Source Data file.

11.2 Additional σ^V data

Additional replicate measurements of the σ^V system, not displayed in the main manuscript, are displayed in Supplementary Figures 38, 39.

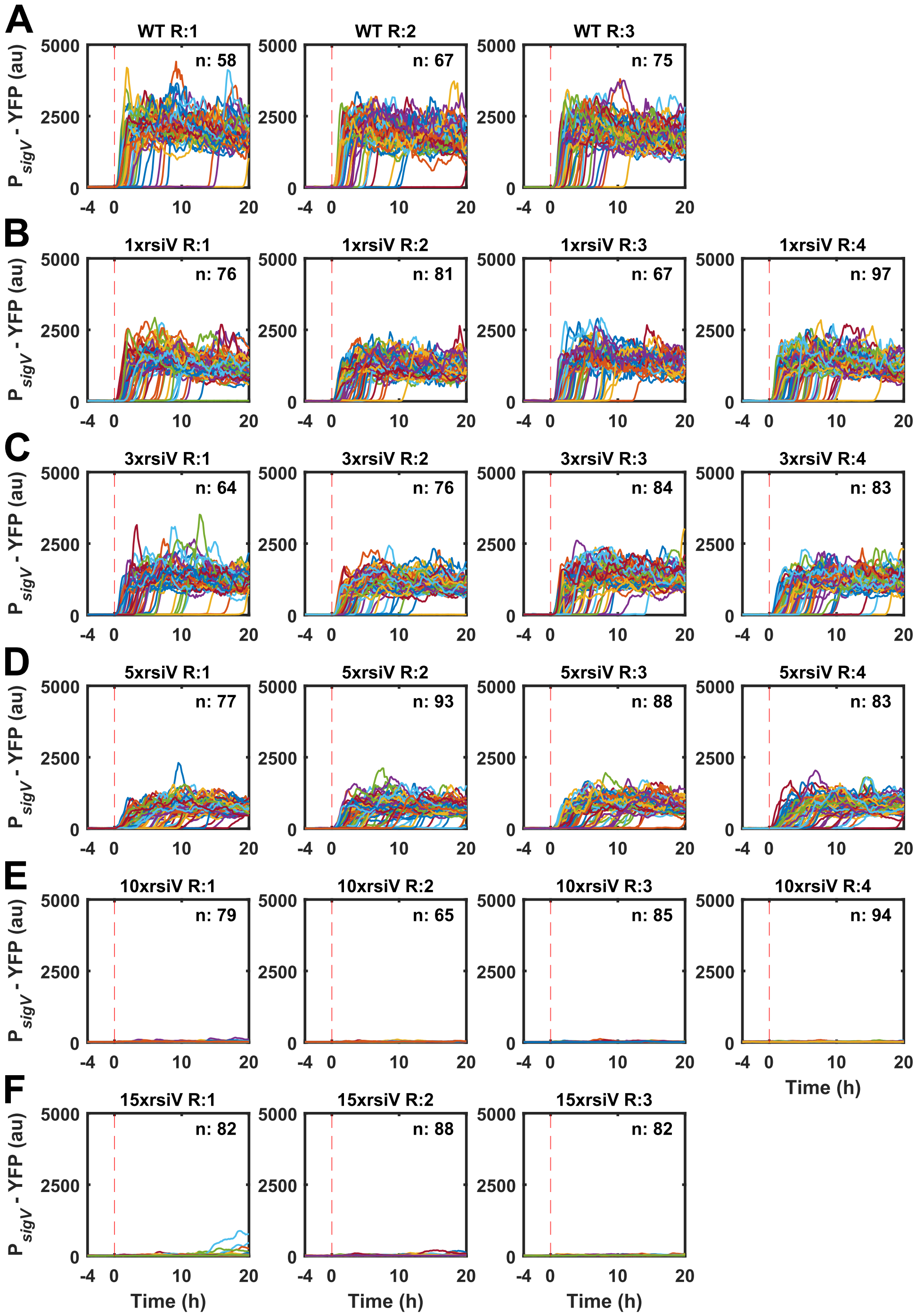

Supplementary Figure 38: **With increasing copy number of *rsiV* single cell P_{sigV}-YFP is decreasing under 1 µg/ml Lysozyme stress.** All shown repeats were grown overnight in the mother machine with no stress before exposing them to 1 µg/ml Lysozyme at time 0h. Each row is one *rsiV* mutant and each column is a repeat. A) WT (JLB130), B) 1x *rsiV* (JLB212), C) 3x *rsiV* (JLB288), D) 5x *rsiV* (JLB293), E) 10x rsiV (JLB325), F) 15x *rsiV* (JLB324). The trajectory colour represents the P_{sigV}-YFP time trajectory of one cell. n indicates the number of cells in each subplot. Source data for this figure is available in the Source Data file.

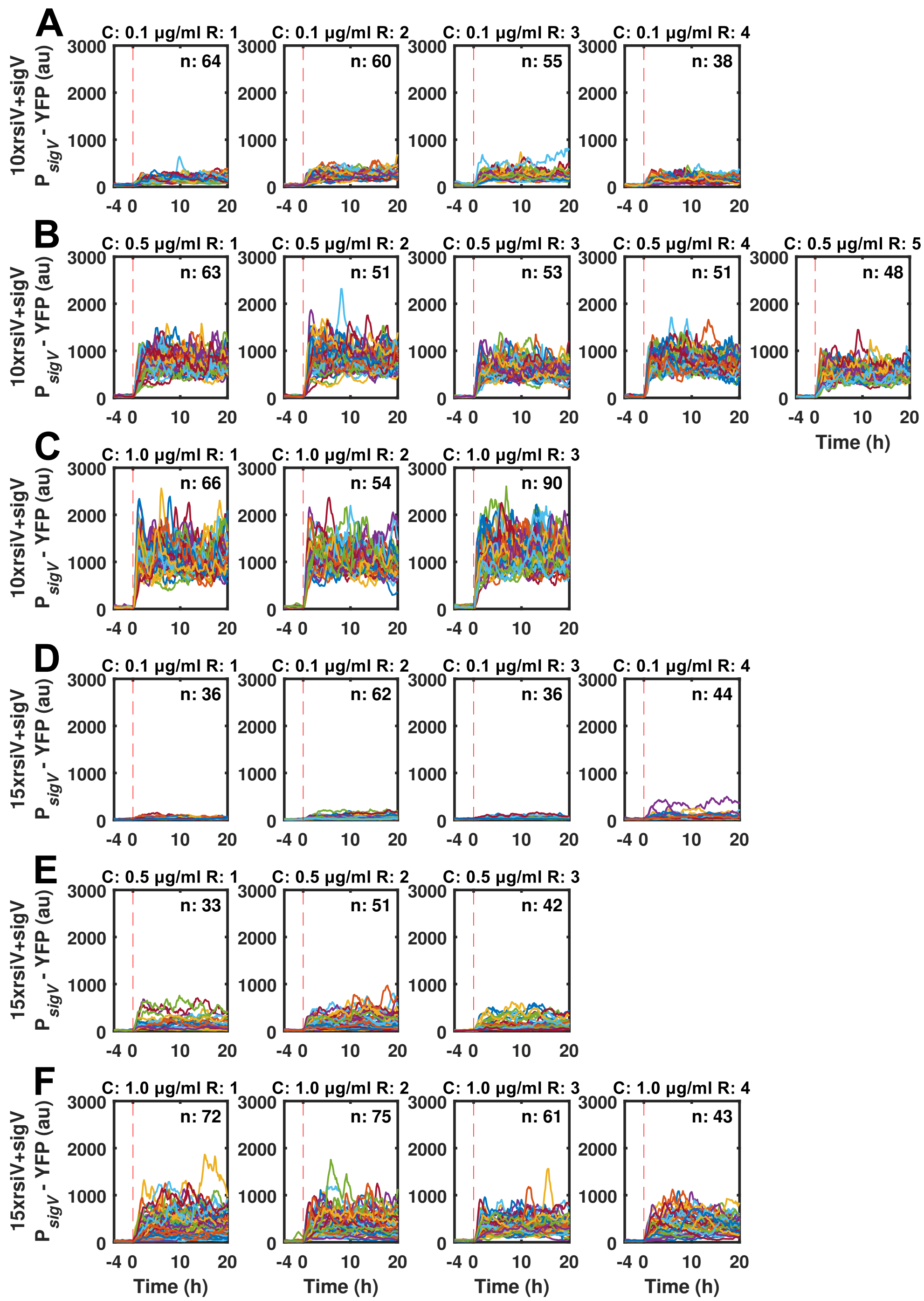

Supplementary Figure 39: **Adding an extra copy of *sigV* to 10x *rsiV* or 15x *rsiV* moves the behaviour into the intermediate regime.** All shown repeats were grown overnight in the mother machine with no stress before exposing them to 0.1, 0.5 or 1.0 µg/ml Lysozyme at time 0h. Each row is one *rsiV* mutant with its respective lysozyme concentration and each column is a repeat. A) 10x *rsiV* and *sigV* (JLB327) + 0.1 µg/ml Lysozyme, B) 10x rsiV and *sigV* (JLB327) + 0.5 µg/ml Lysozyme, C) 10x rsiV and *sigV* (JLB327) + 1.0 µg/ml Lysozyme, D) 15x rsiV and *sigV* (JLB345) + 0.1 µg/ml Lysozyme, E) 15x rsiV and *sigV* (JLB345) + 0.5 µg/ml Lysozyme, F) 15x *rsiV* and *sigV* (JLB345) + 1.0 µg/ml Lysozyme. The trajectory colour represents the P_{sigV}-YFP time trajectory of one cell. n indicates the number of cells in each subplot. Source data for this figure is available in the Source Data file.

11.3 Additional σ^B data

Additional replicate measurements of the σ^B system, not displayed in the main manuscript, are displayed in Supplementary Figures 40-43.

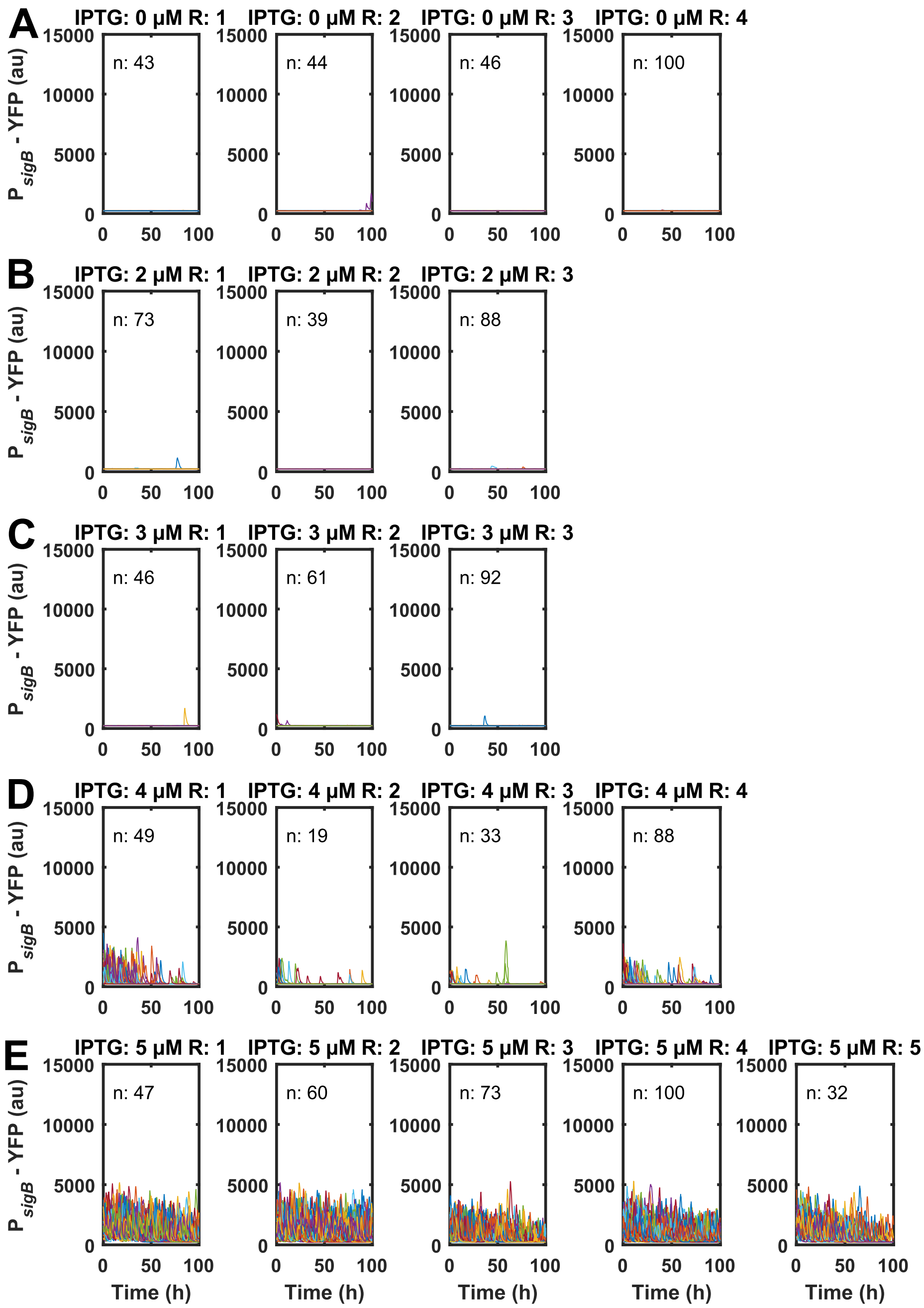

Supplementary Figure 40: **In the Phs-rsbPΔ4 (ΔPAS-CC) ΔrsbQ ΔrsbU mutant we can tune the P_{sigB}-YFP output behaviour based on the IPTG levels.** The Phs - rsbPΔ4 (ΔPAS-CC) ΔrsbQ ΔrsbU mutant (JLB346) was grown with the following IPTG levels: A) 0 μM, B) 2 μM, C) 3 μM, D) 4 μM, E) 5 μM. The trajectory colour represents the P_{sigB}-YFP time trajectory of one cell. n indicates the number of cells in each subplot. Source data for this figure is available in the Source Data file.

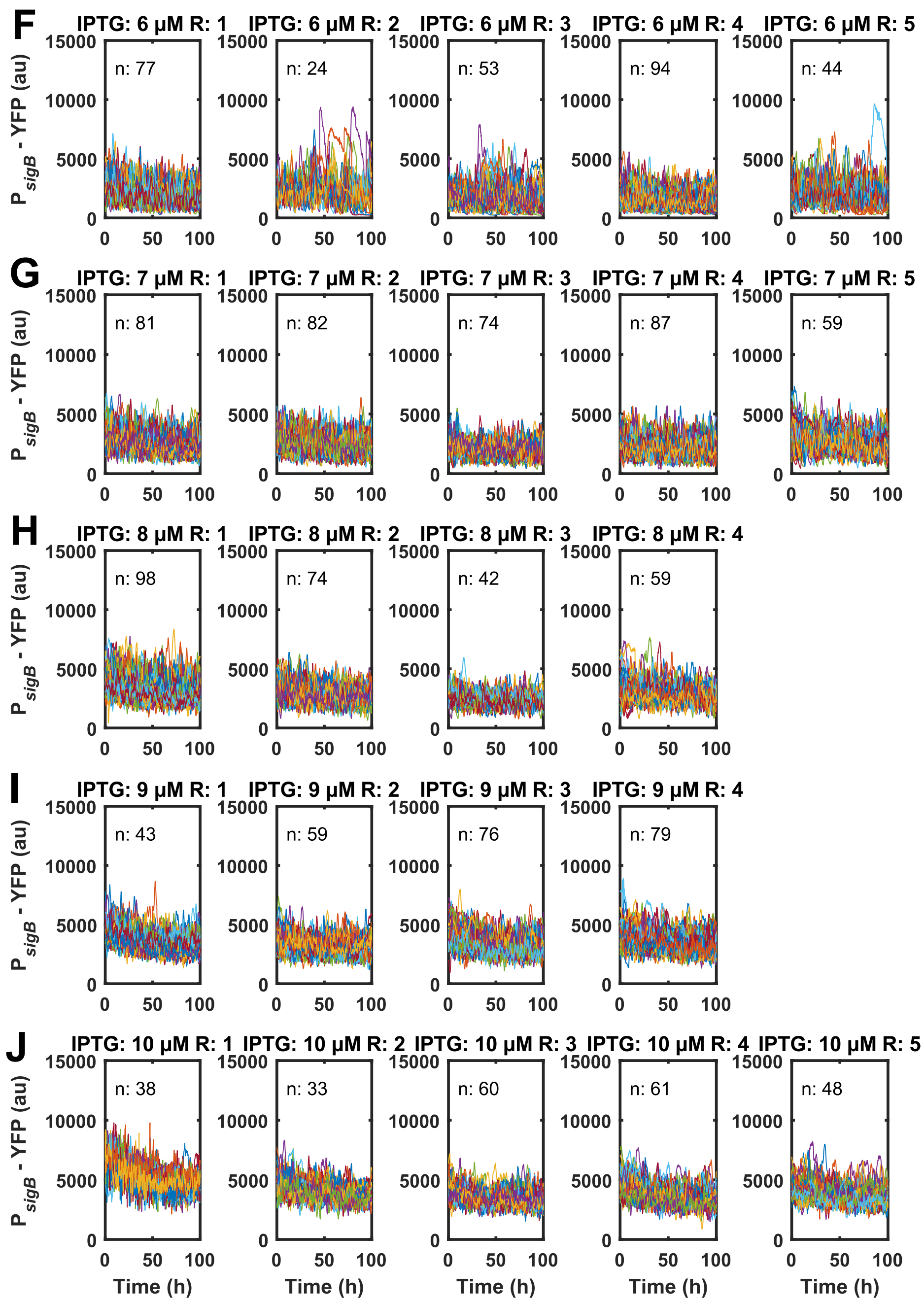

Supplementary Figure 41: **In the Phs - rsbPΔ4 (ΔPAS-CC) ΔrsbQ ΔrsbU mutant we can tune the P_{sigB}-YFP output behaviour based on the IPTG levels.** The Phs - rsbPΔ4 (ΔPAS-CC) ΔrsbQ ΔrsbU mutant (JLB346) was grown with the following IPTG levels: F) 6 μM, G) 7 μM, H) 8 μM, I) 9 μM, J) 10 μM. The trajectory colour represents the P_{sigB}-YFP time trajectory of one cell. n indicates the number of cells in each subplot. Source data for this figure is available in the Source Data file.

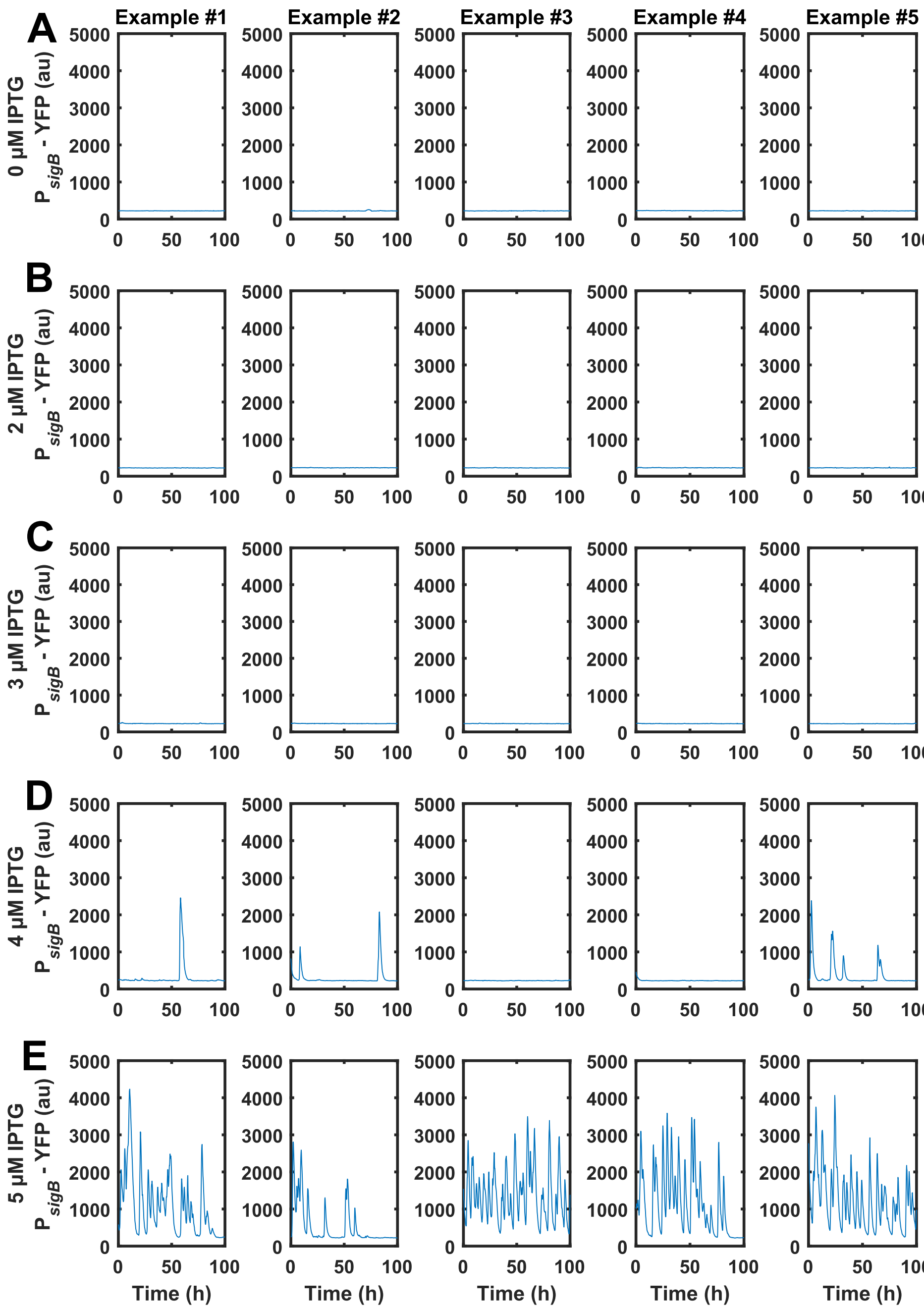

Supplementary Figure 42: **In the Phs - rsbPΔ4 (ΔPAS-CC) ΔrsbQ ΔrsbU mutant we can tune the P_{sigB}-YFP output behaviour based on the IPTG levels on a single cell level.** The Phs - rsbPΔ4 (ΔPAS-CC) ΔrsbQ ΔrsbU mutant (JLB346) was grown with the following IPTG levels: A) 0 µM, B) 2 µM, C) 3 µM, D) 4 µM, E) 5 µM. In each subfigure the P_{sigB}-YFP time trajectory of one cell is plotted (blue line). Source data for this figure is available in the Source Data file.

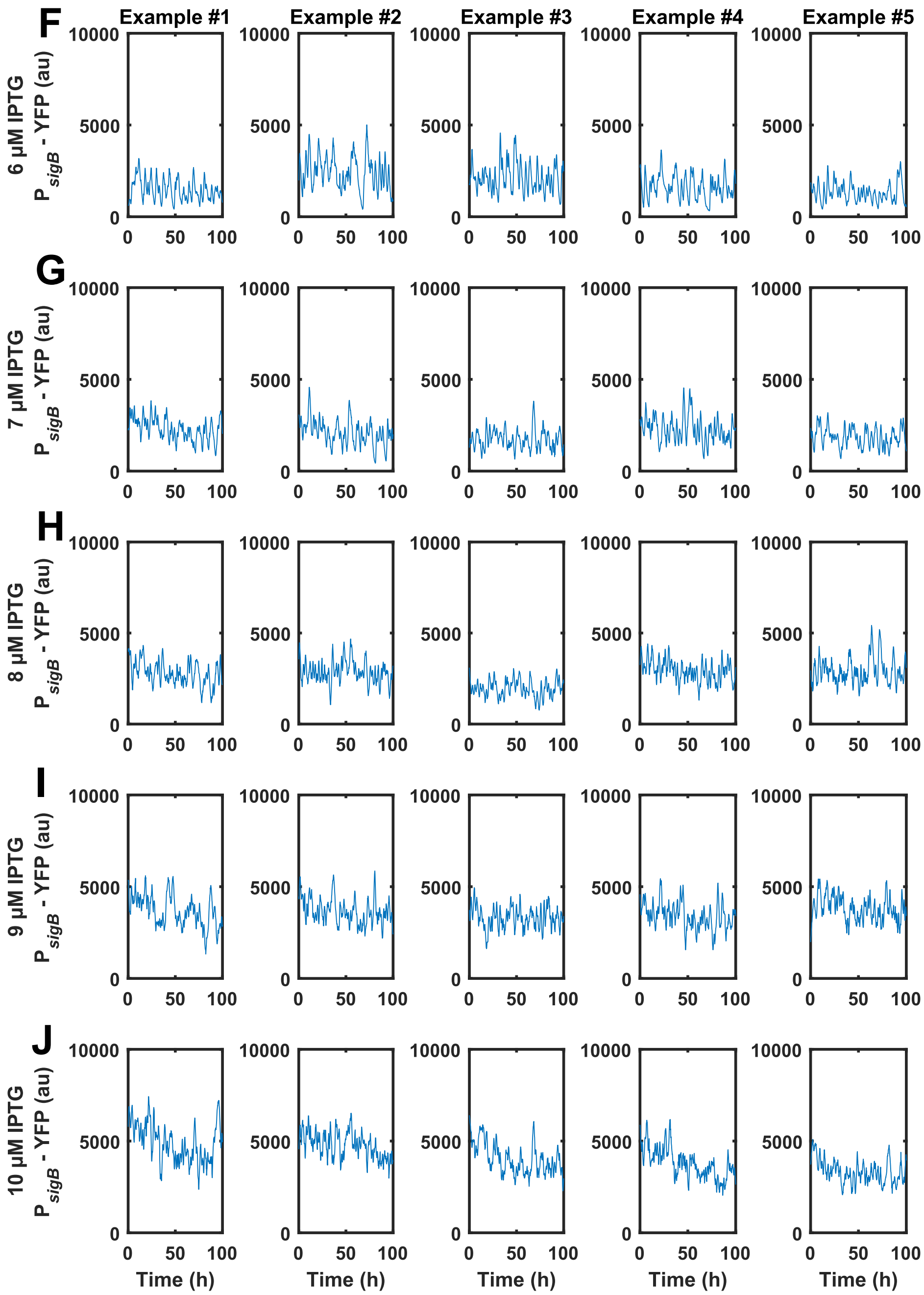

Supplementary Figure 43: **In the Phs - rsbPΔ4 (ΔPAS-CC) ΔrsbQ ΔrsbU mutant we can tune the P_{sigB}-YFP output behaviour based on the IPTG levels on a single cell level.** The Phs - rsbPΔ4 (ΔPAS-CC) ΔrsbQ ΔrsbU mutant (JLB346) was grown with the following IPTG levels: F) 6 µM, G) 7 µM, H) 8 µM, I) 9 µM, J) 10 µM. In each subfigure the P_{sigB}-YFP time trajectory of one cell is plotted (blue line). Source data for this figure is available in the Source Data file.

Supplementary Note 12: Generalised classifier

The classifier described previously is designed to assign behaviours to *points in parameter space of the model.* It does so by performing steady state analysis (taking into account the stochastic nature of the system), and then performing multiple simulations from various points in phase space, determining transition times, and using this to find a corresponding classification. This approach is not applicable to the experiments, where we instead only have access to single trajectories. This prevents us from using steady state analysis and also from performing multiple follow-up simulations. To confirm that the experimental systems undergo similar transitions as the model, we need a generalised classifier that can be applied to both experimentally measured and simulated trajectories.

Our generalised classifier is based on a machine learning/neural network approach. By generating large amounts of simulated training data, and augmenting it with experimentally measured trajectories, we generalise the principles of our specialised classifier to one which can directly assign classifications to single trajectories. This section describes the generalised classifier. Finally, to validate our model, we use a combination of new repeats from the experimental systems that were not used in the training, as well as from new experimental systems that were not used in training, and exhibiting behaviours only seen in the simulated training data. The success of these validations confirms that our classifier enables a generalised version of our previous one, but which can instead classify single trajectories either from simulations or experiments.

12.1 Generalised classifier description

The input to a classifier is a trajectory, and the output is one of the 10 behaviours described in Supplementary Note 7.1. The classifier can handle both

- Asymptotic trajectories: I.e. where the system is considered to be in its steady state distribution throughout the trajectory.
- Transient trajectories. I.e. where the system is considered to be inactive at the first time point, however, the input has just been activated. Here, the trajectory will experience an initially transient response followed by its asymptotic behaviour. The heterogeneous activation and single response pulse behaviours can only be detected for transient trajectories.

From a trajectory, a number of characteristic features are extracted. Examples include mean activity, standard deviation, maximum value, and skewness. To these, a feature describing whether the trajectory is of the steady state or transient type is added. These features are used as input to a neural network (trained on training data, Supplementary Note 12.2), which assigns an output class.

To ensure robustness, we train a large number of neural networks ($n = 60$) with different seeds. The final class is assigned based on the majority vote of these neural networks. The neural networks used the Leaky ReLU activation function, and contain 64, followed by 32, followed by 16 nodes per hidden layer. We use a combination of Flux.jl and MLJ.jl to implement the classifier [32, 33].

All trajectories used for training and testing are normalised to have values in the range $(0, 1)$ before feature extraction. Here, for each trajectory, we estimate a minimum and maximum possible value. For simulated trajectories, minimums are estimated by setting $S = 0$, and finding the minimum value encountered over a long simulation. Similarly, the maximum value is attained by setting S very high (in practise, $S = 100$ is typically used) and performing a long simulation. For experiments, for each repeat (which contains multiple trajectories from multiple stress levels, including no stress), we find the minimum and maximum values occurring across all trajectories across all stress levels. Next, for a trajectory σ_i with $(\sigma_{min}, \sigma_{max})$ we generate the new normalised trajectory $(\sigma_i - \sigma_{min})/(\sigma_{max} - \sigma_{min})$. Note here that the trajectories in practise are discrete vectors, either due to experimental sampling of the continuous process, or as the discrete time points at which the simulation is saved.

Finally, we note that, for single trajectories, the stable bistability behaviour cannot be distinguished from

the no activation behaviour. Hence, we will not consider the stable bistability behaviour for this classifier. Next, the name of the homogeneous activation behaviour is slightly non-intuitive in the asymptotic case (i.e. where the system is only measured in the steady state distribution). Here, this simply denotes trajectories that are perpetually active without showing any other form of behaviour (just like homogeneous activation in the transient case, after it has activated).

12.2 Generalised classifier training data

We train the classifier on a combination of simulated data and experimental data from the σ^{B} and σ^{V} systems. For the simulations, for all nine relevant behaviours (i.e. the ten main behaviours with stable bistability excluded), we sample a large number of parameter sets exhibiting that behaviour ($n = 1,000$). We do this for both the asymptotic and transient cases. For each behaviour, we also carry out a filter where we check that the simulated trajectory belongs to that behaviour. I.e. a parameter set may correspond to stochastic pulsing, however, this does not mean that a pulse will necessarily occur in every single (finite) simulation from that parameter set. Such a trajectory should not be used in the training data, and a check is used to repeat the simulations that do not exhibit the correct behaviour. This example also demonstrates a fundamental difference between classifying parameter sets and trajectories. I.e., while a *parameter set* may exhibit a certain behaviour, it might not necessarily be exhibited by every single *trajectory* simulated from that parameter set (due to the fundamental nature of stochastic behaviours).

Our experiments measuring the activity of the σ^{B} and σ^{V} systems are divided into multiple repeats. Here, we divide the repeats into those used for training and those used for testing. This way, when evaluating our classifier on an experimental trajectory, we will not have trained on a trajectory from the same repeat.

Finally, we used a semi-automated, semi-manual approach to assign ground-truth behaviours to each experimental training trajectory. Here, we used heuristics (such as the presence of low activity value only, or a delayed onset of activity) to assign initial classes to each trajectory. Next, we manually inspected the trajectories to ensure that these heuristics yielded accurate classes.

12.3 Generalised classifier validation

To confirm that the neural networks have correctly learnt to identify the different behaviours, we validate it on additional trajectories on which they were not trained. This includes simulated trajectories (both at steady state and with a transient) and experimentally measured ones from the σ^{B} and σ^{V} systems.

Finally, we performed additional experiments measuring σ^{M} activity in *Bacillus subtilis* in response to bacitracin stress. In total, we analysed 379 single-cell trajectories across three conditions (0, 10, and 25 μg/ml bacitracin), with each condition measured in 3-4 biological repeats. Here, the system exhibits pulsing behaviours in σ^{M} activity. Notably, this includes the single response pulse behaviour. This behaviour is only encountered in simulated training data, not experimental training data. Furthermore, the classifier has never been trained on the σ^{M} system. By confirming that the classifier can correctly identify the σ^{M} trajectories, we can confirm that it is able to learn from information both in simulated and experimental data, and use this to correctly assign classes to trajectories with no direct relation to the training set.

Randomly selected training example classifications for all testing sets and all behaviours can be found in Supplementary Figures 44-50.

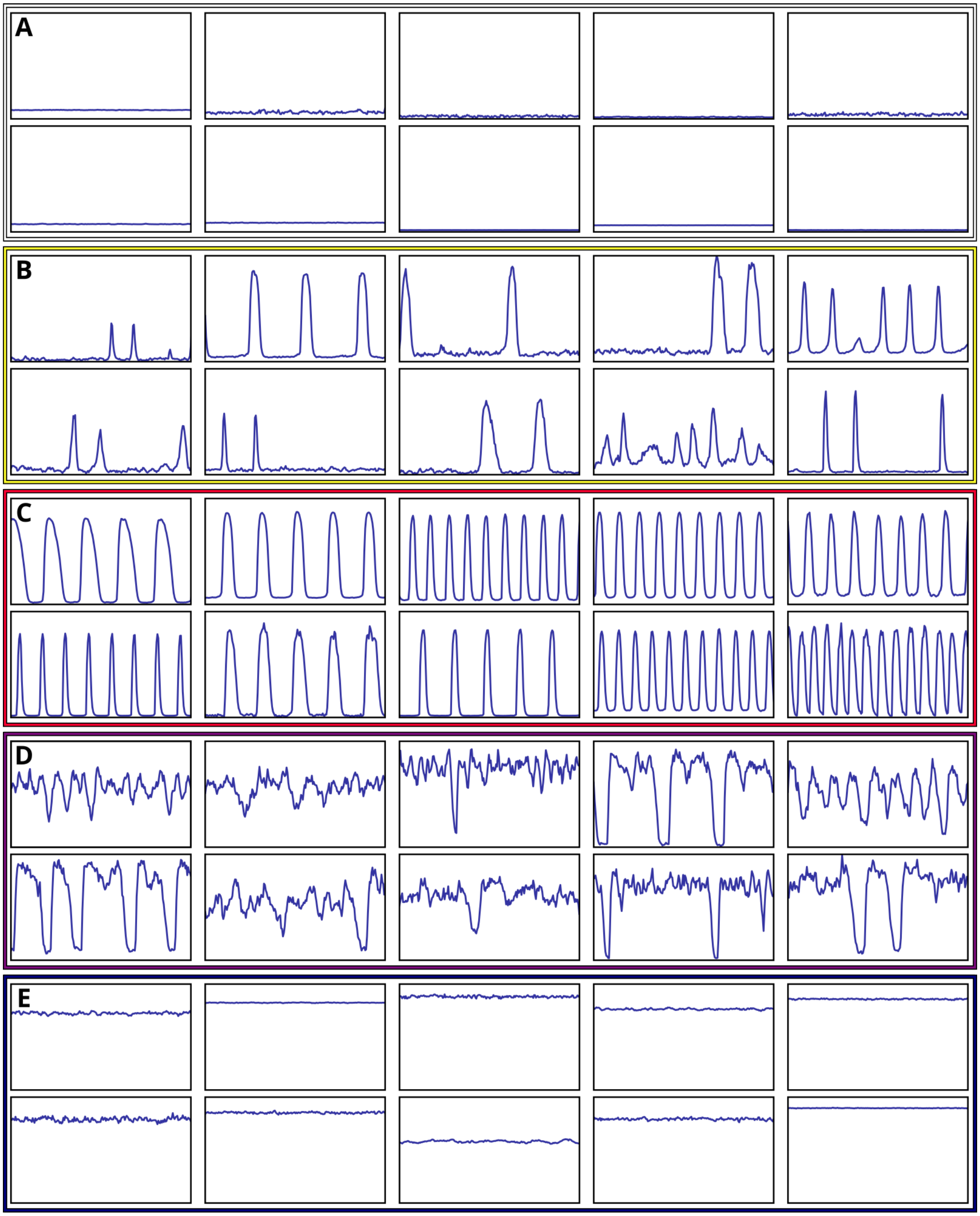

Supplementary Figure 44: **Example asymptotic model simulations and the generalised classifier's corresponding classification part 1.** For each relevant behaviour, 10 trajectories were randomly selected from those that were assigned to that behaviour. (A) Sample simulated trajectories that were classified as the "no activation" behaviour. (B) Sample simulated trajectories that were classified as the "stochastic pulsing" behaviour. (C) Sample simulated trajectories that were classified as the "oscillation" behaviour. (D) Sample simulated trajectories that were classified as the "stochastic anti-pulsing" behaviour. (E) Sample simulated trajectories that were classified as the "homogeneous activation" behaviour. Source code used to generate this figure can be found in the provided GitLab repository.

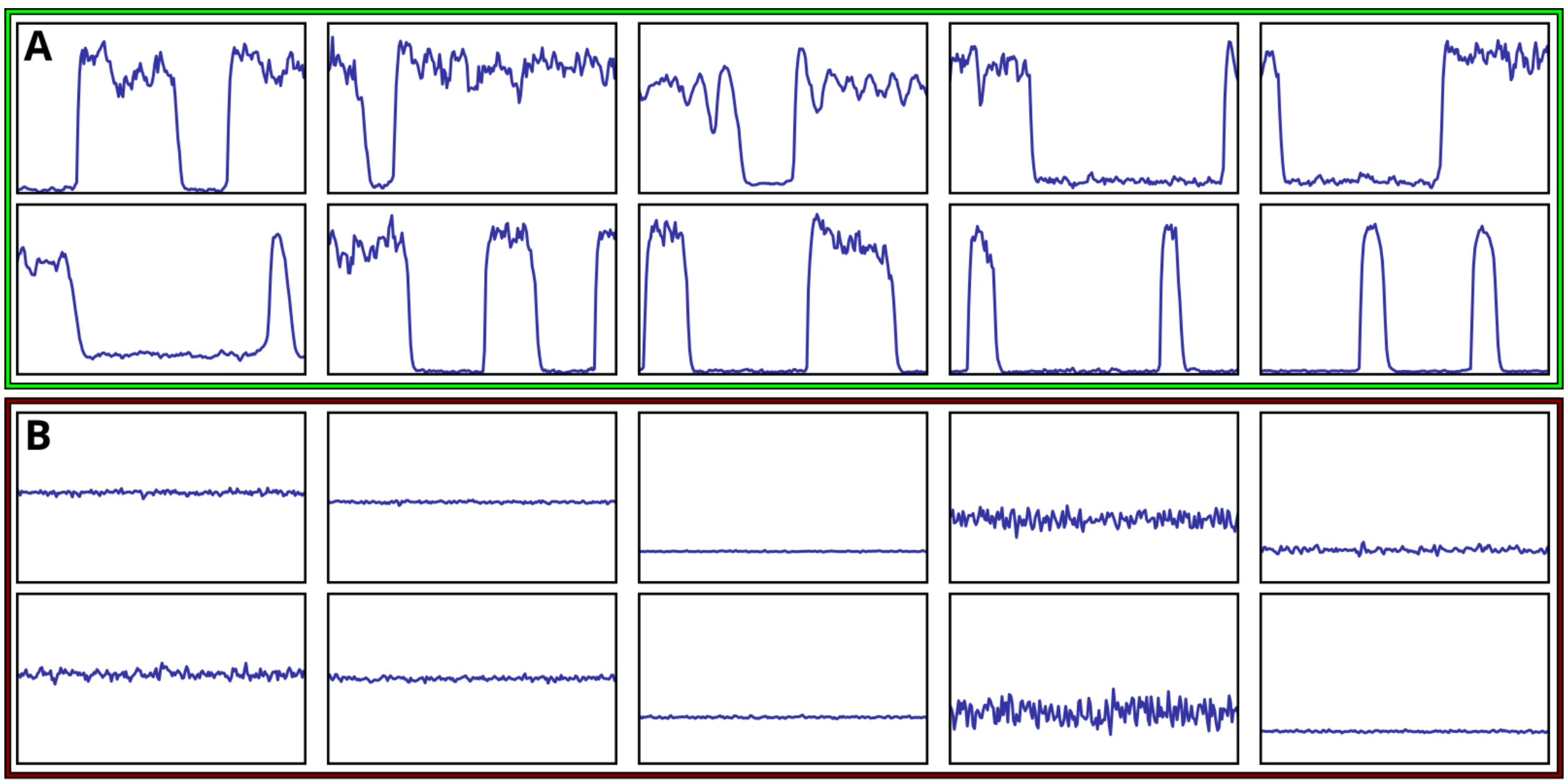

Supplementary Figure 45: **Example asymptotic model simulations and the generalised classifier's corresponding classification part 2.** For each relevant behaviour, 10 trajectories were randomly selected from those that were assigned to that behaviour. (A) Sample simulated trajectories that were classified as the "stochastic switching" behaviour. (B) Sample simulated trajectories that were classified as the "intermediate activation" behaviour. Source code used to generate this figure can be found in the provided GitLab repository.

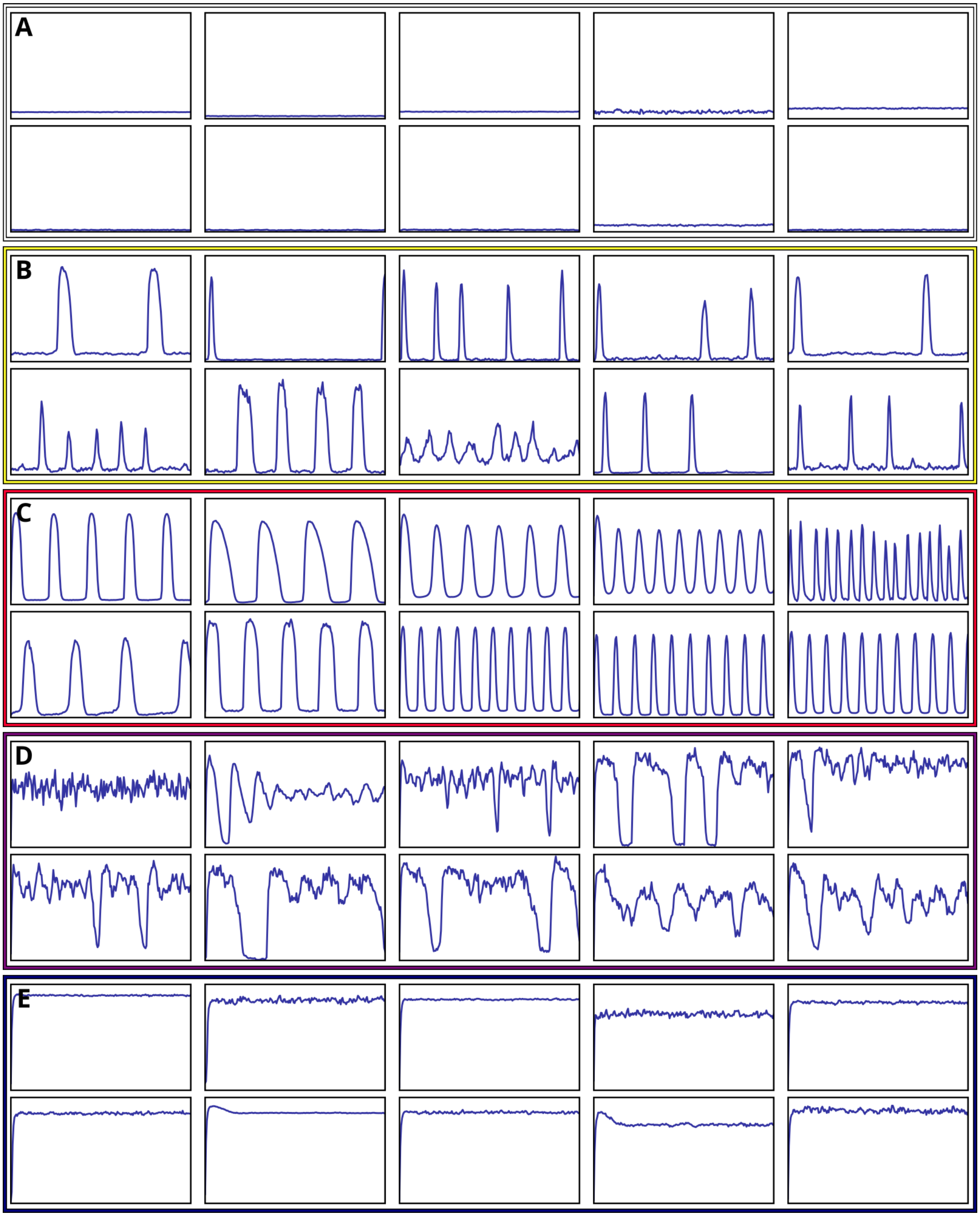

Supplementary Figure 46: **Example transient model simulations and the generalised classifier's corresponding classification part 1.** For each relevant behaviour, 10 trajectories were randomly selected from those that were assigned to that behaviour. (A) Sample simulated trajectories that were classified as the "no activation" behaviour. (B) Sample simulated trajectories that were classified as the "stochastic pulsing" behaviour. (C) Sample simulated trajectories that were classified as the "oscillation" behaviour. (D) Sample simulated trajectories that were classified as the "stochastic anti-pulsing" behaviour. (E) Sample simulated trajectories that were classified as the "homogeneous activation" behaviour. Source code used to generate this figure can be found in the provided GitLab repository.

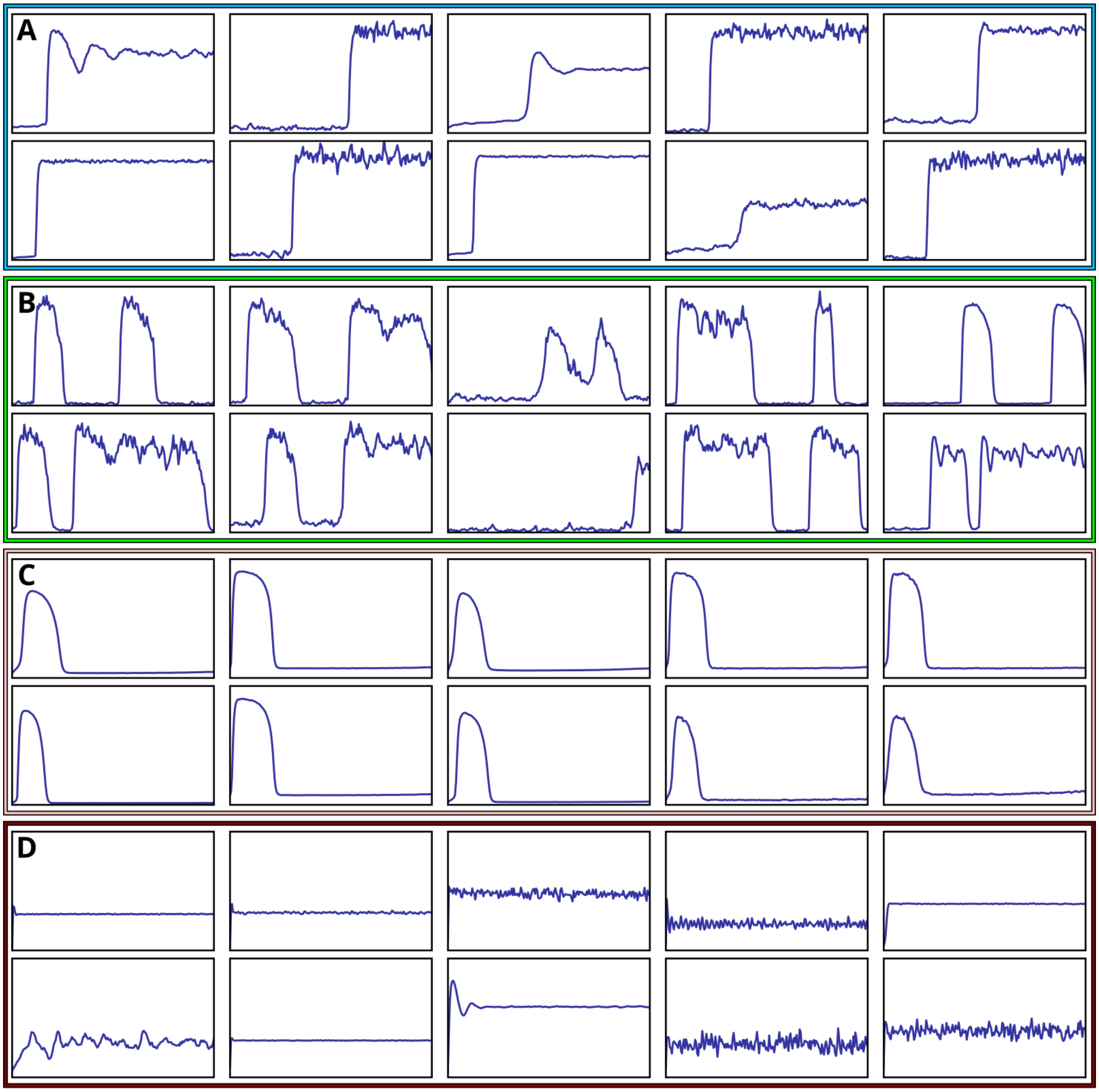

Supplementary Figure 47: **Example transient model simulations and the generalised classifier's corresponding classification part 2.** For each relevant behaviour, 10 trajectories were randomly selected from those that were assigned to that behaviour. (A) Sample simulated trajectories that were classified as the "heterogeneous activation" behaviour. (B) Sample simulated trajectories that were classified as the "stochastic switching" behaviour. (C) Sample simulated trajectories that were classified as the "single response pulse" behaviour. (D) Sample simulated trajectories that were classified as the "intermediate activation" behaviour. Source code used to generate this figure can be found in the provided GitLab repository.

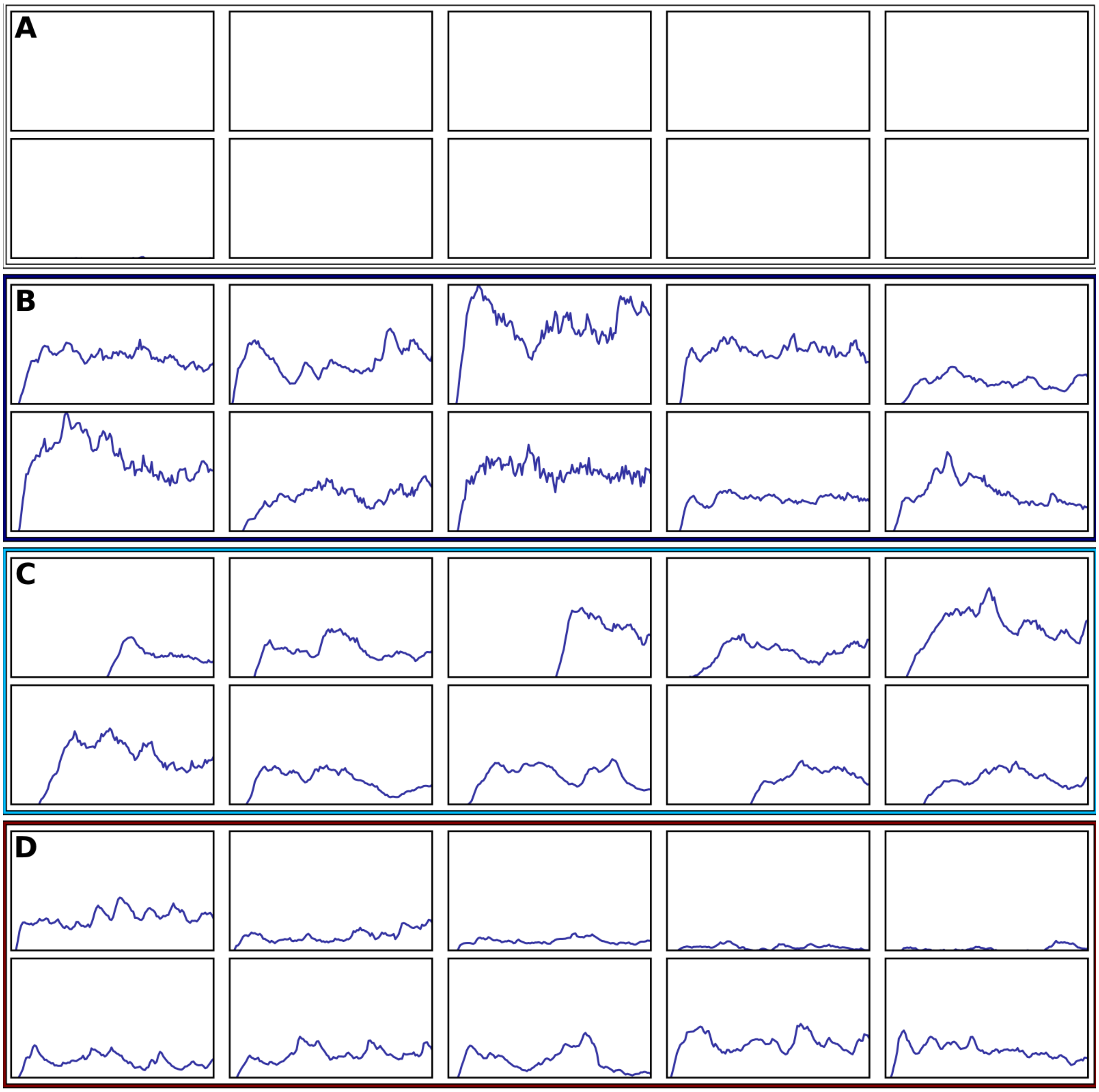

Supplementary Figure 48: **Example σ^V measured trajectories and the generalised classifier's corresponding classification.** For each relevant behaviour, 10 trajectories were randomly selected from those that were assigned to that behaviour. (A) Experimentally measured trajectories that were classified as the "no activation" behaviour. We note that a few trajectories exhibit low-amplitude initial responses. Likely, their activity levels were borderline to low for the trajectories to have been considered activated. (B) Experimentally measured trajectories that were classified as the "homogeneous activation" behaviour. (C) Experimentally measured trajectories that were classified as the "heterogeneous activation" behaviour. (D) Experimentally measured trajectories that were classified as the "intermediate activation" behaviour. Source data for this figure is available in the Source Data file.

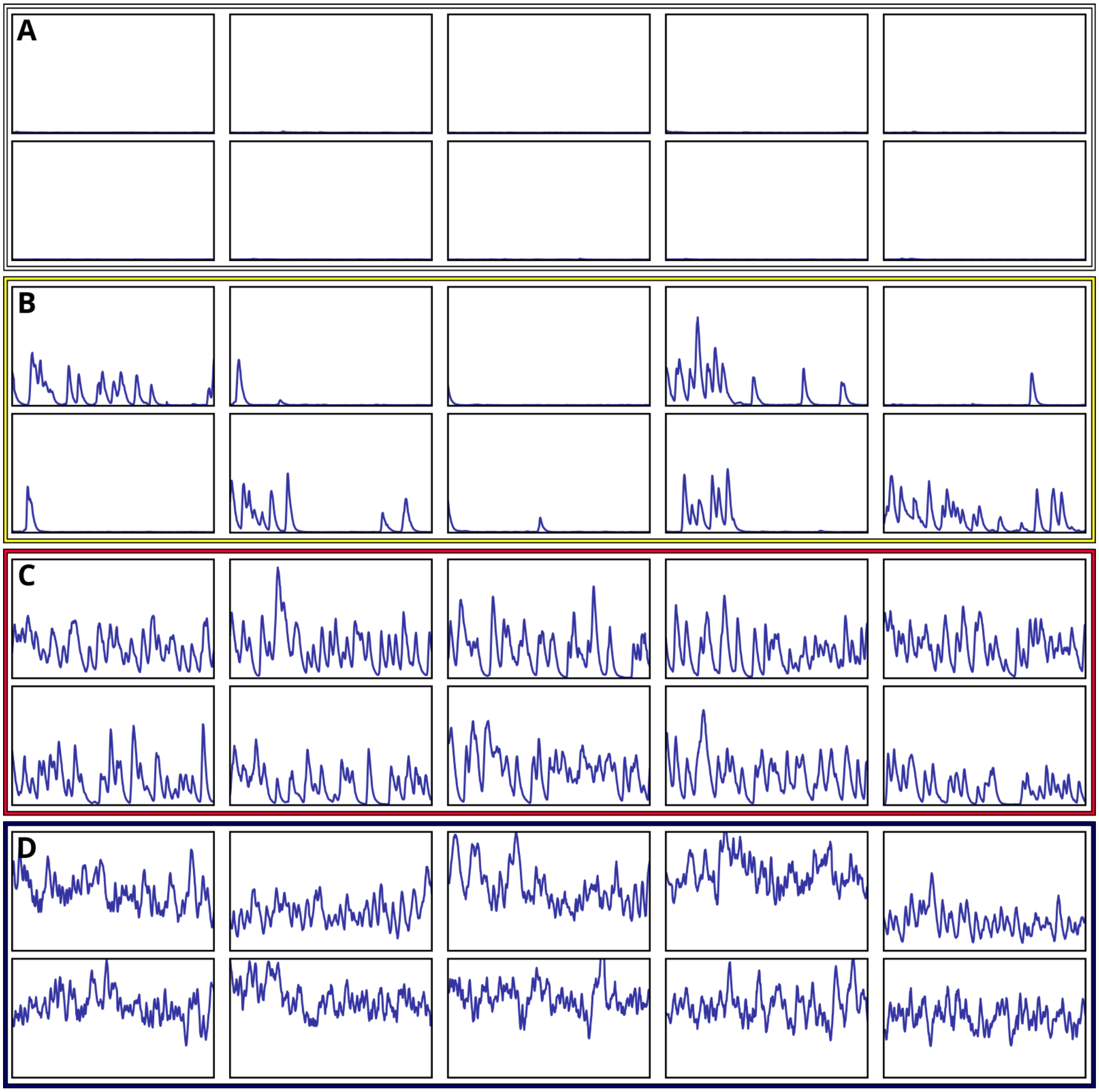

Supplementary Figure 49: **Example σ^B measured trajectories and the generalised classifier's corresponding classification.** For each relevant behaviour, 10 trajectories were randomly selected from those that were assigned to that behaviour. (A) Experimentally measured trajectories that were classified as the "no activation" behaviour. (B) Experimentally measured trajectories that were classified as the "stochastic pulsing" behaviour. (C) Experimentally measured trajectories that were classified as the "oscillation" behaviour. (D) Experimentally measured trajectories that were classified as the "homogeneous activation" behaviour. Source data for this figure is available in the Source Data file.

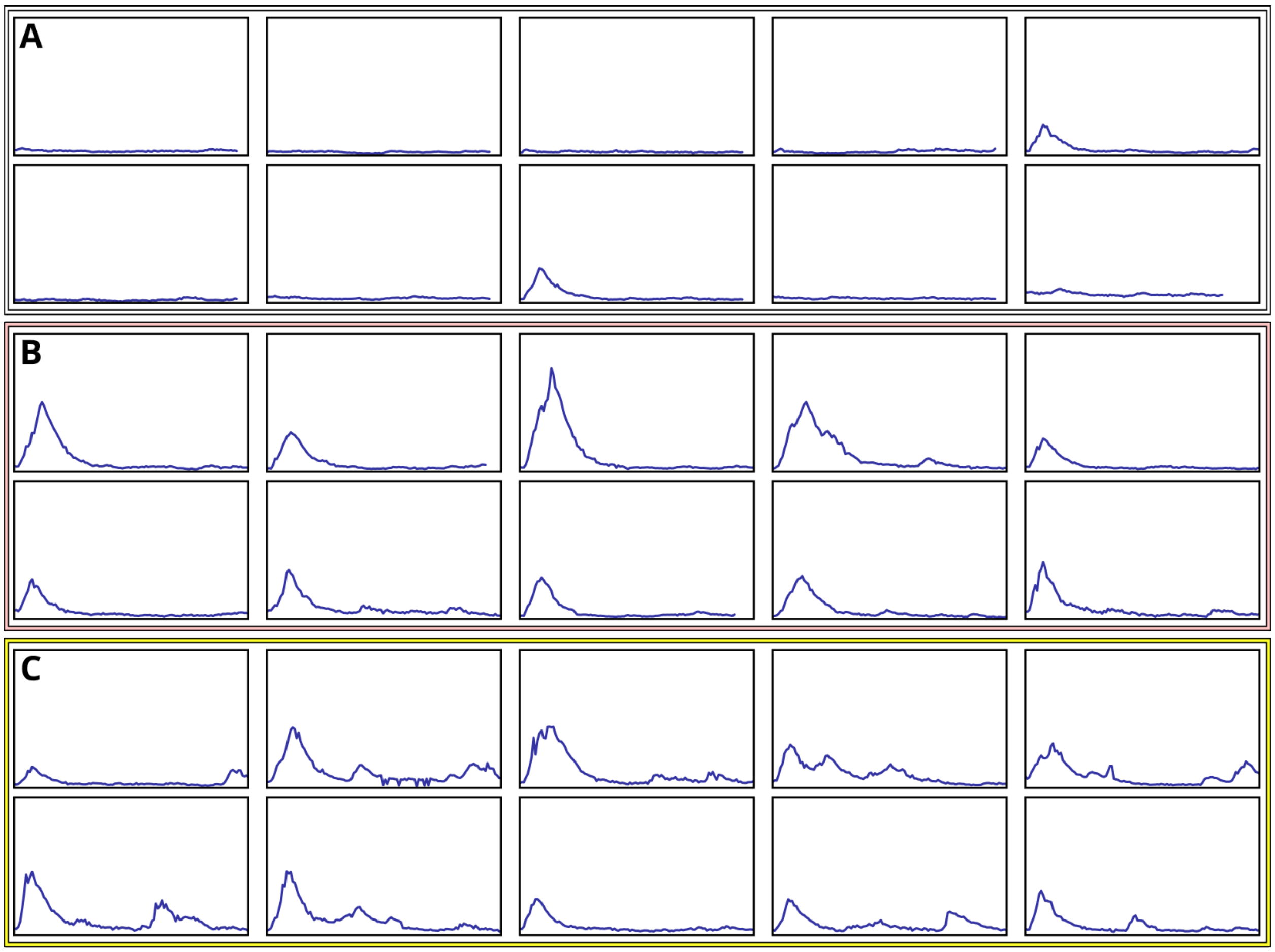

Supplementary Figure 50: **Example σ^{M} measured trajectories and the generalised classifier's corresponding classification.** For each relevant behaviour, 10 trajectories were randomly selected from those that were assigned to that behaviour. (A) Experimentally measured trajectories that were classified as the "no activation" behaviour. (B) Experimentally measured trajectories that were classified as the "single response pulse" behaviour. (C) Experimentally measured trajectories that were classified as the "stochastic pulsing" behaviour. Source data for this figure is available in the Source Data file.

Supplementary Note 13: Non-instantaneous S activation

Throughout this work, we have assumed that $S = 0$ before activation (at $t = 0$). After activation, it instantaneously assumes its full value. However, different inputs, where S (or $\hat{I}$ in the non-nondimensionalised model, Supplementary Note 2) varies with time, are possible. In this section, we will consider a single such alternative input case: where S assumes a ramp function (rather than a step function).

We will consider the input a function of time, $S_{in}(t)$, where S is its maximum value. Here:

$$S_{in}(t) = \begin{cases} 0, & t < 0, \\ t/S, & 0 \leq t \leq S, \\ 1, & t > S. \end{cases} \tag{1}$$

Once the ramp has reached its full value, the system should behave identically to the model where S is a step function. Hence, transient behaviours should be unchanged. Asymptotic behaviours, however, are generally dependent on the sudden onset of the input. As the input onset becomes less distinct (i.e. the ramp longer), these behaviours should be diminished. In Supplementary Figure 51 we consider three behaviours under input ramps of different lengths. Here, we confirm that asymptotic behaviours are diminished by slower input onsets, while transient behaviours remain. Noteworthy is the single pulse response. It exhibits an asymptotic pulse, but has a transient behaviour indistinguishable from no activation. Here, for a very slow input onset, no noticeable behaviour is encountered.

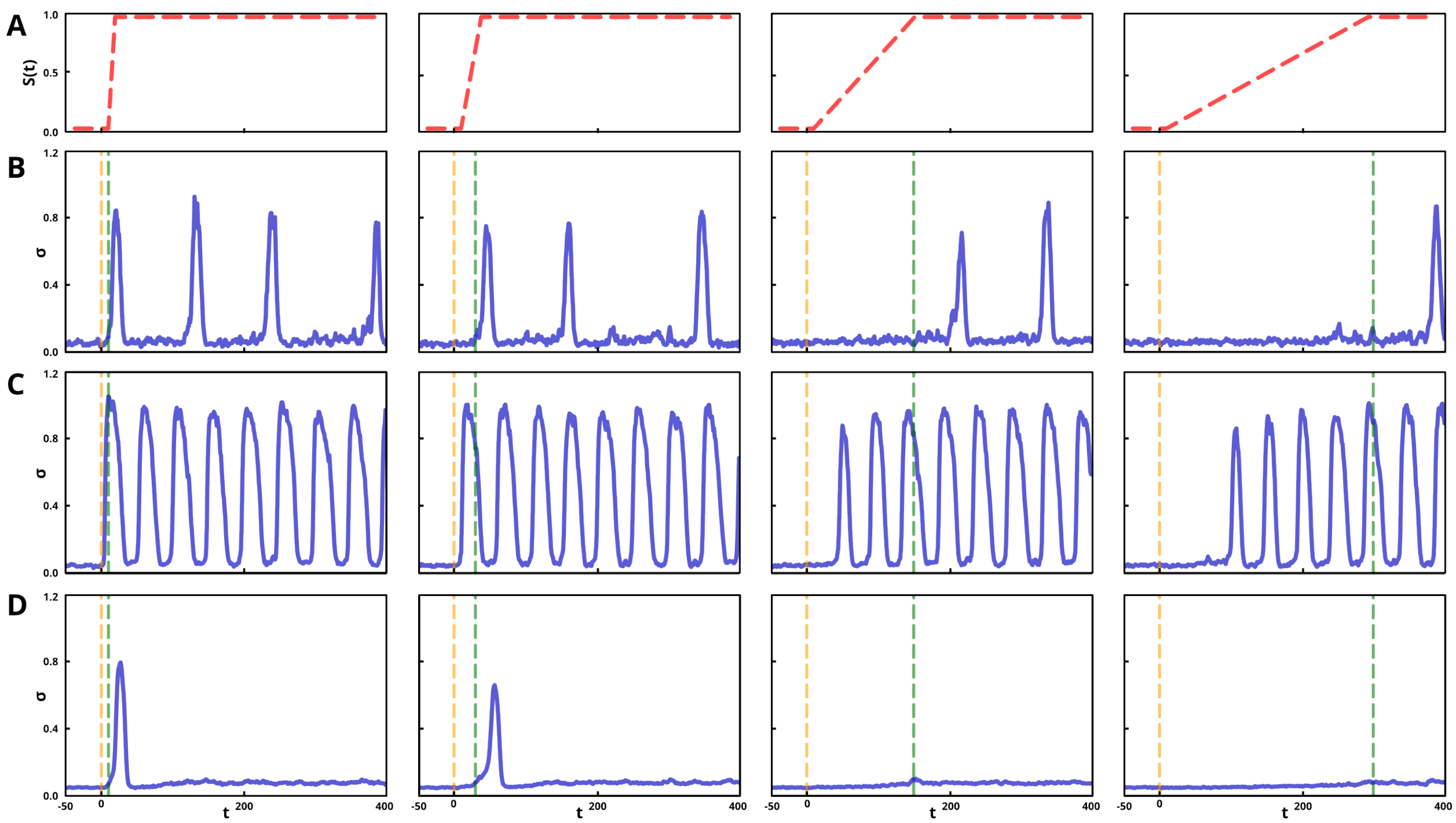

Supplementary Figure 51: **A ramp input diminishes asymptotic behaviours** (A) 4 different input ramps of lengths 10, 30, 150, and 300. The first ramp is very similar to a step input, while the last corresponds to a significant slowdown in input onset. In subfigures B-D, ramp initialisations are marked with yellow dashed lines and ramp endpoints with green dashed lines. (B) Stochastic pulsing in response to the different inputs. In all cases, the transient stochastic pulsing behaviour is exhibited. However, the presence of an initial activation pulse in response to the onset of the stress is diminished by slower input onsets (i.e. longer ramps). (C) Oscillations in response to the different inputs. Here, the oscillation appears smoothly with the onset of the input, reaching its maximum amplitude at the end of the ramp. (D) Single-pulse response in response to the different inputs. Here, as the input onset becomes more gradual, the initial response pulse diminishes. Since the behaviour's transient behaviour is identical to the no activation behaviour, for the longer ramps, no noticeable behaviour is detected. Source code used to generate this figure can be found in the provided GitLab repository.

Supplementary Note 14: Experimental set-up

14.1 Wafer fabrication

The microfluidic master was fabricated using maskless photolithography by exploiting a Dilase 650 Kloe direct laser writer (Kloe, France). The microfluidic design is based on the popular mother machine design (29) but allows for up to eight simultaneous conditions to be investigated. The devices were designed using AutoCad (Autodesk, USA). Briefly, for the first layer (growth channels) SU8 6001 (Kayaku Advanced Material, Japan) was spun coated (LabSpin, Suss, MicroTec,Germany) onto a silicon substrate (Si-Mat, Germany) at 1500 rpm. For the second layer (feeding channels) SU8 2050 (Kayaku Advanced Material, Japan) was spun at 2000 rpm.

Wafer cleaning

- The silicon substrate was cleaned in an acetone bath (Merck, USA) for 5 min.
- Then the wafer was rinsed with IPA (Merck, USA), blow dried with Nitrogen (BOC, UK) and dehydrated on a hot plate (Stuart, UK) at 200°C for 5 min.

Focus test

We found that doing a focus test on each wafer in the different areas where the final design would go before the actual exposure improved our results greatly. This was done before any exposure of the growth channels. Ramping the temperatures was critical to improve adhesion.

- SU8 6001 (Kayaku Advanced Material, Japan) was spun coated (LabSpin, Suss MicroTec, Germany) at 1500 rpm.
- Softbaking: 30 s 65°C → 4 min 110°C → 30 s 65°C.
- The exposure was done with a Dilase 650 direct laser writer (Kloe, France) using a beam size of 1 µm. Speed: 100 mm/s and 100% power.
- Post exposure bake: 30 s 65°C → 30 s 110°C → 30 s 65°C.
- The photoresist was then developed in PGMEA (Merck, USA) for 3 min.
- For each area of a final design the focus was noted. The results greatly depended on the focus and small changes in focus could make a big difference.
- Finally, the crosslinked SU8 was stripped off by wiping the wafer with acetone and cleaned again as described in the section on wafer cleaning.

Growth Channels

The parameters for the growth channel were the same as for the focus test except for a hard bake. Post exposure bake: 30 s 65°C → 30 s 110°C → 30 s 65°C. parameters result in a channel height of 1 µm.

- SU8 6001 (Kayaku Advanced Material, Japan) was spun coated (LabSpin, Suss MicroTec, Germany) at 1500 rpm.
- Softbaking: 30 s 65°C → 4 min 110°C → 30 s 65°C.
- The exposure was done with a Dilase 650 direct laser writer (Kloe, France) using a 1 µm beam size. Speed: 100 mm/s and 100% power.
- Post exposure bake: 30 s 65°C → 30 s 110°C → 30 s 65°C.
- Development in PGMEA for 3 min.
- Hard bake: 30 s 65°C → 30 s 110°C → 5 min 200°C → 30 s 110°C → 30 s 65°C.

Feeding Channels

The outlined parameters below resulted in a channel height of 80 µm.

- SU8 2050 (Kayaku Advanced Material, Japan) was spun coated (Polos Spin 150i, Netherlands) at 2000 rpm.
- Softbaking: 3 min 65°C → 9 min 95°C.
- The exposure was done with a Dilase 650 direct laser writer (Kloe, France) using a 10 µm beam. Speed: 100 mm/s and 25% power.
- Post exposure bake: 2 min 65°C → 7 min 95°C.
- Development in PGMEA for 7 min.

Supplementary Note 15: qPCR Primer Sequences

A list of all qPCR primers, and their sequences, used in this work is contained in Supplementary Table 9.

Supplementary Table 9: **List of qPCR primers.** This table lists all qPCR primers used in this work, as well as their sequences.

Primer name	Sequence	Gene
TS828	ATGCCGCGGTGAATACGTTCCC	16S Fw
TS829	TTCGGCGGCTGGCTCCTAAAAG	16S Rv
TS830	ACGCGTGATTGATGCCGGTGAC	rpoC Fw
TS831	AGCTGGTCGGTTGCCTTCAAGC	rpoC Rv
TS832	TGAGCGTTATCGTGTCCCGTGC	gyrA Fw
TS833	ACTTCTCCAACGATACGCGCGG	gyrA Rv
TS834	AAGCTTGGCTCTGCGGTGATCG	alaS Fw
TS835	TAACCAGCTTGCCGGCGTGAAG	alaS Rv
TS851	GCGCTTGACGAATTGCCGTACC	sigV Fw
TS852	AGGCGCGTTTTGACGGTATTCG	sigV Rv

Supplementary References

[1] Gillespie, D. T. The chemical Langevin equation. *Journal of Chemical Physics* **113**, 297–306 (2000).

[2] Gillespie, D. T. A General Method for Numerically Simulating the Stochastic Time Evolution of Coupled Chemical Reactions. *Journal of Computational Physics* **22**, 403–434 (1976).

[3] Gillespie, D. T. Exact stochastic simulation of coupled chemical reactions. *Journal of Physical Chemistry* **81**, 2340–2361 (1977).

[4] Cushing, J. *Lecture Notes in Biomathematics - Integrdifferential Equations and Delay Models in Population Dynamics* (Springer, 1977).

[5] Hu, S., Dunlavey, M., Guzy, S. & Teuscher, N. A distributed delay approach for modeling delayed outcomes in pharmacokinetics and pharmacodynamics studies. *Journal of Pharmacokinetics and Pharmacodynamics* **45**, 285–308 (2018). URL `https://doi.org/10.1007/s10928-018-9570-4`.

[6] Korsbo, N. & Jönsson, H. It's about time: Analysing simplifying assumptions for modelling multi-step pathways in systems biology. *PLoS Computational Biology* **16**, e1007982 (2020).

[7] Beretta, E. & Breda, D. Discrete or distributed delay? effects on stability of population growth. *Mathematical Biosciences and Engineering* **13**, 19–41 (2016).

[8] Fargue, D. Reductibilite des systemes hereditaires a des systemes dynamiques (regis par des equations differentielles aux derivees partielles). *C.R. Acad. Sci. Paris Sér. A-B* 471–473 (1973).

[9] MacDonald, N. *Lecture Notes in Biomathematics - Time Lags in Biological Models* (Springer, 1978).

[10] Metz, J. & Diekmann, . in *Exact Finite Dimensional Representations of Models for Physiologically Structured Populations.I: The Abstract Foundations of linear Chain Trickery* 269–289 (1991).

[11] Heinrich, R., Neel, B. G. & Rapoport, T. A. Mathematical models of protein kinase signal transduction. *Molecular Cell* **9**, 957–970 (2002).

[12] Veltz, R. BifurcationKit.jl. `https://hal.archives-ouvertes.fr/hal-02902346` (2020). Package version: 0.1.8.

[13] Rao, C. V., Wolf, D. M. & Arkin, A. P. Control, exploitation and tolerance of intracellular noise. *Nature* **420**, 231–237 (2002).

[14] Lindner, B., García-Ojalvo, J., Neiman, A. & Schimansky-Geier, L. Effects of noise in excitable systems (2004).

[15] Patange, O. *et al.* Escherichia coli can survive stress by noisy growth modulation. *Nature Communications* **9**, 5333 (2018).

[16] Park, J. *et al.* Molecular Time Sharing through Dynamic Pulsing in Single Cells. *Cell Systems* **6**, 216–229 (2018).

[17] Goldbeter, A. A minimal cascade model for the mitotic oscillator involving cyclin and cdc2 kinase. *Proceedings of the National Academy of Sciences of the United States of America* **88**, 9107–9111 (1991).

[18] Nelson, D. E. *et al.* Oscillations in NF-κB signaling control the dynamics of gene expression. *Science* **306**, 704–708 (2004).

[19] Friedman, N., Vardi, S., Ronen, M., Alon, U. & Stavans, J. Precise temporal modulation in the response of the SOS DNA repair network in individual bacteria. *PLoS Biology* **3**, e238 (2005).

[20] Bollinger, T. & Schibler, U. Circadian rhythms - From genes to physiology and disease. *Swiss Medical Weekly* **144**, 1–11 (2014).

[21] Schwall, C. P. *et al.* Tunable phenotypic variability through an autoregulatory alternative sigma factor circuit. *Molecular Systems Biology* **17**, 1–16 (2021).

[22] Weber, M. & Buceta, J. Dynamics of the quorum sensing switch: Stochastic and non-stationary effects. *BMC Systems Biology* **7**, 1–15 (2013).

[23] Uphoff, S. *et al.* Stochastic activation of a DNA damage response causes cell-to-cell mutation rate variation. *Science* **351**, 1094–1098 (2016).

[24] Acar, M., Mettetal, J. T. & Van Oudenaarden, A. Stochastic switching as a survival strategy in fluctuating environments. *Nature Genetics* **40**, 471–475 (2008).

[25] Sureka, K. *et al.* Positive feedback and noise activate the stringent response regulator rel in mycobacteria. *PLoS ONE* **3**, e1771 (2008).

[26] Choi, P. J., Cai, L., Frieda, K. & Xie, X. S. A Stochastic Single-Molecule Event Triggers Phenotype Switching of a Bacterial Cell. *Science* **322**, 442–446 (2008).

[27] Frigola, D., Casanellas, L., Sancho, J. M. & Ibañes, M. Asymmetric stochastic switching driven by intrinsic molecular noise. *PLoS ONE* **7**, e31407 (2012).

[28] Young, J. W., Locke, J. C. & Elowitz, M. B. Rate of environmental change determines stress response specificity. *Proceedings of the National Academy of Sciences of the United States of America* **110**, 4140–4145 (2013).

[29] Briat, C., Gupta, A. & Khammash, M. Antithetic Integral Feedback Ensures Robust Perfect Adaptation in Noisy Bimolecular Networks. *Cell Systems* **2**, 15–26 (2016). URL `http://dx.doi.org/10.1016/j.cels.2016.01.004`.

[30] Loman, T. E. & Locke, J. C. W. The σb alternative sigma factor circuit modulates noise to generate different types of pulsing dynamics. *PLOS Computational Biology* **19**, 1–31 (2023). URL `https://doi.org/10.1371/journal.pcbi.1011265`.

[31] Loman, T. E. Library of Basic Chemical Reaction Network Models · Catalyst.jl (2025). URL `https://docs.sciml.ai/Catalyst/stable/model_creation/examples/basic_CRN_library/`.

[32] Innes, M. *et al.* Fashionable modelling with flux. *CoRR* **abs/1811.01457** (2018). URL `https://arxiv.org/abs/1811.01457`.

[33] Blaom, A., Kiraly, F., Lienart, T. & Vollmer, S. alan-turing-institute/mlj.jl: v0.5.3 (2019). URL `https://doi.org/10.5281/zenodo.3541506`.
