# Supplementary material for: Mixed positive and negative feedback loops drive diverse single-cell gene expression dynamics
Source: Nat Commun. 2026 Aug 17;17:9855. doi: 10.1038/s41467-026-75107-4 (PMC13578229; doi:10.1038/s41467-026-75107-4)
Supplement: Supplementary file 2 [file 41467_2026_75107_MOESM2_ESM.pdf]

Description of Additional Supplementary Files

Supplementary Movie 1. Additional copies of *rsiV* and *sigV* shift induction behaviour from heterogeneous to intermediate activation.
B. subtilis cells were grown in a mother machine microfluidics device and imaged every 10 minutes. All strains constitutively expressed RFP for segmentation and carried a P*sigV*-YFP reporter. At time 0 h, cells were exposed to 1 µg/ml lysozyme stress. **Top:** Wild-type strain (JLB130). **Bottom:** Mutant strain containing ten copies of P*sigV-rsiV* and one copy of P*sigV-sigV* (JLB327). The fluorescence intensity was adjusted for visualisation.

Supplementary Movie 2. *The model's predicted transition through the pulsing/oscillatory region is validated in the σ^B system.*
B. subtilis cells were grown in a mother machine microfluidics device and imaged every 10 minutes. All shown channels are loaded with the same strain. This strain had constitutively expressed RFP and had a P*sigB*-YFP reporter. Additionally, the energy pathway (*rsbQP*) and environmental pathway (*rsbU*) were knocked out. The σ^B circuit was driven by IPTG inducible expression of a mutated RsbP that is constitutively active. IPTG was present from the start of the movie. From left to right, the movie shows no activation (0 µM), pulsing (4 µM), oscillation (5 µM), and homogeneous activation (8 µM). The fluorescence intensity was adjusted for visualisation.

Supplementary Data 1. Cloning primer sequences used in this study
