# Supplementary material for: Mixed positive and negative feedback loops drive diverse single-cell gene expression dynamics
Source: Nat Commun. 2026 Aug 17;17:9855. doi: 10.1038/s41467-026-75107-4 (PMC13578229; doi:10.1038/s41467-026-75107-4)
Supplement: Supplementary file 3 [file 41467_2026_75107_MOESM6_ESM.pdf]

nature portfolio

Corresponding author(s): James Locke (james.locke@slcu.cam.ac.uk)

Last updated by author(s): Dec 5, 2026

Reporting Summary

Nature Portfolio wishes to improve the reproducibility of the work that we publish. This form provides structure for consistency and transparency in reporting. For further information on Nature Portfolio policies, see our Editorial Policies and the Editorial Policy Checklist.

Statistics

For all statistical analyses, confirm that the following items are present in the figure legend, table legend, main text, or Methods section.

n/a	Confirmed	
☐	☒	The exact sample size (*n*) for each experimental group/condition, given as a discrete number and unit of measurement
☐	☒	A statement on whether measurements were taken from distinct samples or whether the same sample was measured repeatedly
☒	☐	The statistical test(s) used AND whether they are one- or two-sided<br>*Only common tests should be described solely by name; describe more complex techniques in the Methods section.*
☒	☐	A description of all covariates tested
☒	☐	A description of any assumptions or corrections, such as tests of normality and adjustment for multiple comparisons
☐	☒	A full description of the statistical parameters including central tendency (e.g. means) or other basic estimates (e.g. regression coefficient) AND variation (e.g. standard deviation) or associated estimates of uncertainty (e.g. confidence intervals)
☒	☐	For null hypothesis testing, the test statistic (e.g. *F*, *t*, *r*) with confidence intervals, effect sizes, degrees of freedom and *P* value noted<br>*Give P values as exact values whenever suitable.*
☒	☐	For Bayesian analysis, information on the choice of priors and Markov chain Monte Carlo settings
☒	☐	For hierarchical and complex designs, identification of the appropriate level for tests and full reporting of outcomes
☒	☐	Estimates of effect sizes (e.g. Cohen's *d*, Pearson's *r*), indicating how they were calculated

Our web collection on statistics for biologists contains articles on many of the points above.

Software and code

Policy information about availability of computer code

Data collection	1) The microscopes were controlled with Metamorph 7.10.5. 2) A version of Schnitzcells previously modified for Mother Machine experiments (Park et al. 2018 and Schwall et al. 2021) was used to analyse all mother machines experiments.
Data analysis	Extracted time trajectories are analyzed using Julia version 1.10+ using in-built functions and custom-made scripts. Mathematical model was simulated in Julia using the Catalyst.jl package. A full list of dependent packages, as well scripts required to generate figures in paper, can be found at https://gitlab.developers.cam.ac.uk/slcu/teamjl/loman_schwall_etal_2026

For manuscripts utilizing custom algorithms or software that are central to the research but not yet described in published literature, software must be made available to editors and reviewers. We strongly encourage code deposition in a community repository (e.g. GitHub). See the Nature Portfolio guidelines for submitting code & software for further information.

Data

Policy information about availability of data

All manuscripts must include a data availability statement. This statement should provide the following information, where applicable:

- Accession codes, unique identifiers, or web links for publicly available datasets
- A description of any restrictions on data availability
- For clinical datasets or third party data, please ensure that the statement adheres to our policy

Data availability

All data used in this study are available at: https://gitlab.developers.cam.ac.uk/slcu/teamjl/loman_schwall_etal_2026 (86). Repository README files describe the repository structure.
Code availability
All code for carrying out all analysis and the generation of all figures, can be found here: https://gitlab.developers.cam.ac.uk/slcu/teamjl/loman_schwall_etal_2026 (86). Repository README files and code comments direct users on its use. Julia's default package manager can be used to reproduce and install the exact package environment used. The repository contains the 6d behaviour maps, which is available for download and further investigation (these can also be re-generated using the repository code). Furthermore, our neural network-based classifier of dynamic trajectories is available as a Julia package at: https://gitlab.developers.cam.ac.uk/slcu/teamjl/dynamictrajectoriesclassification (including documentation and installation instructions).

Research involving human participants, their data, or biological material

Policy information about studies with human participants or human data. See also policy information about sex, gender (identity/presentation), and sexual orientation and race, ethnicity and racism.

Reporting on sex and gender	N/A
Reporting on race, ethnicity, or other socially relevant groupings	N/A
Population characteristics	N/A
Recruitment	N/A
Ethics oversight	N/A

Note that full information on the approval of the study protocol must also be provided in the manuscript.

Field-specific reporting

Please select the one below that is the best fit for your research. If you are not sure, read the appropriate sections before making your selection.

☒ Life sciences ☐ Behavioural & social sciences ☐ Ecological, evolutionary & environmental sciences

For a reference copy of the document with all sections, see nature.com/documents/nr-reporting-summary-flat.pdf

Life sciences study design

All studies must disclose on these points even when the disclosure is negative.

Sample size	No predetermined sample size was calculated. For all mother machine experiments, conditions were independently replicated 3–5 times. Individual experiments had between 19 and 100 continuously tracked single-cell channels per condition. These sample sizes were sufficient to identify and classify the dynamic behaviours observed in this study.
Data exclusions	All channels which did not have a surviving cell making it to the end of the movie were excluded from all further analysis.
Replication	All mother machine experiments were independently repeated at least three times and up to five times. qRT-PCR experiment was performed in two biologically independent repeats.
Randomization	Standard randomisation procedures were not applied. While allocation was not randomised, conditions were predefined, independently replicated, and analysed using quantitative pipelines, making allocation bias unlikely to affect results.
Blinding	No blinding was carried out. Experimental conditions were predefined and analysis relied on quantitative pipelines

Reporting for specific materials, systems and methods

We require information from authors about some types of materials, experimental systems and methods used in many studies. Here, indicate whether each material, system or method listed is relevant to your study. If you are not sure if a list item applies to your research, read the appropriate section before selecting a response.

Materials & experimental systems

n/a	Involved in the study
☒	☐ Antibodies
☒	☐ Eukaryotic cell lines
☒	☐ Palaeontology and archaeology
☒	☐ Animals and other organisms
☒	☐ Clinical data
☒	☐ Dual use research of concern
☒	☐ Plants

Methods

n/a	Involved in the study
☒	☐ ChIP-seq
☒	☐ Flow cytometry
☒	☐ MRI-based neuroimaging

Plants

Seed stocks	N/A
Novel plant genotypes	N/A
Authentication	N/A
