# Supplementary material for: Mixed positive and negative feedback loops drive diverse single-cell gene expression dynamics
Source: Nat Commun. 2026 Aug 17;17:9855. doi: 10.1038/s41467-026-75107-4 (PMC13578229; doi:10.1038/s41467-026-75107-4)
Supplement: Supplementary file 4 [file 41467_2026_75107_MOESM7_ESM.pdf]

nature portfolio

Peer Review File

Mixed positive and negative feedback loops drive diverse single-cell gene expression dynamics

Corresponding Author: Professor James Locke

This file contains all reviewer reports in order by version, followed by all author rebuttals in order by version.

Attachments originally included by the reviewers as part of their assessment can be found at the end of this file.

Version 0:

Reviewer comments:

Reviewer #1

(Remarks to the Author)
In this paper, Loman and collaborators perform a systematic analysis of the dynamical regimes exhibited by a small gene regulatory circuit. The circuit contains a positive feedback and a delayed negative feedback, and they use the alternativa sigma factor system of B subtilis as both an inspiration for the model and an experimental validation vehicle for some of the modeling predictions. The specific circuit architecture studied here is ubiquitous in cell types across the tree of life, and as such the results are bound to be of interest to researchers attempting to understand a variety of life forms from a systems biology perspective. The highly comprehensive character of the modeling study, together with the rigorous use of experimental validation, make this paper in my opinion an excellent candidate for publication in Nature Communications. I have, however, a few comments that I would ask the authors to address before publication:

1.- In spite of the large literature already cited (which reflects the long history of research in this topic), some bibliography is either missing or inadequately (in my opinion) cited. In particular, references 9 and 10 are used to argue that stochasticity is beneficial. However, to my knowledge those references (at least reference 10) are general works on stochasticity in biology, but do not relate explicitly to the beneficial role of noise. For that purpose I would rather cite other articles such as DOI 10.1073/pnas.040411297, https://www.nature.com/articles/nature01546, and 10.1126/science.1178962. Additionally, previous work exists exploring experimentally parameter space in the manner done here, coincidentally also in B subtilis, see for instance DOI 10.1073/pnas.1216091110 and reference 20 cited in the manuscript (cited in the context of noise only, but which also contains a successful instance of parameter mapping, in particular regarding the induction of oscillatory behavior in genetic competence). I think those references should be cited appropriately.

2.- While I appreciate the approach established by the authors to classify the dynamics of the circuit in different classes, some aspects of this method are unclear to me. For instance, the method seems to rely on stable fixed points, but (apart from the fact that their model also has unstable fixed points, which is an academic matter here nevertheless, since those points will not appear in a simulation), some of the dynamical regimes, in particular oscillations, do not relate directly with such stable fixed points but with stable limit cycles, which do not correspond in general to continuous switching between two fixed points. I would ask the authors to clarify this point.

3.- As another instance of the lack of clarity of the method, the authors say in lines 212-214 that "The classifier uses a decision tree in which each node performs simulations initialised at the model's steady states and measures the transition times to other steady states." How are the oscillatory regimes identified using this approach?

4.- Also, regarding the behavior classification, the difference between regimes 5 and 10 (lines 172 and 185) is merely quantitative, and thus they could very well be the same regime. Why do the authors distinguish among the two?

5.- The authors claim that their approach offers general predictive scope by modelling effective properties of the circuit instead of circuit-specific topologies (lines 50-54). However, besides the fact that they also model a specific topology (whose ubiquity makes the paper of broad interest, as I said above), they also assume a specific form of the integration between the positive and negative feedback, among the many options available (see e.g. DOI 10.1016/j.gde.2005.02.007). Could the authors comment on their choice, and have they analyzed what happens with alternative integration choices? I understand

that their chosen model might be appropriate for alternative sigma factors, but if the authors want to claim generality (in the light of the different examples shown in Fig. 1b), they should also consider these other integration options.

6.- Figure 4 has many panels, and it would be easier to understand if only relevant information were shown. For instance, why is it necessary to show both cases D=15 and D=30 in panels f-l? They are qualitatively the same. The situation would change if the authors could show experiments (and numerics) for larger S, but a simpler alternative would be so show only one of the two values of D. Still, it's a pity that no bifurcations are traversed in this case.

I would also like to raise the following minor issues:

7.- In line 41 the authors talk about DNA *control* when citing reference 15, when in my opinion they should talk about DNA *repair*.

8.- In line 118 the authors talk of "stochastic interpretation", but I guess they mean "stochastic modelling strategy". The term "stochastic interpretation" has a very well defined meaning in the theory of stochastic processes (e.g. the Ito vs Stratonovich interpretations of an SDE).

9.- Even though the three different regimes shown in Fig 1c are for illustration purposes, the reader is left wondering at this point of the paper what are the different conditions that lead to the different regimes.

10.- References to 2d slices for different parameter pairs in the supplementary figures should be made in line 220 besides figure 3, where only one parameter pair is shown.

11.- The comparative discussion of the results obtained for D>1 and D<1 throughout the results section could be linked more strongly to the intuitive fact that large D corresponds to increasing the relative strength of the (delayed) negative feedback over the positive feedback, thereby leading to regimes such as oscillations and pulsing, which do not exist when the negative feedback is negligible with respect to the positive feedback (D<1). This is an easy intuitive explanation that is mostly missing from the text.

12.- In line 243 the authors say that "all behaviours in the D<1 grew less prevalent as v0 grew larger...". How can that be? Some behaviour should be prevalent, or all of them equally so.

13.- In line 297 the authors say: "Our model predicts that the D<1 and D>1 regions coexists (sic, note verbal tense) within the same system...". But "coexistence" of dynamical regimes usually refers to regimes that exist for the same parameter values, and here D changes. I would not call this "coexistence".

14.- It's unclear (to me) what Suppl. Movie 2 shows (the absence of a caption doesn't help).

(Remarks on code availability)
I have not run the code, but a README file is provided, and the scripts seem to be standard enough to be usable by the community.

Reviewer #2

(Remarks to the Author)
(see our comments in attached pdf file)

(Remarks on code availability)

Reviewer #3

(Remarks to the Author)
The authors present a theoretical and experimental analysis of a simple genetic circuit in bacteria cosnisting of a positive and negative feedback loops. The authors provide a comprehensive exploration of the dynamic behaviors such circuits can generate using a delayed stocatistic differential equation model and supported by detailed modeling and experimnetal validation in B subtilis using "in vivo" sigma/nati-sigma systems.
My main concern regards the level of originality of the findings. Indeed, the core circuit studied here is a very well-known topology, essentially an instance of the amplified negative feedback oscillator (a motif described extensively in the literature, including in reviews published over 15 years ago (e.g. https://royalsocietypublishing.org/doi/full/10.1098/rsif.2010.0183).
While the mapping of behaviors across parameter space is interesting, the conceptual framework is not original. The behaviors demonstrated are those already known to arise from such network motifs. Indeed, when the negative feedback is weaker, bistability is expected. When the negative feedback gets stronger and delayed oscillations are expected, and intermediate behaviors are also expected. So, despite the thorough work, there is not enough novelty in my opinion.

(Remarks on code availability)

Reviewer #4

(Remarks to the Author)
I co-reviewed this manuscript with one of the reviewers who provided the listed reports. This is part of the Nature Communications initiative to facilitate training in peer review and to provide appropriate recognition for Early Career Researchers who co-review manuscripts.

(Remarks on code availability)

Version 1:

Reviewer comments:

Reviewer #1

(Remarks to the Author)
The authors have satisfactorily addressed, in my opinion, the reviewers' comments.

(Remarks on code availability)
I have not run the code, but there is an extensive set of readme files, with installation instructions where appropriate. The documentation seems thorough.

Reviewer #2

(Remarks to the Author)
The authors addressed all our comments satisfactorily, and we recommend accepting the manuscript for publication.

(Remarks on code availability)

Open Access This Peer Review File is licensed under a Creative Commons Attribution 4.0 International License, which permits use, sharing, adaptation, distribution and reproduction in any medium or format, as long as you give appropriate credit to the original author(s) and the source, provide a link to the Creative Commons license, and indicate if changes were made.
In cases where reviewers are anonymous, credit should be given to 'Anonymous Referee' and the source.
The images or other third party material in this Peer Review File are included in the article's Creative Commons license, unless indicated otherwise in a credit line to the material. If material is not included in the article's Creative Commons license and your intended use is not permitted by statutory regulation or exceeds the permitted use, you will need to obtain permission directly from the copyright holder.

To view a copy of this license, visit https://creativecommons.org/licenses/by/4.0/

The authors study the small gene circuit known as positive/negative feedback loop. They present a minimal, one-species model that captures key dynamical features of the system while minimizing complexity and the number of parameters. The dynamic output is categorized into distinct classes. To validate the model predictions, the authors use *Bacillus subtilis* sigma factor circuits for a selection of dynamic behaviours and their transitions.

The manuscript is carefully constructed and well written. The simulations are comprehensive and logically structured and of high quality.

The major limitation, however, is that the behavioral classifications are somewhat arbitrary, and the compartmentalization of behaviors remains heuristic. This issue is understandable, given the stochastic nature of the system and the lack of a rigorous method for categorizing such dynamics.

Below are several suggestions and comments for improvement. Many reflect personal preferences and are offered for the authors' consideration. The questions are intended to reflect curiosity and opportunities for further strengthening the manuscript. These suggestions are not essential for publication, but they may enhance clarity, rigor, and interpretability for a broader audience.

- A different classifier was used to analyze the simulations and experimental data, and the authors demonstrate that both yield consistent results. However, the experimental classifier identifies only 4 out of the 10 behavior classes. Would it be possible to extend this classifier to encompass all 10 classes and use it across both simulations and experiments for consistency? Additionally, some of the class definitions appear arbitrary. For instance, the distinction between "homogeneous" and "heterogeneous" activation is applied to high activation states but not to intermediate ones. (For example, in Figure 4d, there appears to be a continuous transition from heterogeneous to intermediate activation. The nonzero activation time persists throughout this transition, yet the distinction between heterogeneous and homogeneous activation is only applied at high activation levels, not for intermediate cases.) Simplifying the classification scheme (such as replacing "Homogeneous" and "Heterogeneous" Activation with a unified "Full/High Activation" category) and introducing a continuous color-coded scale in the behavior maps (e.g., mapping activation time to color intensity) could improve both the interpretability and the informational value of the results.

- The authors could stronger highlight the influence of stochasticity, which introduces novel behaviors absent in the deterministic case. For example, the third row of Supplementary Figure 28 powerfully illustrates how noise gives rise to new dynamical regions. This result is currently underemphasized, yet it may represent one of the most compelling findings of the study.

Minor comments and technical corrections:

- Line 151: It is stated that the trajectory approaches one of the fixed points (disregarding noise). This is not true in the case of a stable limit cycle.

- Line 163: The list could be labeled with letters for easier reference to the panels in Figure 2.

- Figure 3 (and multiple supporting figures): some of the legend boxes are hard to read to due the colored background (e.g. oscillations and stochastic switching). Consider only coloring the lines of the boxes, rather than filling them with color.

- Figure 5: caption a) is incomplete. It only describes one transition. caption c) "increasing IPTG concentration". It depends if one reads the figure top down or bottom up. Maybe replace with "changing IPTG concentration"? It

took me a while to understand the color code of the boxes in b) and c). Maybe it would be easier to understand with a color scheme according the regime, rather than the points?

- Consider briefly comparing heterogeneous activation to clock reactions.

- In the SI, line 110 contains an extra bracket: $[[\hat{\sigma}]$. Lines 114 and 119 omit the hat from $\hat{\sigma}$. Lines 128–132 lack hats entirely, although these parameters have dimensions. Also, in lines 130–132, k_{deg} is replaced by d. Moreover, the differential symbol d in the equations should be typeset upright (roman font), not italic. Line 136 replaces $\hat{I}$ with $\hat{\imath}$.

- The original dimensional model contains 9 parameters, 2 fundamental units, and 2 redundant parameters. Upon nondimensionalization and removal of these redundancies (e.g., $\hat{I}$ and $\hat{K}$), the system could be reduced to 5 independent dimensionless parameters. However, the final model includes 6, due to the undocumented introduction of a noise strength parameter η. According to line 136, η is defined as $\eta = 1/\sqrt{\hat{v}}$, which raises concerns: if this relationship holds, then varying η would implicitly alter other parameters involving $\hat{v}$, compromising their independence. Furthermore, this definition makes the "dimensionless" model not truly dimensionless, as it introduces parameters that retain physical units: $[\eta] = \sqrt{\text{min}/\mu\text{M}}$ and $[W_i] = \sqrt{\mu\text{M}/\text{min}}$. A more consistent approach might be to define $W_i = \hat{k}_{deg}\hat{W}_i/\sqrt{\hat{v}}$ and introduce η in the dimensional equations, maintaining dimensional consistency without introducing an artificial parameter.

- It would be helpful to include axis scales (e.g., labeled x- and y-axes) in all figures, including Supplementary Figures 5, 9, 15, 16, and 27–32, to improve clarity and interpretability.

- Supplementary Figure 7: Adding DDE simulation results for comparison, alongside the LCT, would strengthen the figure.

- Supplementary Section 5: Consider adding a symbol to denote steady-state concentrations.

- Table captions should appear above the tables.

- In discussing the results: "For large values of η, the behaviour can also occur in the ($D \lesssim 1$) region, but this is likely rather a result of a very noisy system occasionally passing the threshold to being active, rather than a genuine pulsing behavior (Supplementary Figure 28)." — The mechanism behind the so-called "genuine pulsing" behavior is not entirely clear. It appears to be similar to the case where the system occasionally crosses the activation threshold due to noise. As far as I can tell, the main difference lies in the height of the threshold, making such crossings less frequent, rather than in the underlying mechanism. Is there a qualitative distinction or fundamentally different process that separates this from what is described as "genuine pulsing"?

- In the behavior occurrence analysis, normalization was performed by dividing by the maximum value. Would it not be more appropriate to normalize by the sum of occurrences or by the total number of simulations? For example, if all 10 classes occurred with equal frequency across all parameters, this method would assign a normalized value of 1 to each—despite each appearing in only 10% of cases. If this interpretation is correct, it seems inconsistent with the expectation of low occurrence values for rarely observed classes. Additionally, it would be helpful to specify the parameter ranges used in the occurrence plots, the total number of simulations performed, and how many times each point in the parameter space was evaluated. These details were also unclear in the behavior maps.

- Supplementary Figure 27: Adding both stable and unstable periodic orbits to the bifurcation diagrams would aid interpretation. It is not obvious why the bifurcation diagrams for $D = 1$ and $D = 5$ appear qualitatively similar, yet

their associated regions differ in the behavior maps. Conversely, between $D = 5$ and $D = 25$, a qualitative change in the bifurcation structure occurs, but the behavior map remains unchanged. Is this because the stochastic dynamics cause non-oscillatory regions to exhibit noise-induced oscillations, thereby widening the apparent oscillatory domain?

- The self-activation parameter S was altered using a Heaviside step function to represent a sudden change in the input signal, resulting in a strong perturbation of the system. However, in many natural systems, input signals change more gradually rather than instantaneously. It would therefore be valuable to investigate how the system responds to smoother or more realistic input profiles, particularly in cases where the model yields a sharp, single-peak response resembling a Dirac delta function.
- If multiple copies of a gene were inserted, how were they arranged? Facing in the same direction? As operon? Did you not observe recombination? Was the copy number stable? Related to this, in Table 3, the constructs are named for example "lacA::5xPsigV-rsiV NatR" while in the plasmid list it is "lacA::PsigV-5x rsiV NatR", suggesting that the promoter is only inserted once. Please extend the description of these multi-copy constructs.
- line 664 "Ellowitz" should be "Elowitz"
- Supplementary information pages 61/62: on several occasions it is written "um" instead of "μm"
- With exception for some of the data in Fig. 4 and Fig. 5, the experimental system is mainly in the supporting information. The manuscript could be made more attractive to experimentalists if more illustrations of the experimental system would be in the main manuscript. For example snapshots of the mother machine and a small schemes of the sigma factor systems, so it is visually immediately clear which system (σ V vs B) was used and what parameters were varied (copy number, lysozyme concentrations)
- line 404: what is the (literature) half-life of your fluorescent marker? Does it contain a degradation tag?

Response to reviewers

We thank the reviewers for their careful consideration and helpful suggestions for our manuscript. We have substantially revised the manuscript based on their points. Below, we provide a point by point response to their concerns. Our responses are in **bold**.

Reviewer #1 (Remarks to the Author)

In this paper, Loman and collaborators perform a systematic analysis of the dynamical regimes exhibited by a small gene regulatory circuit. The circuit contains a positive feedback and a delayed negative feedback, and they use the alternativa sigma factor system of B subtilis as both an inspiration for the model and an experimental validation vehicle for some of the modeling predictions. The specific circuit architecture studied here is ubiquitous in cell types across the tree of life, and as such the results are bound to be of interest to researchers attempting to understand a variety of life forms from a systems biology perspective. The highly comprehensive character of the modeling study, together with the rigorous use of experimental validation, make this paper in my opinion an excellent candidate for publication in Nature Communications. I have, however, a few comments that I would ask the authors to address before publication:

We thank the reviewer for their positive assessment of the manuscript. We agree with their comments and have addressed them, as outlined below:

1.- In spite of the large literature already cited (which reflects the long history of research in this topic), some bibliography is either missing or inadequately (in my opinion) cited. In particular, references 9 and 10 are used to argue that stochasticity is beneficial. However, to my knowledge those references (at least reference 10) are general works on stochasticity in biology, but do not relate explicitly to the beneficial role of noise. For that purpose I would rather cite other articles such as DOI 10.1073/pnas.040411297, https://www.nature.com/articles/nature01546, and 10.1126/science.1178962. Additionally, previous work exists exploring experimentally parameter space in the manner done here, coincidentally also in B subtilis, see for instance DOI 10.1073/pnas.1216091110 and reference 20 cited in the manuscript (cited in the context of noise only, but which also contains a successful instance of parameter mapping, in particular regarding the induction of oscillatory behavior in genetic competence). I think those references should be cited appropriately.

We agree with the reviewer. In the original version, refs 9 and 10 were inadvertently cited in support of the beneficial role of noise; we have corrected this so that refs. 9 and 10 now support the general point that stochasticity modulates single-cell dynamics, while the functional role of noise is supported by the studies suggested by the reviewer. In addition, we now cite experimental precedents for in vivo exploration of dynamical regimes by varying circuit inputs/parameters, including the *B. subtilis* competence study

(DOI: 10.1073/pnas.1216091110) and ref. 20, in the appropriate context in the Introduction (line 68).

2.- While I appreciate the approach established by the authors to classify the dynamics of the circuit in different classes, some aspects of this method are unclear to me. For instance, the method seems to rely on stable fixed points, but (apart from the fact that their model also has unstable fixed points, which is an academic matter here nevertheless, since those points will not appear in a simulation), some of the dynamical regimes, in particular oscillations, do not relate directly with such stable fixed points but with stable limit cycles, which do not correspond in general to continuous switching between two fixed points. I would ask the authors to clarify this point.

We agree with the reviewer that oscillations correspond to stable limit cycles, rather than switching between two stable fixed points, and have improved our description in the text to make this clear. We utilise steady state analysis to classify behaviours (or, since we are analysing a stochastic model, the steady state distributions, with the relation between deterministic and stochastic model discussed extensively in the supplementary material). However, we do not solely rely on it. Oscillations, as the reviewer points out, are a primary example of this. For the deterministic case, these coincide with cases where the system's single steady state is unstable. However, since they also can occur in a system with a combination of stable and unstable steady states, and because conclusions of deterministic parameter sets do not fully translate to stochastic ones (as discussed in new Figure 4 Supplementary Figure 36), we cannot fully rely on this. Here, our classifier also utilises Fourier analysis of simulated trajectories to e.g. distinguish oscillations from other behaviours. The full classifier is described in the supplementary, with the main text only providing a briefer summary. We have updated the main text and methods to better clarify the existence of additional considerations within the classifier, such as this (lines 166-172).

3.- As another instance of the lack of clarity of the method, the authors say in lines 212-214 that "The classifier uses a decision tree in which each node performs simulations initialised at the model's steady states and measures the transition times to other steady states." How are the oscillatory regimes identified using this approach?

Thanks for pointing this out. In the main text, we can only provide a short description to provide readers an idea of the components of the classifier, with an exhaustive description being provided in the Supplementary. As described above, Fourier analysis is used to detect oscillations, which, as the reviewer accurately points out, do not correspond to transitions between fixed points. We have modified the main text to clarify the oscillation case and have added that the description of the classifier is non-exhaustive (Lines 233-234 and 666-671).

4.- Also, regarding the behavior classification, the difference between regimes 5 and 10 (lines

172 and 185) is merely quantitative, and thus they could very well be the same regime. Why do the authors distinguish among the two?

There is a qualitative difference, although we agree that this difference is by no means obvious from the brief description in the text. Mathematically, for both cases, the model has a single steady state; however, the intermediate activation behaviour's fixed point can transition to instability (and the behaviour to an oscillation) as tau is increased (Supplementary Figure 14J is helpful here). Such a transition does not exist for homogeneous activation. Furthermore, the intermediate activation behaviour's steady state levels exist between the inactive and active state thresholds as derived in section 7.2. Considering the behaviours themselves, the activity levels of the intermediate activation behaviours are smoothly tuned by S and D (increasing with S and decreasing with D). The homogeneous activation behaviour's levels, on the other hand, are robust to modulations of S and D (where no real change is achieved unless S is reduced/D decreased enough to produce a change to another behaviour). Indeed, this is important for Figure 5, where we demonstrate that SigV (in accordance with the model prediction) has entered the intermediate activation behaviour by showing that the steady state levels are tuned by modulations by S and D.

We have updated the main text to clarify this, especially regarding new Figure 6 (lines 205-209, 387-390, 398-418). We have also added a new paragraph in the supplementary specifically discussing this in detail (Supplementary lines 970-977).

5.- The authors claim that their approach offers general predictive scope by modelling effective properties of the circuit instead of circuit-specific topologies (lines 50-54). However, besides the fact that they also model a specific topology (whose ubiquity makes the paper of broad interest, as I said above), they also assume a specific form of the integration between the positive and negative feedback, among the many options available (see e.g. DOI 10.1016/j.gde.2005.02.007). Could the authors comment on their choice, and have they analyzed what happens with alternative integration choices? I understand that their chosen model might be appropriate for alternative sigma factors, but if the authors want to claim generality (in the light of the different examples shown in Fig. 1b), they should also consider these other integration options.

This is a good point. To test whether our conclusions are robust to alternative choices for integrating positive and negative feedback, we have added a new Supplementary Section (5) and two Supplementary Figures (11 and 12). Here, we consider two alternative implementations of a mixed feedback loop model (one using an alternative but still fairly standard Hill-based logic, one less conventional one where A3 directly subtracts from the sigma activity in the production reaction. We show that both of these models reproduce the full set of ten dynamic behaviours identified in our original framework.

6.- Figure 4 has many panels, and it would be easier to understand if only relevant information were shown. For instance, why is it necessary to show both cases D=15 and D=30 in panels f-I? They are qualitatively the same. The situation would change if the authors could show experiments (and numerics) for larger S, but a simpler alternative would be so show only one of the two values of D. Still, it's a pity that no bifurcations are traversed in this case.

We agree that panels f–I for D = 15 and D = 30 look qualitatively similar. We nevertheless include both values, together with the corresponding experiments, because this comparison is essential to show that activity levels are tuned by D and not only by S, supporting our identification of the intermediate activation behaviour predicted by the model. We have updated the text to make this motivation clearer (lines 387-390, 398-418).

We also agree that the original Figure 4 contained too many panels and was difficult to follow. To address this, we have split it into two figures and adjusted the main text accordingly. In addition, as proposed by reviewer 2, we have added representative mother machine images to Figure 4 to make the experimental validation clearer. Finally, while it would indeed be interesting to directly traverse a clear deterministic bifurcation experimentally, here our goal was to test parameter-dependent tuning and behaviour changes in the experimentally accessible range.

I would also like to raise the following minor issues:

7.- In line 41 the authors talk about DNA *control* when citing reference 15, when in my opinion they should talk about DNA *repair*.

We have updated the text to instead mention DNA repair (line 44).

8.- In line 118 the authors talk of "stochastic interpretation", but I guess they mean "stochastic modelling strategy". The term "stochastic interpretation" has a very well defined meaning in the theory of stochastic processes (e.g. the Ito vs Stratonovich interpretations of an SDE).

We used interpretation in reference to interpretations of chemical reaction networks (e.g. Chemical master equation versus Langevin approximations). However, we agree that this could be confused with the Ito/Stratonovich interpretation of an SDE, and have now updated the text to avoid ambiguity (lines 132-133).

9.- Even though the three different regimes shown in Fig 1c are for illustration purposes, the reader is left wondering at this point of the paper what are the different conditions that lead to the different regimes.

We have updated the figure with the parameter sets used to generate each behaviour.

10.- References to 2d slices for different parameter pairs in the supplementary figures should be made in line 220 besides figure 3, where only one parameter pair is shown.

We have added references to the supplementary figures as requested.

11.- The comparative discussion of the results obtained for D>1 and D<1 throughout the results section could be linked more strongly to the intuitive fact that large D corresponds to increasing the relative strength of the (delayed) negative feedback over the positive feedback, thereby leading to regimes such as oscillations and pulsing, which do not exist when the negative feedback is negligible with respect to the positive feedback (D<1). This is an easy intuitive explanation that is mostly missing from the text.

Thanks for this suggestion. We have updated the Results text to explicitly link D to the relative strength of delayed negative feedback versus self-activation, explaining why bistable behaviours dominate for D$\lesssim$1 and oscillatory/excitable behaviours emerge for D$\gtrsim$1 (lines 251-257).

12.- In line 243 the authors say that "all behaviours in the D<1 grew less prevalent as v0 grew larger...". How can that be? Some behaviour should be prevalent, or all of them equally so.

What we meant was the non-trivial behaviours, i.e. all behaviours except for "no activation" and "homogeneous activation" become less prevalent. I.e. as *v0* increases, these two behaviours become more prevalent, at the expense of all other behaviours. We agree that this is not clear, and we have updated the text to clarify this (lines 268-270).

13.- In line 297 the authors say: "Our model predicts that the D<1 and D>1 regions coexists (sic, note verbal tense) within the same system...". But "coexistence" of dynamical regimes usually refers to regimes that exist for the same parameter values, and here D changes. I would not call this "coexistence".

We agree and have revised the wording to avoid claiming "coexistence" (line 338).

14.- It's unclear (to me) what Suppl. Movie 2 shows (the absence of a caption doesn't help).

We note that a caption for Supplementary Movie 2 was included in the submitted Supplementary Information, but we agree it may be easy to miss. For clarity, we reproduce the movie captions here:

Movie 1. Additional copies of *rsiV* and *sigV* shift induction behaviour from heterogeneous to intermediate activation.
B. subtilis cells were grown in a mother machine microfluidics device and imaged every 10 minutes. All strains constitutively expressed RFP for segmentation and carried a P*sigV*-YFP reporter. At time 0 h, cells were exposed to 1 μg/ml lysozyme stress. **Top:** Wild-type strain (JLB130). **Bottom:** Mutant strain containing ten copies of P*sigV*-*rsiV* and one copy of P*sigV*-*sigV* (JLB327). The fluorescence intensity was adjusted for visualisation.

Movie 2. ***The model's predicted transition through the pulsing/oscillatory region is validated in the σ^B system.***
B. subtilis cells were grown in a mother machine microfluidics device and imaged every 10 minutes. All shown channels are loaded with the same strain. This strain had constitutively expressed RFP and had a P*sigB*-YFP reporter. Additionally, the energy pathway (*rsbQP*) and environmental pathway (*rsbU*) were knocked out. The σ^B circuit was driven by IPTG inducible expression of a mutated RsbP that is constitutively active. IPTG was present from the start of the movie. From left to right, the movie shows no activation (0 μM), pulsing (4 μM), oscillation (5 μM), and homogeneous activation (8 μM). The fluorescence intensity was adjusted for visualisation.

Reviewer #1 (Remarks on code availability)

I have not run the code, but a README file is provided, and the scripts seem to be standard enough to be usable by the community.

Reviewer #2 (Remarks to the Author)

The authors study the small gene circuit known as positive/negative feedback loop. They present a minimal, one-species model that captures key dynamical features of the system while minimizing complexity and the number of parameters. The dynamic output is categorized into distinct classes. To validate the model predictions, the authors use Bacillus subtilis sigma factor circuits for a selection of dynamic behaviours and their transitions.

The manuscript is carefully constructed and well written. The simulations are comprehensive and logically structured and of high quality. The major limitation, however, is that the behavioral classifications are somewhat arbitrary, and the compartmentalization of behaviors remains heuristic. This issue is understandable, given the stochastic nature of the system and the lack of a rigorous method for categorizing such dynamics.

Below are several suggestions and comments for improvement. Many reflect personal preferences and are offered for the authors' consideration. The questions are intended to reflect

curiosity and opportunities for further strengthening the manuscript. These suggestions are not essential for publication, but they may enhance clarity, rigor, and interpretability for a broader audience.

• A different classifier was used to analyze the simulations and experimental data, and the authors demonstrate that both yield consistent results. However, the experimental classifier identifies only 4 out of the 10 behavior classes. Would it be possible to extend this classifier to encompass all 10 classes and use it across both simulations and experiments for consistency?

Thank you for this interesting suggestion. The reason for the split in the classifier was that the classification problems are fundamentally slightly different. When classifying the behaviour of model parameter sets, we can generate an infinite number of simulations from across phase space, and can also utilise our knowledge of the model to e.g. compute steady states. While these are for the deterministic model, they still serve as good approximate guesses of the stochastic model's behaviours (considerations in comparing the deterministic and stochastic versions of the models are discussed extensively, especially in the Supplementary material, with Supplementary Figure 36 providing a good summary of some considerations and new Figure 4 giving a briefer summary). In practice, we use steady states as initial guesses when determining approximate steady state distributions (the high computational cost of the scans means that performance increases from guesses like this is important). The second classifier, which we used on models and simulations in Figure 6, instead works on single trajectories. To keep it as simple as possible, we limited it to the four behaviours encountered in the model with which we analysed it.

However, in response to the reviewers' feedback, we have fully reworked the trajectory-level analysis. We have now created a new trajectory-based classifier, which replaces the previous trajectory classifier used in Figure 7 (previously Figure 5). The new one is based on a neural network, and trained on a mix of simulated and experimentally measured trajectories from the σ^B and σ^V systems (Supplementary Section 12). Notably, it can classify all relevant behaviours (i.e. all behaviours except for stable bistability, which is fundamentally indistinguishable from no activation when only single trajectories are available). We validate the classifier both on simulated and experimental data (taking care not to validate on trajectories from a repeat that contains trajectories in the training set) (Supplementary Figures 44-49). As an additional test of generalisation, we applied the classifier to a new system (σ^M) measured using a P_{sigM}-YFP reporter in the mother machine across a range of bacitracin concentrations. Notably, the classifier could classify these trajectories despite never being trained on σ^M data and despite σ^M exhibiting single-response pulses that were present only in the simulated training set (Supplementary Figure 50).

Additionally, some of the class definitions appear arbitrary. For instance, the distinction between "homogeneous" and "heterogeneous" activation is applied to high activation states but not to intermediate ones. (For example, in Figure 4d, there appears to be a continuous transition from

heterogeneous to intermediate activation. The nonzero activation time persists throughout this transition, yet the distinction between heterogeneous and homogeneous activation is only applied at high activation levels, not for intermediate cases.) Simplifying the classification scheme (such as replacing ”Homogeneous” and ”Heterogeneous” Activation with a unified ”Full/High Activation” category) and introducing a continuous color-coded scale in the behavior maps (e.g., mapping activation time to color intensity) could improve both the interpretability and the informational value of the results.

In the supplementary, we discuss potential classes that can be distinguished from the 10 we present in the main paper, and our reasons for not considering them distinct classes in the paper. This includes a discussion on a potential heterogeneous intermediate activation. However, we agree we could highlight these discussions better in the main text, and have modified it to do so properly (lines 176-177).

At an earlier stage of the writing, we considered continuous colour scales as the reviewer described. Other scales which we also considered include the smooth transition between heterogeneous and homogeneous activation (as activation times get shorter) and from no activation to stochastic pulsing to oscillation (as pulsing goes from extremely infrequent to frequent, to so dense it can be considered an oscillation). Similarly, one could have an activation scale from no activation, through intermediate activation, to homogeneous activation. However, the behaviour maps are already describing a lot of information, and expanding them in this manner made them highly cluttered. They were also inaccessible to people with e.g. colour blindness. Hence, we decided on this approach using fully discrete categories. We do discuss in the paper the existence of such underlying gradients, and that the exact boundaries between behaviours cannot be determined exactly in a stochastic setting (i.e. while there are behaviours that are clearly “homogenous activation” and that are clearly “heterogeneous activation”, the exact position of the boundary line is arbitrary). However, we have now updated the text to specifically highlight the existence of such gradients (lines 306-308).

• The authors could stronger highlight the influence of stochasticity, which introduces novel behaviors absent in the deterministic case. For example, the third row of Supplementary Figure 28 powerfully illustrates how noise gives rise to new dynamical regions. This result is currently underemphasized, yet it may represent one of the most compelling findings of the study.

We agree that these results are very interesting, and thank the reviewer for highlighting them. We have now moved some of these results to a new main paper figure (Figure 4 in the updated text), which also now has an extended description in the main text (lines 275-282). This should better emphasise these results to the reader.

Minor comments and technical corrections

• Line 151: It is stated that the trajectory approaches one of the fixed points (disregarding noise). This is not true in the case of a stable limit cycle.

We agree. In the deterministic model, trajectories approach the system's attractor, which can be a stable fixed point or a stable limit cycle, so the original wording was imprecise. We have corrected the text accordingly (lines 166-172).

• Line 163: The list could be labeled with letters for easier reference to the panels in Figure 2.

Thanks for this suggestion. We have replaced the numbered list with a letter-based list (lines 182-204).

• Figure 3 (and multiple supporting figures): some of the legend boxes are hard to read to due the colored background (e.e. oscillations and stochastic switching). Consider only coloring the lines of the boxes, rather than filling them with color.

We agree that some of the legends are hard to read. We tried with just coloured boxes, but did not find it to be an improvement (see attached image). Instead, we have enlarged these boxes in the main text to make them easier to read. We have also updated the colour used for the "Oscillation" text, to increase the contrast and improve readability further.

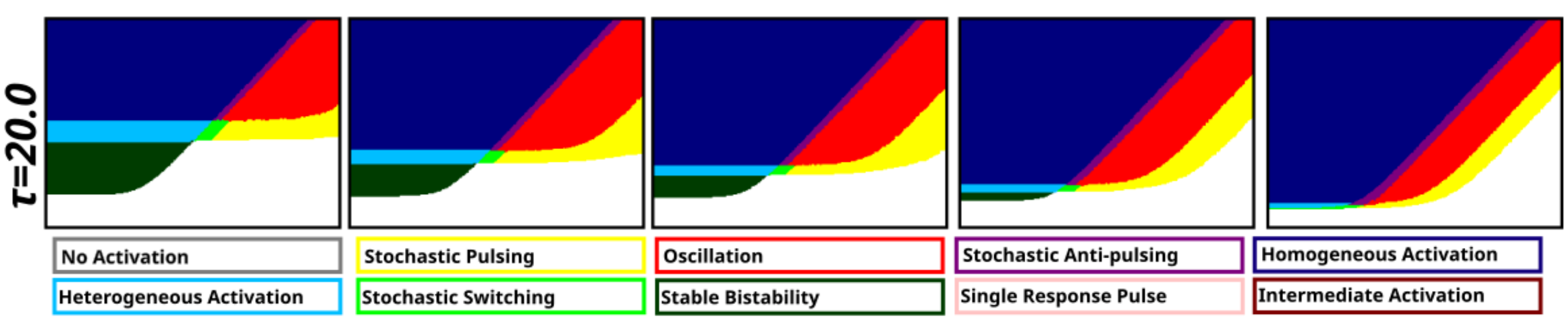

• Figure 5: caption a) is incomplete. It only describes one transition. caption c) "increasing IPTG concentration". It depends if one reads the figure top down or bottom up. Maybe replace with "changing IPTG concentration"? It took me a while to understand the color code of the boxes in b) and c). Maybe it would be easier to understand with a color scheme according the regime, rather than the points?

We have revised the Figure 5 caption to fully describe both transitions shown in panel (a). We have also replaced "increasing IPTG concentration" with "changing IPTG concentration" in panel (c) to avoid implying a reading direction. Regarding the colour code, the boxes are coloured using a continuous scale to reflect the continuous change in S (as IPTG is varied). The colours were selected to differ from those in the behaviour colour scheme to make the dots more distinct.

• Consider briefly comparing heterogeneous activation to clock reactions.

We are unsure exactly what the reviewer means here. However, we do reference circadian clocks in the main text, and the supplementary section contains extended discussions of biological examples of each behaviour.

• In the SI, line 110 contains an extra bracket: [[ˆσ]. Lines 114 and 119 omit the hat from ˆσ. Lines 128–132 lack hats entirely, although these parameters have dimensions. Also, in lines 130–132, kdeg is replaced by d. Moreover, the differential symbol d in the equations should be typeset upright (roman font), not italic. Line 136 replaces ˆI with ˆi.

We thank the reviewer for pointing out these details, and have fixed them in the updated version.

• The original dimensional model contains 9 parameters, 2 fundamental units, and 2 redundant parameters. Upon nondimensionalization and removal of these redundancies (e.g., ˆI and Kˆ), the system could be reduced to 5 in[1]dependent dimensionless parameters. However, the final model includes 6, due to the undocumented introduction of a noise strength parameter η. According to line 136, η is defined as η = 1/√vˆ, which raises concerns: if this relationship holds, then varying η would implicitly alter other parameters involving ˆv, compromising their inde[1]pendence. Furthermore, this definition makes the "dimensionless" model not truly dimensionless, as it introduces parameters that retain physical units: [η] = pmin/µM and [Wi] = pµM/min. A more consistent approach might be to define Wi = ˆkdegWˆi/√vˆ and introduce η in the dimensional equations, maintaining dimensional consistency without introducing an artificial parameter.

We agree that we assigned *η* the wrong value with respect to the non-nondimensionalised model's parameters, and thank the reviewer for pointing it out. We have now fixed this in the derivation of the non-dimensionalised model. We did not want to introduce eta in the dimensional equation, as this is not something which appears when the SDE is derived (using the chemical Langevin equation) from the fundamental reactions presented in Supplementary Table 1, but only during the non-dimensionalisation process. We also would not call the introduction of *η* undocumented, as we clearly state that it is introduced into the system at the moment of

nondimensionalisation (which is when it appears). However, for the benefit of the readers, we have added additional text to further point out when this is happening (supplementary lines 142-143).

• It would be helpful to include axis scales (e.g., labeled x- and y-axes) in all figures, including Supplementary Figures 5, 9, 15, 16, and 27–32, to improve clarity and interpretability.

Thanks for this suggestion. We have added additional axis labels to these figures.

• Supplementary Figure 7: Adding DDE simulation results for comparison, alongside the LCT, would strengthen the figure.

We agree, and have added an additional Figure (Supplementary Figure 10) comparing discrete delay and ODE Linear chain trick-based simulations.

• Supplementary Section 5: Consider adding a symbol to denote steady-state concentrations.

In the section, we mainly discuss steady states as solutions of a polynomial, rather than referring to the steady state concentrations directly. In line with the reviewer comment, we have now added a mention of the root value itself, along with designating it with a new symbol.

• Table captions should appear above the tables.

We have, both in supplementary and main text, changed all table captions to appear before the table, rather than after.

• In discussing the results: “For large values of η, the behaviour can also occur in the ($D \lesssim 1$) region, but this is likely rather a result of a very noisy system occasionally passing the threshold to being active, rather than a genuine pulsing behavior (Supplementary Figure 28).” — The mechanism behind the so-called ”genuine pulsing” behavior is not entirely clear. It appears to be similar to the case where the system occasionally crosses the activation threshold due to noise. As far as I can tell, the main difference lies in the height of the threshold, making such crossings less frequent, rather than in the underlying mechanism. Is there a qualitative distinction or fundamentally different process that separates this from what is described as ”genuine pulsing”?

Please see the attached figure of an example of pulsing and something which is a purely noisy fluctuation, hopefully it should clarify the difference. The phase-space plots from the SI where we discuss various behaviours might also be helpful (Supplementary Figure 20-29).

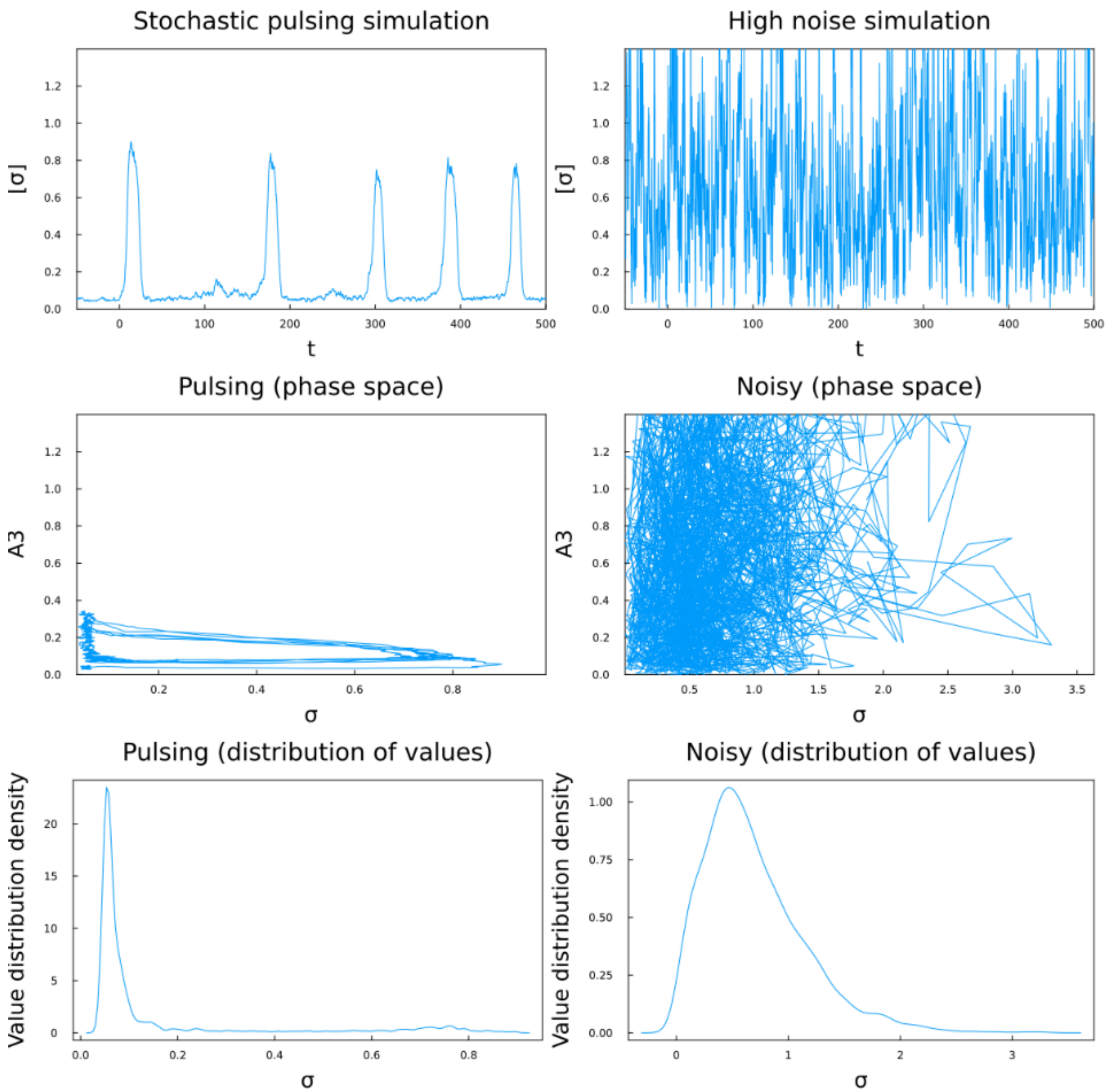

In practise, we find a threshold to determine when the system activates, if we increase the noise parameter enough, the system will just fluctuate so widely that this value is occasionally passed (top right figure). This is different from the pulsing behaviour where fluctuations are amplified by the system dynamics (excitability). You can see in the phase space plots that the excitable system (once noise is present) exhibits single oscillation cycles, which yield the pulses. The noisy system is instead distributed as a “blob”. If we look at the distribution of values encountered in both systems, the pulsing one has a single “peak” around the steady state, and then a fairly flat distribution of values along the height of the peaks. The noisy process is somewhere between a normal-like distribution that is skewed to one side (because it takes values below zero). We have updated the text to clarify this (Supplementary lines 723-728).

• In the behavior occurrence analysis, normalization was performed by dividing by the maximum value. Would it not be more appropriate to normalize by the sum of occurrences or by the total number of simulations? For example, if all 10 classes occurred with equal frequency across all parameters, this method would assign a normalized value of 1 to each—despite each appearing in only 10% of cases. If this interpretation is correct, it seems inconsistent with the expectation of low occurrence values for rarely observed classes. Additionally, it would be helpful to specify the parameter ranges used in the occurrence plots, the total number of simulations performed, and how many times each point in the parameter space was evaluated. These details were also unclear in the behavior maps.

We thank the reviewer for this point and agree the normalisation choice should be stated more clearly. In the figures concerned, we plot how each behaviour's occurrence depends on each parameter's value. The reason for our chosen normalisation is just to have each plot bounded by the range (0,1). That way, we can simplify the display of the plots. Given the large number of these individual plots (40 subfigures across 10 figures), we wanted to present everything in as non-cluttered a way as possible. To further make things easier for the reader, we now explicitly point this out in the text (Supplementary lines 906-907).

Finally, we note that the occurrence plots are performed directly on the behavioural map already computed (i.e. where we for each parameter combination have determined a specific behaviour). I.e. the same settings are used for classifying a parameter set as described in the previous setting. The parameter ranges considered are those described in the supplementary section "Behaviour occurrence analysis". We have modified the text to make this clearer (Supplementary lines 988-982).

• Supplementary Figure 27: Adding both stable and unstable periodic orbits to the bifurcation diagrams would aid interpretation. It is not obvious why the bifurcation diagrams for D = 1 and D = 5 appear qualitatively similar, yet their associated regions differ in the behavior maps. Conversely, between D = 5 and D = 25, a qualitative change in the bifurcation structure occurs, but the behavior map remains unchanged. Is this because the stochastic dynamics cause non-oscillatory regions to exhibit noise-induced oscillations, thereby widening the apparent oscillatory domain?

The bifurcation diagrams selected were not selected to describe 4 unique and distinct regions, but rather to give examples of how the bifurcation structure changes as D varies from low to high. We included 4 diagrams because that was how many diagrams we could fit along the figure without it becoming too cramped. We also note that the D = 1 and D = 5 diagrams are different: The D = 5 diagram contains a region with a single unstable steady state (just before the Hopf bifurcation), while in the D = 1 diagram, the unstable steady state always coexists with at least one stable steady state.

It would be possible to compute (and plot) the periodic orbits associated with each of the diagrams. However, these computations are rather involved and given the high number

of figures in the supplementary material, we aimed to keep each figure focused and visually interpretable. In this case, we have computed periodic orbits for multiple additional bifurcation diagrams, but the small size of these plots would mean the addition of additional trajectories and bifurcation points would make the plots substantially more cluttered. We have therefore expanded the figure caption to state explicitly that periodic orbits have been omitted from the plot (Supplementary lines 1038-1039).

• The self-activation parameter S was altered using a Heaviside step function to represent a sudden change in the input signal, resulting in a strong perturbation of the system. However, in many natural systems, input signals change more gradually rather than instantaneously. It would therefore be valuable to investigate how the system responds to smoother or more realistic input profiles, particularly in cases where the model yields a sharp, single-peak response resembling a Dirac delta function.

We agree that step inputs are an idealisation and that many natural inputs change more gradually. In accordance with the reviewer's suggestion, we have added an additional Supplementary section (13) and Figure (51) in which we test smoother input profiles in place of a Heaviside step. We find that the qualitative behaviours are largely preserved across a range of input smoothness, with the main effect being on the timing, amplitude and existence of transient responses (including the single-peak response regime).

• If multiple copies of a gene were inserted, how were they arranged? Facing in the same direction? As operon? Did you not observe recombination? Was the copy number stable? Related to this, in Table 3, the constructs are named for example "lacA::5xPsigV-rsiV NatR" while in the plasmid list it is "lacA::PsigV-5x rsiV NatR", suggesting that the promoter is only inserted once. Please extend the description of these multi-copy constructs.

Thank you for raising this. The plasmid list entry is a typo; the promoter is present upstream of each inserted copy, and we have corrected the naming in Table 3 and the plasmid list. Each multi-copy insertion consists of a tandem repeat of promoter–gene units (promoter–gene, promoter–gene, …) at a single genomic locus, with all repeats oriented in the same direction. These do not function as a single operon, since each gene copy has its own promoter. We did not observe obvious instability during strain propagation. We include new RT-qPCR measurements showing a graded suppression of σ^V expression with increasing *rsiV* copy number (new Supplementary Fig. 37), consistent with the intended multi-copy dosage design under our experimental conditions

• line 664 "Ellowitz" should be "Elowitz" line 664 "Ellowitz" should be "Elowitz"

Thanks for pointing this error out, we have now fixed it.

• Supplementary information pages 61/62: on several occasions it is written “um” instead of “µm”

Thanks for pointing this error out, we have now fixed it.

• With exception for some of the data in Fig. 4 and Fig. 5, the experimental system is mainly in the supporting infor[1]mation. The manuscript could be made more attractive to experimentalists if more illustrations of the experimental system would be in the main manuscript. For example snapshots of the mother machine and a small schemes of the sigma factor systems, so it is visually immediately clear which system (σ V vs B) was used and what parameters were varied (copy number, lysozyme concentrations)

We thank the reviewer for this suggestion and agree that additional visual context would make the experimental validation more immediately accessible. In the revised manuscript, we have reorganised and expanded the experiment-focused presentation in the main text. In particular, we split the original Figure 4 into two figures to improve clarity (now Figures 5 and 6), and we added representative snapshot images from a *sigV* microfluidics (mother-machine) experiment to make the experimental approach more visible. We have also revised the text/figure legends to more explicitly indicate which sigma factor system (σ^V versus σ^B) is used in each experiment and which parameters are varied.

• line 404: what is the (literature) half-life of your fluorescent marker? Does it contain a degradation tag?

We are using a Venus YFP reporter without a degradation tag, In our *B. subtilis* experiments, the effective decay is therefore expected to be dominated by dilution through cell division (i.e. an effective half-life on the order of the doubling time), rather than active degradation). It has a maturation time of 18 min (https://www.fpbase.org/protein/venus/).

Reviewer #3 (Remarks to the Author)

The authors present a theoretical and experimental analysis of a simple genetic circuit in bacteria cosnisting of a positive and negative feedback loops. The authors provide a comprehensive exploration of the dynamic behaviors such circuits can generate using a delayed stocatistic differential equation model and supported by detailed modeling and experimnetal validation in B subtilis using "in vivo" sigma/nati-sigma systems.

My main concern regards the level of originality of the findings. Indeed, the core circuit studied here is a very well-known topology, essentially an instance of the amplified negative feedback oscillator (a motif described extensively in the literature, including in reviews published over 15 years ago (e.g. https://royalsocietypublishing.org/doi/full/10.1098/rsif.2010.0183). While the mapping of behaviors across parameter space is interesting, the conceptual framework is not original. The behaviors demonstrated are those already known to arise from such network motifs. Indeed, when the negative feedback is weaker, bistability is expected. When the negative feedback gets stronger and delayed oscillations are expected, and intermediate behaviors are also expected. So, despite the thorough work, there is not enough novelty in my opinion.

We thank the reviewer for this thoughtful comment on the originality of the work. We agree that the mixed positive and negative feedback loop is a classic and widely studied circuit topology, and we chose it deliberately because it is a canonical regulatory architecture found across many biological contexts. Previous theoretical and experimental studies have shown that this motif can support a range of dynamical behaviours under different assumptions and parameter regimes. In the revised manuscript, we have substantially rewritten the Introduction, Results and Discussion to make clearer how our work differs from these earlier studies.

Our contribution is not to claim that these classes of behaviour are unexpected in this topology, or that it is surprising that parameter changes alter dynamics. Rather, the novelty of our work is that we provide a quantitative and experimentally testable framework for how a mixed positive/negative feedback loop generates trajectory-level behaviours in the noisy single-cell regime, and how transitions between those behaviours depend on effective circuit properties.

Specifically, we now make clearer that the manuscript:

(1) develops a minimal stochastic model in terms of a small number of biologically interpretable effective parameters, including feedback strengths, delay, noise amplitude and ultrasensitivity; (2) maps the ten trajectory-level dynamical regimes and their transitions across parameter space; (3) shows that many of these regime boundaries and intermediate transitions, including pulsatile regimes and their dependence on delay and noise, are not captured by simple qualitative intuition or deterministic steady-state analysis alone; and (4) validates this framework experimentally by rewiring alternative sigma-factor circuits and quantifying their behaviour using single-cell microfluidics time-lapse microscopy.

We agree that it is broadly expected that weaker negative feedback favours bistability, whereas stronger delayed negative feedback can favour oscillations. However, our results go beyond this general intuition by identifying the transition structure between these regimes in the stochastic single-cell setting, and by showing how noise reshapes behavioural boundaries and enables additional trajectory-level behaviours. In this sense,

our work provides a unified and experimentally testable transition map linking effective circuit properties to observable single-cell dynamics.

To further strengthen the revised manuscript, we have also added substantial new analysis and experiments. First, we now simulate alternative mixed-feedback circuit model formulations and show that they produce a similar range of behaviours (Supplementary Figs. 11 and 12), and we also test alternative input functions (Supplementary Fig. 51). Second, we have added a new main-text figure highlighting how stochasticity shifts behavioural boundaries in parameter space and enables additional behaviours. Third, we now introduce a neural-network-based single-trajectory classifier that permits consistent classification of both simulated and experimental time series (Supplementary Section 12). We validate this classifier on held-out data, with training and validation performed on separate experimental repeats (Supplementary Figures 44-49), and make the analysis available as a documented and tested software package on GitLab.

Importantly, we also extend the experimental validation using a third alternative sigma-factor system, σ^M. Despite not being trained on σ^M trajectories, or on experimental single-response-pulse trajectories, the classifier successfully identifies the corresponding behaviour classes in this independent dataset (Supplementary Figure 50). We believe this provides a strong test of classifier generalizability and broadens the experimental support for the model.

We hope these revisions and additions make the novelty and scope of the work much clearer.

Reviewer #4 (Remarks to the Author)

I co-reviewed this manuscript with one of the reviewers who provided the listed reports. This is part of the Nature Communications initiative to facilitate training in peer review and to provide appropriate recognition for Early Career Researchers who co-review manuscripts.

Thank you for co-reviewing
